# Direct asymmetric N-propargylation of indoles and carbazoles catalyzed by lithium SPINOL phosphate

**DOI:** 10.1038/s41467-019-13886-9

**Published:** 2020-01-13

**Authors:** Yingcheng Wang, Sheng Wang, Wenyu Shan, Zhihui Shao

**Affiliations:** Key Laboratory of Medicinal Chemistry for Natural Resource, Ministry of Education, School of Chemical Science and Technology, State Key Laboratory for Conservation and Utilization of Bio-Resources in Yunnan, Yunnan University, Kunming, 650091 China

**Keywords:** Asymmetric catalysis, Homogeneous catalysis, Synthetic chemistry methodology

## Abstract

Catalytic asymmetric functionalization of the N–H groups of indoles and carbazoles constitutes an important but less developed class of reactions. Herein, we describe a propargylation protocol involving the use of a lithium SPINOL phosphate as the chiral catalyst and our recently developed C-alkynyl N,O-acetals as propargylating reagents. The direct asymmetric N-propargylation of indoles and carbazoles provides hitherto inaccessible N-functionalized products. Notably, the efficiency of the system allows reactions to be run at a very low catalyst loading (as low as 0.1 mol%). Mechanistic information about the titled reaction is also disclosed. This study represents an advance in the direct asymmetric functionalization of the N–H bonds of indoles and carbazoles, and additionally expands on the application of chiral alkali metal salts of chiral phosphoric acids in asymmetric catalysis.

## Abstract (web summary)

Asymmetric functionalization of N–H bonds constitutes an important but less developed class of reactions. Here, the authors report the asymmetric direct N-propargylation of indoles and carbazoles with a lithium SPINOL phosphate as the chiral catalyst and shed light on the reaction mechanism.

## Introduction

The synthesis of enantioenriched indoles is of wide interest, owing to their prevalence in natural products and pharmaceuticals, and agrochemicals. Therefore, extensive efforts have been devoted to developing catalytic asymmetric methods for the functionalization of indoles^1,2^. Great progress has been made in the catalytic asymmetric C-alkylation, especially at the C3 position, due to the innate nucleophilicity of C3 of the indole ring. In contrast, the catalytic asymmetric functionalization of the N–H of indole, particularly in an intermolecular manner, has remained underdeveloped^3–16^, owing to the mitigated nucleophilicity of this position. To avoid the regioselectivity issue, several indirect strategies have also been elegantly designed^17–21^. To date, efficient strategies for the asymmetric N-functionalization of indoles are still rather limited and most reported intermolecular reactions relied on N-allylation. Very recently, the catalytic asymmetric N-benzylation has been elegantly developed to afford chiral N-benzylic indoles^22–27^. Nevertheless, despite these elegant achievements, there is still no report for the direct catalytic asymmetric functionalization of the N–H of indole through a propargylation strategy.

Catalytic asymmetric propargylation has been recognized as an important class of reactions^28^ in organic chemistry, because they create a propargylic chiral center and introduce a synthetically versatile and biologically important alkyne functional group in one step^29^. Although great efforts have been devoted in this area, the potential of catalytic asymmetric propargylations has not been fully exploited and the scope of applicable nucleophiles and electrophiles is still rather limited. The direct catalytic asymmetric N-propargylation of indoles and carbazoles as nucleophiles has not yet been achieved. In addition, there are only limited types of chiral catalyst systems for the asymmetric propargylation to date.

**Fig. 1:**
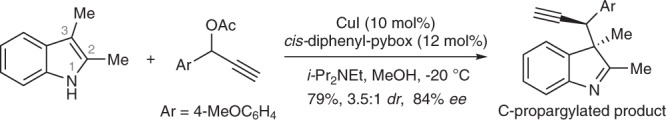
Cu-catalyzed asymmetric propargylation of 2,3-dimethyl indole. C-propargylated product was exclusively obtained.

The direct catalytic asymmetric N-propargylations using 1H-indole derivatives as nucleophiles have proven far more challenging than allied reactions such as allylations and have not been successful. Indeed, You and colleagues^30,31^ studied the reaction of 2,3-dimethyl indole with propargylic acetates using a chiral copper–pybox complex as the catalyst, but only obtained the C-propargylated product (Fig. 1). Thus, the challenges in the development of the direct catalytic asymmetric N-propargylation of indoles and carbazoles as the nucleophiles are to find a suitable propargylating reagent and an efficient catalyst system.

**Fig. 2:**
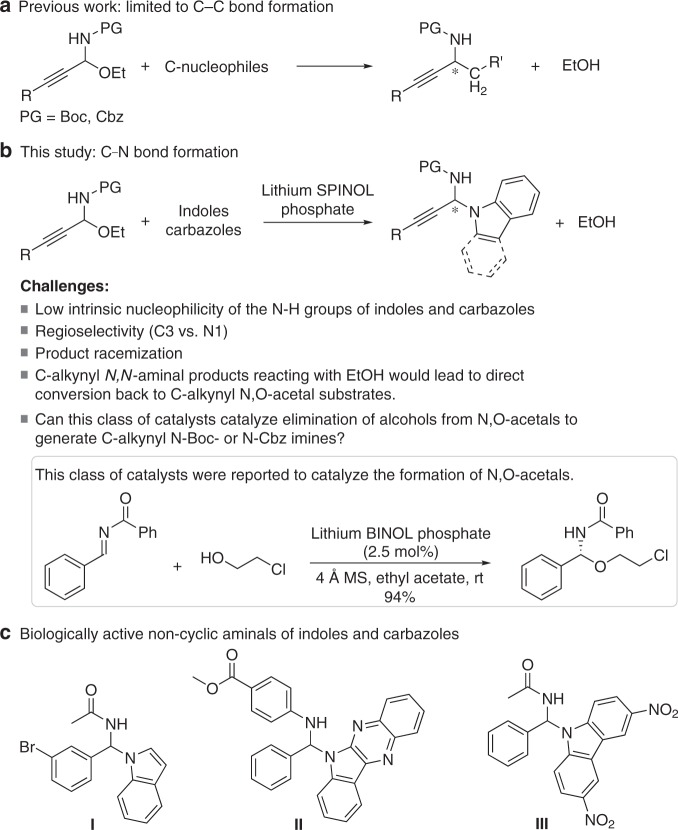
Asymmetric reactions of C-alkynyl N,O-acetals with nucleophiles. **a** C-based nucleophiles. **b** 1H-Indoles and carbazoles as N-based nucleophiles (several challenges are shown). **c** Non-cyclic aminals of indoles and carbazoles as antibiotics. Boc, *t*-butyloxy carbonyl, Cbz benzyloxycarbonyl, PG protecting group.

We recently introduced C-alkynyl N-Boc- and N-Cbz-protected N,O-acetals as a class of coupling partners for asymmetric catalytic transformations; to date, these transformations have been limited to the formation of C–C bonds with the use of carbon-based nucleophiles (Fig. 2a)^32–35^. However, the corresponding asymmetric reaction of C-alkynyl N,O-acetals through carbon–heteroatom bond formation has remained an unmet task. Given the lack of direct methods available for the catalytic asymmetric N-propargylation of indoles and carbazoles, we chose to explore the possibility that 1H-indoles and carbazoles^36^ might serve as the first effective heteroatom-based nucleophiles to react with C-alkynyl N,O-acetals (Fig. 2b). Such reaction would provide straightforward access to chiral alkynylated acyclic N,N-aminals of indoles and carbazoles. Recent studies have shown that acyclic N,N-aminal indoles **I**–**III** have significant antibiotic properties (Fig. 2c)^37–40^. Thus, there is a need for organocatalytic methods that enable the enantioselective synthesis of acyclic N,N-aminals of indoles or carbazoles, biologically important yet synthetically challenging molecules bearing an acyclic N,N-substituted α-chiral carbon center on the N1-position of indoles or carbazoles. Meanwhile, incorporating alkynes into N,N-aminals is made interesting, owing to the versatile transformations of the alkyne group and the ubiquitous occurrence as a key structural motif in natural products and pharmaceuticals. However, the direct catalytic asymmetric synthesis of N,N-aminals through a propargylation method has not been reported.

On the other hand, chiral alkali and alkaline earth metal-derived salts of chiral phosphoric acids have recently emerged as a class of effective catalysts in various asymmetric transformations^41–60^. However, this class of chiral catalysts have not been successfully employed in the catalytic asymmetric functionalization of the N–H of indoles or carbazoles. Meanwhile, the direct catalytic asymmetric propargylation reaction catalyzed by chiral alkali or alkaline earth-derived salts of chiral phosphoric acids has remained elusive.

Besides these, there are several other challenges for the catalytic enantioselective C–N bond formation between indoles and carbazoles, and C-alkynyl N,O-acetals. C-alkynyl N,N-aminals have been reported to react with EtOH to form the corresponding C-alkynyl N,O-acetals^61^. Thus, unlike the products generated by C-based nucleophiles, for the products produced by N-centered nucleophiles, there is a risk of a direct conversion of newly formed C-alkynyl N,N-aminal products back to the C-alkynyl N,O-acetals (starting materials). In addition, C-alkynyl N,N-aminals reacting with EtOH would also lead to product racemization. Second, due to the low intrinsic nucleophilicity of the N–H motif of indoles and carbazoles, together with low reactivity of C-alkynyl N-Boc or N-Cbz N,O-acetals, high reaction temperature might be needed for the N-propargylation, which cause difficulties in selective control. Moreover, the C3 position of the indole could compete with the nitrogen atom as the nucleophile, as exemplified by You and colleagues^30^ in the catalytic asymmetric indole C-propargylation reaction. Finally, chiral alkali and alkaline earth salts of chiral phosphoric acids have been shown to efficiently catalyze the addition of alcohols to imines, to form the corresponding N,O-acetals (see Fig. 2b)^57^. In contrast, can this class of catalysts catalyze the elimination of alcohols from N,O-acetals to generate the corresponding C-alkynyl N-Boc or N-Cbz imines? Unlike C-aryl N-Boc- or N-Cbz-protected imines, C-alkynyl N-Boc- or N-Cbz-protected imines cannot be prepared by existing methods. Such imines have also proven more difficult to be generated by the traditional methods. Amidosulfones^62–67^, which are widely used imine precursors, are not suitable for this purpose. This process must overcome the potential 1,4-addition onto the alkynyl imines^68^.

Herein, we report the development of highly enantioselective direct catalytic asymmetric N-propargylation of indoles and carbazoles. Mechanistic investigations are also disclosed.

## Results

### Reaction development

We first explored the N-propargylation of carbazole **2a** with our C-alkynyl N,O-acetal **1a** (Table 1). Chiral bifunctional Brønsted base catalysts such as **BB1** and **BB2**, which have been shown to be efficient in the asymmetric reaction of C-alkynyl N,O-acetals with carbon-based nucleophiles^32^, failed to promote the reaction, while chiral phosphoric acids^33^ promoted the model reaction between C-alkynyl N,O-acetal **1a** and carbazole **2a** but with very poor enantioselectivity (Table 1, entries 1 and 2). These results highlight the challenges of developing the proposed catalytic asymmetric N-propargylation. These results prompted us to identify an alternative organocatalyst that must be capable of both catalyzing the EtOH elimination from N,O-acetals to generate challenging N-Cbz-protected C-alkynyl imines and promoting the subsequent N-propargylation reaction, as well as imposing effective stereocontrol. After extensive investigations, we found that chiral alkali and alkaline earth metal-derived salts of chiral phosphoric acids were promising chiral catalysts for the tandem process combining the in situ generation of C-alkynyl N-Cbz imines and N-propargylation. Among them, a lithium SPINOL phosphate, Li[**P2**], catalyzed the EtOH elimination from N,O-acetal **1a** for in situ generation of difficult accessible C-alkynyl N-Cbz imines and the subsequent asymmetric N-propargylic alkylation with 93% enantiomeric excess (*ee*) (Table 1, entry 3).

**Table 1:** Optimization of the reaction conditions^a^.


Entry	Catalyst	T [^o^C]	Yield [%]^b^	*ee* [%]^c^
1	PA1 washed with HCl	70	68	−5
2	PA2 washed with HCl	70	66	7
3	Li[P2]	90	70	93
4	Na[P2]	90	70	19
5	K[P2]	90	68	22
6	Mg[P2]_2_	90	71	74
7	Ca[P2]_2_	90	67	2
8	Li[P3]	90	21	70
9	Li[P4]	90	72	39
10	Li[P5]	90	69	24
11	Li[P6]	90	38	16
12	Li[P7]	90	69	21
13	Li[P8]	90	64	15
14	Li[P9]	90	58	10
15	Li[P10]	90	71	0
16	Li[P11]	90	43	−11
17	Li[P1]	90	76	−48
18	Mg[P3]_2_	90	74	5
19	Mg[P4]_2_	90	71	3
20	Mg[P5]_2_	90	68	4

^a^Reaction conditions: **1a** (0.08 mmol), **2a** (0.05 mmol), toluene (1 mL)

^b^Yield of isolated product

^c^Determined by HPLC analysis on a chiral stationary phase

### Mechanistic investigations

**Fig. 3:**
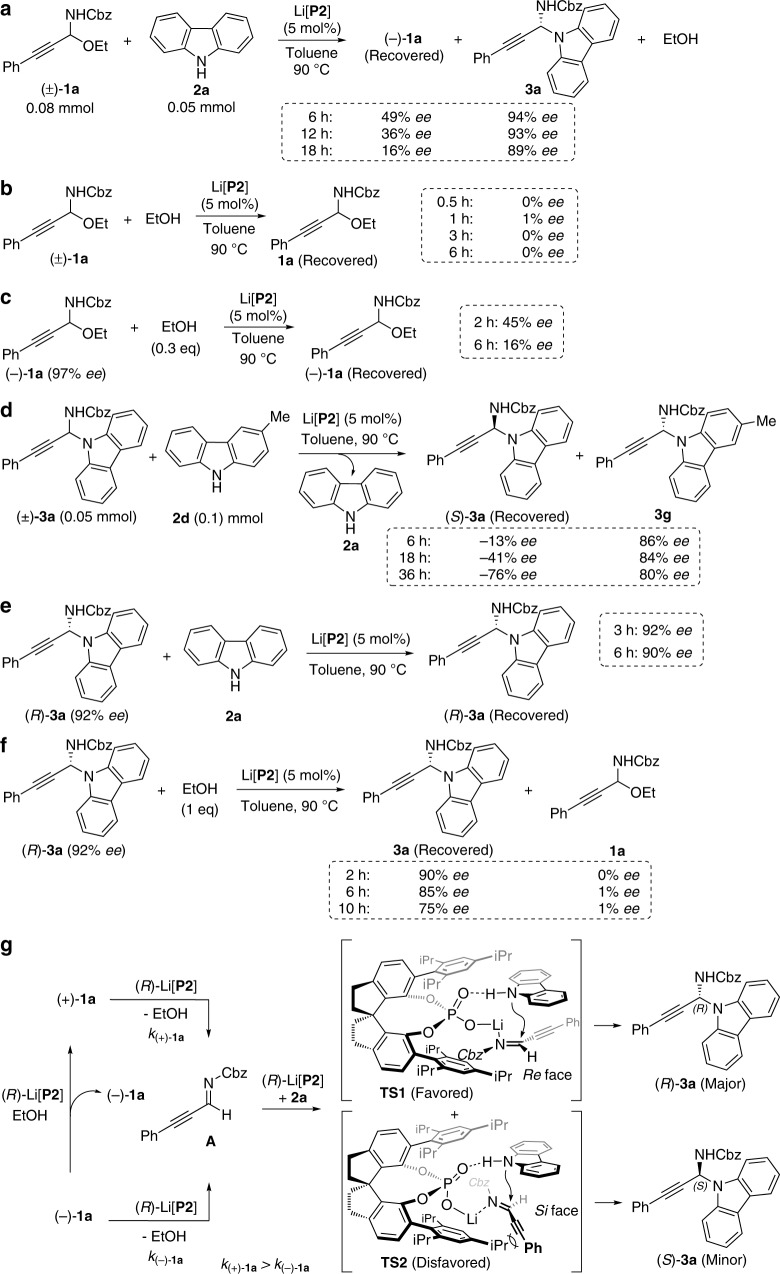
Mechanistic investigations. **a** Enantioselectivity of the product **3a** and the recovered substrate **1a** over time. **b** The reaction of racemic **1a** with EtOH under the chiral catalysis of Li[**P2**]. **c** The reaction of chiral **1a** with EtOH under the chiral catalysis of Li[**P2**]. **d** Crossover experiment. **e** The reaction of chiral **3a** with **2a** under the chiral catalysis of Li[**P2**]. **f** The reaction of chiral **3a** with EtOH under the chiral catalysis of Li[**P2**]. **g** Proposed reaction mechanism.

To get an understanding of the N-propargylation, we monitored the reaction of racemic **1a** with **2a** under the standard conditions by determining *ee* for both the generated product **3a** and the recovered substrate **1a** over time (Fig. 3a). It was found that after a reaction time of 6 h, **1a** was recovered with 49% *ee*. With the reaction time further increasing, *ee* of the recovered **1a** began to decrease. Meanwhile, *ee* of the product **3a** was decreased relatively slowly (for details, see Supplementary Table 2).

**Fig. 4:**
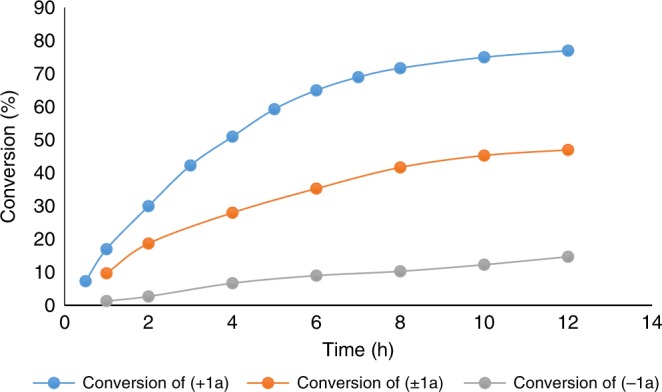
Kinetic profile. The reaction with (+)-**1a** was faster than that of (−)-**1a**.

To investigate whether the enantioriched **1a** arised from the reaction of racemic **1a** with EtOH under the chiral catalysis of Li[**P2**], we performed the reaction between racemic **1a** and EtOH in the presence of (*R*)-Li[**P2**]. The recovered **1a** was still racemic and no chiral induction was observed (Fig. 3b), thus excluding such possibility. This result combined with the experiment shown in Fig. 3a implied there might be a kinetic resolution during the process between racemic **1a** and **2a** in the presence of Li[**P2**]. To further confirm this issue, we conducted kinetic studies. The (+)-**1a** (97% *ee*) and (−)-**1a** (97% *ee*) were reacted with **2a** in the presence of (*R*)-Li[**P2**] as the catalyst, respectively. We found that both enantiomers of **1a** delivered the same enantiomer of the product, (*R*)-**3a**, regardless of the original configuration of the C-alkynyl N,O-acetal, and the reaction with (+)-**1a** was faster than that of (−)-**1a** (Fig. 4. For details, see Supplementary Table 3), thus verifying that there was a moderate degree of kinetic resolution of **1a** during the reaction with **2a**.

To understand why the recovered **1a** and the product **3a** decreased gradually with the reaction time further increasing and to gain further insight into the N-propargylation, we made some control experiments. The experiment shown in Fig. 3c (for details, see Supplementary Tables 4 and 5) explained a possible reason for the decrease of enantioselectivity of the recovered **1a** over the time. The crossover experiment shown in Fig. 3d (for details, see Supplementary Table 6) indicated that the chiral catalyst was discriminating the two enantiomers of the C-alkynyl N,N-aminal and *(R)***-3a** was converted into the corresponding C-alkynyl N-Cbz imine more faster than *(S)***-3a** in the presence of Li[**P2**] (there was a kinetic resolution of **3a** during this process). These results combined with the control experiments in Fig. 3e (for details, see Supplementary Table 7) and Fig. 3f (for details, see Supplementary Table 8) explained a possible reason for the decrease of *ee* of the product **3a** over the time. The experiment in Fig. 3f also suggested that the alkynylated N,N-aminal can react with EtOH to form the corresponding N,O-acetal. Pleasingly, the racemization of the product **3a** was found to be effectively suppressed by increasing the amount of the C-alkynyl N,O-acetal substrate **1a** to 1.6 eq. (for details, see Supplementary Table 9).

Next, we studied the relationship between the *ee* of the catalyst Li[**P2**] and that of the product **3a**^69^, and a linear effect was observed (for details, see Supplementary Table 10). On the basis of the above observations, we proposed a possible reaction mechanism shown in Fig. 3g.

### Substrates scope

**Fig. 5:**
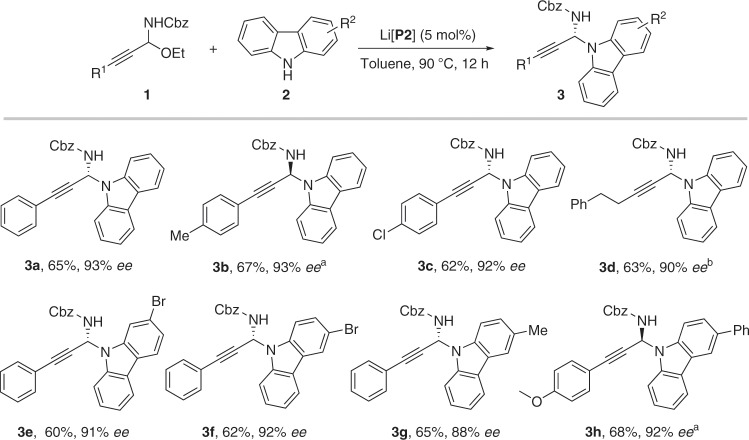
Scope of N-propargylation of carbazoles. Reaction conditions: **1** (0.08 mmol), **2** (0.05 mmol), (*R*)-Li[**P2**] (5 mol%), toluene (1 mL). **a** Run with (*S*)-Li[**P2**]. **b** Run at 100 °C.

**Fig. 6:**
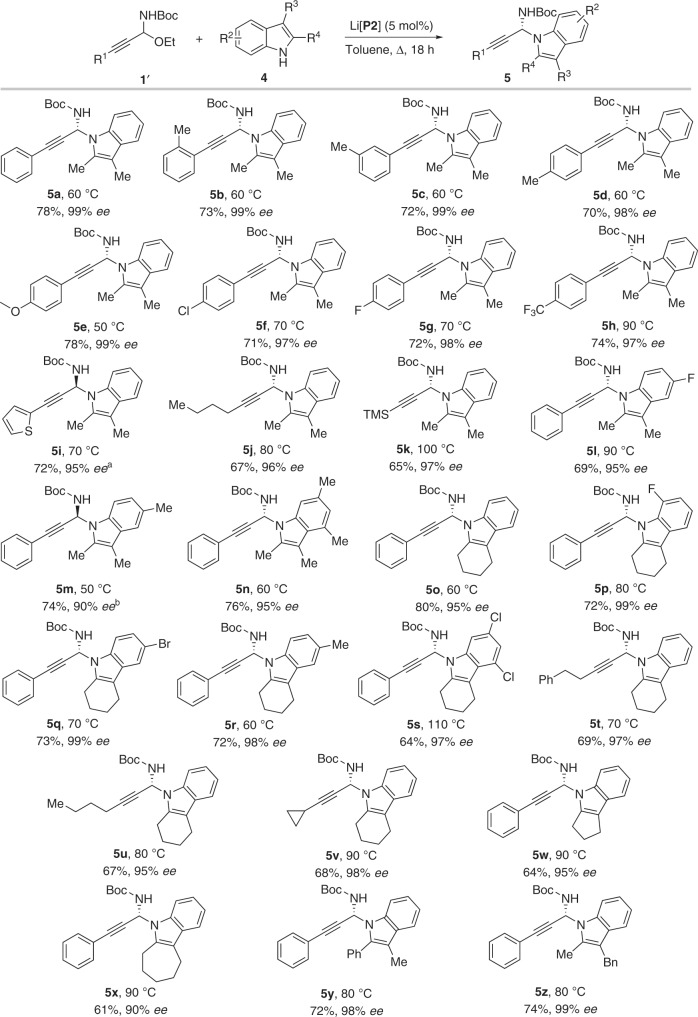
Scope of N-propargylation of indoles. Reaction conditions: **1'** (0.05 mmol), **4** (0.08 mmol), (*R*)-Li[**P2**] (5 mol%), toluene (1 mL). **a** Run with (*S*)-Li[**P2**]. **b** Run with (*R*)-Li[**P1**].

With the optimized chiral catalyst and suitable propargylic alkylating reagent in hand, we investigated the scope of the direct catalytic asymmetric N-propargylation of carbazoles. Various C-alkynyl N-Cbz N,O-acetals **1** were reacted with carbazoles **2** to afford the desired N-propargylated products (**3a**–**3h**) in high enantioselectivities with good yields (Fig. 5). Moreover, this system was also applicable for the enantioselective N-propargylation of indoles (Fig. 6). The reaction displayed broad substrate scope of both C-alkynyl N,O-acetals and substituted indoles. The corresponding N-propargylic indoles **5a–z** were obtained in good yields with high to excellent enantioselectivities (up to 99% *ee*) in all the cases examined (26 examples). Notably, in contrast to the work by You and colleagues^30^, our propargylation reactions occurred at the N1-position exclusively and no competing C3-propargylaed product was observed. On the other hand, impressively, the reaction at high temperature (110 °C) can still provide excellent enantioselectivity (**5s**). Examples with high stereocontrol at such a high temperature are scarce in asymmetric catalysis by chiral alkali and alkaline earth-derived salts of chiral phosphoric acids.

### Product configuration determination and transformations

**Fig. 7:**
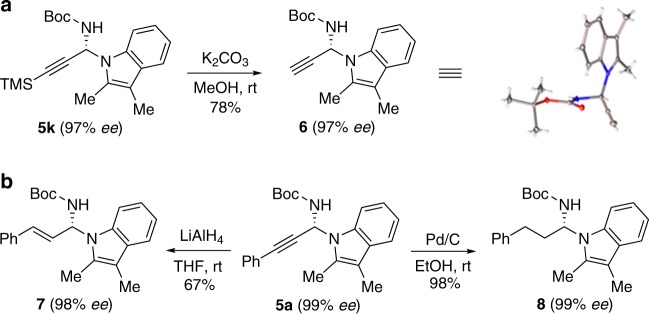
Product configuration determination and transformations. **a** Product configuration determination by X-ray diffraction analysis. **b** Reduction of the alkynyl group into the alkenyl and alkyl groups.

The absolute configuration of the N-propargylation products was determined by converting **5k** into **6** (Fig. 7a), and the structure of **6** was unambiguously confirmed through X-ray crystal analysis. Reduction of **5a** with LiAlH_4_ afforded **7**, which is formally derived from the N-selective and 1,2-selective addition of α,β-unsaturated imine (Fig. 7b). When **5a** was reduced with Pd/C under H_2_ atmosphere, the primary alkyl-substituted product **8** was obtained in quantitative yield.

### Low catalyst-loading experiments

Promoted by the unusual stereocontrol of Li[**P2**], we examined the N-propargylation reaction with low catalyst loading (Table 2). We found that when the catalyst loading was decreased from 5 to 0.5 mol%, the yield and enantioselectivity were not affected (Table 2, entry 3 vs. entries 1 and 2). Further decreasing the catalyst loading to 0.2 mol% has only a slight impact on the enantioselectivity (Table 2, entry 4). Even 0.1 mol% catalyst loading can still provide high enantioselectivity and yield (Table 2, entry 5). This is the lowest catalyst loading that has been achieved so far for the asymmetric catalysis by chiral alkali and alkaline earth-derived salts of chiral phosphoric acids^70^.

**Table 2:** Reaction with low catalyst loading.


Entry	*X*	Yield [%]	*ee* [%]
1	5	78	99
2	1	78	99
3	0.5	76	99
4	0.2	76	97
5	0.1	72	92

### N-benzylation of indoles

**Fig. 8:**
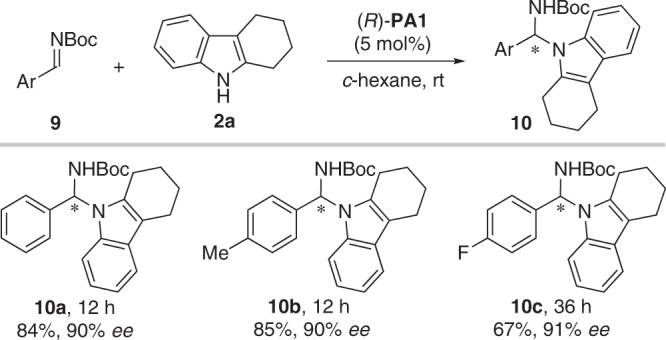
N-Benzylation of indoles. Reaction conditions: **9** (0.05 mmol), **2a** (0.06 mmol), (*R*)-**PA1** (5 mol%), *c*-hexane (1.0 mL), rt.

Finally, we explored the direct catalytic asymmetric N-benzylation of indoles with C-aryl N-Boc N,O-acetals in the presence of (*R*)-Li[**P2**]. Interestingly, the reaction did not occur. These results indicate the reactivity difference between C-alkynyl N-Boc N,O-acetals and C-aryl N-Boc N,O-acetals. Considering that C-aryl N-Boc imines can be prepared, we turned our attention to the use of C-aryl N-Boc imines preformed. By utilizing a complementary catalytic mode, hydrogen bonding enantiocontrol, we developed an enantioselective N-benzylation of indoles using a chiral phosphoric acid **PA1** as the catalyst (Fig. 8).

## Discussion

In summary, we have developed an organocatalytic strategy for the direct asymmetric N-functionalization of indoles and carbazoles through a propargylation. Specifically, we have successfully realized the direct catalytic asymmetric intermolecular N-propargylation of indoles and carbazoles, providing hitherto inaccessible acyclic N,N-aminals of indoles and carbazoles in good yields with high enantiocontrol, despite many potential challenges of this reaction. This C–N formation process is enabled by the use of our newly developed C-alkynyl N,O-acetals as the alkylating reagent and a lithium SPINOL phosphate as the chiral catalyst. The product racemization has been suppressed effectively. A chiral lithium SPINOL phosphate catalyst promotes the activation of N,O-acetals for the in situ formation of challenging accessible C-alkynyl N-Boc or N-Cbz imines, although this class of catalysts were previously employed for the addition of alcohols to imines to form N,O-acetals. Notably, the efficiency of the system allows reactions to be run at a very low catalyst loading of 0.1 mol%. The present protocol represents a significant advance in the asymmetric functionalization of the N–H of indoles and carbazoles, and in the catalytic asymmetric propargylation, as well as opens an application of chiral alkali and alkaline earth-derived salts of chiral phosphoric acids in the asymmetric catalysis and synthesis.

## Methods

### General procedure for the N-propargylation of carbazoles **2**

To a solution of **1** (0.08 mmol) and **2** (0.05 mmol) in toluene (1.0 mL) was added the catalyst (*R*)*-*Li[**P2**] (1.8 mg, 5 mol %) at 90 °C. After stirring for 12 h, the mixture was directly purified by silica gel chromatography (ethyl acetate/petroleum ether = 1/30 to 1/20) to afford the products **3**.

### General procedure for the N-propargylation of indoles **4**

To a solution of **1’** (0.05 mmol) and **4** (0.08 mmol) in toluene (1.0 mL) was added the catalyst (*R*)*-*Li[**P2**] (1.8 mg, 5 mol%) at the designated temperature. After stirring for 18 h, the mixture was directly purified by silica gel chromatography (ethyl acetate/petroleum ether = 1/100 to 1/50) to afford the products **5**.

## Supplementary information

Supplementary Information

## Data Availability

The authors declare that the data supporting the findings of this study are available within the article and the Supplementary Information, as well as from the authors upon reasonable request. The X-ray crystallographic coordinate for structure **6** reported in this study has been deposited at the Cambridge Crystallographic Data Centre (CCDC), under CCDC 1881884. These data can be obtained free of charge from The Cambridge Crystallographic Data Centre via www.ccdc.cam.ac.uk/data_request/cif.
