# Supplementary material for: Direct asymmetric N-propargylation of indoles and carbazoles catalyzed by lithium SPINOL phosphate
Source: Nat Commun. 2020 Jan 13;11:226. doi: 10.1038/s41467-019-13886-9 (PMC6957506; doi:10.1038/s41467-019-13886-9)
Supplement: Supplementary file 1 — Supplementary Information [file 41467_2019_13886_MOESM1_ESM.pdf]

Supplementary Information

Direct Asymmetric N-Propargylation of Indoles and Carbazoles Catalyzed by Lithium SPINOL Phosphate

Wang *et al.*

Supplementary Note 1

General information

^{1}H-NMR and ^{13}C-NMR spectra were recorded at 400 MHz and 600 MHz spectrophotometer. Chemical shifts (δ) are expressed in ppm, and J values are given in Hz. The enantiomeric excess was determined by chiral HPLC with *n*-hexane and *i*-propanol as eluents. High resolution mass spectrometry (HRMS) was recorded on a VG Auto Spec-3000 spectrometer. Optical rotations were measured on a JASCO DIP-370 polarimeter.

All chemicals were used as received without further purification unless otherwise stated. All solvents were purified and dried by standard techniques, and distilled prior to use. Flash column chromatography was performed on silica gel (200–300 mesh).

Supplementary Note 2

General procedure for the N-propargylation of carbazoles 2 with C-alkynyl N,O-acetals 1.

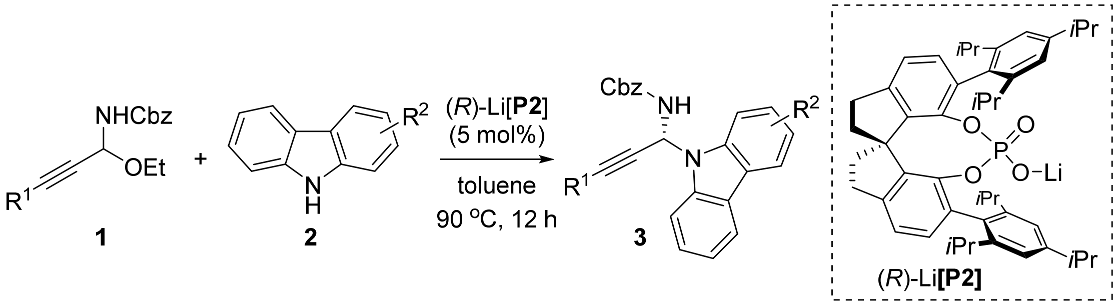

To a solution of **1** (0.08 mmol) and **2** (0.05 mmol) in toluene (1.0 mL) was added the catalyst (*R*)-Li[**P2**] (1.8 mg, 5 mol%) at 90 °C. After stirring for 12 h, the mixture was directly purified by silica gel chromatography (ethyl acetate:petroleum ether = 1:30 to 1:20) to afford the products **3**.

Benzyl (*R*)-(1-(9H-carbazol-9-yl)-3-phenylprop-2-yn-1-yl)carbamate (3a)

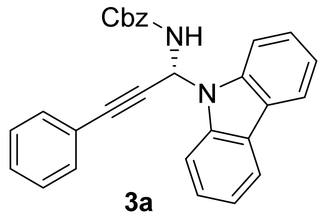

3a

Compound **3a** was obtained as colorless liquid (14.0 mg, 65% yield, 93% ee) after flash chromatography (elution gradient: ethyl acetate:petroleum ether = 1:20); **[α]$_D$^{25}** = + 59.3 (*c* 1.0, $CHCl_3$); **^{1}H-NMR** (400 MHz, $CDCl_3$, ppm): *δ* 8.10 (d, *J* = 8.0 Hz, 2H), 7.88 (d, *J* = 7.6 Hz, 2H), 7.52-7.41 (m, 5H), 7.35-7.27 (m, 10H), 6.21 (d, *J* = 7.2 Hz, 1H), 5.17 (d, *J* = 12.0 Hz, 1H), 4.96 (d, *J* = 12.0 Hz, 1H); **^{13}C-NMR** (100 MHz, $CDCl_3$, ppm): *δ* 155.2, 138.9, 135.6, 132.0, 129.3, 128.7, 128.5, 126.1, 123.9, 121.4, 120.5, 120.3, 110.4, 86.5, 83.1, 67.8, 53.3; **HRMS** calcd. for $C_{29}H_{22}N_2NaO_2$ $[M+Na]^+$: 453.1573, found: 453.1573; **HPLC analysis:** Daicel CHIRALPAK AD-H, *n*-hexane:*i*-PrOH = 80:20, flow rate = 0.8 mL·min^{-1}, λ = 254 nm, retention time: t_R = 13.0 min (minor), t_R = 13.8 min (major).

Benzyl (*S*)-(1-(9H-carbazol-9-yl)-3-(*p*-tolyl)prop-2-yn-1-yl)carbamate (3b)

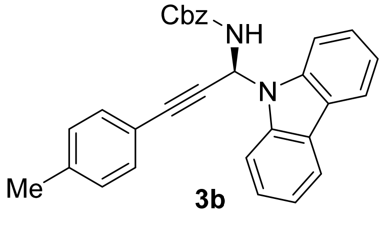

3b

In the presence of (*S*)-Li[**P2**] catalyst, compound **3b** was obtained as colorless liquid (14.9 mg, 67% yield, 93% ee) after flash chromatography (elution gradient: ethyl acetate:petroleum ether = 1:20); **[α]$_D$^{25}** = - 28.7 (*c* 1.0, $CHCl_3$); **^{1}H-NMR** (600 MHz, $CDCl_3$, ppm): *δ* 8.11 (d, *J* = 7.8 Hz, 2H), 7.91 (d, *J* = 6.0 Hz, 2H), 7.53-7.52 (m, 3H), 7.33-7.30 (m, 9H), 7.13 (d, *J* = 7.8 Hz, 2H), 6.25 (br, 1H), 5.18 (d, 1H, *J* = 11.4 Hz), 4.97 (d, *J* = 11.4 Hz, 1H), 2.36 (s, 3H); **^{13}C-NMR** (150 MHz, $CDCl_3$, ppm): *δ* 155.2, 139.6, 138.9, 135.7, 131.9, 129.3, 128.7, 128.5, 128.5, 126.1, 123.9, 120.5, 120.2, 118.3, 110.5, 86.7, 82.4, 67.7, 53.4, 21.6; **HRMS** calcd. for $C_{30}H_{24}N_2NaO_2$ $[M+Na]^+$:

467.1730, found: 467.1731; **HPLC analysis:** Daicel CHIRALPAK AD-H, *n*-hexane:*i*-PrOH = 80:20, flow rate = 0.8 mL·min^{-1}, λ = 254 nm, retention time: t_R = 11.0 min (major), t_R = 13.2 min (minor).

Benzyl (*R*)-(1-(9H-carbazol-9-yl)-3-(4-chlorophenyl)prop-2-yn-1-yl)carbamate (3c)

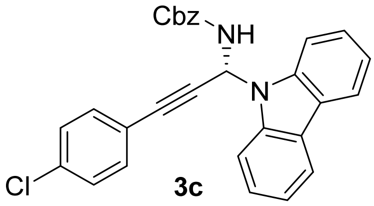

3c

Compound **3c** was obtained as colorless liquid (14.4 mg, 62% yield, 92% ee) after flash chromatography (elution gradient: ethyl acetate:petroleum ether = 1:20); $[\alpha]_D^{25}$ = + 25.7 (*c* 1.0, $CHCl_3$); **^{1}H-NMR** (600 MHz, $CDCl_3$, ppm): *δ* 8.11 (d, *J* = 7.8 Hz, 2H), 7.87 (d, *J* = 6.0 Hz, 2H), 7.51 (br, 3H), 7.35-7.28 (m, 11H), 6.23 (d, *J* = 5.4 Hz, 1H), 5.18 (d, *J* = 11.4 Hz, 1H), 4.97 (d, *J* = 11.4 Hz, 1H); **^{13}C-NMR** (150 MHz, $CDCl_3$, ppm): *δ* 155.2, 138.8, 135.5, 133.2, 128.9, 128.7, 128.6, 128.5, 126.1, 123.9, 120.6, 120.3, 119.9, 110.3, 85.3, 84.1, 67.8, 53.3; **HRMS** calcd. for $C_{29}H_{21}ClN_2NaO_2$ $[M+Na]^+$: 487.1184, found: 487.1184; **HPLC analysis:** Daicel CHIRALPAK AD-H, *n*-hexane:*i*-PrOH = 80:20, flow rate = 0.8 mL·min^{-1}, λ = 254 nm, retention time: t_R = 13.4 min (minor), t_R = 16.3 min (major).

Benzyl (*R*)-(1-(9H-carbazol-9-yl)-5-phenylpent-2-yn-1-yl)carbamate (3d)

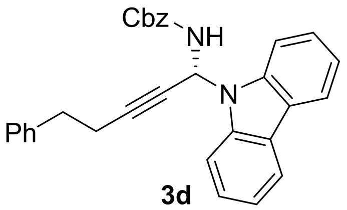

3d

The reaction was conducted at 100 °C, compound **3d** was obtained as colorless liquid (14.4 mg, 63% yield, 90% ee) after flash chromatography (elution gradient: ethyl acetate:petroleum ether = 1:20); $[\alpha]_D^{25}$ = + 72.6 (*c* 1.0, $CHCl_3$); **^{1}H-NMR** (600 MHz, $CDCl_3$, ppm): *δ* 8.04 (d, *J* = 7.8 Hz, 2H), 7.67 (d, *J* = 5.4 Hz, 2H), 7.41-7.17 (m, 13H),

7.11 (d, J = 7.2 Hz, 2H), 6.00 (br, 1H), 5.10 (d, J = 11.4 Hz, 1H), 4.88 (d, J = 11.4 Hz, 1H), 2.82-2.73 (m, 2H), 2.53-2.49 (m, 2H); **^{13}C-NMR** (150 MHz, $CDCl_3$, ppm): δ 155.1, 140.2, 138.8, 135.7, 128.7, 128.6, 128.5, 128.4, 126.6, 125.9, 123.8, 120.4, 120.1, 110.4, 86.8, 75.4, 67.6, 53.0, 34.4, 20.8; **HRMS** calcd. for $C_{31}H_{26}N_2NaO_2$ $[M+Na]^+$: 481.1886, found: 481.1886; **HPLC analysis:** Daicel CHIRALPAK AD-H, *n*-hexane:*i*-PrOH = 80:20, flow rate = 0.8 mL·min^{-1}, λ = 254 nm, retention time: t_R = 14.8 min (minor), t_R = 18.0 min (major).

Benzyl (*R*)-(1-(2-bromo-9H-carbazol-9-yl)-3-phenylprop-2-yn-1-yl)carbamate (3e)

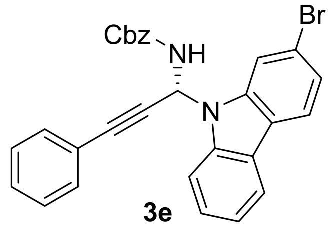

3e

Compound **3e** was obtained as white solid (15.2 mg, 60% yield, 91% ee) after flash chromatography (elution gradient: ethyl acetate:petroleum ether = 1:20); **$[\alpha]_D^{25}$** = + 93.5 (*c* 1.0, $CHCl_3$); **^{1}H-NMR** (600 MHz, $CDCl_3$, ppm): δ 8.09-8.04 (m, 2H), 7.94-7.87 (m, 2H), 7.52-7.29 (m, 13H), 6.23 (d, J = 6.0 Hz, 1H), 5.18 (d, J = 11.4 Hz, 1H), 4.99 (d, J = 10.8 Hz, 1H); **^{13}C-NMR** (150 MHz, $CDCl_3$, ppm): δ 155.1, 139.6, 139.2, 135.6, 132.0, 129.5, 128.7, 128.6, 128.5, 126.6, 123.5, 123.2, 123.0, 121.6, 121.2, 120.7, 120.5, 119.6, 113.8, 110.6, 87.0, 82.6, 67.9, 53.8; **HRMS** calcd. for $C_{29}H_{21}BrN_2NaO_2$ $[M+Na]^+$: 531.0679, found: 531.0679; **HPLC analysis:** Daicel CHIRALPAK IC, *n*-hexane:*i*-PrOH = 96:4, flow rate = 0.8 mL·min^{-1}, λ = 254 nm, retention time: t_R = 13.4 min (major), t_R = 15.0 min (minor).

Benzyl (*R*)-(1-(3-bromo-9H-carbazol-9-yl)-3-phenylprop-2-yn-1-yl)carbamate (3f)

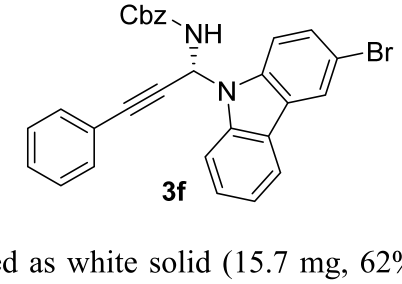

3f

Compound **3f** was obtained as white solid (15.7 mg, 62% yield, 92% ee) after flash

chromatography (elution gradient: ethyl acetate:petroleum ether = 1:20); **[α]$_D^{25}$** = + 114.6 (*c* 1.0, $CHCl_3$); **^{1}H-NMR** (400 MHz, $CDCl_3$, ppm): *δ* 8.20 (s, 1H), 8.04 (d, *J* = 7.6 Hz, 1H), 7.88-7.75 (m, 2H), 7.58-7.28 (m, 14H), 6.19 (br, 1H), 5.16 (d, *J* = 11.2 Hz, 1H), 4.97 (d, *J* = 11.2 Hz, 1H); **^{13}C-NMR** (100 MHz, $CDCl_3$, ppm): *δ* 155.1, 139.2, 137.6, 135.5, 132.0, 129.5, 128.7, 128.6, 128.5, 126.8, 125.7, 123.2, 122.8, 121.2, 120.7, 113.2, 111.9, 110.7, 86.8, 82.6, 67.9, 53.5; **HRMS** calcd. for $C_{29}H_{21}BrN_2NaO_2$ $[M+Na]^+$: 531.0679, found: 531.0679; **HPLC analysis:** Daicel CHIRALPAK AD-H, *n*-hexane:*i*-PrOH = 80:20, flow rate = 0.8 mL·min^{-1}, λ = 254 nm, retention time: t_R = 10.8 min (minor), t_R = 12.5 min (major).

Benzyl (*R*)-(1-(3-methyl-9H-carbazol-9-yl)-3-phenylprop-2-yn-1-yl)carbamate (3g)

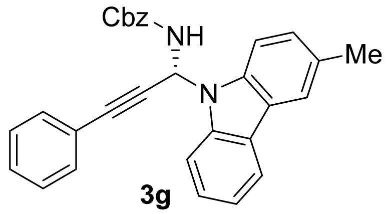

3g

Compound **3g** was obtained as white solid (14.4 mg, 65% yield, 90% ee) after flash chromatography (elution gradient: ethyl acetate:petroleum ether = 1:20); **[α]$_D^{25}$** = + 54.1 (*c* 1.0, $CHCl_3$); **^{1}H-NMR** (400 MHz, $CDCl_3$, ppm): *δ* 8.05 (d, *J* = 7.6 Hz, 1H), 7.89-7.76 (m, 3H), 7.47-7.25 (m, 14H), 6.18 (d, *J* = 6.4 Hz, 1H), 5.17 (d, *J* = 11.2 Hz, 1H), 4.95 (d, *J* = 11.2 Hz, 1H), 2.54 (s, 3H); **^{13}C-NMR** (100 MHz, $CDCl_3$, ppm): *δ* 155.2, 139.2, 137.1, 135.7, 132.0, 129.7, 129.3, 128.7, 128.5, 127.4, 125.9, 124.1, 123.8, 121.5, 120.5, 120.4, 120.1, 110.4, 110.1, 86.4, 83.2, 67.8, 53.4, 21.5; **HRMS** calcd. for $C_{30}H_{24}N_2NaO_2$ $[M+Na]^+$: 467.1730, found: 467.1730; **HPLC analysis:** Daicel CHIRALPAK AD-H, *n*-hexane:*i*-PrOH = 80:20, flow rate = 0.8 mL·min^{-1}, λ = 254 nm, retention time: t_R = 10.7 min (minor), t_R = 12.5 min (major).

Benzyl (*S*)-(3-(4-methoxyphenyl)-1-(3-phenyl-9H-carbazol-9-yl)prop-2-yn-1-yl) carbamate (3h)

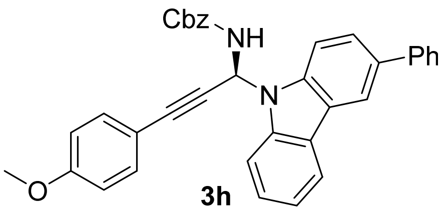

3h

In the presence of (*S*)-Li[**P2**] catalyst, compound **3h** was obtained as white solid (18.2 mg, 68% yield, 92% ee) after flash chromatography (elution gradient: ethyl acetate:petroleum ether = 1:20); **[α]$_D$^{25}** = - 108.2 (*c* 1.0, $CHCl_3$); **^{1}H-NMR** (600 MHz, $CDCl_3$, ppm): δ 8.31 (s, 1H), 8.15 (d, *J* = 6.8 Hz, 1H), 7.96-7.91 (m, 2H), 7.76-7.73 (m, 3H), 7.53-7.49 (m, 4H), 7.38-7.36 (m, 4H), 7.33-7.31 (m, 5H), 6.85-6.83 (m, 2H), 6.28 (d, *J* = 6.0 Hz, 1H), 5.19 (d, *J* = 11.4 Hz, 1H), 4.97 (d, *J* = 11.6 Hz, 1H), 3.80 (s, 3H); **^{13}C-NMR** (150 MHz, $CDCl_3$, ppm): δ 160.5, 155.2, 142.0, 139.4, 138.4, 135.7, 133.8, 133.5, 128.9, 128.7, 128.5, 128.5, 127.5, 126.7, 126.2, 125.6, 124.4, 124.0, 120.5, 120.3, 119.0, 114.2, 113.4, 110.7, 110.7, 86.7, 81.8, 67.8, 55.4, 53.6; **HRMS** calcd. for $C_{36}H_{28}N_2NaO_3$ $[M+Na]^+$: 559.1992, found: 559.1992; **HPLC analysis:** Daicel CHIRALPAK AD-H, *n*-hexane:*i*-PrOH = 80:20, flow rate = 0.8 mL·min^{-1}, λ = 254 nm, retention time: t_R = 19.0 min (major), t_R = 22.9 min (minor).

Supplementary Note 3

General procedure for the N-propargylation of indoles 4 with C-alkynyl N,O-acetals 1

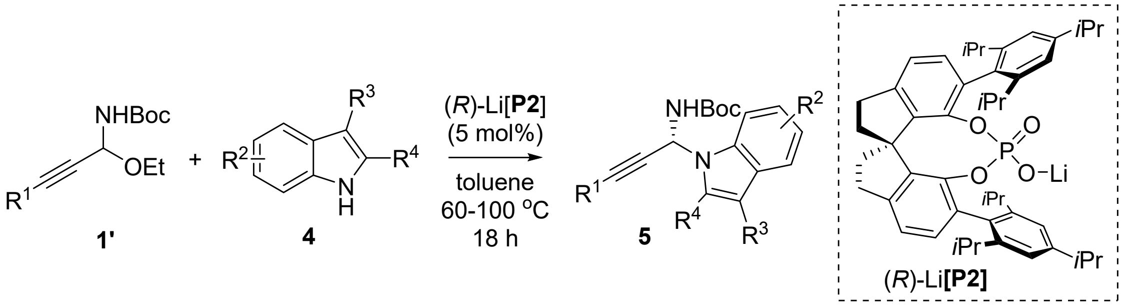

To a solution of **1** (0.05 mmol) and **4** (0.08 mmol) in toluene (1.0 mL) was added the catalyst (*R*)-Li[**P2**] (1.8 mg, 5 mol%) at the range from 60 °C to 110 °C. After stirring

for 18 h, the mixture was directly purified by silica gel chromatography (ethyl acetate:petroleum ether = 1:100 to 1:50) to afford the products **5**.

***Tert*-butyl (*R*)-(1-(2,3-dimethyl-1H-indol-1-yl)-3-phenylprop-2-yn-1-yl)carbamate (5a)**

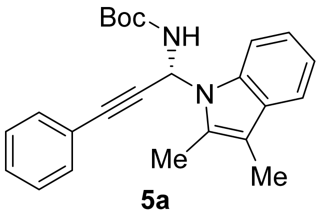

5a

Compound **5a** was obtained as colorless liquid (14.6 mg, 78% yield, 99% ee) after flash chromatography (elution gradient: ethyl acetate:petroleum ether = 1:50); **[α]$_D^{25}$** = + 36.6 (*c* 1.0, $CHCl_3$); **^{1}H-NMR** (400 MHz, $CDCl_3$, ppm): *δ* 7.75 (d, *J* = 7.6 Hz, 1H), 7.52 (d, *J* = 7.6 Hz, 1H), 7.44-7.42 (m, 2H), 7.37-7.29 (m, 3H), 7.23-7.13 (m, 2H), 7.12-7.07 (m, 1H), 5.87 (d, *J* = 5.2 Hz, 1H), 2.58 (s, 3H), 2.25 (s, 3H), 1.43 (s, 9H); **^{13}C-NMR** (100 MHz, $CDCl_3$, ppm): *δ* 154.0, 134.6, 131.9, 129.9, 129.1, 128.5, 121.8, 121.2, 119.7, 118.4, 110.4, 108.2, 85.5, 84.3, 81.0, 52.9, 28.4, 10.6, 8.9; **HRMS** calcd. for $C_{24}H_{26}N_2NaO_2$ [M+Na]$^+$: 397.1886, found: 397.1886; **HPLC analysis:** Daicel CHIRALCEL OD-H, *n*-hexane:*i*-PrOH = 95:5, flow rate = 0.8 mL·min^{-1}, λ = 254 nm, retention time: t_R = 8.7 min (major), t_R = 11.3 min (minor).

***Tert*-butyl (*R*)-(1-(2,3-dimethyl-1H-indol-1-yl)-3-(*o*-tolyl)prop-2-yn-1-yl)carbamate (5b)**

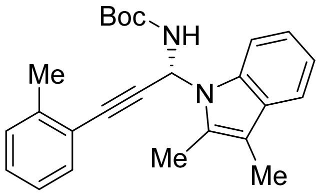

5b

Compound **5b** was obtained as colorless liquid (14.2 mg, 73% yield, 99% ee) after flash chromatography (elution gradient: ethyl acetate:petroleum ether = 1:50); **[α]$_D^{25}$** = + 39.5 (*c* 1.0, $CHCl_3$); **^{1}H-NMR** (400 MHz, $CDCl_3$, ppm): *δ* 7.74 (d, *J* = 7.6 Hz, 1H), 7.50 (d, *J* = 7.6 Hz, 1H), 7.38 (d, *J* = 7.6 Hz, 1H), 7.25-7.10 (m, 6H), 5.84 (br, 1H), 2.51 (s, 3H),

2.37 (s, 3H), 2.24 (s, 3H), 1.42 (s, 9H); **^{13}C-NMR** (100 MHz, $CDCl_3$, ppm): *δ* 154.0, 140.6, 134.6, 132.4, 131.9, 129.9, 129.7, 129.1, 125.7, 121.6, 121.1, 119.7, 118.4, 110.5, 108.2, 88.0, 84.5, 81.1, 53.1, 28.4, 20.9, 10.7, 8.9; **HRMS** calcd. for $C_{25}H_{28}N_2NaO_2$ $[M+Na]^+$: 411.2043, found: 411.2043; **HPLC analysis:** Daicel CHIRALCEL OD-H, *n*-hexane:*i*-PrOH = 95:5, flow rate = 0.8 mL·min^{-1}, λ = 254 nm, retention time: t_R = 7.9 min (major), t_R = 9.7 min (minor).

***Tert*-butyl (*R*)-(1-(2,3-dimethyl-1H-indol-1-yl)-3-(m-tolyl)prop-2-yn-1-yl) carbamate (5c)**

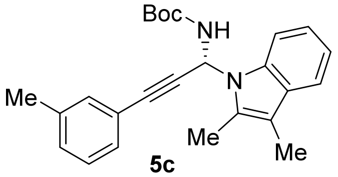

5c

Compound **5c** was obtained as colorless liquid (14.0 mg, 72% yield, 99% ee) after flash chromatography (elution gradient: ethyl acetate:petroleum ether = 1:50); **$[\alpha]_D^{25}$** = + 20.1 (*c* 1.0, $CHCl_3$); **^{1}H-NMR** (400 MHz, $CDCl_3$, ppm): *δ* 7.73 (d, *J* = 7.2 Hz, 1H), 7.50 (d, *J* = 7.6 Hz, 1H), 7.24-7.12 (m, 6H), 7.06-7.04 (m, 1H), 5.84 (br, 1H), 2.57 (s, 3H), 2.31 (s, 3H), 2.24 (s, 3H), 1.42 (s, 9H); **^{13}C-NMR** (100 MHz, $CDCl_3$, ppm): *δ* 154.0, 138.2, 134.6, 132.5, 131.8, 130.0, 129.9, 129.0, 128.4, 121.6, 121.1, 119.6, 118.4, 110.4, 108.2, 85.7, 84.0, 81.0, 53.0, 28.4, 21.3, 10.7, 8.9; **HRMS** calcd. for $C_{25}H_{28}N_2NaO_2$ $[M+Na]^+$: 411.2043, found: 411.2043; **HPLC analysis:** Daicel CHIRALPAK IC, *n*-hexane:*i*-PrOH = 97:3, flow rate = 0.8 mL·min^{-1}, λ = 254 nm, retention time: t_R = 7.3 min (minor), t_R = 9.5 min (major).

***Tert*-butyl (*R*)-(1-(2,3-dimethyl-1H-indol-1-yl)-3-(*p*-tolyl)prop-2-yn-1-yl) carbamate (5d)**

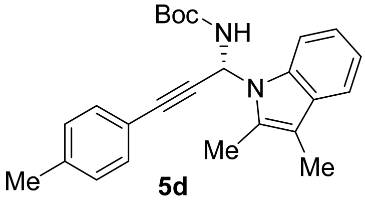

5d

Compound **5d** was obtained as colorless liquid (14.0 mg, 72% yield, 98% ee) after flash

chromatography (elution gradient: ethyl acetate:petroleum ether = 1:50); **[α]$_D$^{25}** = + 70.3 (*c* 1.0, $CHCl_3$); **^{1}H-NMR** (400 MHz, $CDCl_3$, ppm): δ 7.74 (d, J = 7.6 Hz, 1H), 7.50 (d, J = 7.6 Hz, 1H), 7.32-7.30 (m, 2H), 7.21-7.04 (m, 5H), 5.84 (br, 1H), 2.57 (s, 3H), 2.34 (s, 3H), 2.24 (s, 3H), 1.42 (s, 9H); **^{13}C-NMR** (100 MHz, $CDCl_3$, ppm): δ 154.0, 139.3, 134.6, 131.8, 129.9, 129.2, 121.1, 119.6, 118.7, 118.4, 110.4, 108.2, 85.7, 83.7, 81.0, 53.0, 28.4, 21.6, 10.7, 8.9; **HRMS** calcd. for $C_{25}H_{28}N_2NaO_2$ $[M+Na]^+$: 411.2043, found: 411.2045; **HPLC analysis:** Daicel CHIRALPAK AD-H, *n*-hexane:*i*-PrOH = 96:4, flow rate = 0.8 mL·min^{-1}, λ = 254 nm, retention time: t_R = 7.8 min (minor), t_R = 8.9 min (major).

***Tert*-butyl (*R*)-(1-(2,3-dimethyl-1H-indol-1-yl)-3-(4-methoxyphenyl)prop-2-yn-1-yl) carbamate (5e)**

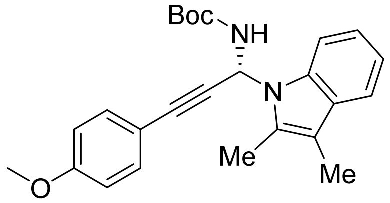

5e

Compound **5e** was obtained as colorless liquid (15.8 mg, 78% yield, 99% ee) after flash chromatography (elution gradient: ethyl acetate:petroleum ether = 1:50); **[α]$_D$^{25}** = + 35.0 (*c* 1.0, $CHCl_3$); **^{1}H-NMR** (400 MHz, $CDCl_3$, ppm): δ 7.75 (d, J = 7.2 Hz, 1H), 7.51 (d, J = 7.2 Hz, 1H), 7.37-7.35 (m, 2H), 7.22-7.13 (m, 2H), 7.06-7.04 (m, 1H), 6.84-6.82 (m, 2H), 5.87 (d, J = 4.8 Hz, 1H), 3.80 (s, 3H), 2.58 (s, 3H), 2.25 (s, 3H), 1.43 (s, 9H); **^{13}C-NMR** (100 MHz, $CDCl_3$, ppm): δ 160.2, 154.0, 134.6, 133.4, 131.9, 129.9, 121.1, 119.6, 118.4, 114.1, 113.8, 110.5, 108.1, 85.5, 83.0, 80.9, 55.4, 53.0, 28.4, 10.6, 8.9; **HRMS** calcd. for $C_{25}H_{28}N_2NaO_3$ $[M+Na]^+$: 427.1992, found: 427.1992; **HPLC analysis:** Daicel CHIRALCEL OD-H, *n*-hexane:*i*-PrOH = 95:5, flow rate = 0.8 mL·min^{-1}, λ = 254 nm, retention time: t_R = 9.7 min (minor), t_R = 10.8 min (major).

***Tert*-butyl (*R*)-(3-(4-chlorophenyl)-1-(2,3-dimethyl-1H-indol-1-yl)prop-2-yn-1-yl) carbamate (5f)**

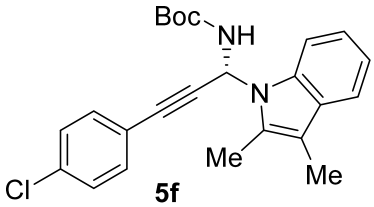

5f

Compound **5f** was obtained as colorless liquid (14.5 mg, 71% yield, 97% ee) after flash chromatography (elution gradient: ethyl acetate:petroleum ether = 1:50); **$[\alpha]_D^{25}$** = + 43.4 (*c* 1.0, $CHCl_3$); **^{1}H-NMR** (400 MHz, $CDCl_3$, ppm): δ 7.69 (d, J = 7.2 Hz, 1H), 7.50 (d, J = 7.6 Hz, 1H), 7.35-7.26 (m, 4H), 7.21-7.12 (m, 2H), 7.05-7.03 (m, 1H), 5.83 (d, J = 6.8 Hz, 1H), 2.55 (s, 3H), 2.24 (s, 3H), 1.41 (s, 9H); **^{13}C-NMR** (100 MHz, $CDCl_3$, ppm): δ 154.0, 135.3, 134.5, 133.2, 131.8, 129.9, 128.9, 120.3, 119.7, 118.4, 110.3, 108.3, 85.3, 84.4, 81.2, 52.9, 28.4, 10.6, 8.9; **HRMS** calcd. for $C_{24}H_{25}ClN_2NaO_2$ $[M+Na]^+$: 431.1497, found: 431.1497; **HPLC analysis:** Daicel CHIRALCEL OD-H, *n*-hexane:*i*-PrOH = 98:2, flow rate = 0.8 mL·min^{-1}, λ = 254 nm, retention time: t_R = 12.5 min (minor), t_R = 15.3min (major).

***Tert*-butyl (*R*)-(1-(2,3-dimethyl-1H-indol-1-yl)-3-(4-fluorophenyl)prop-2-yn-1-yl) carbamate (5g)**

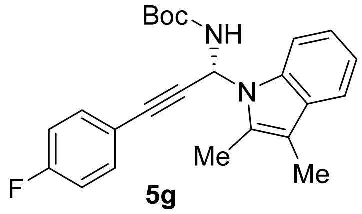

5g

Compound **5g** was obtained as colorless liquid (14.1 mg, 72% yield, 98% ee) after flash chromatography (elution gradient: ethyl acetate:petroleum ether = 1:50); **$[\alpha]_D^{25}$** = + 50.6 (*c* 1.0, $CHCl_3$); **^{1}H-NMR** (400 MHz, $CDCl_3$, ppm): δ 7.71 (d, J = 7.6 Hz, 1H), 7.50 (d, J = 7.6 Hz, 1H), 7.41-7.38 (m, 2H), 7.22-7.12 (m, 2H), 7.09-6.98 (m, 3H), 5.84 (d, J = 6.0 Hz, 1H), 2.56 (s, 3H), 2.24 (s, 3H), 1.42 (s, 9H); **^{13}C-NMR** (100 MHz, $CDCl_3$, ppm): δ 164.2, 161.8, 154.0, 134.5, 134.0, 133.9, 131.8, 129.9, 129.2, 128.4, 121.2, 119.7,

118.5, 118.0, 117.9, 116.0, 115.7, 110.3, 108.2, 84.4, 84.1, 81.1, 52.8, 28.4, 10.6, 8.9; **HRMS** calcd. for $C_{24}H_{25}FN_2NaO_2$ [M+Na]$^+$: 415.1792, found: 415.1792; **HPLC analysis:** Daicel CHIRALCEL OD-H, *n*-hexane:*i*-PrOH = 95:5, flow rate = 0.8 mL·min^{-1}, λ = 254 nm, retention time: t_R = 7.7 min (minor), t_R = 8.9 min (major).

***Tert*-butyl (*R*)-(1-(2,3-dimethyl-1H-indol-1-yl)-3-(4-(trifluoromethyl)phenyl)prop-2-yn-1-yl) carbamate (5h)**

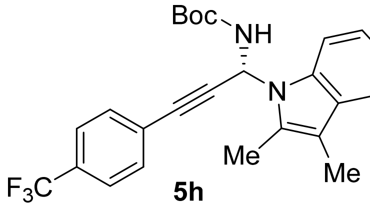

5h

Compound **5h** was obtained as colorless liquid (16.4 mg, 74% yield, 97% ee) after flash chromatography (elution gradient: ethyl acetate:petroleum ether = 1:50); **[α]$_D$^{25}** = + 46.4 (*c* 1.0, $CHCl_3$); **^{1}H-NMR** (400 MHz, $CDCl_3$, ppm): *δ* 7.69 (d, *J* = 7.6 Hz, 1H), 7.67-7.50 (m, 5H), 7.25-7.07 (m, 3H), 5.85 (d, *J* = 7.2 Hz, 1H), 2.55 (s, 3H), 2.24 (s, 3H), 1.42 (s, 9H); **^{13}C-NMR** (100 MHz, $CDCl_3$, ppm): *δ* 154.0, 134.5, 132.2, 131.8, 131.0, 130.7, 130.0, 125.6, 125.5, 125.4, 125.4, 125.2, 122.5, 121.3, 119.9, 118.6, 110.2, 108.5, 86.7, 84.0, 81.3, 52.8, 28.4, 10.6, 8.9; **HRMS** calcd. for $C_{25}H_{25}F_3N_2NaO_2$ [M+Na]$^+$: 465.1760, found: 465.1761; **HPLC analysis:** Daicel CHIRALCEL OD-H, *n*-hexane:*i*-PrOH = 98:2, flow rate = 0.8 mL·min^{-1}, λ = 254 nm, retention time: t_R = 10.6 min (minor), t_R = 12.9 min (major).

***Tert*-butyl (*S*)-(1-(2,3-dimethyl-1H-indol-1-yl)-3-(thiophen-2-yl)prop-2-yn-1-yl)carbamate (5i)**

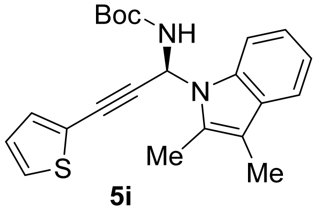

5i

In the presence of (*S*)-Li[**P2**] catalyst, compound **5i** was obtained as colorless liquid

(13.7 mg, 72% yield, 98% ee) after flash chromatography (elution gradient: ethyl acetate:petroleum ether = 1:50); **[α]$_D^{25}$** = - 43.9 (*c* 1.0, $CHCl_3$); **^{1}H-NMR** (400 MHz, $CDCl_3$, ppm): *δ* 7.69 (d, *J* = 7.6 Hz, 1H), 7.50 (d, *J* = 7.6 Hz, 1H), 7.27 (d, *J* = 5.2 Hz, 1H), 7.22-7.06 (m, 4H), 6.97-6.95 (m, 1H), 5.84 (d, *J* = 6.4 Hz, 1H), 2.55 (s, 3H), 2.24 (s, 3H), 1.42 (s, 9H); **^{13}C-NMR** (100 MHz, $CDCl_3$, ppm): *δ* 154.0, 134.5, 133.1, 131.8, 129.9, 128.1, 127.2, 121.5, 121.2, 119.7, 118.4, 110.3, 108.3, 88.0, 81.0, 79.0, 53.0, 28.4, 10.6, 8.9; **HRMS** calcd. for $C_{22}H_{24}N_2NaO_2S$ $[M+Na]^+$: 403.1451, found: 403.1451; **HPLC analysis:** Daicel CHIRALCEL OD-H, *n*-hexane:*i*-PrOH = 92:8, flow rate = 0.8 mL·min^{-1}, λ = 254 nm, retention time: t_R = 7.9 min (minor), t_R = 10.3 min (major).

***Tert*-butyl (*R*)-(1-(2,3-dimethyl-1H-indol-1-yl)hept-2-yn-1-yl)carbamate (5j)**

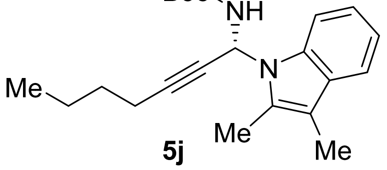

5j

Compound **5j** was obtained as white solid (13.5 mg, 67% yield, 96% ee) after flash chromatography (elution gradient: ethyl acetate:petroleum ether = 1:50); **[α]$_D^{25}$** = + 10.4 (*c* 1.0, $CHCl_3$); **^{1}H-NMR** (400 MHz, $CDCl_3$, ppm): *δ* 7.67 (d, *J* = 7.6 Hz, 1H), 7.49 (d, *J* = 7.6 Hz, 1H), 7.19-7.11 (m, 2H), 6.80 (d, *J* = 7.2 Hz, 1H), 5.74 (d, *J* = 6.8 Hz, 1H), 2.52 (s, 3H), 2.23-2.19 (m, 5H), 1.51-1.45 (m, 3H), 1.42-1.35 (m, 10H), 0.90 (t, *J* = 7.2 Hz, 3H); **^{13}C-NMR** (100 MHz, $CDCl_3$, ppm): *δ* 154.0, 134.4, 131.9, 129.8, 120.8, 119.5, 118.3, 110.5, 107.8, 86.6, 80.8, 75.5, 52.6, 30.4, 28.4, 22.1, 18.4, 13.7, 10.6, 8.9; **HRMS** calcd. for $C_{22}H_{30}N_2NaO_2$ $[M+Na]^+$: 377.2199, found: 377.2199; **HPLC analysis:** Daicel CHIRALPAK IC, *n*-hexane:*i*-PrOH = 95:5, flow rate = 0.8 mL·min^{-1}, λ = 230 nm, retention time: t_R = 5.7 min (minor), t_R = 7.0 min (major).

***Tert*-butyl (*R*)-(1-(2,3-dimethyl-1H-indol-1-yl)-3-(trimethylsilyl)prop-2-yn-1-yl)carbamate (5k)**

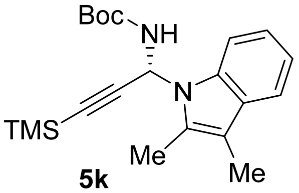

5k

Compound **5k** was obtained as colorless liquid (12.0 mg, 65% yield, 97% ee) after flash chromatography (elution gradient: ethyl acetate:petroleum ether = 1:50); **[α]$_D$^{25}** = + 23.8 (*c* 1.0, $CHCl_3$); **^{1}H-NMR** (400 MHz, $CDCl_3$, ppm): δ 7.66 (d, J = 7.6 Hz, 1H), 7.49 (d, J = 7.6 Hz, 1H), 7.19-7.10 (m, 2H), 6.80 (d, J = 7.6 Hz, 1H), 5.73 (d, J = 6.0 Hz, 1H), 2.51 (s, 3H), 2.22 (s, 3H), 1. 40 (s, 9H), 0.17 (s, 9H); **^{13}C-NMR** (100 MHz, $CDCl_3$, ppm): δ 153.9, 134.5, 131.9, 129.8, 120.9, 119.6, 118.3, 110.5, 108.1, 99.9, 91.0, 81.0, 52.7, 28.4, 10.6, 8.9, 0.3; **HRMS** calcd. for $C_{21}H_{30}N_2NaO_2Si$ [M+Na]$^+$: 393.1969, found: 393.1969; **HPLC analysis:** Daicel CHIRALCEL IB-3, *n*-hexane:*i*-PrOH = 95:5, flow rate = 0.8 mL·min^{-1}, λ = 254 nm, retention time: t_R = 8.7 min (minor), t_R = 9.7 min (major).

***Tert*-butyl (*R*)-(1-(5-fluoro-2,3-dimethyl-1H-indol-1-yl)-3-phenylprop-2-yn-1-yl)carbamate (5l)**

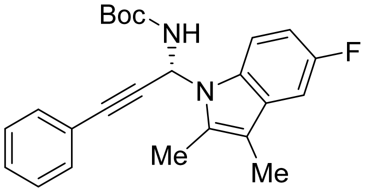

5l

Compound **5l** was obtained as colorless liquid (13.5 mg, 69% yield, 95% ee) after flash chromatography (elution gradient: ethyl acetate:petroleum ether = 1:50); **[α]$_D$^{25}** = + 44.6 (*c* 1.0, $CHCl_3$); **^{1}H-NMR** (400 MHz, $CDCl_3$, ppm): δ 7.62 (br, 1H), 7.42-7.40 (m, 2H), 7.35-7.29 (m, 3H), 7.13 (dd, J = 2.0, 9.6 Hz, 1H), 7.01 (d, J = 7.2 Hz, 1H), 6.92 (dq, J = 2.0, 9.2 Hz, 1H), 5.80 (br, 1H), 2.56 (s, 3H), 2.20 (s, 3H), 1. 43 (s, 9H); **^{13}C-NMR** (100 MHz, $CDCl_3$, ppm): δ 159.4, 157.0, 154.0, 133.8, 131.9, 131.1, 130.4, 129.2, 128.5, 121.6, 110.9, 110.8, 109.1, 108.9, 103.7, 103.5, 85.7, 84.0, 81.2, 53.1, 28.4, 10.8, 8.9;

HRMS calcd. for $C_{24}H_{25}FN_2NaO_2$ $[M+Na]^+$: 415.1792, found: 415.1792; **HPLC analysis:** Daicel CHIRALPAK IC, *n*-hexane:*i*-PrOH = 95:5, flow rate = 0.8 mL·min^{-1}, λ = 254 nm, retention time: t_R = 7.4 min (minor), t_R = 8.6 min (major).

***Tert*-butyl (*S*)-(3-phenyl-1-(2,3,5-trimethyl-1H-indol-1-yl)prop-2-yn-1-yl) carbamate (5m)**

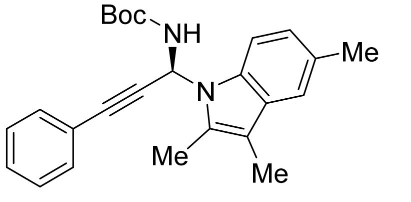

5m

In the presence of (*S*)-Li[**P2**] catalyst, compound **5m** was obtained as colorless liquid (14.4 mg, 74% yield, 90% ee) after flash chromatography (elution gradient: ethyl acetate:petroleum ether = 1:50); $[\alpha]_D^{25}$ = - 41.4 (*c* 1.0, $CHCl_3$); **^{1}H-NMR** (600 MHz, $CDCl_3$, ppm): δ 7.63-7.62 (m, 1H), 7.43-7.41 (m, 2H), 7.35-7.29 (m, 4H), 7.02 (d, *J* = 7.8 Hz, 2H), 5.84 (br, 1H), 2.55 (s, 3H), 2.47 (s, 3H), 2.22 (s, 3H), 1.42 (s, 9H); **^{13}C-NMR** (150 MHz, $CDCl_3$, ppm): δ 154.0, 132.9, 131.9, 130.2, 129.1, 128.9, 128.6, 128.5, 122.6, 121.9, 118.3, 110.1, 107.8, 85.4, 84.5, 81.0, 53.0, 28.4, 21.5, 10.6, 8.9; **HRMS** calcd. for $C_{25}H_{28}N_2NaO_2$ $[M+Na]^+$: 411.2043, found: 411.2043; **HPLC analysis:** Daicel CHIRALPAK IC, *n*-hexane:*i*-PrOH = 96:4, flow rate = 0.8 mL·min^{-1}, λ = 254 nm, retention time: t_R = 6.3 min (major), t_R = 7.2 min (minor).

***Tert*-butyl (*R*)-(3-phenyl-1-(2,3,4,6-tetramethyl-1H-indol-1-yl)prop-2-yn-1-yl)carbamate (5n)**

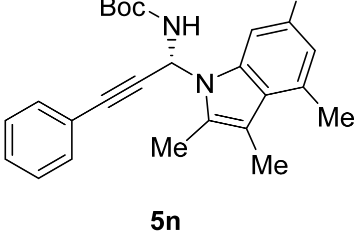

5n

Compound **5n** was obtained as colorless liquid (15.3 mg, 76% yeild, 95% ee) after flash

chromatography (elution gradient: ethyl acetate:petroleum ether = 1:50); **[α]$_D^{25}$** = + 11.7 (*c* 1.0, $CHCl_3$); **^{1}H-NMR** (400 MHz, $CDCl_3$, ppm): δ 7.42-7.35 (m, 3H), 7.33-7.27 (m, 3H), 7.03 (d, *J* = 7.6 Hz, 1H), 6.68 (s, 1H), 5.85 (d, *J* = 6.8 Hz, 1H), 2.66 (s, 3H), 2.51 (s, 3H), 2.44 (s, 3H), 2.40 (s, 3H), 1.42 (s, 9H); **^{13}C-NMR** (100 MHz, $CDCl_3$, ppm): δ 155.5, 138.7, 135.2, 131.9, 130.7, 130.4, 129.0, 128.5, 123.3, 122.0, 108.5, 85.3, 84.6, 81.0, 52.9, 28.4, 21.9, 20.5, 12.0, 10.4; **HRMS** calcd. for $C_{26}H_{30}N_2NaO_2$ $[M+Na]^+$: 425.2199, found: 425.2199; **HPLC analysis:** Daicel CHIRALCEL OD-H, *n*-hexane:*i*-PrOH = 97:3, flow rate = 0.6 mL·min^{-1}, λ = 254 nm, retention time: t_R = 9.9 min (major), t_R = 14.5 min (minor).

***Tert*-butyl (*R*)-(3-phenyl-1-(1,2,3,4-tetrahydro-9H-carbazol-9-yl)prop-2-yn-1-yl)carbamate (5o)**

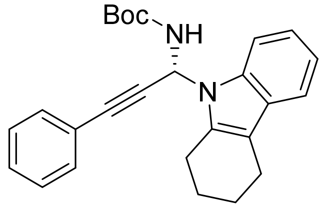

5o

Compound **5o** was obtained as colorless liquid (16.0 mg, 80% yield, 95% ee) after flash chromatography (elution gradient: ethyl acetate:petroleum ether = 1:50); **[α]$_D^{25}$** = + 23.1 (*c* 1.0, $CHCl_3$); **^{1}H-NMR** (400 MHz, $CDCl_3$, ppm): δ 7.75 (d, *J* = 7.2 Hz, 1H), 7.50 (d, *J* = 7.6 Hz, 1H), 7.44-7.42 (m, 2H), 7.35-7.29 (m, 3H), 7.23-7.12 (m, 2H), 7.02 (d, *J* = 8.4 Hz, 1H), 5.85 (d, *J* = 5.2 Hz, 1H), 3.16-3.12 (m, 1H), 2.88-2.81 (m, 1H), 2.73 (t, *J* = 6.0 Hz, 2H), 2.00-1.86 (m, 4H), 1.44 (s, 9H); **^{13}C-NMR** (100 MHz, $CDCl_3$, ppm): δ 154.0, 134.9, 134.8, 131.9, 129.1, 128.7, 128.5, 121.8, 121.2, 119.7, 118.2, 111.1, 110.5, 85.4, 84.3, 81.0, 52.5, 28.4, 23.4, 23.1, 22.5, 21.1; **HRMS** calcd. for $C_{26}H_{28}N_2NaO_2$ $[M+Na]^+$: 423.2043, found: 423.2043; **HPLC analysis:** Daicel CHIRALPAK OD-H, *n*-hexane:*i*-PrOH = 97:3, flow rate = 0.8 mL·min^{-1}, λ = 254 nm, retention time: t_R = 13.8 min (minor), t_R = 19.1 min (major).

***Tert*-butyl (*R*)-(1-(8-fluoro-1,2,3,4-tetrahydro-9H-carbazol-9-yl)-3-phenylprop-2-yn-1-yl)carbamate (5p)**

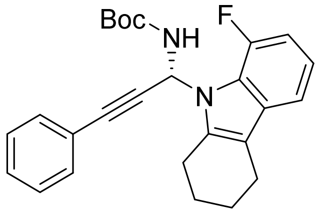

5p

Compound **5p** was obtained as colorless liquid (15.0 mg, 72% yield, 99% ee) after flash chromatography (elution gradient: ethyl acetate:petroleum ether = 1:50); $[\alpha]_D^{25}$ = + 34.4 (*c* 1.0, $CHCl_3$); **^{1}H-NMR** (400 MHz, $CDCl_3$, ppm): δ 7.37-7.36 (m, 2H), 7.29-7.22 (m, 4H), 7.04-6.89 (m, 3H), 6.16 (br, 1H), 3.15-3.11 (m, 1H), 2.88-2.81 (m, 1H), 2.68 (br, 2H), 1.99-1.85 (m, 4H), 1.43 (s, 9H); **^{13}C-NMR** (100 MHz, $CDCl_3$, ppm): δ 154.2, 150.4, 148.0, 136.3, 133.4, 133.0, 132.0, 131.4, 128.9, 128.4, 122.0, 120.2, 120.2, 114.0, 114.0, 111.1, 107.6, 107.4, 84.7, 84.3, 81.0, 52.7, 28.4, 23.3, 23.0, 22.5, 21.3; **HRMS** calcd. for $C_{26}H_{27}FN_2NaO_2$ $[M+Na]^+$: 441.1949, found: 441.1949; **HPLC analysis:** Daicel CHIRALCEL IB-3, *n*-hexane:*i*-PrOH = 98:2, flow rate = 0.7 mL·min^{-1}, λ = 254 nm, retention time: t_R = 20.0 min (major), t_R = 21.5 min (minor).

***Tert*-butyl (*R*)-(1-(6-bromo-1,2,3,4-tetrahydro-9H-carbazol-9-yl)-3-phenylprop-2-yn-1-yl)carbamate (5q)**

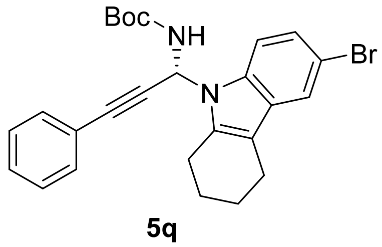

5q

Compound **5q** was obtained as colorless liquid (17.4 mg, 73% yield, 99% ee) after flash chromatography (elution gradient: ethyl acetate:petroleum ether = 1:50); $[\alpha]_D^{25}$ = + 55.7 (*c* 1.0, $CHCl_3$); **^{1}H-NMR** (400 MHz, $CDCl_3$, ppm): δ 7.59-7.58 (m, 2H), 7.41-7.39 (m, 2H), 7.37-7.29 (m, 3H), 7.27-7.25 (m, 1H), 6.95 (d, *J* = 8.0 Hz, 1H), 5.78 (d, *J* = 6.0 Hz, 1H), 3.15-3.11 (m, 1H), 2.85-2.78 (m, 1H), 2.65 (t, *J* = 6.0 Hz, 2H), 1.96-1.85 (m,

4H), 1.42 (s, 9H); **^{13}C-NMR** (100 MHz, $CDCl_3$, ppm): δ 153.9, 136.4, 133.5, 131.9, 130.5, 129.3, 128.5, 123.8, 121.5, 120.8, 113.1, 111.9, 110.8, 85.8, 83.8, 81.3, 52.7, 28.4, 23.2, 22.9, 22.5, 21.0; **HRMS** calcd. for $C_{26}H_{27}BrN_2NaO_2$ $[M+Na]^+$: 501.1148, found: 501.1148; **HPLC analysis:** Daicel CHIRALCEL OD-H, *n*-hexane:*i*-PrOH = 95:5, flow rate = 0.8 mL·min^{-1}, λ = 254 nm, retention time: t_R = 9.0 min (major).

***Tert*-butyl (*R*)-(1-(6-methyl-1,2,3,4-tetrahydro-9H-carbazol-9-yl)-3-phenylprop-2-yn-1-yl)carbamate (5r)**

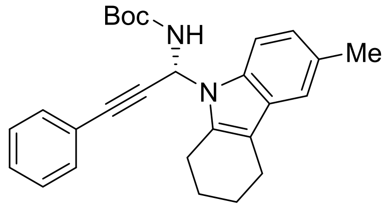

5r

Compound **5r** was obtained as colorless liquid (14.9 mg, 72% yield, 98% ee) after flash chromatography (elution gradient: ethyl acetate:petroleum ether = 1:50); **$[\alpha]_D^{25}$** = + 51.8 (*c* 1.0, $CHCl_3$); **^{1}H-NMR** (400 MHz, $CDCl_3$, ppm): δ 7.60 (d, *J* = 7.2 Hz, 1H), 7.42-7.40 (m, 2H), 7.32-7.24 (m, 4H), 7.00 (d, *J* = 8.4 Hz, 1H), 6.96-6.94 (m, 1H), 5.79 (br, 1H), 3.11-3.01 (m, 1H), 2.83-2.77 (m, 1H), 2.67 (t, *J* = 5.6 Hz, 2H), 2.44 (s, 3H),1.95-1.84 (m, 4H), 1.41 (s, 9H); **^{13}C-NMR** (100 MHz, $CDCl_3$, ppm): δ 154.0, 135.0, 133.1, 131.9, 131.8, 129.0, 129.0, 128.5, 122.6, 121.9, 118.0, 110.6, 110.2, 85.3, 84.5, 81.0, 52.6, 28.4, 23.4, 23.1, 22.5, 21.5, 21.2; **HRMS** calcd. for $C_{27}H_{30}N_2NaO_2$ $[M+Na]^+$: 437.2199, found: 437.2199; **HPLC analysis:** Daicel CHIRALCEL OD-H, *n*-hexane:*i*-PrOH = 95:5, flow rate = 0.8 mL·min^{-1}, λ = 254 nm, retention time: t_R = 6.8 min (major), t_R = 8.1 min (minor).

***Tert*-butyl (*R*)-(1-(5,7-dichloro-1,2,3,4-tetrahydro-9H-carbazol-9-yl)-3-phenylprop-2-yn-1-yl)carbamate (5s)**

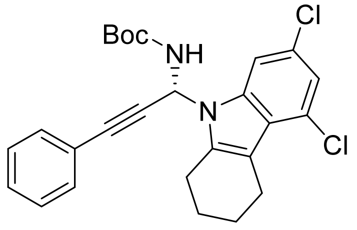

5s

Compound **5s** was obtained as colorless liquid (15.0 mg, 62% yield, 97% ee) after flash chromatography (elution gradient: ethyl acetate:petroleum ether = 1:50); **[α]$_D$^{25}** = + 81.0 (*c* 1.0, $CHCl_3$); **^{1}H-NMR** (400 MHz, $CDCl_3$, ppm): *δ* 7.62 (br, 1H), 7.42-7.40 (m, 2H), 7.38-7.30 (m, 4H), 7.06 (d, *J* = 1.6 Hz, 1H), 6.89 (d, *J* = 8.0 Hz, 1H), 5.79 (br, 1H), 3.14-3.10 (m, 1H), 3.04-3.02 (m, 2H), 2.81-2.75 (m, 1H), 1.95-1.89 (m, 2H), 1.86-1.81 (m, 2H), 1.43 (s, 9H); **^{13}C-NMR** (100 MHz, $CDCl_3$, ppm): *δ* 153.9, 136.8, 135.9, 131.9, 129.4, 129.2, 128.6, 128.4, 126.5, 121.3, 120.7, 111.4, 109.4, 86.3, 83.3, 81.5, 52.8, 28.4, 23.1, 23.0, 22.7, 22.6; **HRMS** calcd. for $C_{26}H_{26}Cl_2N_2NaO_2$ $[M+Na]^+$: 491.1264, found: 491.1264; **HPLC analysis:** Daicel CHIRALCEL OD-H, *n*-hexane:*i*-PrOH = 95:5, flow rate = 0.8 mL·min^{-1}, λ = 254 nm, retention time: t_R = 7.5 min (major), t_R = 12.3 min (minor).

***Tert*-butyl (*R*)-(5-phenyl-1-(1,2,3,4-tetrahydro-9H-carbazol-9-yl)pent-2-yn-1-yl)carbamate (5t)**

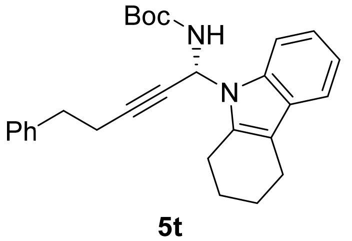

5t

Compound **5t** was obtained as colorless liquid (14.8 mg, 69% yield, 97% ee) after flash chromatography (elution gradient: ethyl acetate:petroleum ether = 1:50); **[α]$_D$^{25}** = + 18.5 (*c* 1.0, $CHCl_3$); **^{1}H-NMR** (400 MHz, $CDCl_3$, ppm): *δ* 7.50-7.44 (m, 2H), 7.29-7.20 (m, 3H), 7.16-7.07 (m, 4H), 6.71 (d, *J* = 10.8 Hz, 1H), 5.61 (d, *J* = 4.8 Hz, 1H), 3.03-2.99

(m, 1H), 2.84-2.78 (m, 2H), 2.74-2.67 (m, 3H), 2.51-2.47 (m, 2H), 1.93-1.89 (m, 2H), 1.86-1.81 (m, 2H),1.3 (s, 9H); **^{13}C-NMR** (100 MHz, $CDCl_3$, ppm): δ 153.9, 140.3, 134.9, 134.6, 128.6, 128.5, 126.6, 121.0, 119.5, 118.0, 110.7, 110.6, 85.6, 80.8, 76.5, 52.1, 34.6, 28.4, 23.4, 23.0, 22.4, 21.1, 20.9; **HRMS** calcd. for $C_{28}H_{32}N_2NaO_2$ $[M+Na]^+$: 451.2356, found: 451.2356; **HPLC analysis:** Daicel CHIRALPAK IC, *n*-hexane:*i*-PrOH = 95:5, flow rate = 0.8 mL·min^{-1}, λ = 230 nm, retention time: t_R = 7.6 min (minor), t_R = 10.2 min (major).

***Tert*-butyl (*R*)-(1-(1,2,3,4-tetrahydro-9H-carbazol-9-yl)hept-2-yn-1-yl)carbamate (5u)**

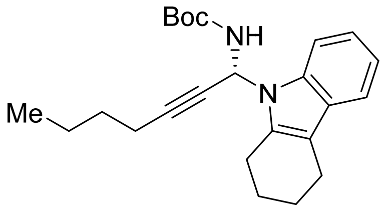

5u

Compound **5u** was obtained as white solid (12.7 mg, 67% yield, 95% ee) after flash chromatography (elution gradient: ethyl acetate:petroleum ether = 1:50); **$[\alpha]_D^{25}$** = + 14.0 (*c* 1.0, $CHCl_3$); **^{1}H-NMR** (400 MHz, $CDCl_3$, ppm): δ 7.65 (d, *J* = 7.2 Hz, 1H), 7.46 (d, *J* = 7.6 Hz, 1H), 7.18-7.09 (m, 2H), 6.74 (d, *J* = 6.0 Hz, 1H), 5.67 (br, 1H), 3.10-3.06 (m, 1H), 2.80-2.69 (m, 3H), 2.21 (dq, *J* = 2.0, 6.8 Hz, 2H), 1.96-1.84 (m, 4H), 1.52-1.45 (m, 2H), 1.40-1.35 (m, 11H), 0.89 (t, *J* = 7.2 Hz, 3H); **^{13}C-NMR** (100 MHz, $CDCl_3$, ppm): δ 154.0, 135.0, 134.7, 128.6, 120.9, 119.5, 118.0, 110.7, 86.6, 80.8, 75.6, 52.3, 30.4, 28.4, 23.4, 23.1, 22.5, 22.1, 21.2, 18.5, 13.7; **HRMS** calcd. for $C_{24}H_{32}N_2NaO_2$ $[M+Na]^+$: 403.2356, found: 403.2356; **HPLC analysis:** Daicel CHIRALPAK IC, *n*-hexane:*i*-PrOH = 95:5, flow rate = 0.8 mL·min^{-1}, λ = 230 nm, retention time: t_R = 5.9 min (minor), t_R = 6.9min (major).

***Tert*-butyl (*R*)-(3-cyclopropyl-1-(1,2,3,4-tetrahydro-9H-carbazol-9-yl)prop-2-yn-1-yl)carbamate (5v)**

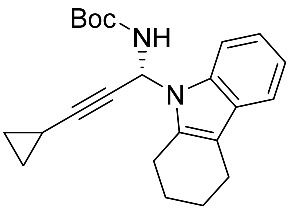

5v

Compound **5v** was obtained as colorless liquid (12.4 mg, 68% yield, 98% ee) after flash chromatography (elution gradient: ethyl acetate:petroleum ether = 1:50); **[α]$_D$^{25}** = + 48.7 (*c* 1.0, $CHCl_3$); **^{1}H-NMR** (400 MHz, $CDCl_3$, ppm): *δ* 7.64 (d, *J* = 6.0 Hz, 1H), 7.47 (d, *J* = 7.6 Hz, 1H), 7.20-7.10 (m, 2H), 6.72 (d, *J* = 6.4 Hz, 1H), 5.70 (br, 1H), 3.09-3.05 (m, 1H), 2.79-2.71 (m, 3H), 1.96-1.86 (m, 4H), 1.41 (s, 9H), 1.28-1.25 (m, 1H), 0.79-0.70 (m, 4H); **^{13}C-NMR** (100 MHz, $CDCl_3$, ppm): *δ* 153.9, 134.9, 134.6, 128.6, 120.9, 119.5, 118.0, 110.6, 89.3, 80.8, 70.7, 52.2, 28.4, 23.4, 23.1, 22.4, 21.1, 8.3, 8.2, -0.5; **HRMS** calcd. for $C_{23}H_{28}N_2NaO_2$ $[M+Na]^+$: 387.2043, found: 387.2043; **HPLC analysis:** Daicel CHIRALPAK AD-H, *n*-hexane:*i*-PrOH = 90:10, flow rate = 0.8 mL·min^{-1}, λ = 254 nm, retention time: t_R = 5.6 min (major), t_R = 6.4 min (minor).

***Tert*-butyl (*R*)-(1-(2,3-dihydrocyclopenta[b]indol-4(1H)-yl)-3-phenylprop-2-yn-1-yl) carbamate (5w)**

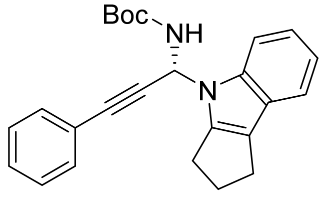

5w

Compound **5w** was obtained as colorless liquid (12.4 mg, 64% yield, 95% ee) after flash chromatography (elution gradient: ethyl acetate:petroleum ether = 1:50); **[α]$_D$^{25}** = + 10.9 (*c* 1.0, $CHCl_3$); **^{1}H-NMR** (400 MHz, $CDCl_3$, ppm): *δ* 7.70 (br, 1H), 7.45-7.44 (m, 3H), 7.37-7.31 (m, 3H), 7.19-7.17 (m, 1H), 7.14-7.11 (m, 1H), 7.03 (d, *J* = 4.0 Hz, 1H), 5.69 (br, 1H), 3.22-3.19 (m, 1H), 3.06-3.01 (m, 1H), 2.85-2.83 (m, 2H), 2.59-2.54 (m, 2H), 1.44 (s, 9H); **^{13}C-NMR** (100 MHz, $CDCl_3$, ppm): *δ* 154.0, 144.7, 139.8, 131.9,

129.2, 128.5, 125.3, 121.7, 120.8, 120.2, 120.1, 118.8, 111.1, 85.6, 84.1, 81.0, 53.9, 28.7, 28.4, 26.5, 24.5; **HRMS** calcd. for $C_{25}H_{26}N_2NaO_2$ [M+Na]$^+$: 409.1886, found: 409.1886; **HPLC analysis:** Daicel CHIRALCEL OD-H, *n*-hexane:*i*-PrOH = 95:5, flow rate = 0.8 mL·min^{-1}, λ = 254 nm, retention time: t_R = 9.2 min (major), t_R = 12.4 min (minor).

***Tert*-butyl (*R*)-(3-phenyl-1-(7,8,9,10-tetrahydrocyclohepta[b]indol-5(6H)-yl)prop-2-yn-1-yl)carbamate (5x)**

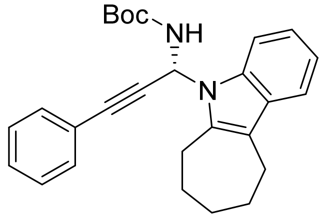

5x

Compound **5x** was obtained as colorless liquid (12.6 mg, 61% yield, 90% ee) after flash chromatography (elution gradient: ethyl acetate:petroleum ether = 1:50); **[α]$_D$^{25}** = + 7.0 (*c* 1.0, $CHCl_3$); **^{1}H-NMR** (400 MHz, $CDCl_3$, ppm): *δ* 7.75 (br, 1H), 7.50-7.48 (m, 1H), 7.42-7.40 (m, 2H), 7.35-7.28 (m, 3H), 7.19-7.11 (m, 3H), 5.78 (br, 1H), 3.26-3.22 (m, 1H), 3.03-2.999 (m, 1H), 2.88-2.84 (m, 1H), 2.80-2.75 (m, 1H), 1.97-1.70 (m, 6H), 1.42 (s, 9H); **^{13}C-NMR** (100 MHz, $CDCl_3$, ppm): *δ* 154.0, 138.2, 134.4, 131.9, 129.1, 129.0, 128.5, 121.9, 121.0, 119.7, 118.0, 115.8, 110.5, 85.5, 84.7, 81.1, 52.6, 31.6, 28.4, 28.1, 27.1, 27.0, 24.2; **HRMS** calcd. for $C_{27}H_{30}N_2NaO_2$ [M+Na]$^+$: 437.2199, found: 437.2199; **HPLC analysis:** Daicel CHIRALCEL OD-H, *n*-hexane:*i*-PrOH = 95:5, flow rate = 0.8 mL·min^{-1}, λ = 254 nm, retention time: t_R = 7.5 min (major), t_R = 10.1 min (minor).

***Tert*-butyl (*R*)-(1-(3-methyl-2-phenyl-1H-indol-1-yl)-3-phenylprop-2-yn-1-yl)carbamate (5y)**

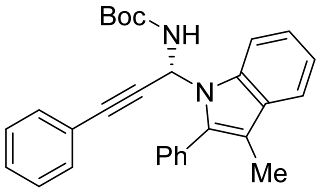

5y

Compound **5y** was obtained as colorless liquid (16.2 mg, 72% yield, 98% ee) after flash chromatography (elution gradient: ethyl acetate:petroleum ether = 1:50); **[α]$_D^{25}$** = + 5.2 (*c* 1.0, $CHCl_3$); **^{1}H-NMR** (400 MHz, $CDCl_3$, ppm): *δ* 7.85 (d, *J* = 8.0 Hz, 1H), 7.61 (d, *J* = 8.0 Hz, 1H), 7.54-7.45 (m, 5H), 7.41-7.28 (m, 6H), 7.24-7.20 (m, 1H), 6.91 (d, *J* = 8.0 Hz, 1H), 5.66 (br, 1H), 2.23 (s, 3H), 1.36 (s, 9H); **^{13}C-NMR** (100 MHz, $CDCl_3$, ppm): *δ* 153.3, 135.3, 131.9, 131.8, 131.1, 129.9, 129.0, 128.6, 128.4, 122.5, 121.9, 120.3, 119.4, 111.2, 110.8, 85.3, 84.9, 80.7, 54.1, 28.3, 9.4; **HRMS** calcd. for $C_{29}H_{28}N_2NaO_2$ [M+Na]$^+$: 437.2224, found: 437.2223; **HPLC analysis:** Daicel CHIRALPAK AD-H, *n*-hexane:*i*-PrOH = 90:10, flow rate = 0.8 mL·min^{-1}, λ = 254 nm, retention time: t_R = 8.5 min (minor), t_R = 11.8 min (major).

***Tert*-butyl (*R*)-(1-(3-benzyl-2-methyl-1H-indol-1-yl)-3-phenylprop-2-yn-1-yl)carbamate (5z)**

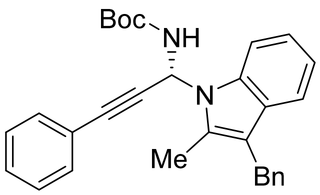

5z

Compound **5z** was obtained as colorless liquid (16.7 mg, 74% yield, 99% ee) after flash chromatography (elution gradient: ethyl acetate:petroleum ether = 1:50); **[α]$_D^{25}$** = -102.5 (*c* 1.0, $CHCl_3$); **^{1}H-NMR** (400 MHz, $CDCl_3$, ppm): *δ* 7.76 (d, *J* = 7.6 Hz, 1H), 7.43-7.41 (m, 3H), 7.35-7.27 (m, 3H), 7.24-7.18 (m, 5H), 7.16-7.05 (m, 3H), 5.88 (d, *J* = 7.2 Hz, 1H), 4.08 (s, 2H), 2.60 (s, 3H), 1.42 (s, 9H); **^{13}C-NMR** (100 MHz, $CDCl_3$,

ppm): δ 154.0, 141.5, 134.8, 133.0, 131.9, 129.5, 129.2, 128.5, 128.4, 128.4, 121.7, 121.2, 119.9, 118.8, 111.5, 110.6, 85.6, 84.2, 81.1, 53.0, 30.4, 28.4, 10.9; **HRMS** calcd. for $C_{30}H_{30}N_2NaO_2$ [M+Na]$^+$: 473.2199, found: 473.2198; **HPLC analysis:** Daicel CHIRALPAK IC, *n*-hexane:*i*-PrOH = 97:3, flow rate = 0.8 mL·min^{-1}, λ = 254 nm, retention time: t_R = 10.8 min (major).

Supplementary Note 4

Synthetic application of the N-propargylation products

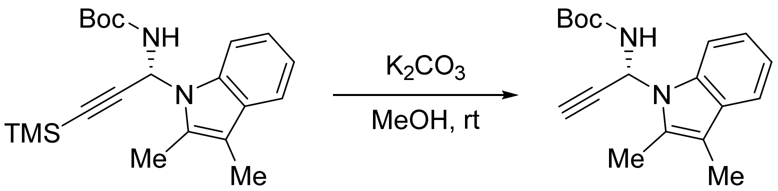

5k **6**

Synthesis of compound 6: Solid potassium carbonate (27.6 mg, 0.2 mmol) was added to a solution of **5k** (37.0 mg, 0.1 mmol, 97% ee) in MeOH (1 mL). The reaction mixture was allowed to react at room temperature for 1 h. Then, the reaction was concentrated and purified by silica gel column chromatography, using ethyl acetate:petroleum ether (1:30-1:10) as the eluent, affording compound **6** as a white solid (23.2 mg, 78% yield, 97% ee); **[α]$_D^{25}$** = - 36.0 (*c* 1.0, $CHCl_3$); **^{1}H-NMR** (400 MHz, $CDCl_3$, ppm): δ 7.65 (d, *J* = 7.6 Hz, 1H), 7.51 (d, *J* = 7.6 Hz, 1H), 7.23-7.14 (m, 2H), 6.87 (d, *J* = 7.6 Hz, 1H), 5.82 (d, *J* = 6.4 Hz, 1H), 2.62 (d, *J* = 2.4 Hz, 1H), 2.53 (s, 3H), 2.24 (s, 3H), 1.42 (s, 9H); **^{13}C-NMR** (100 MHz, $CDCl_3$, ppm): δ 153.9, 134.4, 131.7, 129.9, 121.1, 119.8, 118.5, 110.3, 108.3, 81.2, 78.9, 74.0, 52.2, 28.3, 10.5, 8.9; **HRMS** calcd. for $C_{18}H_{22}N_2NaO_2$ [M+Na]$^+$: 321.1573, found: 321.1573; **HPLC analysis:** Daicel CHIRALPAK AD-H, *n*-hexane:*i*-PrOH = 90:10, flow rate = 0.8 mL·min^{-1}, λ = 254 nm, retention time: t_R = 6.2 min (major), t_R = 7.4 min (minor).

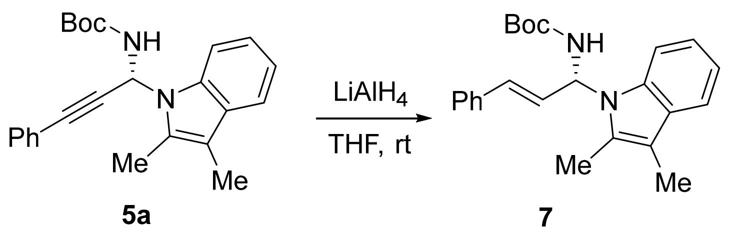

Synthesis of compound 7: A solution of $LiAlH_4$ in THF (1 M, 0.2 mL) was added dropwise to a solution of **5a** (37.4 mg, 0.1 mmol, 99% ee) in THF (0.8 mL) at 0 ºC under nitrogen. The reaction mixture was allowed to stir at room temperature for 8 h. Then the reaction was quenched with water (2 mL). The aqueous layer was extracted with ethyl acetate (3×5 mL). The combined organic layer was washed with brine (10 mL), dried over Na_2SO_4 and concentrated to afford the residue mixture. The residue mixture was purified by silica gel column chromatography with the eluent of ethyl acetate:petroleum ether (1:30-1:20), affording compound **7** as a colorless liquid (25.2 mg, 67% yield, 98% ee). $[\alpha]_D^{25}$ = - 30.6 (*c* 1.0, $CHCl_3$); **^{1}H-NMR** (400 MHz, $CDCl_3$, ppm): δ 7.51-7.49 (m, 1H), 7.35-7.25 (m, 6H), 7.11-7.05 (m, 2H), 6.81 (br, 1H), 6.61 (d, J = 16.0 Hz, 1H), 6.47 (d, J = 16.0 Hz, 1H), 5.59 (d, J = 6.4 Hz, 1H), 2.49 (s, 3H), 2.25 (s, 3H), 1.42 (s, 9H); **^{13}C-NMR** (100 MHz, $CDCl_3$, ppm): δ 154.7, 135.9, 134.7, 132.3, 129.9, 128.8, 128.4, 127.4, 126.9, 121.0, 119.3, 118.4, 110.5, 107.8, 80.7, 61.9, 28.4, 10.8, 9.0; **HRMS** calcd. for $C_{24}H_{28}N_2NaO_2$ $[M+Na]^+$: 399.2043, found: 399.2043; **HPLC analysis:** Daicel CHIRALPAK IC, *n*-hexane:*i*-PrOH = 95:5, flow rate = 0.8 mL·min^{-1}, λ = 254 nm, retention time: t_R = 7.6 min (minor), t_R = 10.0 min (major).

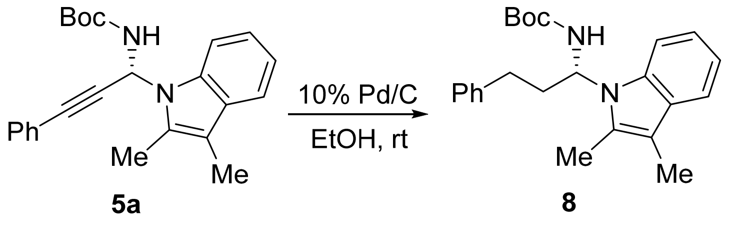

Synthesis of compound 8: Under hydrogen atmosphere, a solution of **5a** (37.4 mg, 0.1 mmol, 99% ee) in anhydrous EtOH (10 mL) was stirred with Pd/C (5 %, 10 mg) for 1 h. The reaction mixture was then filtered through silica gel with the elution of EtOAc. The solvent was removed under reduced pressure to give the product **8** as a colorless

liquid (99% yield, 99% ee). **[α]$_D^{25}$** = - 51.8 (*c* 1.0, $CHCl_3$); **^{1}H-NMR** (400 MHz, $CDCl_3$, ppm): *δ* 7.50-7.48 (m, 1H), 7.35-7.33 (m, 1H), 7.26-7.22 (m, 2H), 7.19-7.15 (m, 1H), 7.10-7.05 (m, 4H), 5.97 (br, 1H), 5.50 (br, 1H), 2.55-2.40 (m, 4H), 2.33 (s, 3H), 2.21 (s, 3H), 1.38 (s, 9H); **^{13}C-NMR** (100 MHz, $CDCl_3$, ppm): *δ* 154.7, 140.4, 134.0, 132.9, 130.1, 129.5, 128.6, 128.5, 126.3, 120.7, 119.0, 118.5, 110.2, 107.2, 80.3, 61.8, 36.3, 32.1, 28.4, 10.8, 8.9; **HRMS** calcd. for $C_{24}H_{30}N_2NaO_2$ $[M+Na]^+$: 401.2199, found: 401.2199; **HPLC analysis:** Daicel CHIRALPAK AD-H, *n*-hexane:*i*-PrOH = 85:15, flow rate = 0.8 mL·min^{-1}, λ = 230 nm, retention time: t_R = 6.3 min (major), t_R = 7.6 min (minor).

Supplementary Note 5

General procedure for the N-benzylation of carbazoles

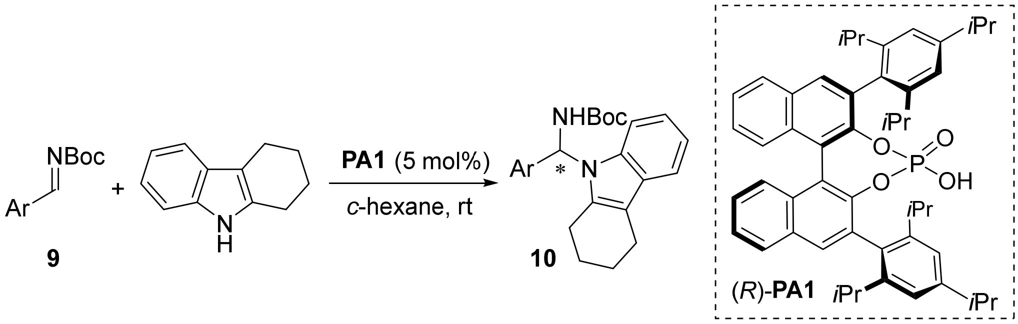

To a solution of **9** (0.05 mmol) and tetrahydrocarbazole (0.06 mmol) in *c*-hexane (1.0 mL) was added the catalyst (*R*)-**PA1** (2.0 mg, 5 mol%) at rt. After stirring for 12 h, the mixture was directly purified by silica gel chromatography (ethyl acetate:petroleum ether = 1:50 to 1:30) to afford the products **10**.

Tert-butyl (phenyl(1,2,3,4-tetrahydro-9H-carbazol-9-yl)methyl)carbamate (10a)

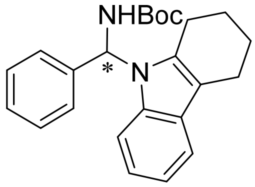

10a

Compound **10a** was obtained as white solid (16.0 mg, 84% yield, 90% ee) after flash

chromatography (elution gradient: ethyl acetate:petroleum ether = 1:40); **[α]$_D^{25}$** = - 105.3 (*c* 1.0, $CHCl_3$); **^{1}H-NMR** (400 MHz, $CDCl_3$, ppm): *δ* 7.51 (d, *J* = 7.6 Hz, 1H), 7.34-7.28 (m, 3H), 7.18-7.17 (m, 2H), 7.10-6.89 (m, 3H), 5.74 (d, *J* = 9.2 Hz, 1H), 2.89-2.71 (m, 4H), 1.95-1.89 (m, 4H), 1.47 (s, 9H); **^{13}C-NMR** (100 MHz, $CDCl_3$, ppm): *δ* 155.0, 138.7, 135.9, 134.9, 128.9, 128.7, 128.3, 126.2, 120.9, 119.4, 118.1, 110.7, 80.7, 62.7, 28.4, 23.4, 23.1, 22.7, 21.2; **HRMS** calcd. for $C_{24}H_{28}N_2NaO_2$ $[M+Na]^+$: 399.2043, found: 399.2043; **HPLC analysis:** Daicel CHIRALPAK AD-H, *n*-hexane:*i*-PrOH = 95:5, flow rate = 0.8 mL·min^{-1}, λ = 254 nm, retention time: t_R = 7.4 min (major), t_R = 10.2 min (minor).

***Tert*-butyl ((1,2,3,4-tetrahydro-9H-carbazol-9-yl)(*p*-tolyl)methyl)carbamate (10b)**

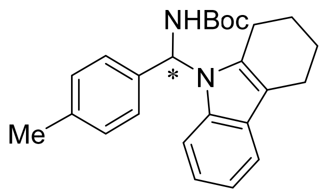

10b

Compound **10b** was obtained as white solid (16.6 mg, 85% yield, 90% ee) after flash chromatography (elution gradient: ethyl acetate:petroleum ether = 1:40); **[α]$_D^{25}$** = - 84.7 (*c* 1.0, $CHCl_3$); **^{1}H-NMR** (400 MHz, $CDCl_3$, ppm): *δ* 7.51 (d, *J* = 7.6 Hz, 1H), 7.24-7.13 (m, 3H), 7.10-6.92 (m, 5H), 5.72 (d, *J* = 8.4 Hz, 1H), 2.90-2.70 (m, 4H), 2.35 (s, 3H), 1.95-1.89 (m, 4H), 1.47 (s, 9H); **^{13}C-NMR** (100 MHz, $CDCl_3$, ppm): *δ* 155.0, 138.0, 135.9, 135.7, 135.0, 129.6, 128.7, 126.1, 120.9, 119.3, 118.1, 110.8, 80.6, 62.7, 28.4, 23.5, 23.2, 22.8, 21.2, 21.2; **HRMS** calcd. for $C_{25}H_{30}N_2NaO_2$ $[M+Na]^+$: 413.2199, found: 413.2199; **HPLC analysis:** Daicel CHIRALPAK AD-H, *n*-hexane:*i*-PrOH = 95:5, flow rate = 0.8 mL·min^{-1}, λ = 254 nm, retention time: t_R = 8.6 min (major), t_R = 11.5 min (minor).

***Tert*-butyl ((4-fluorophenyl)(1,2,3,4-tetrahydro-9H-carbazol-9-yl)methyl) carbamate (10b)**

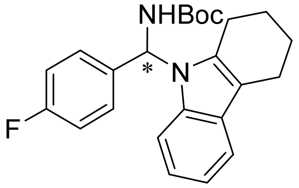

10c

Compound **10c** was obtained as white solid (13.2 mg, 67% yield, 91% ee) after flash chromatography (elution gradient: ethyl acetate:petroleum ether = 1:40); **$[\alpha]_D^{25}$** = - 86.9 (*c* 1.0, $CHCl_3$); **^{1}H-NMR** (400 MHz, $CDCl_3$, ppm): δ 7.50 (d, J = 7.6 Hz, 1H), 7.22-7.20 (m, 1H), 7.15-7.06 (m, 3H), 7.03-6.99 (m, 3H), 6.85 (d, J = 7.2 Hz, 1H), 5.68 (d, J = 8.4 Hz, 1H), 2.86-2.68 (m, 4H), 1.94-1.88 (m, 4H), 1.45 (s, 9H); **^{13}C-NMR** (100 MHz, $CDCl_3$, ppm): δ 164.0, 161.5, 154.9, 135.8, 134.8, 134.5, 134.5, 129.2, 128.8, 128.4, 128.1, 128.0, 121.1, 119.5, 118.2, 115.9, 115.7, 111.0, 110.7, 80.9, 62.4, 28.4, 23.4, 23.1, 22.8, 21.2; **HRMS** calcd. for $C_{24}H_{27}FN_2NaO_2$ $[M+Na]^+$: 417.1949, found: 417.1949; **HPLC analysis:** Daicel CHIRALPAK AD-H, *n*-hexane:*i*-PrOH = 95:5, flow rate = 0.8 mL·min^{-1}, λ = 254 nm, retention time: t_R = 8.1 min (major), t_R = 11.5 min (minor).

Supplementary Note 6

Determination of the absolute stereochemistry for N-proparglation products

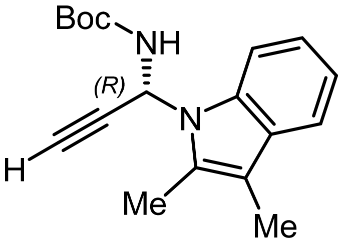

6
CCDC No.: 1881884
(30% ellipsoid probability)

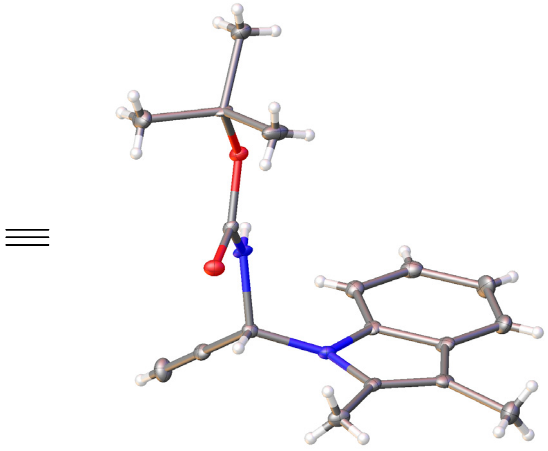

Supplementary Figure 1. Crystal Structure of (*R*)-**6**

Supplementary Table 1. Crystal data and structure refinement for **6**.

Identification code	**6**
Empirical formula	$C_{18}H_{22}N_2O_2$
Formula weight	298.37
Temperature/K	100(2)
Crystal system	monoclinic
Space group	$P2_1$
a/Å	19.3422(7)
b/Å	17.6581(6)
c/Å	22.7455(8)
α/°	90
β/°	114.736(2)
γ/°	90
Volume/Å^3	7055.8(4)
Z	16
ρ_{calc}g/cm^3	1.124
μ/mm^{-1}	0.587
F(000)	2560.0
Crystal size/mm^3	0.700 × 0.280 × 0.180
Radiation	CuKα (λ = 1.54178)
2Θ range for data collection/°	4.278 to 138.718
Index ranges	-23 ≤ h ≤ 23, -20 ≤ k ≤ 21, -27 ≤ l ≤ 27
Reflections collected	79016
Independent reflections	22499 [R_{int} = 0.0591, R_{sigma} = 0.0585]
Data/restraints/parameters	22499/1/1626

Goodness-of-fit on F^2	1.105
Final R indexes [I>=2σ (I)]	$R_1 = 0.0899$, $wR_2 = 0.2399$
Final R indexes [all data]	$R_1 = 0.0910$, $wR_2 = 0.2405$
Largest diff. peak/hole / e Å^{-3}	0.41/-0.38
Flack parameter	0.15(6)

Supplementary Note 7

Control experiments for reaction mechanism.

Supplementary Table 2. Enantioselectivity of **3a** and recovered **1a** over time [a]

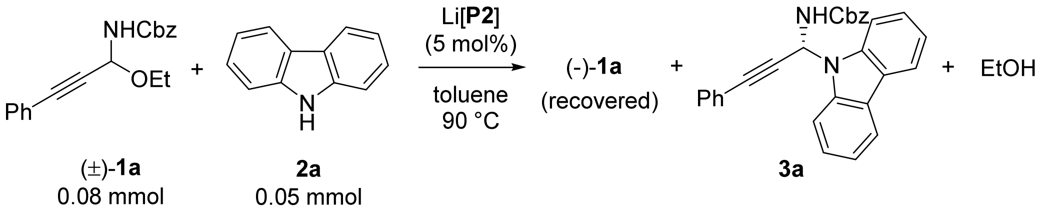

entry	t (h)	*ee* of recovered **1a** (%)	yield of **3a** (%)	*ee* of **3a** (%)
1	3	38	42	94
2	6	49	54	94
3	9	47	62	93
4	12	36	70	93
5	15	24	72	91
6	18	16	70	89
7	24	12	71	86

[a] Reaction conditions: **(±)-1a** (0.08 mmol), **2a** (0.05 mmol), PhMe (1 mL). Yields refer to isolated product. Ee's are determined by HPLC analysis on a chiral stationary phase.

Supplementary Table 3. Kinetic profile for **1a**. [a]

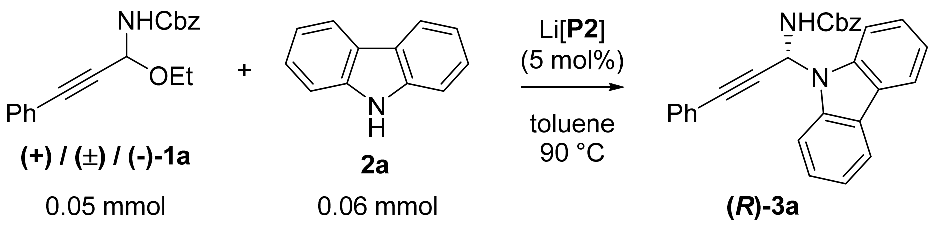

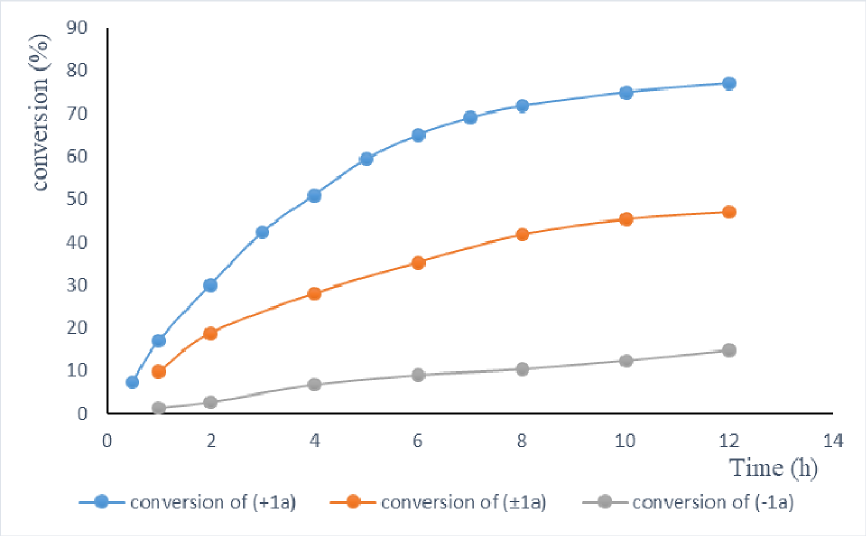

entry	t/h	Conversion of (+)-**1a** (%)	Conversion of (±)-**1a** (%)	Conversion of (-)-**1a** (%)
1	0.5	7.3±0.6	/	/
2	1	17.0±1.7	9.7±0.6	1.3±0.6
3	2	30.0±1.0	18.7±1.2	2.7±0.6
4	3	42.3±2.5	/	/
5	4	51.0±1.7	28.0±1.0	6.7±1.2
6	5	59.3±1.2	/	/
7	6	65.0±2.6	35.3±1.2	9.0±1.0
8	7	69.0±1.7	/	/
9	8	71.7±1.5	41.7±1.5	10.3±0.6
10	10	75.0±1.7	45.3±0.6	12.3±1.5
11	12	77.0±1.0	47.0±1.0	14.7±1.2

Values are means of three determinations, and error bars are standard deviations.

[a] Reaction conditions: (+)/**(±)/(-)-1a** (0.05 mmol), **2a** (0.06 mmol), PhMe (1 mL). Yields are determined by NMR. Ee's are determined by HPLC analysis on a chiral stationary phase.

Supplementary Table 4. Asymmetric reaction of **(-)-1a** and EtOH [a]

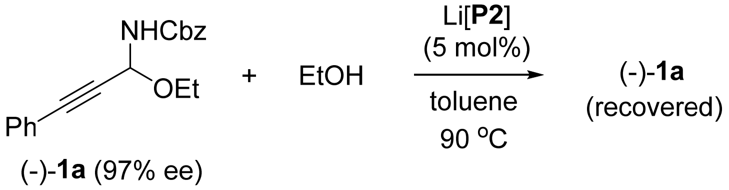

entry	amount of EtOH	t (h)	*ee* of recovered (-)-**1a** (%)
1	0	2	97
2	0	4	97
3	0	6	96
4	0.3 eq.	2	45
5	0.3 eq.	4	26
6	0.3 eq.	6	16

[a] Reaction conditions: **(-)-1a** (0.05 mmol), PhMe (1 mL). Ee's are determined by HPLC analysis on a chiral stationary phase.

Supplementary Table 5. Asymmetric reaction of **(+)-1a** and EtOH [a]

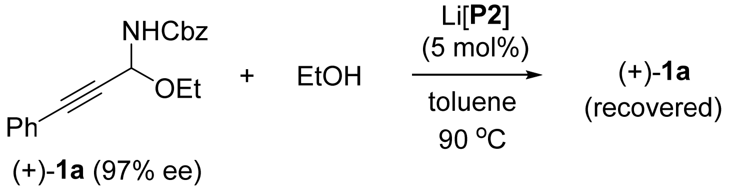

entry	amount of EtOH	t (h)	*ee* of recovered (+)-**1a** (%)
1	0	2	95
2	0	4	91
3	0	6	83
4	0.3 eq.	2	43
5	0.3 eq.	4	23
6	0.3 eq.	6	12

[a] Reaction conditions: **(+)-1a** (0.05 mmol), PhMe (1 mL). Ee's are determined by HPLC analysis on a chiral stationary phase.

Supplementary Table 6. Crossover experiment [a]

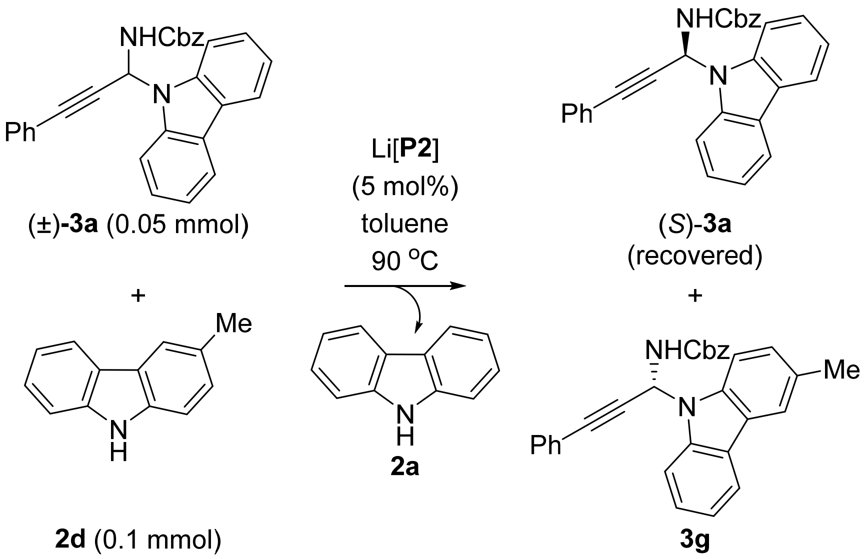

entry	t (h)	yield of recovered **3a** (%)	*ee* of recovered **3a** [(*S*)-**3a**)] (%)	yield of **3g** (%)	*ee* of **3g**(%)
1	6	84	-13	11	86
2	12	75	-26	20	85
3	18	67	-41	28	84
4	24	58	-59	35	82
5	36	53	-76	42	80

[a] Reaction conditions: **(±)-3a** (0.05 mmol), **2d** (0.1 mmol), PhMe (1 mL). Yields refer to isolated product. Ee's are determined by HPLC analysis on a chiral stationary phase.

Supplementary Table 7. The reaction of (*R*)**-3a** with **2a** under the catalysis of Li[**P2**] [a]

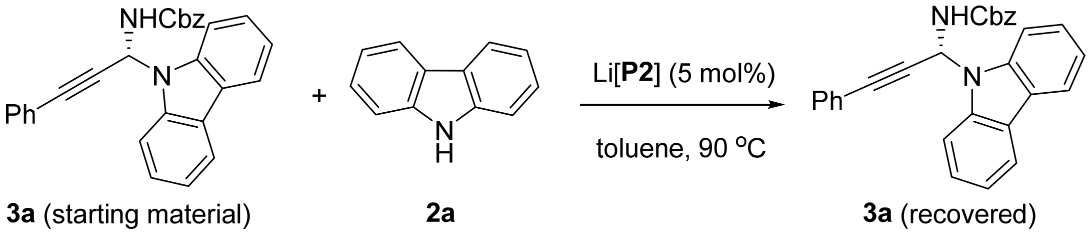

entry	*ee* of starting material **3a** (%)	t (h)	*ee* of recovered **3a** (%)
1	92	3	92
2	92	6	90
3	92	12	88
4	92	24	86
5	70	3	69
6	70	6	69
7	70	12	68
8	70	24	67

[a] Reaction conditions: **(*R*)-3a** (0.05 mmol), **2a** (0.1 mmol), PhMe (1 mL). Ee's were determined by HPLC analysis on a chiral stationary phase.

Supplementary Table 8. The reaction of (*R*)-**3a** with EtOH under the catalysis of Li[**P2**] [a]

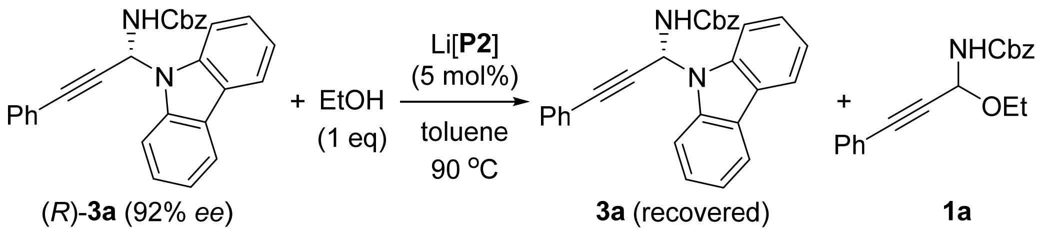

entry	t (h)	yield of recovered **3a** (%)	*ee* of recovered **3a** (%)	yield of **1a** (%)	*ee* of **1a** (%)

1	2	86	90	8	0
2	4	77	88	15	1
3	6	71	85	20	1
4	8	62	81	28	0
5	10	55	75	34	1

[a] Reaction conditions: **(±)-1a** (0.05 mmol), EtOH (0.05 mmol), PhMe (1 mL). Yields refer to isolated product. Ee's are determined by HPLC analysis on a chiral stationary phase.

Supplementary Table 9. The effect of the loading of N,O-acetals [a]

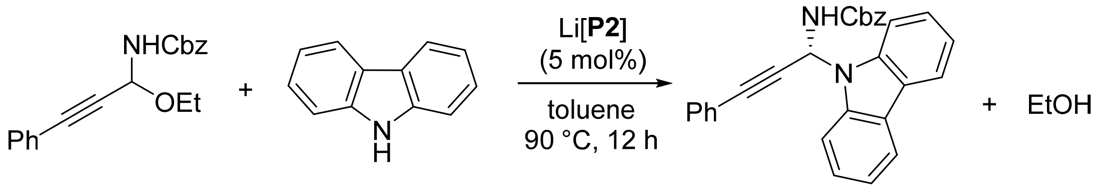

(±)-**1a** (n eq.) **2a** **3a**

entry	n	yield (%)	*ee* (%)
1	0.8	43	85
2	1.0	54	86
3	1.2	63	90
4	1.6	70	93
5	2.0	72	93

[a] Reaction conditions: **2a** (0.05 mmol), PhMe (1 mL). Yields refer to isolated product. Ee's was determined by HPLC analysis on a chiral stationary phase.

Supplementary Table 10. Absence of nonlinear effects [a]

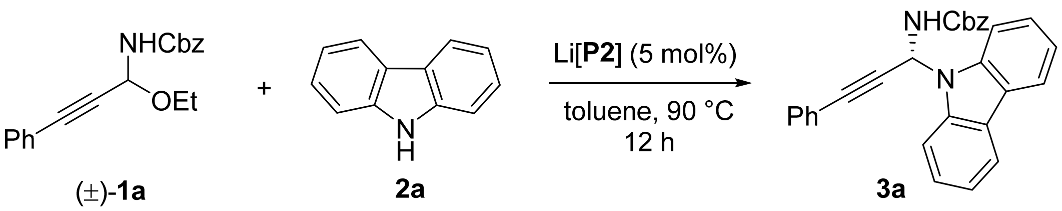

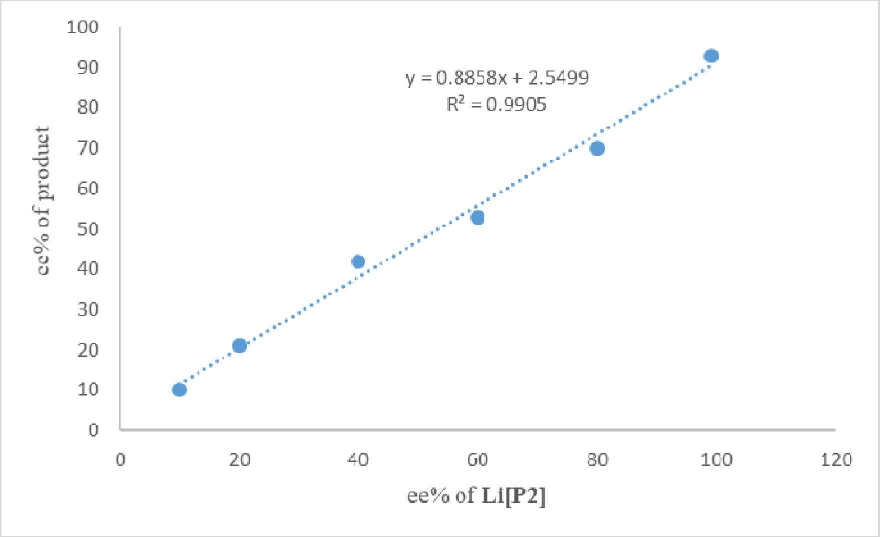

entry	*ee* of Li[**P2**] (%)	*ee* of **3a** (%)
1	10	10
2	20	21
3	40	42
4	60	53
5	80	70
6	>99	93

[a] Reaction conditions: **1a** (0.08 mmol), **2a** (0.05 mmol), PhMe (1 mL). Ee's was determined by HPLC analysis on a chiral stationary phase.

Supplementary Note 8

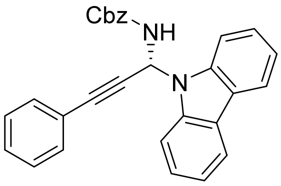

3a

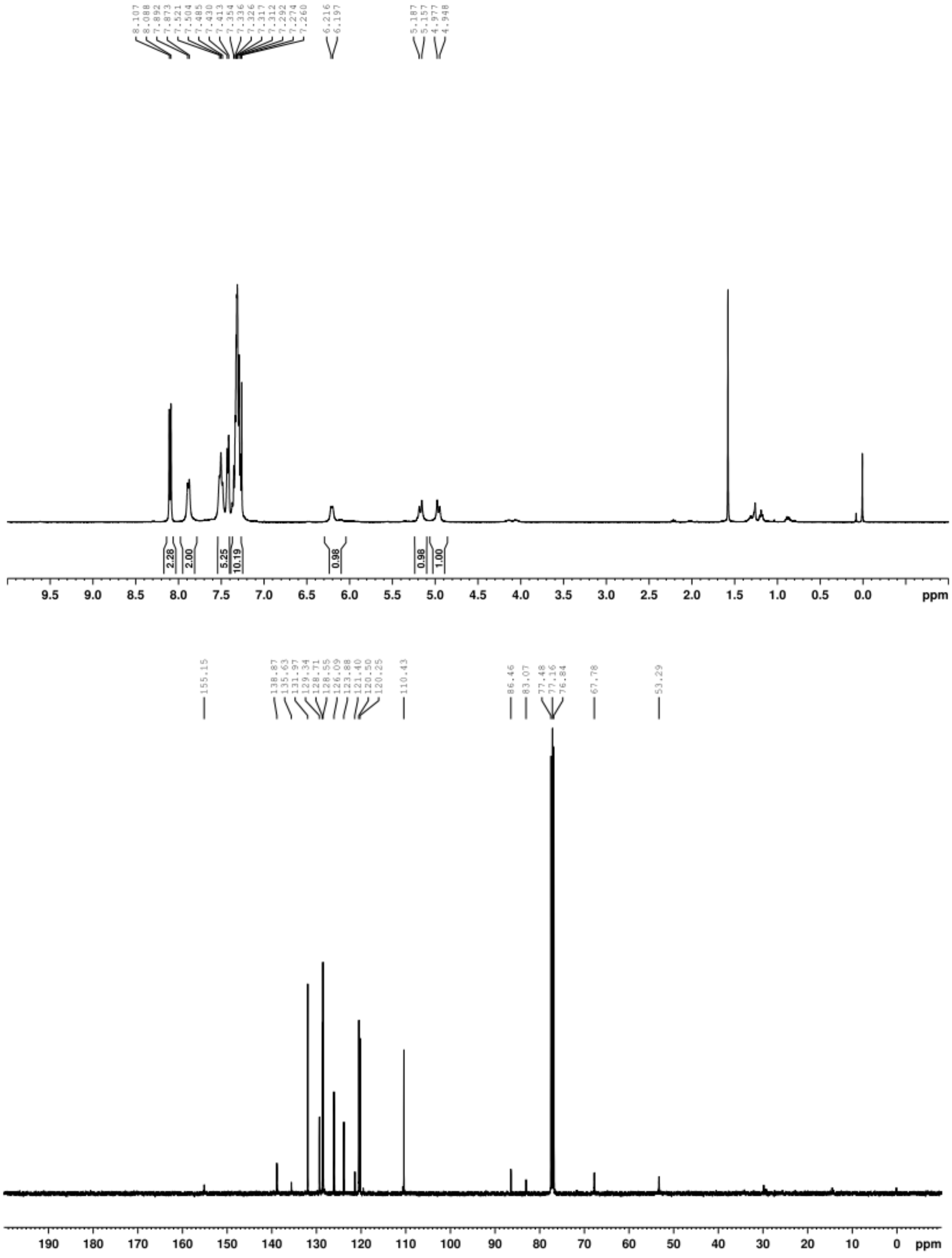

Supplementary Figure 2. ^{1}H and ^{13}C-NMR spectrum for **3a**

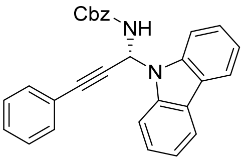

3a

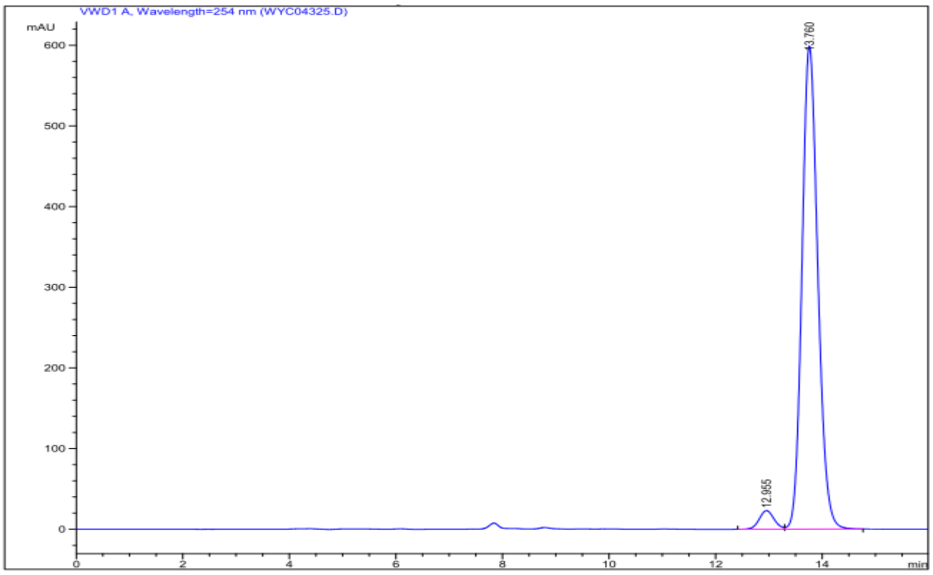

Peak #	RetTime [min]	Type	Width [min]	Area mAU *s	Height [mAU]	Area %
1	12.955	BV	0.2976	445.04633	23.09896	3.4765
2	13.760	VB	0.3194	1.23564e4	598.79895	96.5235

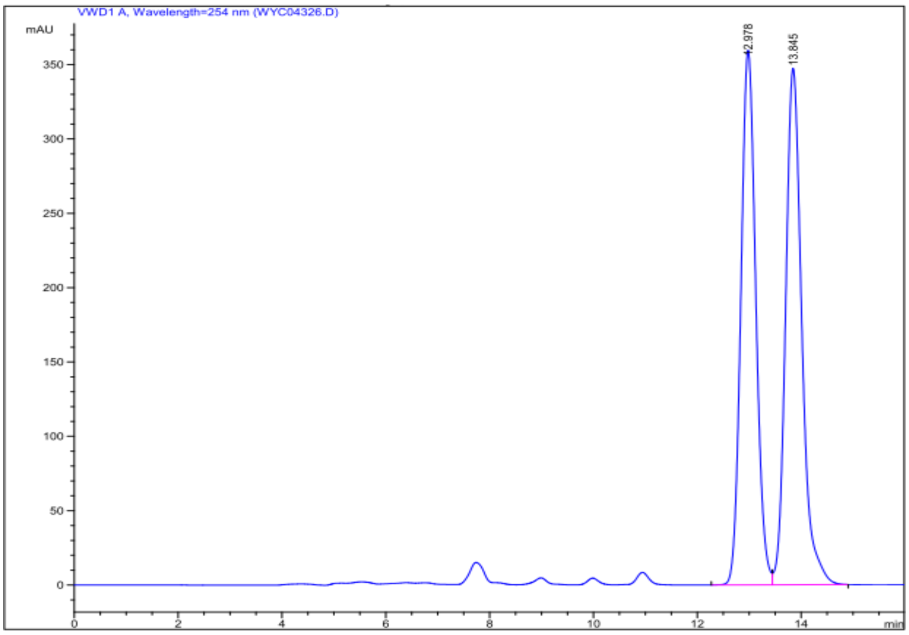

Peak #	RetTime [min]	Type	Width [min]	Area mAU *s	Height [mAU]	Area %
1	12.978	BV	0.3095	7160.20557	359.51477	48.8767
2	13.845	VB	0.3302	7489.32422	347.30011	51.1233

Supplementary Figure 3. HPLC spectrum for **3a**

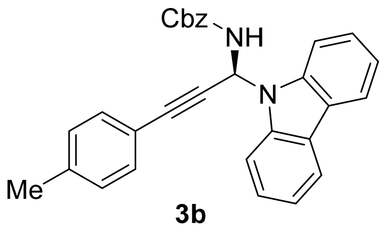

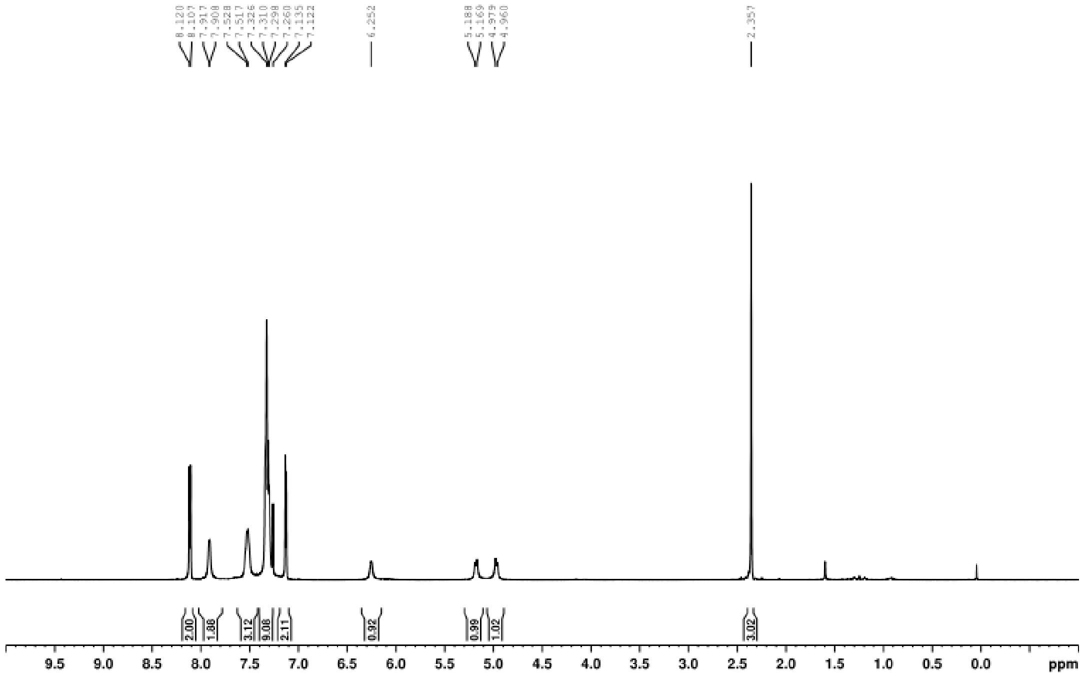

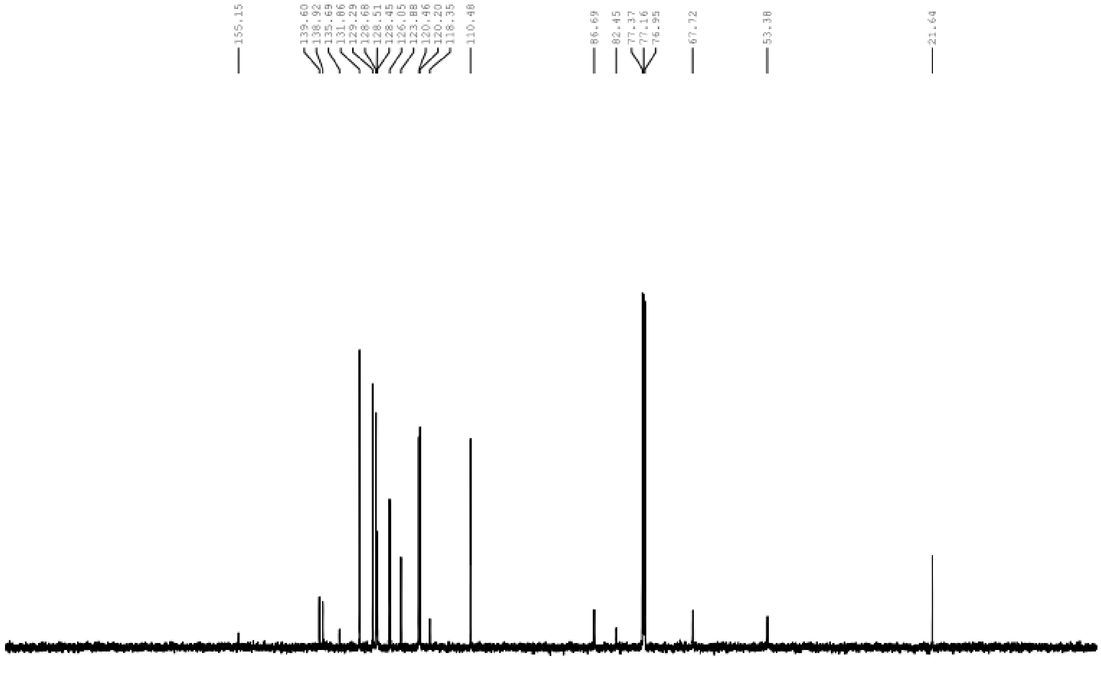

Supplementary Figure 4. ^{1}H and ^{13}C-NMR spectrum for **3b**

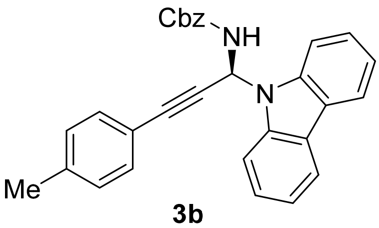

3b

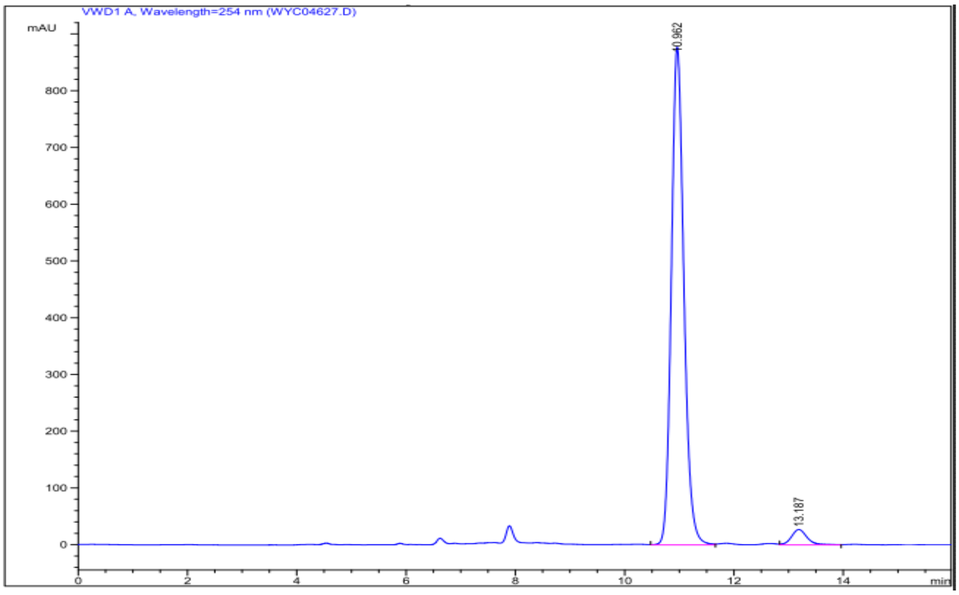

Peak #	RetTime [min]	Type	Width [min]	Area mAU *s	Height [mAU]	Area %
1	10.962	VV	0.2444	1.39933e4	877.01776	96.2684
2	13.187	VV	0.3034	542.41382	27.09637	3.7316

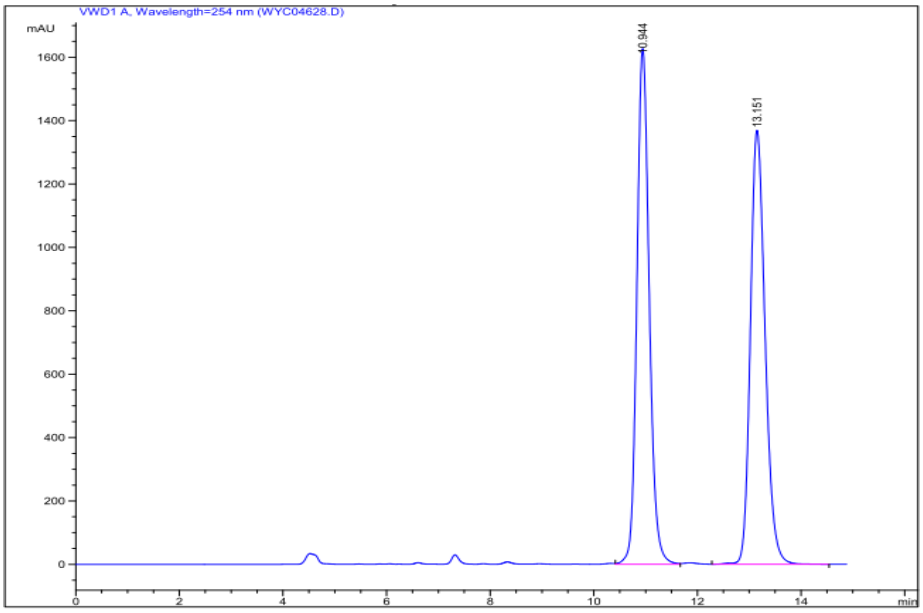

Peak #	RetTime [min]	Type	Width [min]	Area mAU *s	Height [mAU]	Area %
1	10.944	VV	0.2558	2.69423e4	1627.19543	49.9153
2	13.151	VB	0.3045	2.70338e4	1369.55701	50.0847

Supplementary Figure 5. HPLC spectrum for **3b**

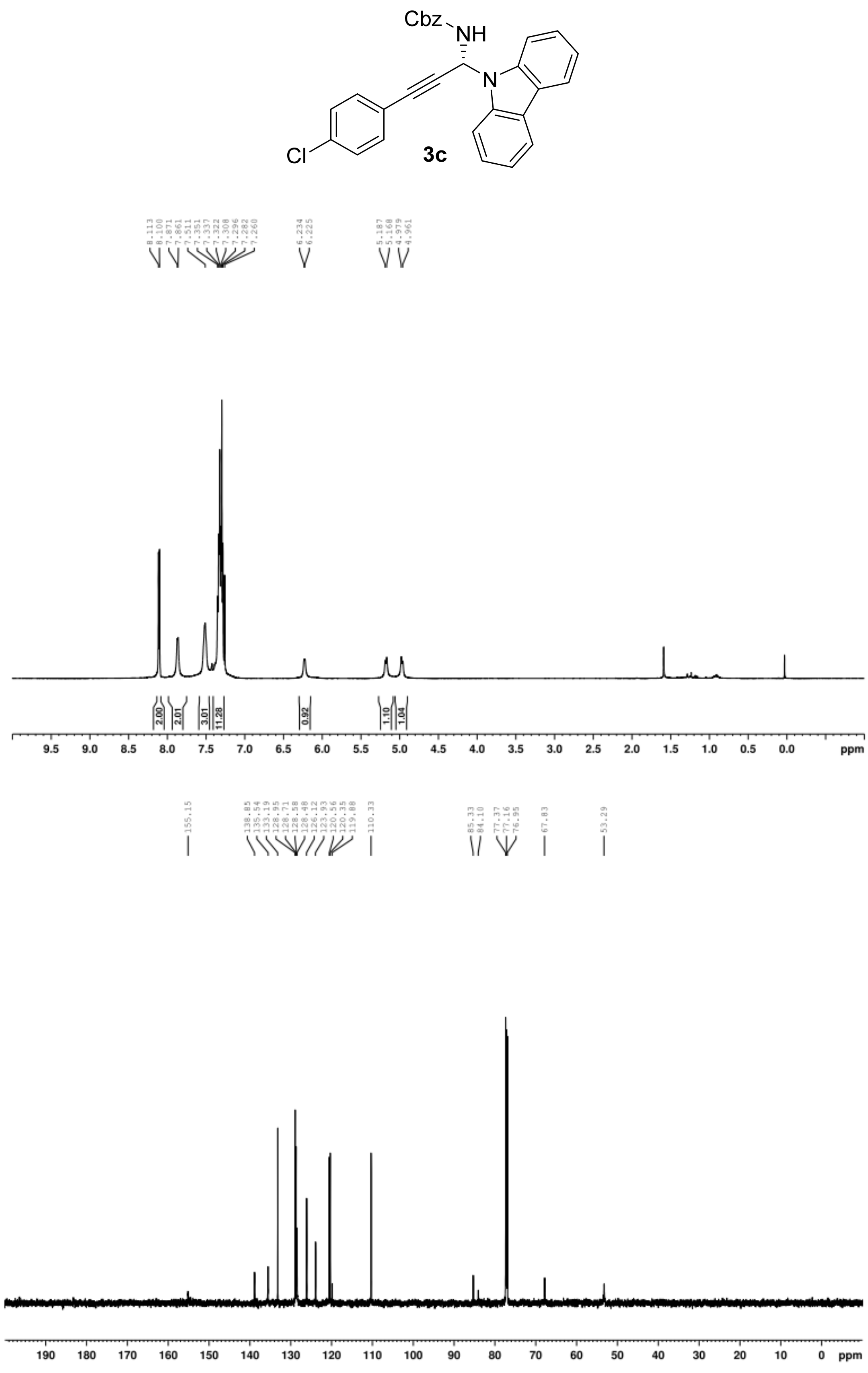

Supplementary Figure 6. ^{1}H and ^{13}C-NMR spectrum for **3c**

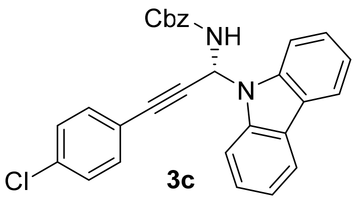

3c

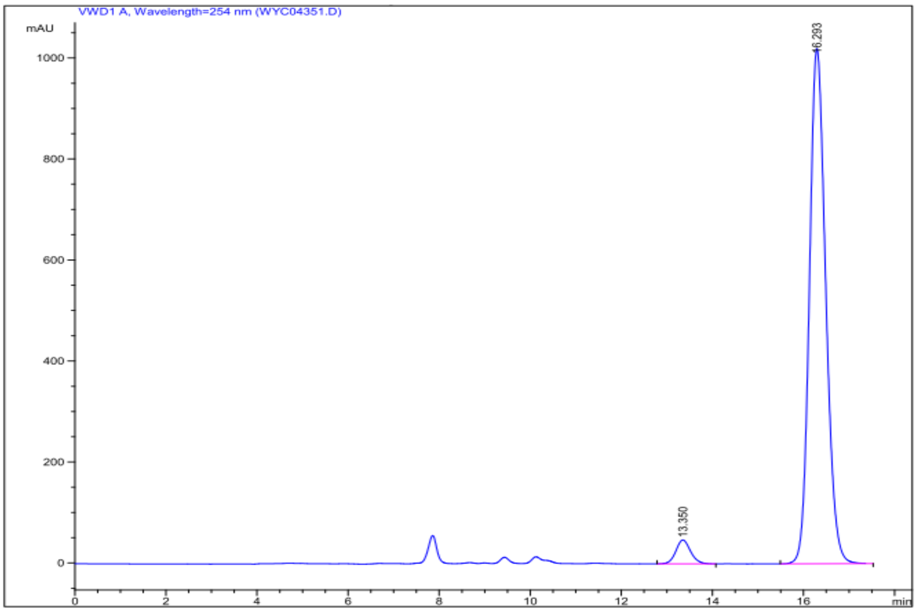

Peak #	RetTime [min]	Type	Width [min]	Area mAU *s	Height [mAU]	Area %
1	13.350	BB	0.3427	1058.73657	47.28767	4.0082
2	16.293	PB	0.3860	2.53553e4	1020.38257	95.9918

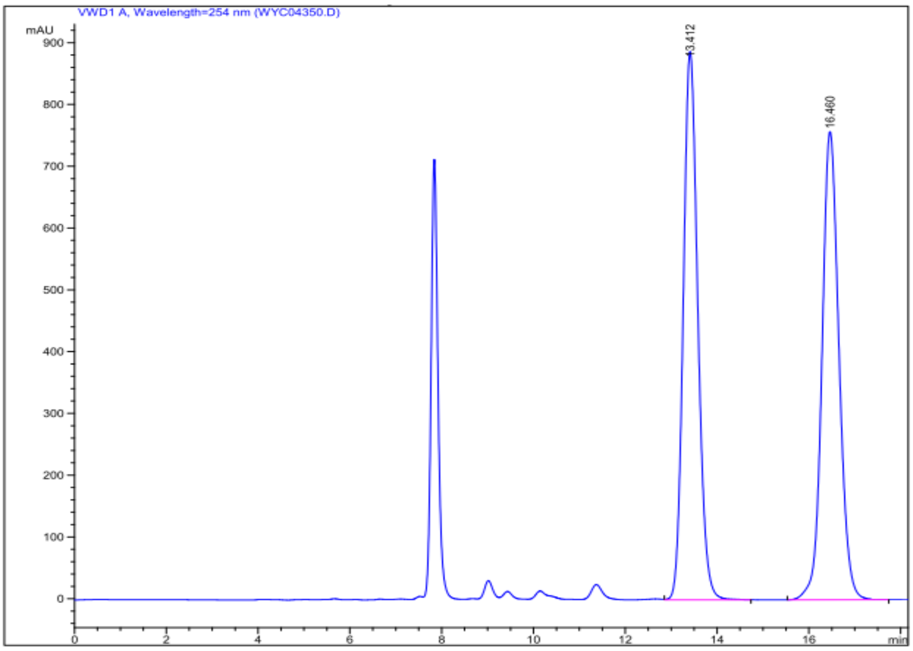

Peak #	RetTime [min]	Type	Width [min]	Area mAU *s	Height [mAU]	Area %
1	13.412	VB	0.3373	1.94584e4	887.47784	50.0399
2	16.460	PB	0.3952	1.94274e4	757.75055	49.9601

Supplementary Figure 7. HPLC spectrum for **3c**

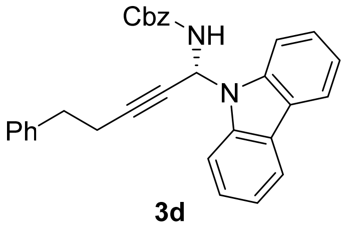

3d

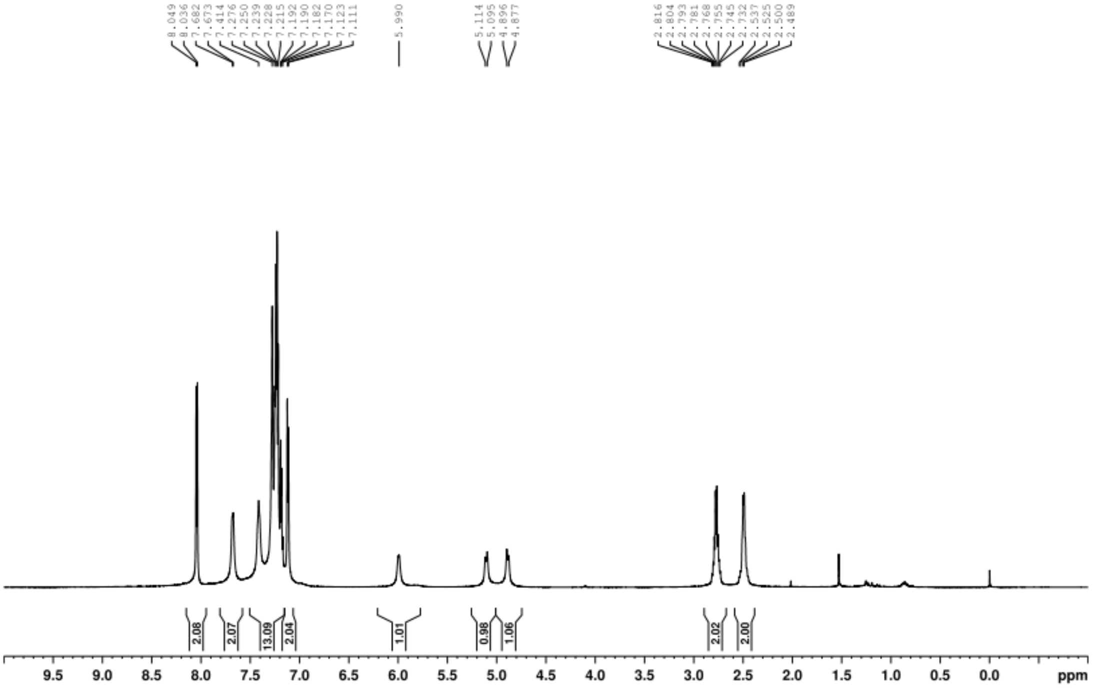

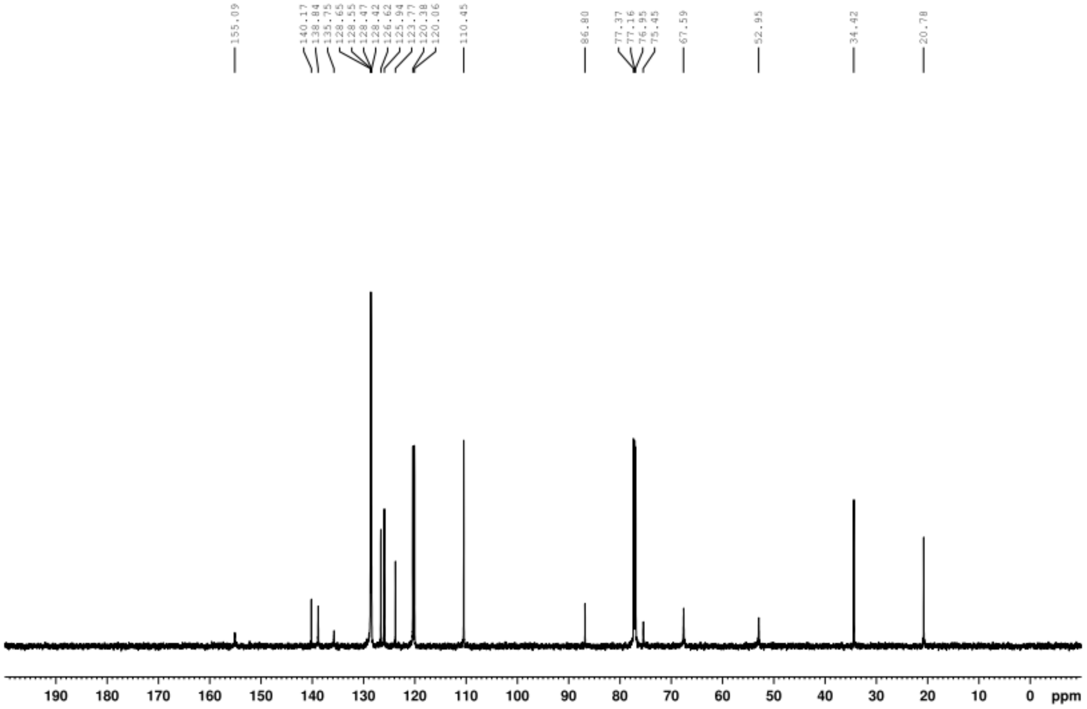

Supplementary Figure 8. ^{1}H and ^{13}C-NMR spectrum for **3d**

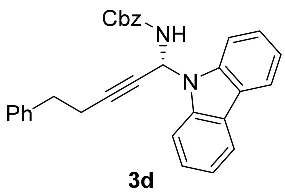

3d

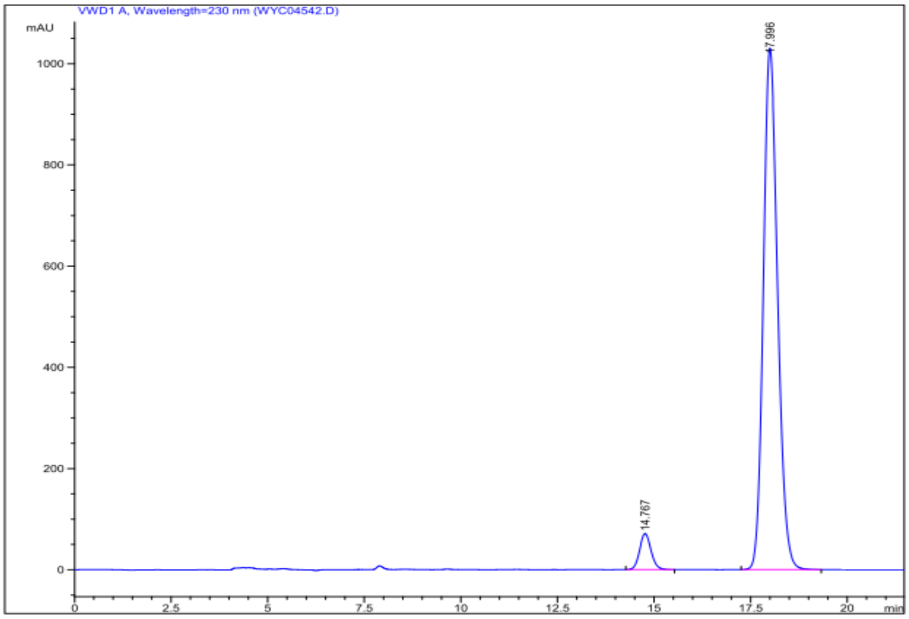

Peak #	RetTime [min]	Type	Width [min]	Area mAU *s	Height [mAU]	Area %
1	14.767	BB	0.3160	1460.04541	71.31631	5.2353
2	17.996	BB	0.3967	2.64286e4	1030.55396	94.7647

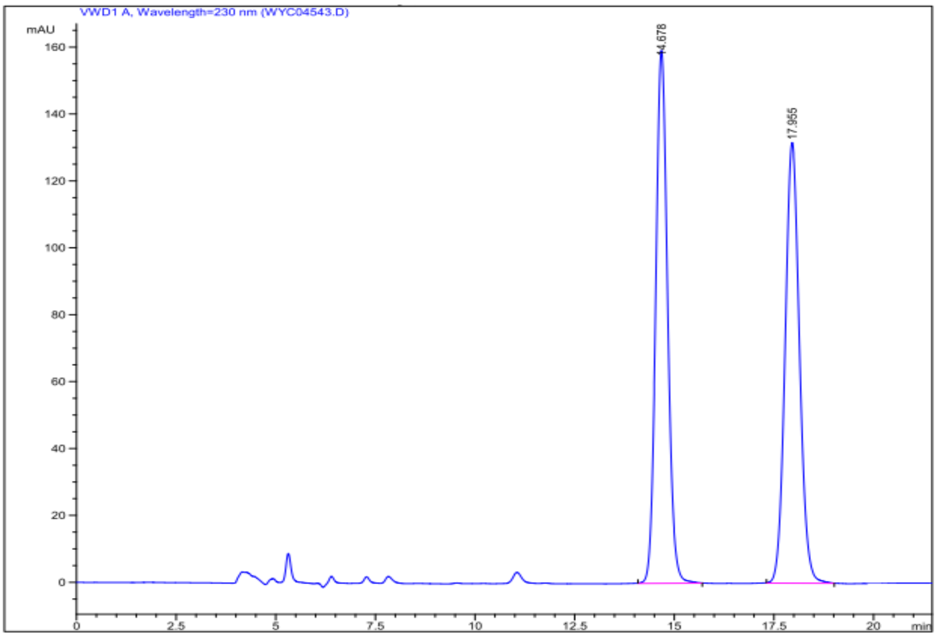

Peak #	RetTime [min]	Type	Width [min]	Area mAU *s	Height [mAU]	Area %
1	14.678	BB	0.3168	3271.90454	159.28456	49.7937
2	17.955	BB	0.3868	3299.01562	131.73601	50.2063

Supplementary Figure 9. HPLC spectrum for **3d**

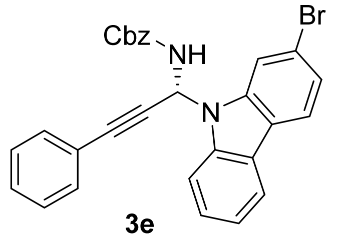

3e

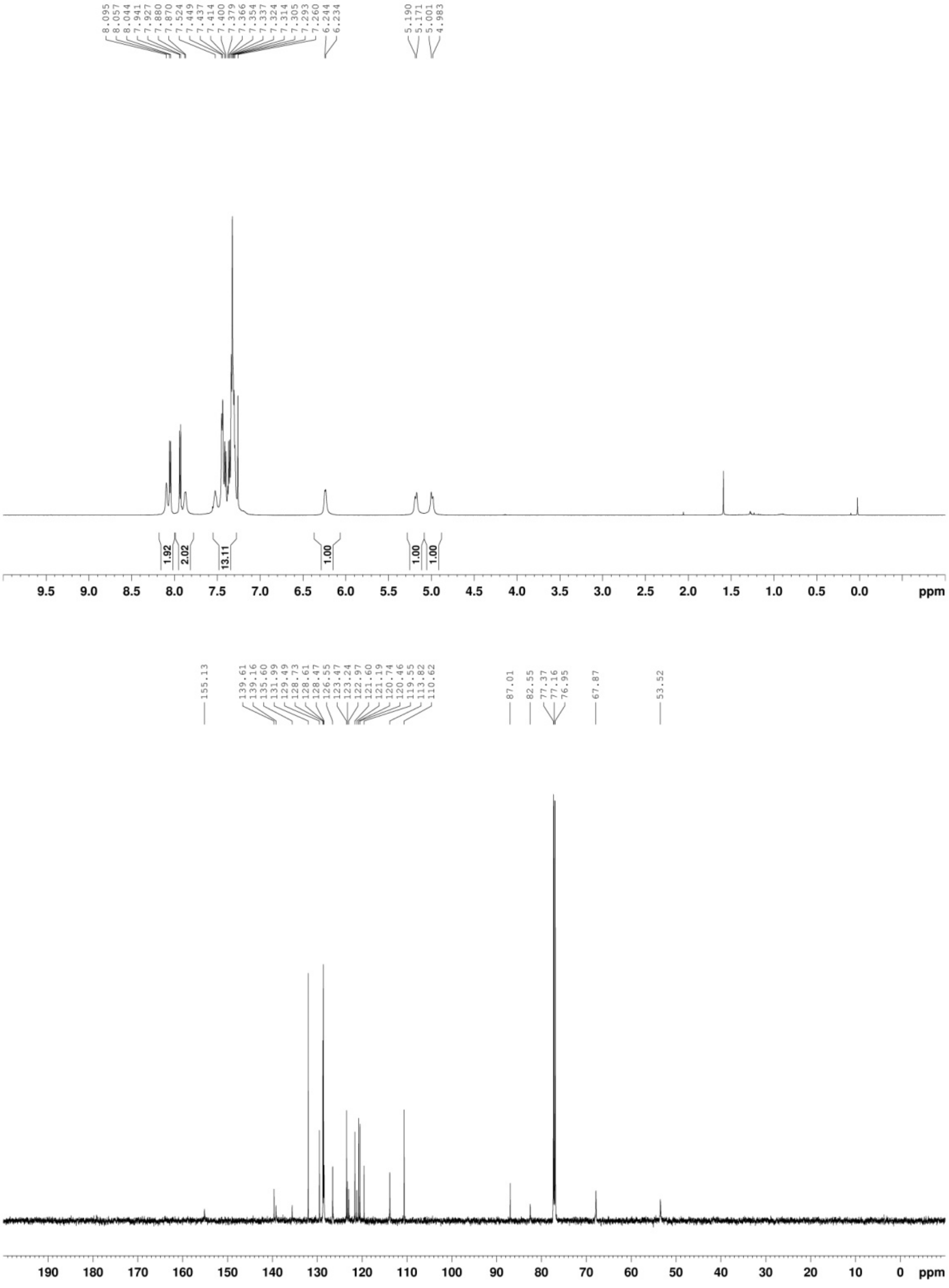

Supplementary Figure 10. ^{1}H and ^{13}C-NMR spectrum for **3e**

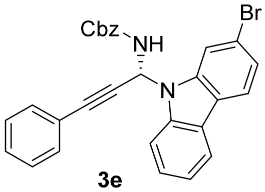

3e

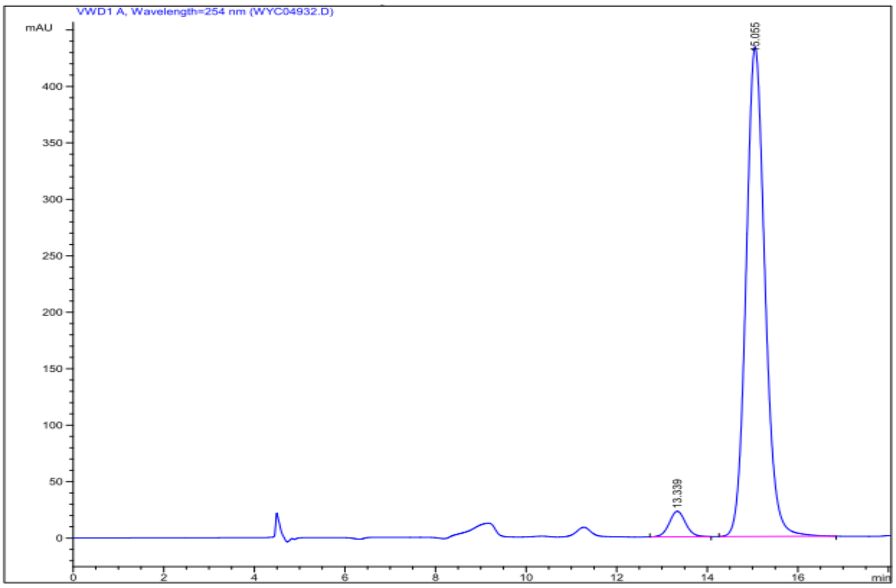

Peak #	RetTime [min]	Type	Width [min]	Area mAU *s	Height [mAU]	Area %
1	13.339	BB	0.3866	570.94977	22.70393	4.3522
2	15.055	BB	0.4439	1.25477e4	433.57007	95.6478

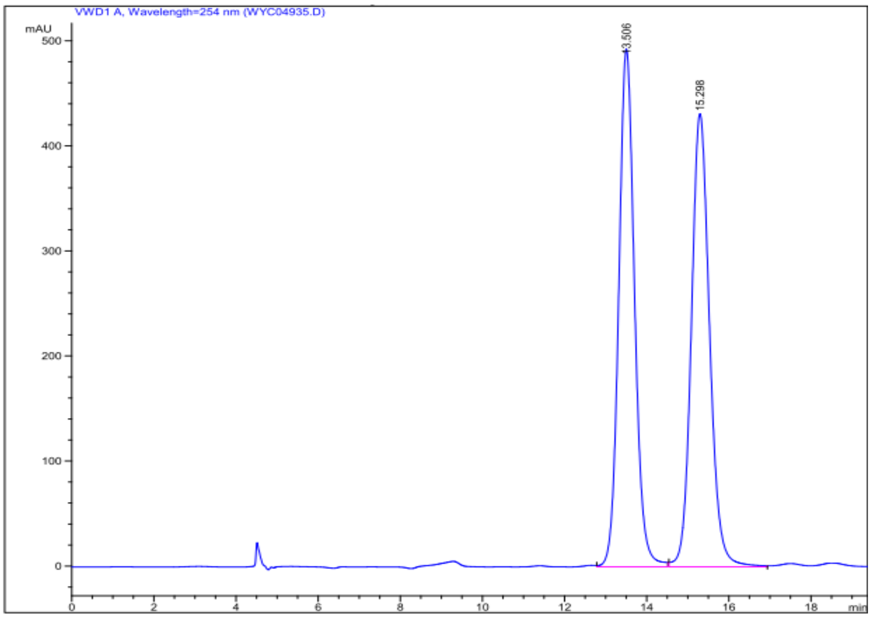

Peak #	RetTime [min]	Type	Width [min]	Area mAU *s	Height [mAU]	Area %
1	13.506	VB	0.4128	1.31938e4	492.88266	49.7634
2	15.298	BB	0.4752	1.33193e4	431.42184	50.2366

Supplementary Figure 11. HPLC spectrum for **3e**

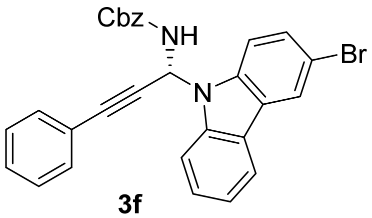

3f

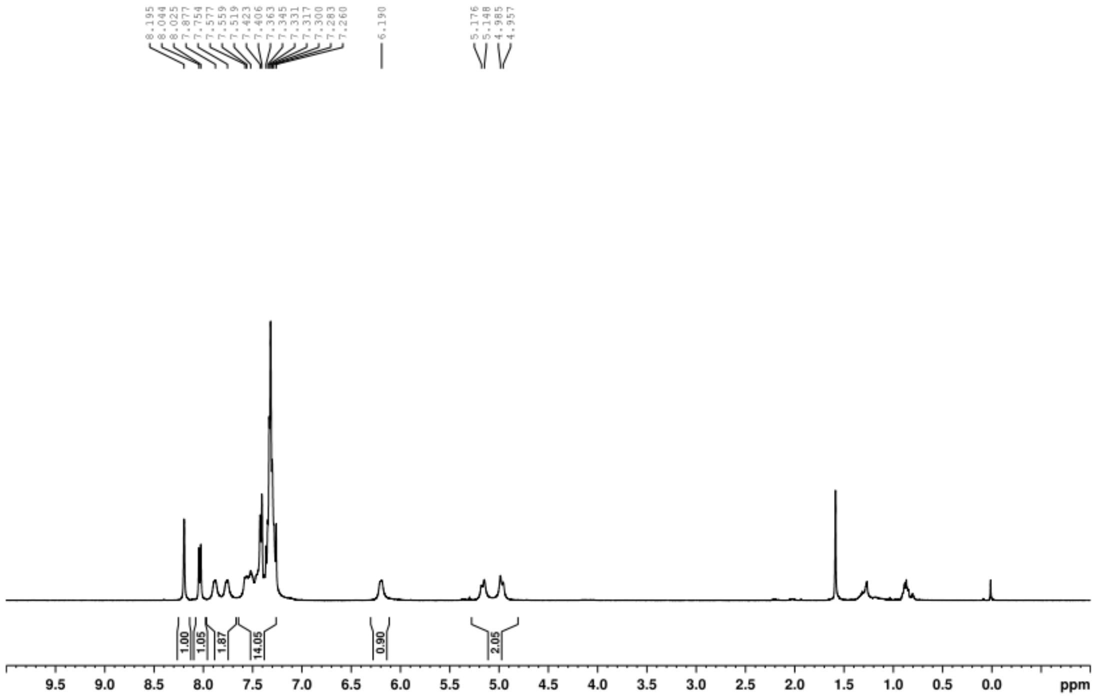

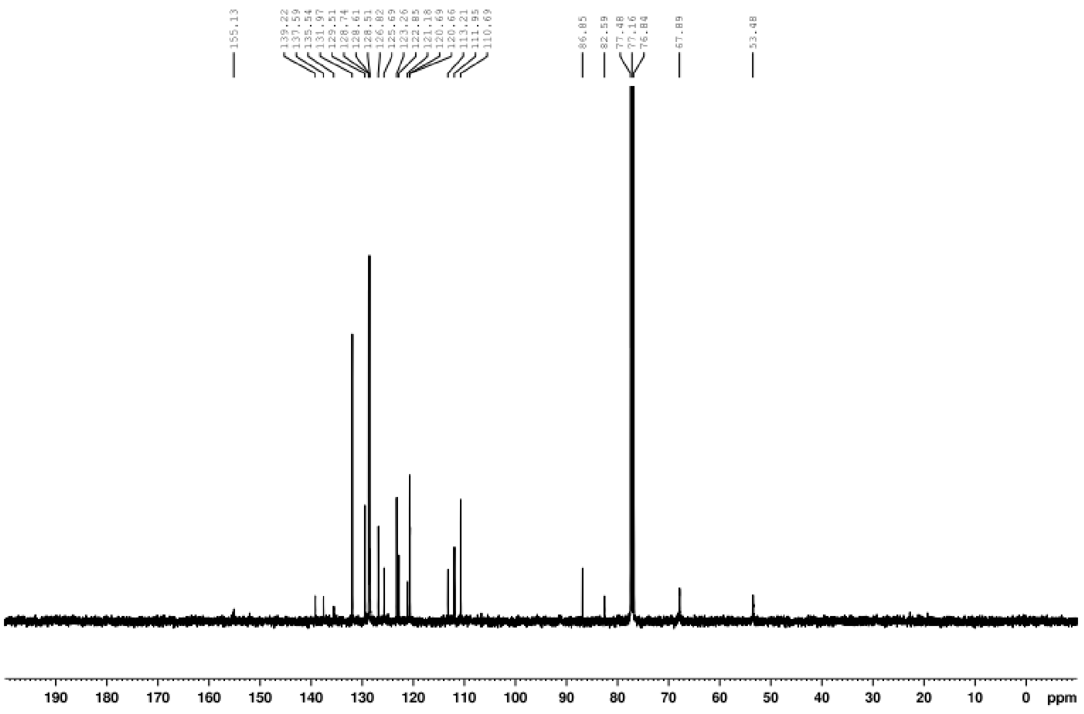

Supplementary Figure 12. ^{1}H and ^{13}C-NMR spectrum for **3f**

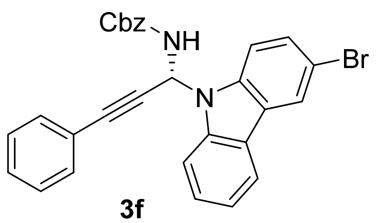

3f

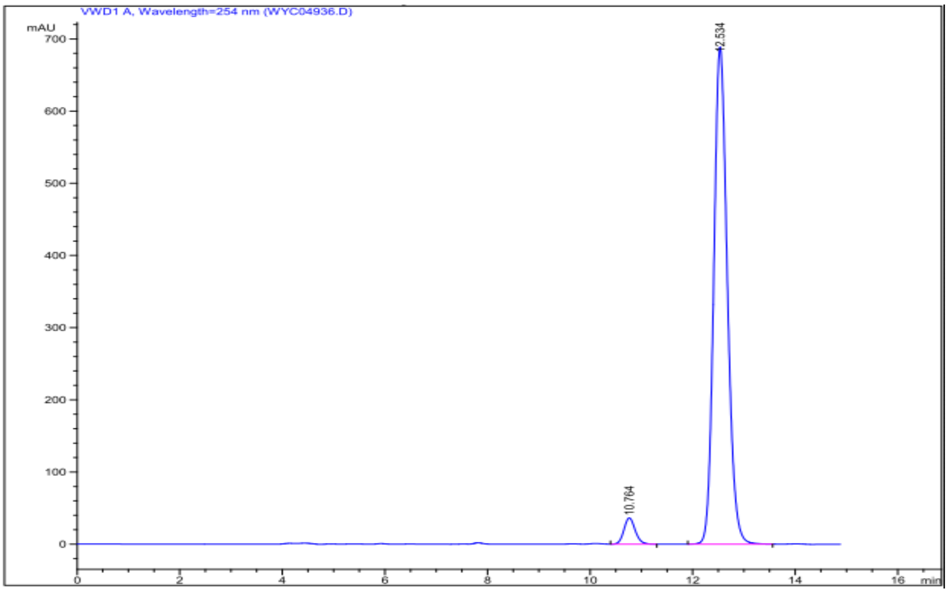

Peak #	RetTime [min]	Type	Width [min]	Area mAU *s	Height [mAU]	Area %
1	10.764	VB	0.2344	553.70331	36.36011	4.2067
2	12.534	BB	0.2820	1.26086e4	688.70874	95.7933

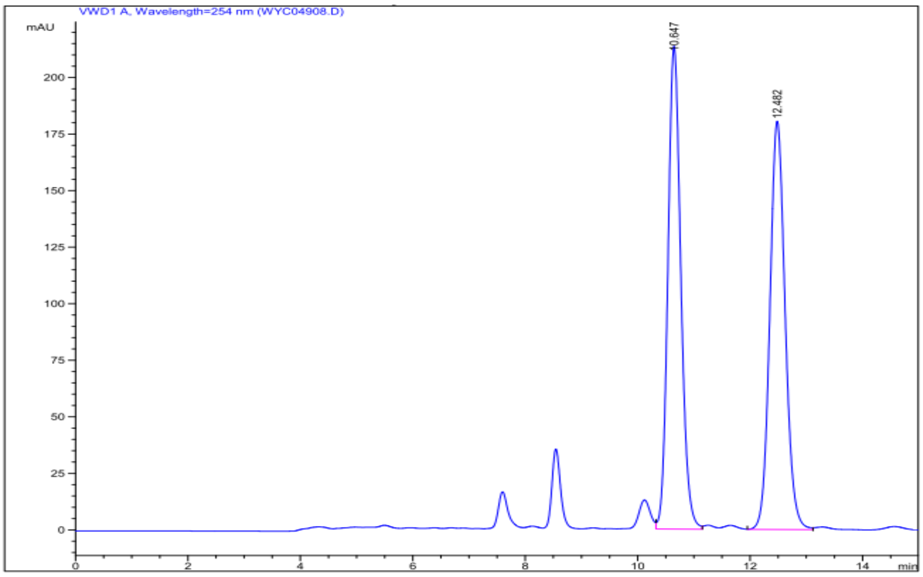

Peak #	RetTime [min]	Type	Width [min]	Area mAU *s	Height [mAU]	Area %
1	10.647	VV	0.2445	3409.63916	213.52443	49.9985
2	12.482	VB	0.2903	3409.84570	180.51567	50.0015

Supplementary Figure 13. HPLC spectrum for **3f**

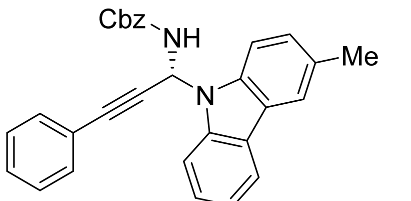

3g

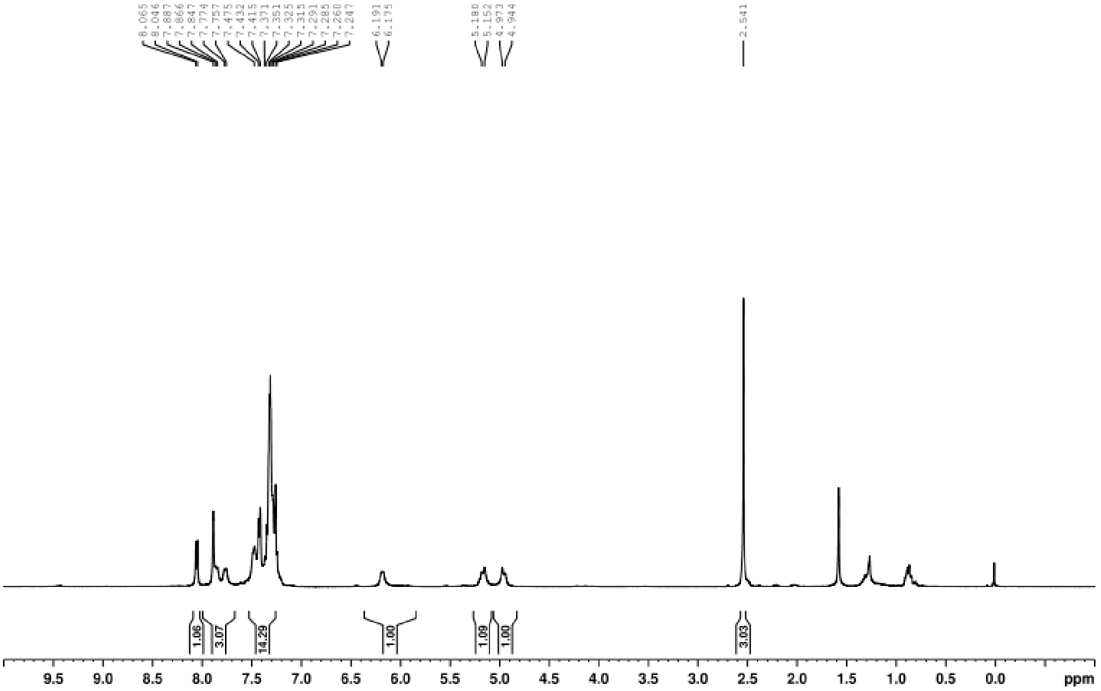

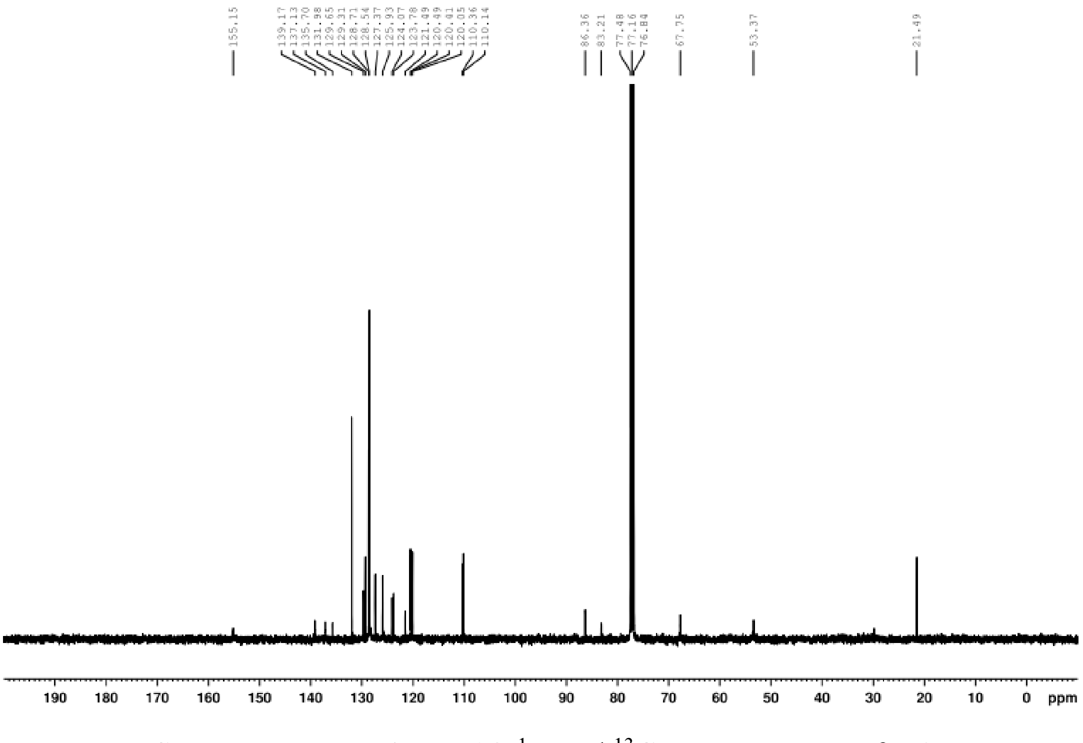

Supplementary Figure 14. ^{1}H and ^{13}C-NMR spectrum for **3g**

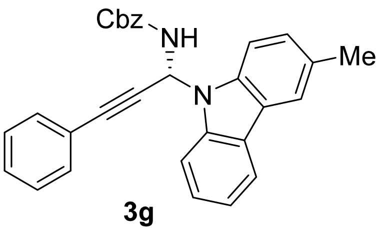

3g

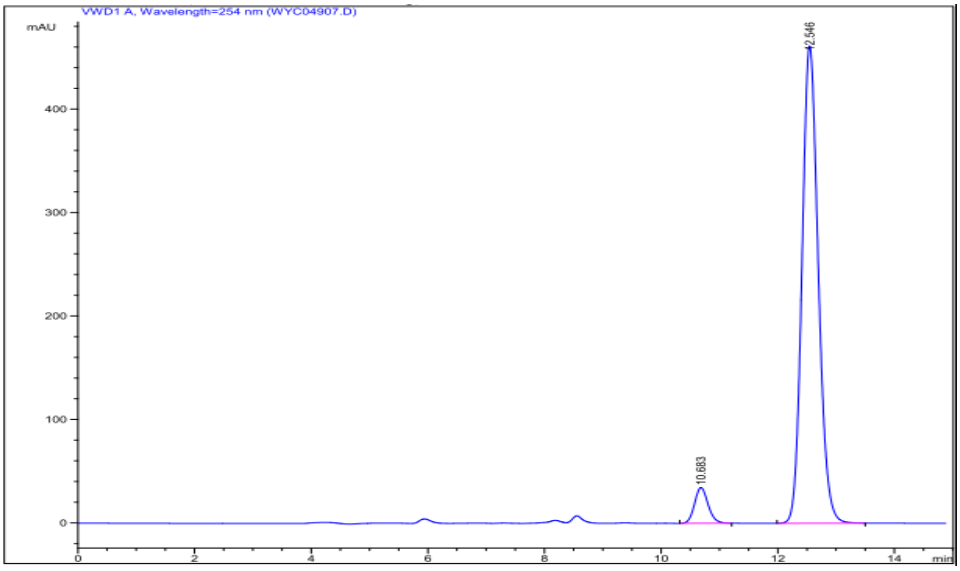

Peak #	RetTime [min]	Type	Width [min]	Area mAU *s	Height [mAU]	Area %
1	10.683	PB	0.2486	554.55902	34.51968	5.8527
2	12.546	BB	0.3013	8920.67578	461.41898	94.1473

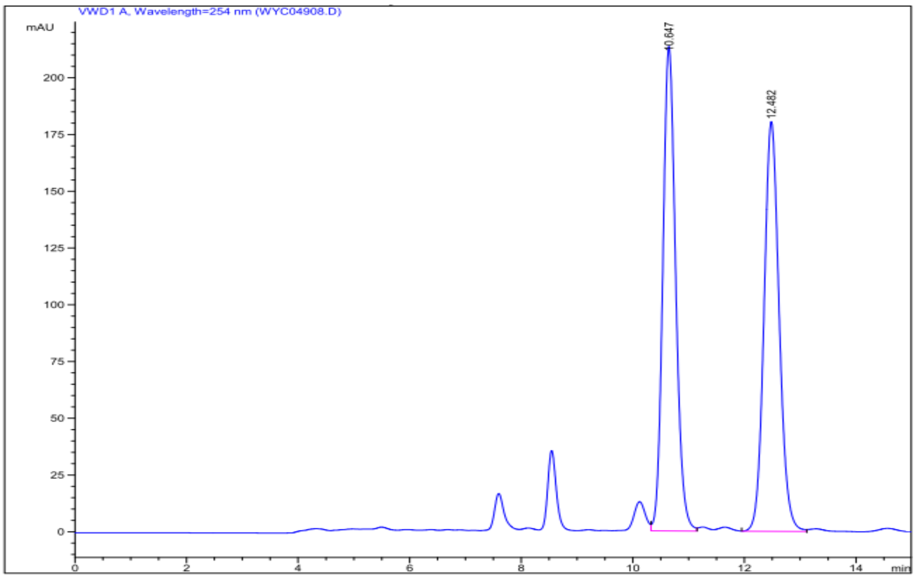

Peak #	RetTime [min]	Type	Width [min]	Area mAU *s	Height [mAU]	Area %
1	10.647	VV	0.2445	3409.63916	213.52443	49.9985
2	12.482	VB	0.2903	3409.84570	180.51567	50.0015

Supplementary Figure 15. HPLC spectrum for **3g**

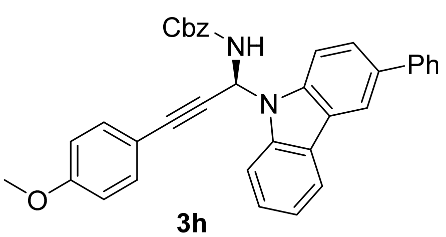

3h

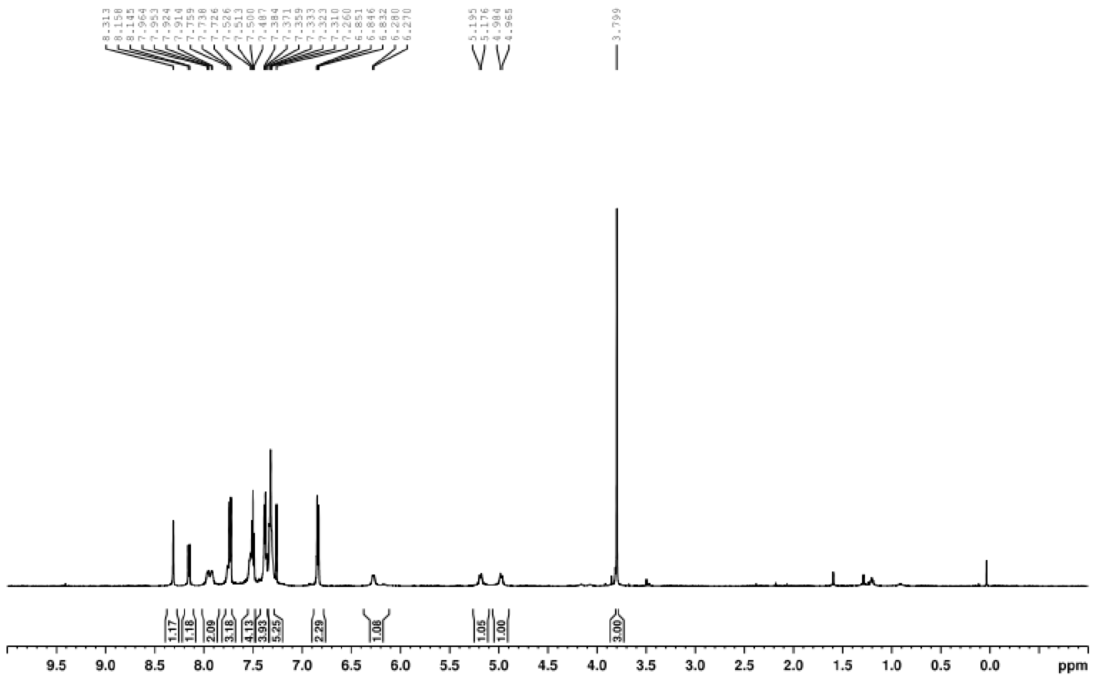

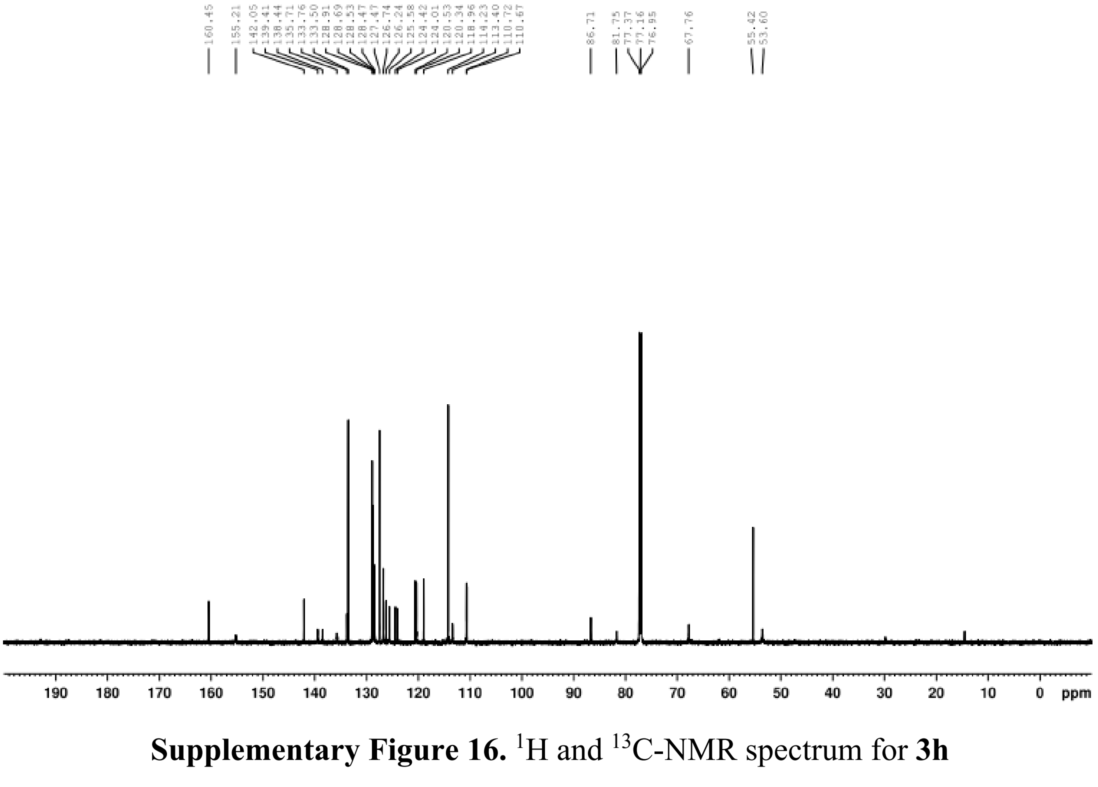

Supplementary Figure 16. ^{1}H and ^{13}C-NMR spectrum for **3h**

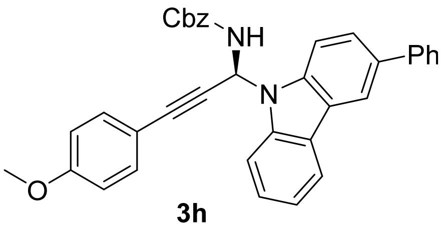

3h

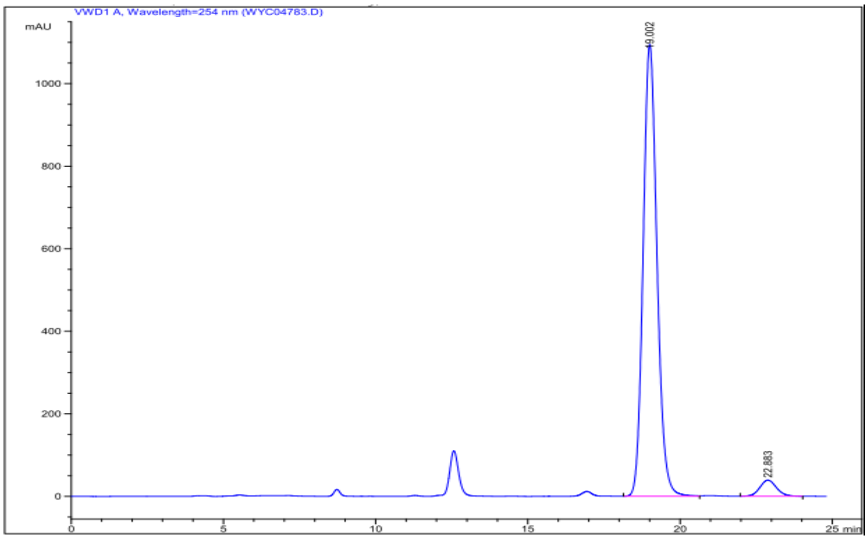

Peak #	RetTime [min]	Type	Width [min]	Area mAU *s	Height [mAU]	Area %
1	19.002	BB	0.4873	3.45353e4	1095.16577	95.8894
2	22.883	BB	0.5842	1480.46167	38.98786	4.1106

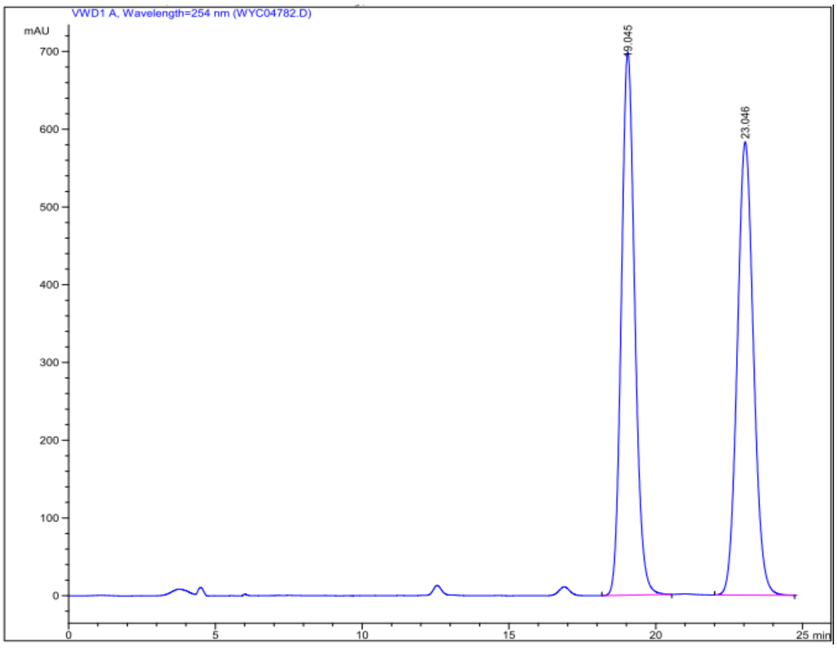

Peak #	RetTime [min]	Type	Width [min]	Area mAU *s	Height [mAU]	Area %
1	19.045	PB	0.4892	2.22353e4	698.64825	49.8305
2	23.046	BB	0.5935	2.23866e4	583.16174	50.1695

Supplementary Figure 17. HPLC spectrum for **3h**

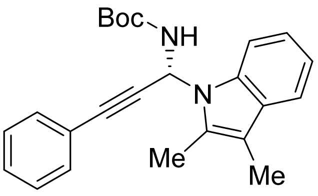

5a

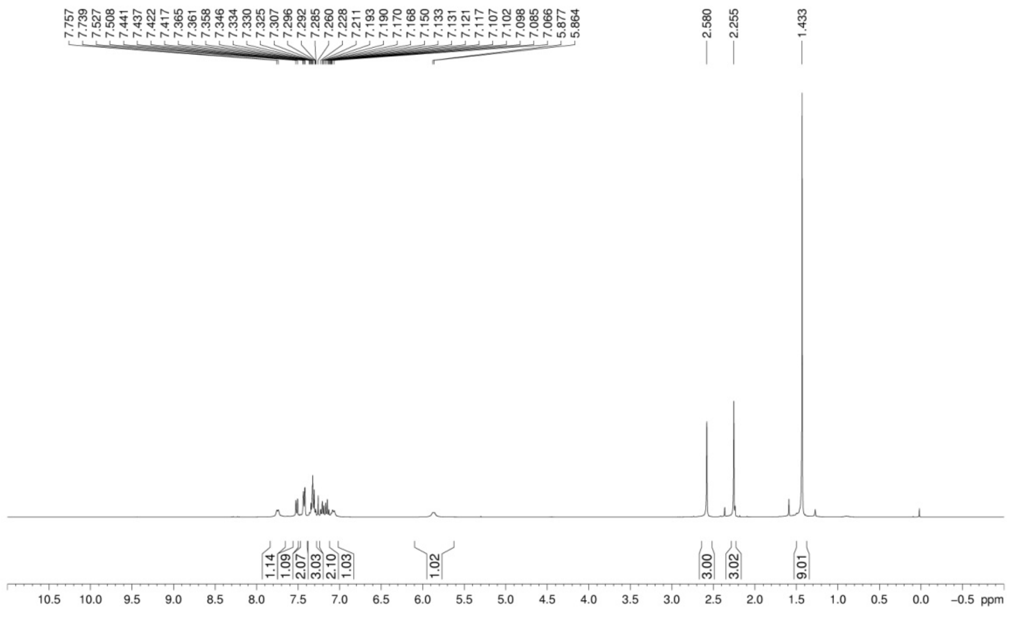

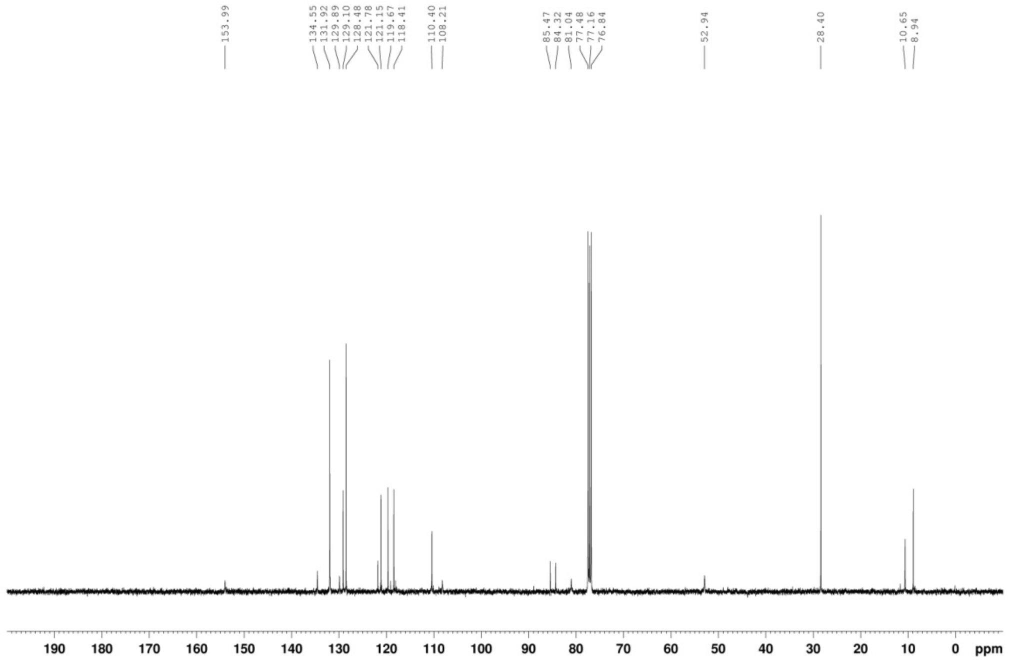

Supplementary Figure 18. ^{1}H and ^{13}C-NMR spectrum for **5a**

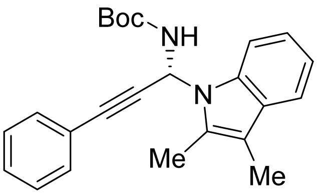

5a

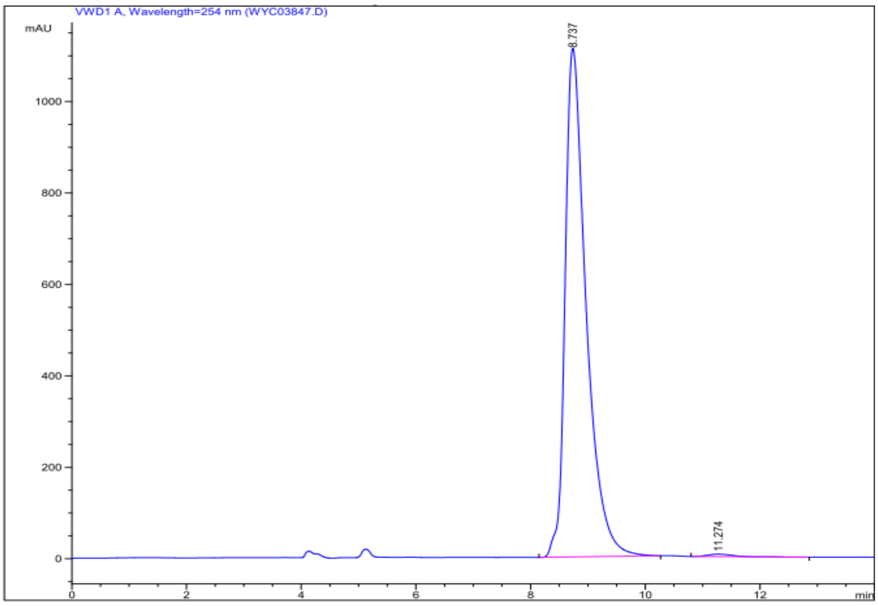

Peak #	RetTime [min]	Type	Width [min]	Area mAU *s	Height [mAU]	Area %
1	8.737	PB	0.3773	2.83532e4	1113.02454	99.4358
2	11.274	PP	0.4418	160.87848	5.42984	0.5642

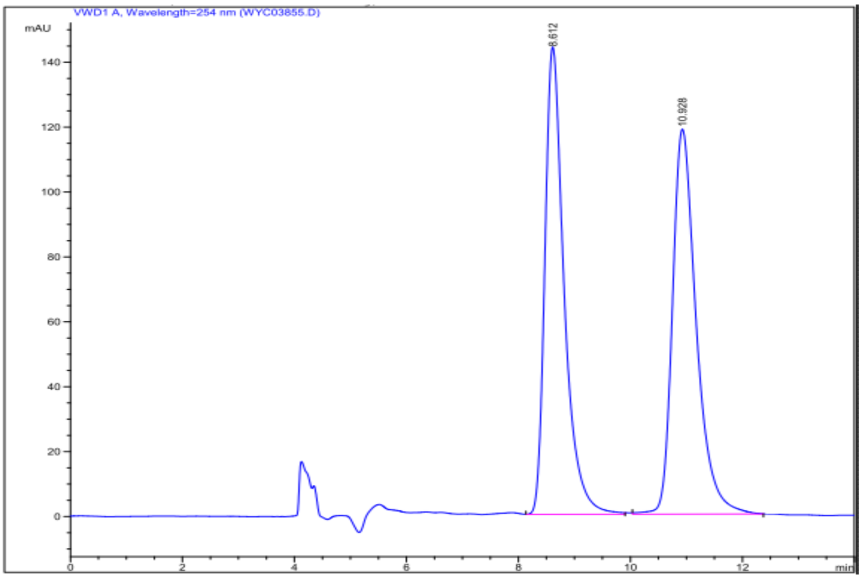

Peak #	RetTime [min]	Type	Width [min]	Area mAU *s	Height [mAU]	Area %
1	8.612	VB	0.3565	3430.20386	144.00903	49.4833
2	10.928	BB	0.4463	3501.83618	118.61591	50.5167

Supplementary Figure 19. HPLC spectrum for **5a**

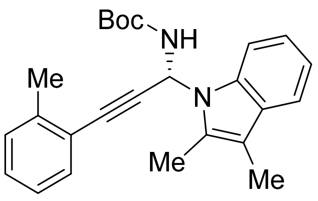

5b

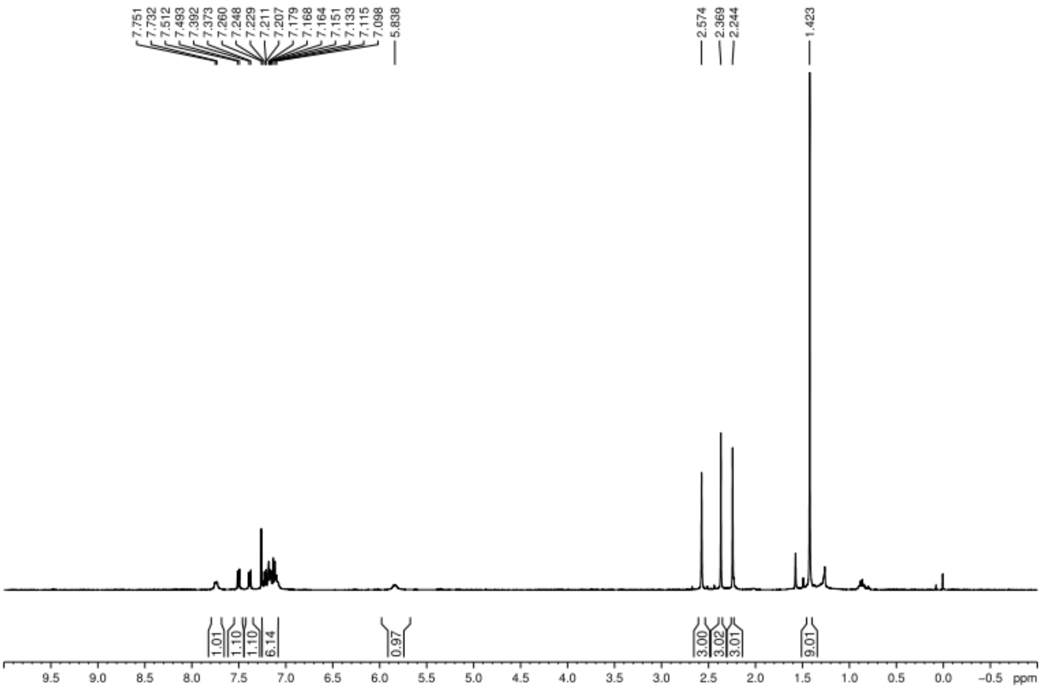

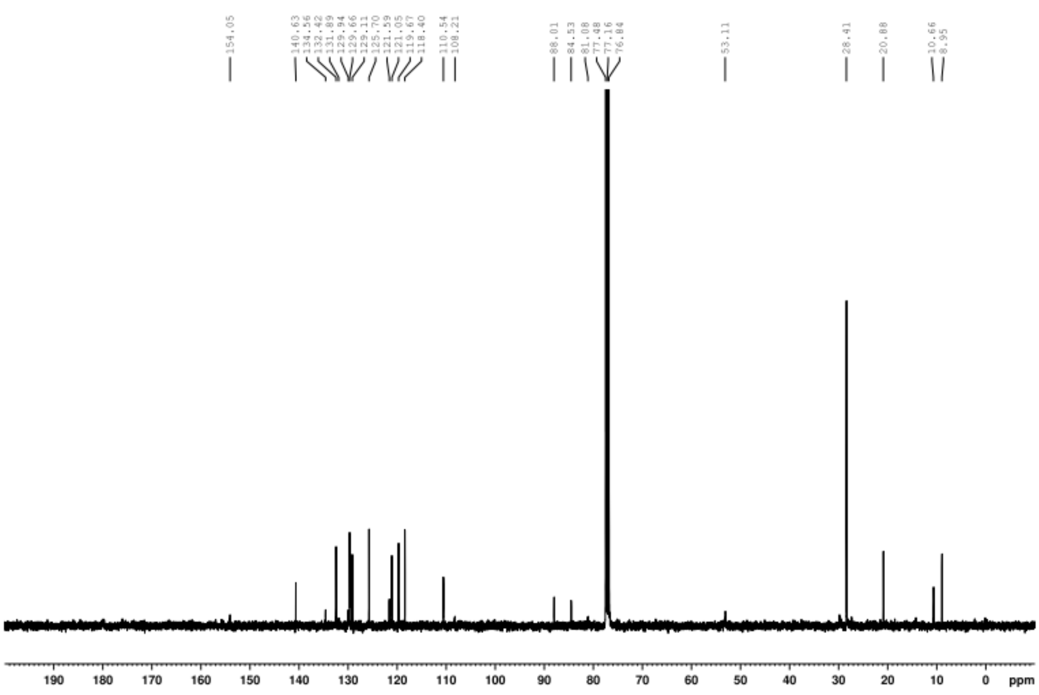

Supplementary Figure 20. ^{1}H and ^{13}C-NMR spectrum for **5b**

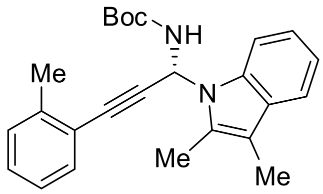

5b

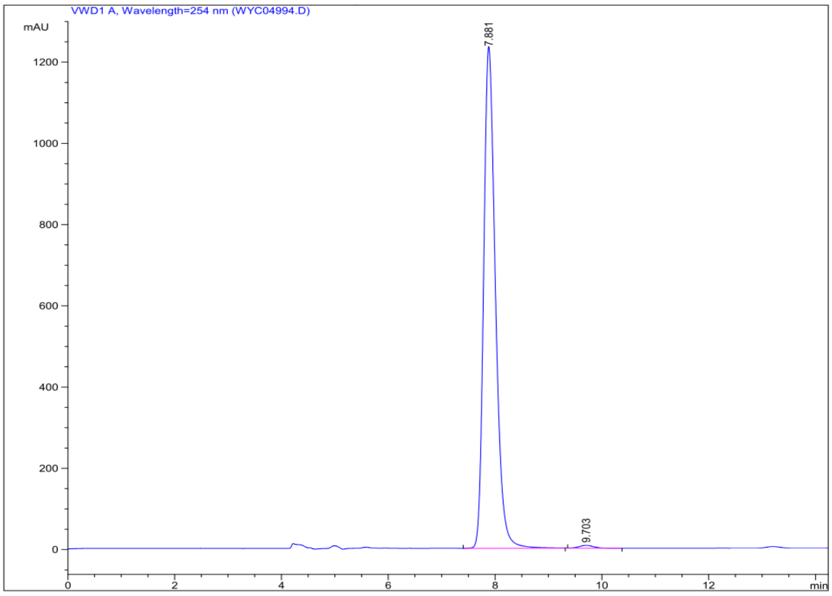

Peak #	RetTime [min]	Type	Width [min]	Area mAU *s	Height [mAU]	Area %
1	7.881	PB	0.2371	1.89325e4	1235.00598	99.2715
2	9.703	BB	0.2987	138.94125	7.13013	0.7285

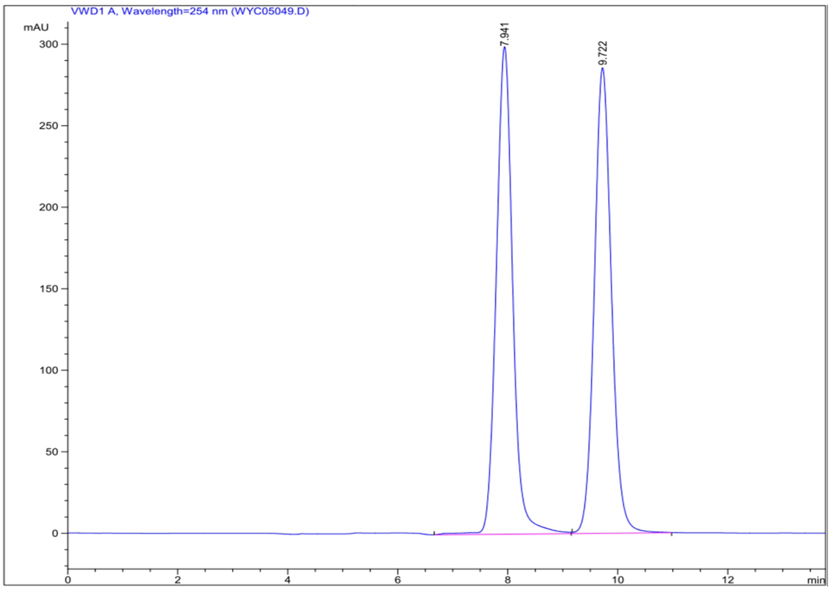

Peak #	RetTime [min]	Type	Width [min]	Area mAU *s	Height [mAU]	Area %
1	7.941	PB	0.3185	6222.39209	298.96881	50.2242
2	9.722	BB	0.3321	6166.83057	285.47186	49.7758

Supplementary Figure 21. HPLC spectrum for **5b**

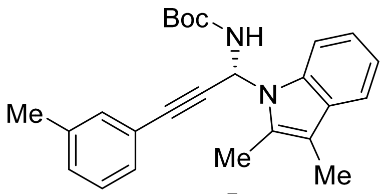

5c

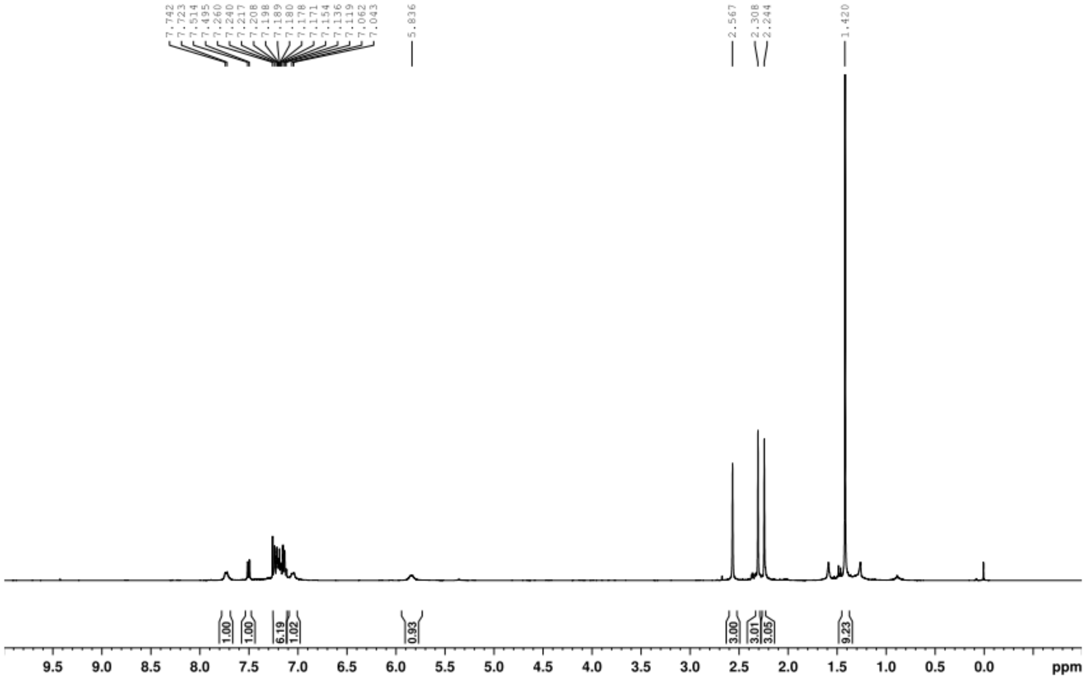

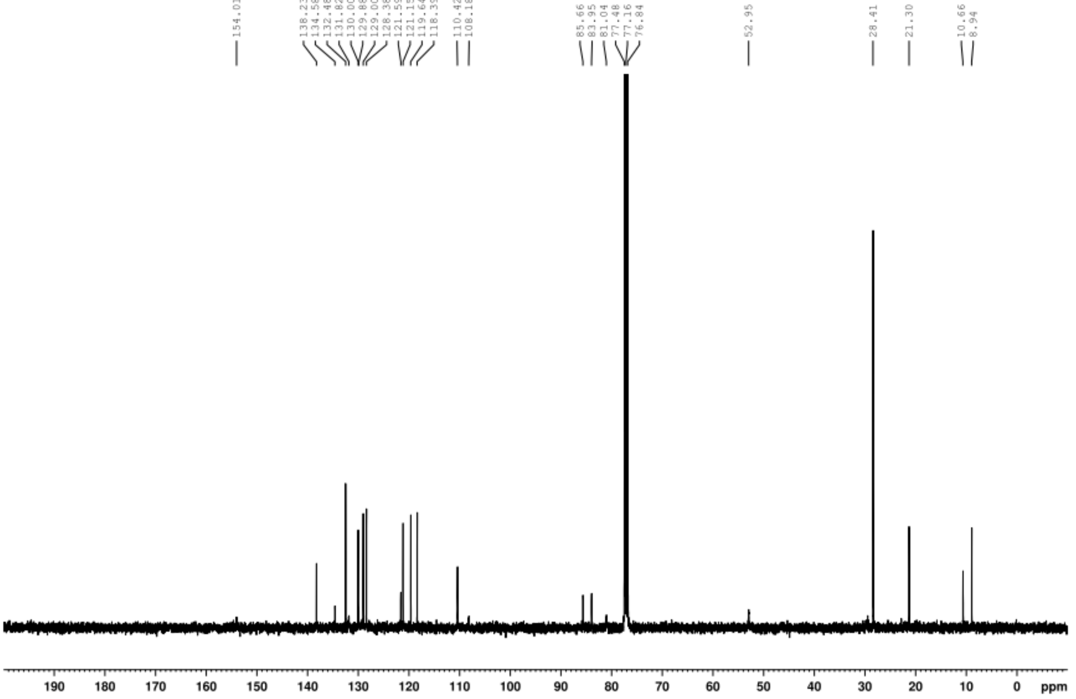

Supplementary Figure 22. ^{1}H and ^{13}C-NMR spectrum for **5c**

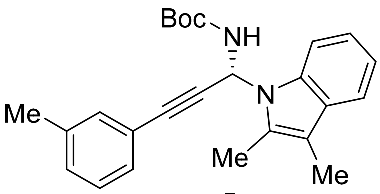

5c

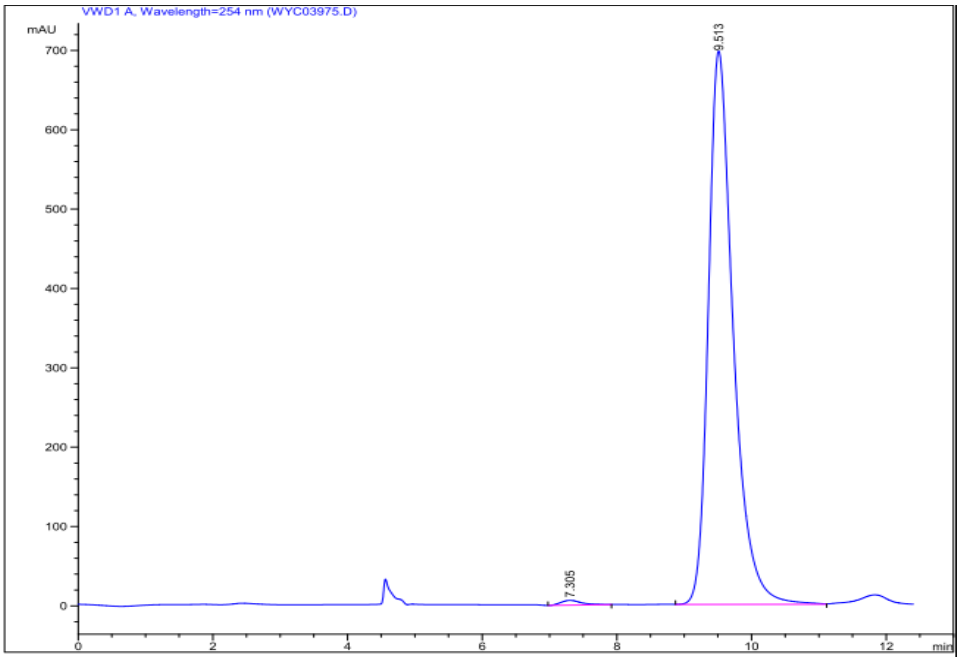

Peak #	RetTime [min]	Type	Width [min]	Area mAU *s	Height [mAU]	Area %
1	7.305	VB	0.3406	132.65895	6.07588	0.7340
2	9.513	VV	0.3832	1.79405e4	697.25262	99.2660

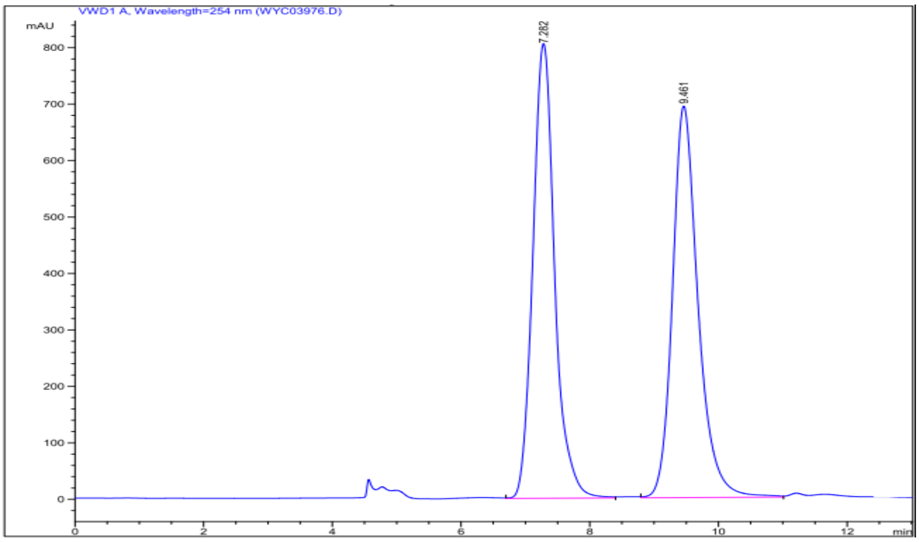

Peak #	RetTime [min]	Type	Width [min]	Area mAU *s	Height [mAU]	Area %
1	7.282	VB	0.3604	1.89483e4	805.43066	49.7128
2	9.461	BV	0.4156	1.91672e4	693.54791	50.2872

Supplementary Figure 23. HPLC spectrum for **5c**

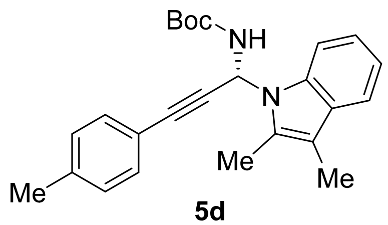

5d

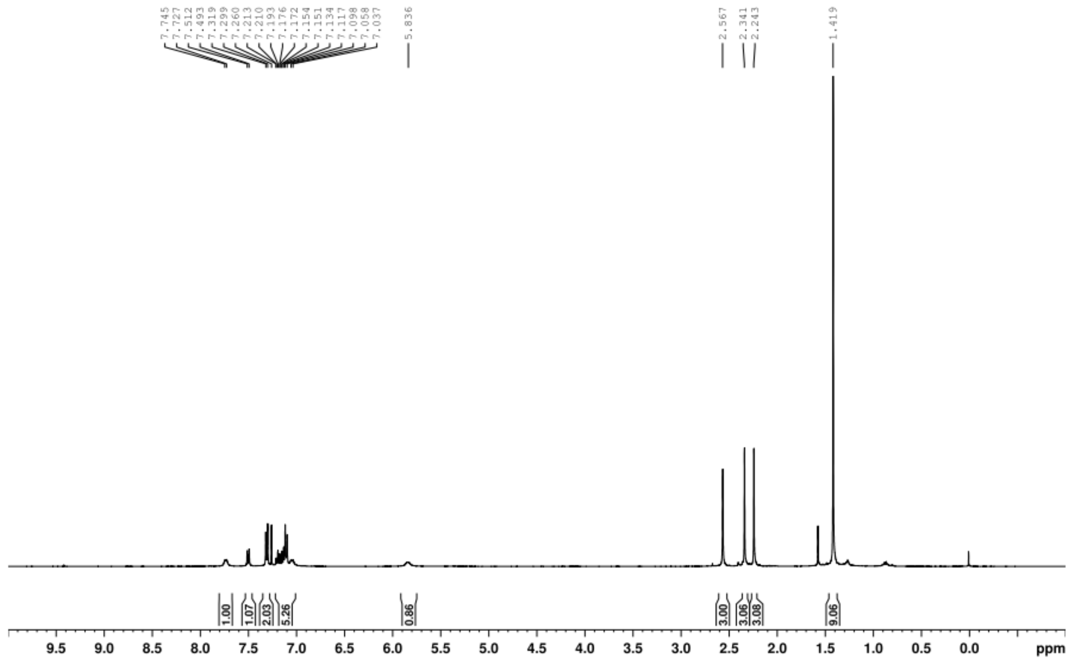

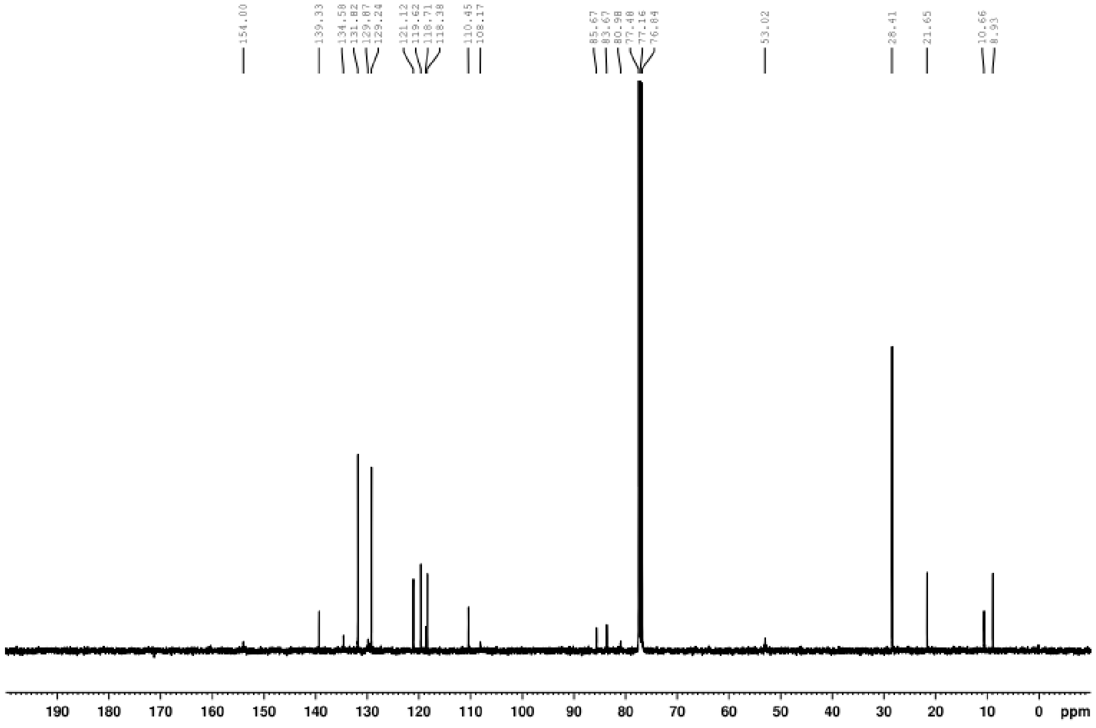

Supplementary Figure 24. ^{1}H and ^{13}C-NMR spectrum for **5d**

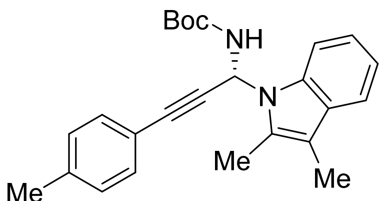

5d

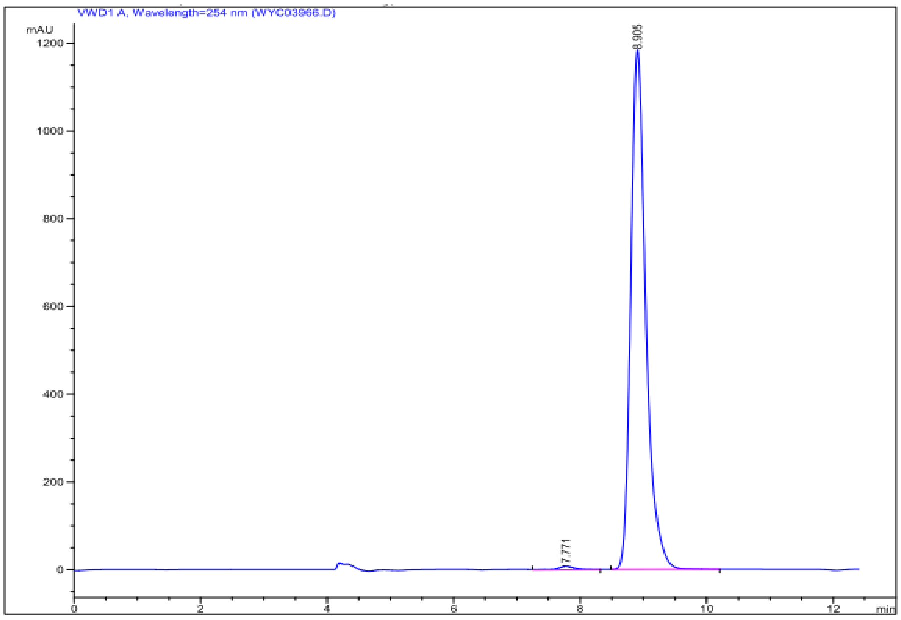

Peak #	RetTime [min]	Type	Width [min]	Area mAU *s	Height [mAU]	Area %
1	7.771	VB	0.2762	161.37204	8.35829	0.8174
2	8.905	PB	0.2512	1.95805e4	1183.72119	99.1826

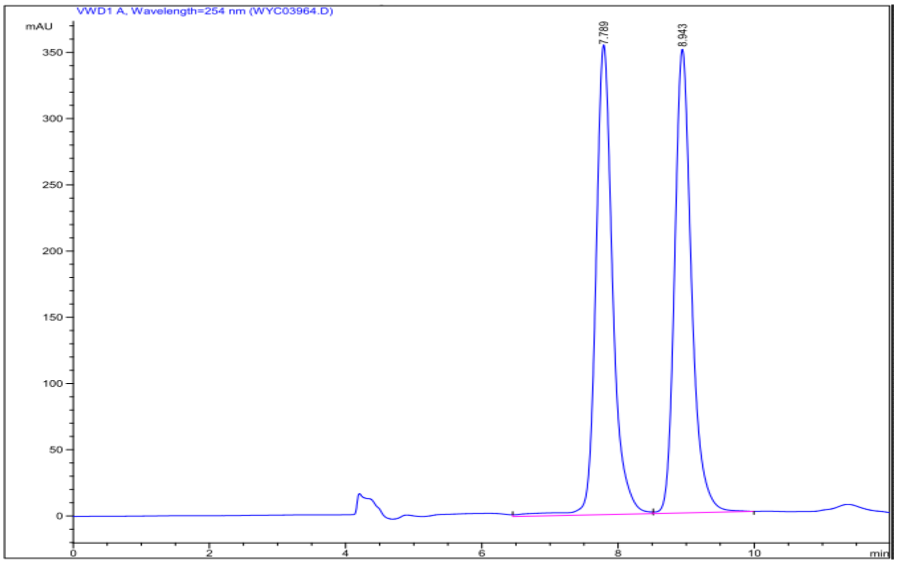

Peak #	RetTime [min]	Type	Width [min]	Area mAU *s	Height [mAU]	Area %
1	7.789	VV	0.2605	6104.24902	354.63028	50.2925
2	8.943	VB	0.2623	6033.23779	350.00809	49.7075

Supplementary Figure 25. HPLC spectrum for **5d**

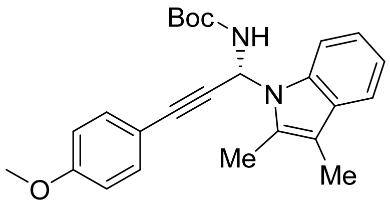

5e

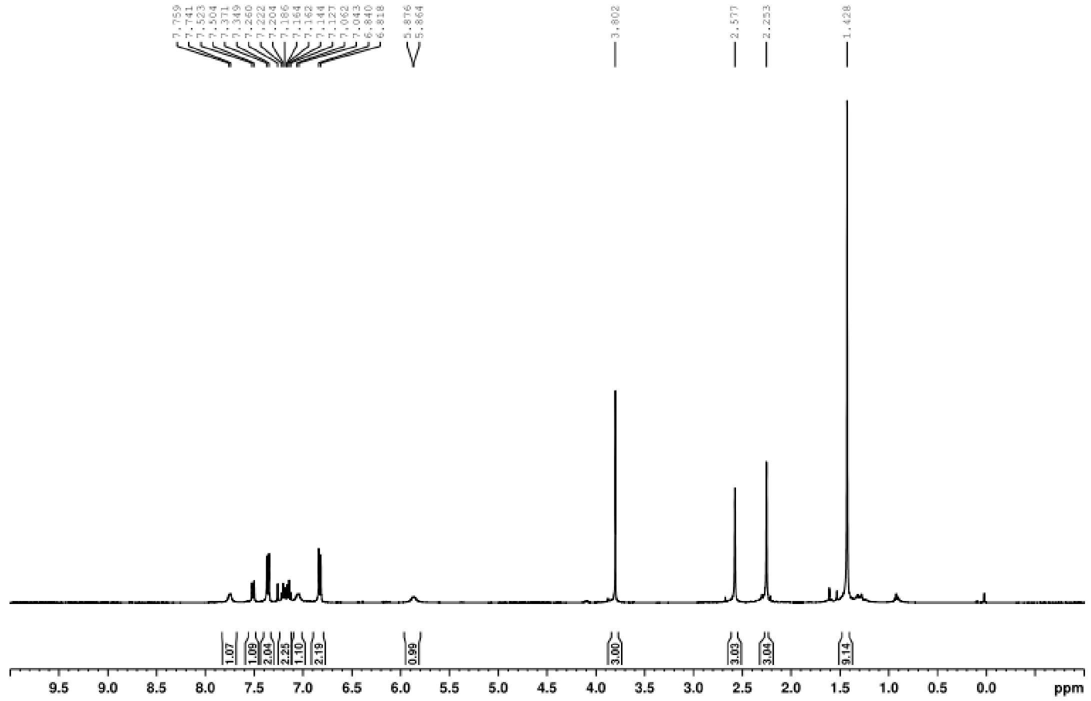

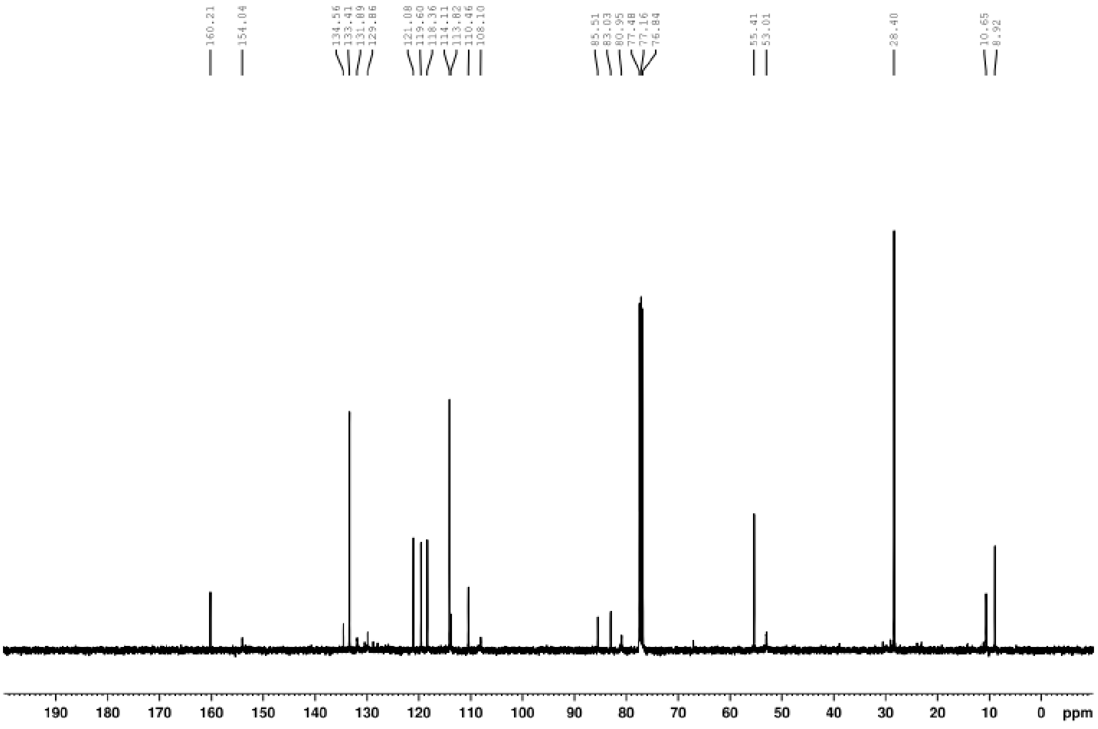

Supplementary Figure 26. ^{1}H and ^{13}C-NMR spectrum for **5e**

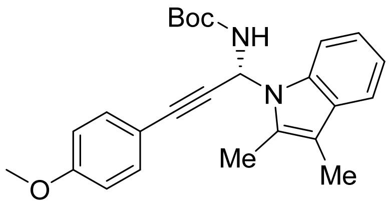

5e

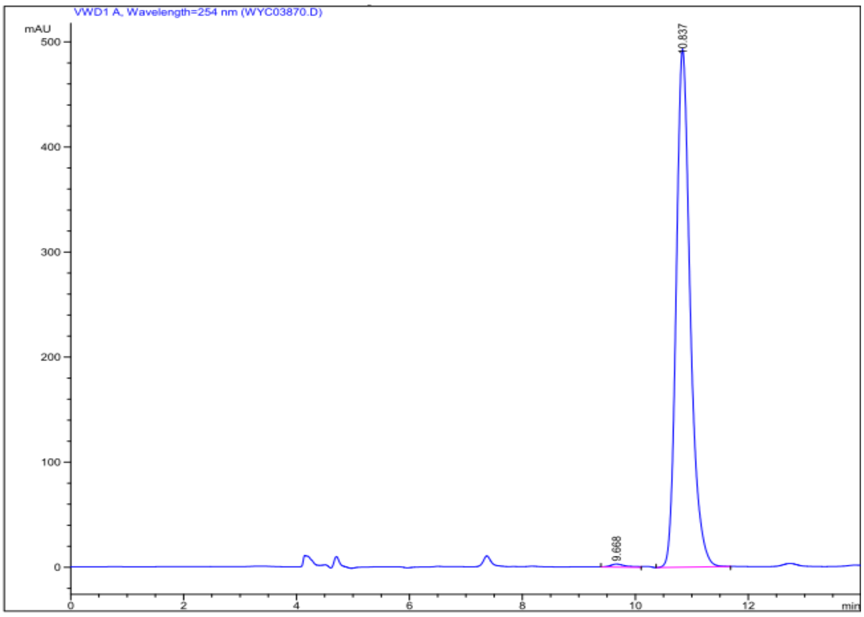

Peak #	RetTime [min]	Type	Width [min]	Area mAU *s	Height [mAU]	Area %
1	9.668	BB	0.2822	56.51989	2.86977	0.6581
2	10.837	VB	0.2630	8532.10840	493.05954	99.3419

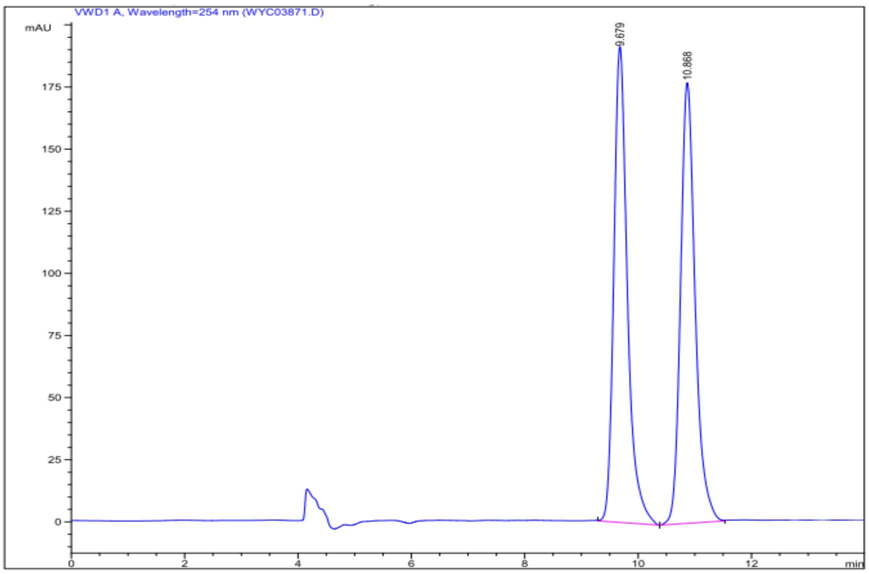

Peak #	RetTime [min]	Type	Width [min]	Area mAU *s	Height [mAU]	Area %
1	9.679	BP	0.2520	3180.84937	191.48039	49.9856
2	10.868	VB	0.2720	3182.68164	177.31310	50.0144

Supplementary Figure 27. HPLC spectrum for **5e**

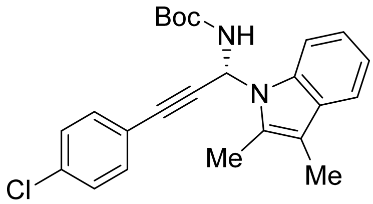

5f

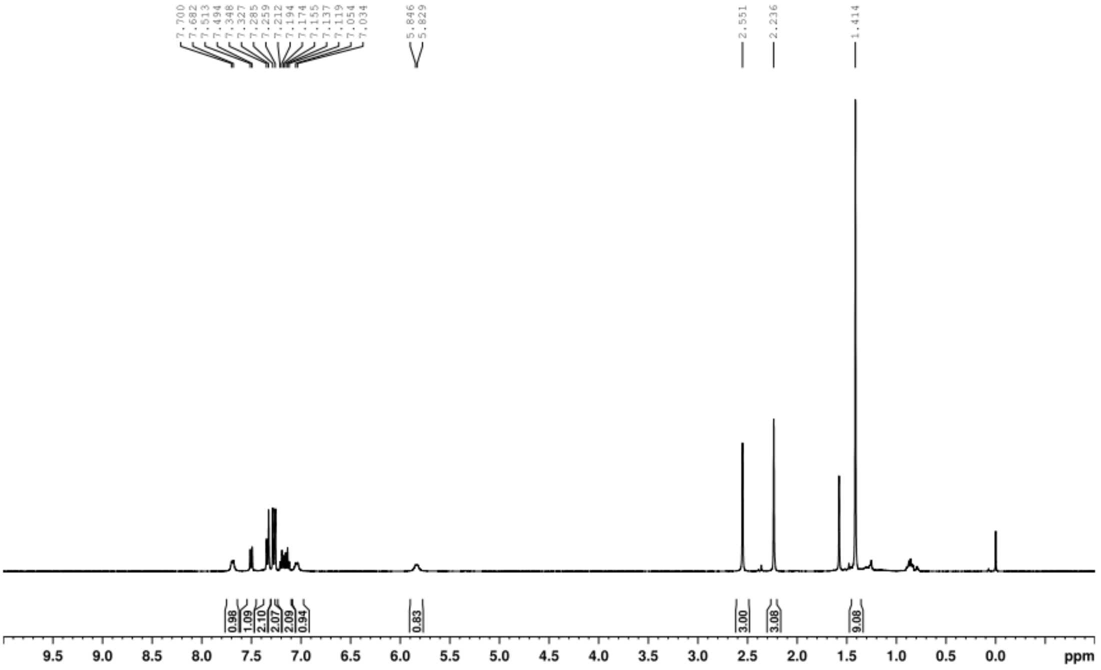

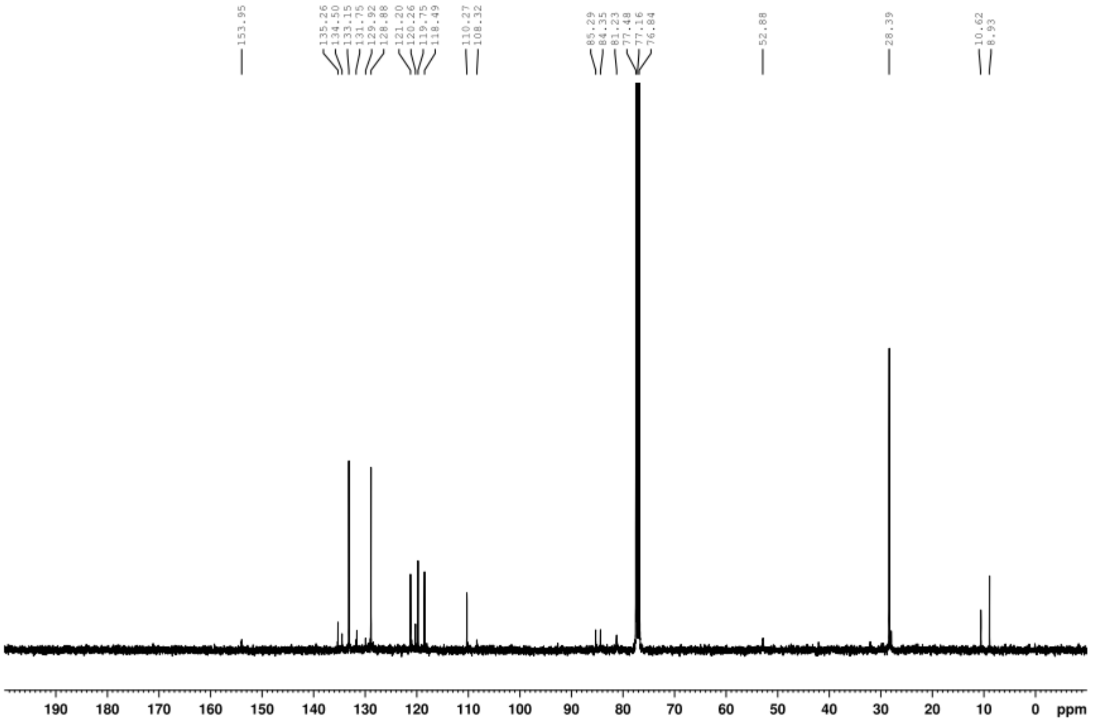

Supplementary Figure 28. ^{1}H and ^{13}C-NMR spectrum for **5f**

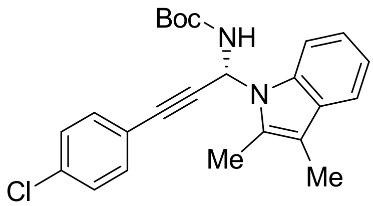

5f

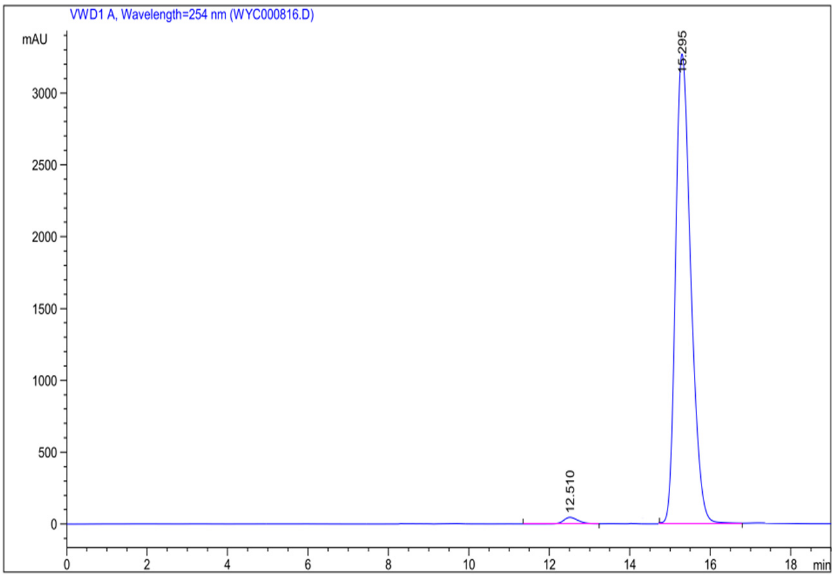

Peak #	RetTime [min]	Type	Width [min]	Area mAU *s	Height [mAU]	Area %
1	12.510	VV	0.3835	1123.64221	45.15251	1.2833
2	15.295	VV	0.4074	8.64338e4	3269.90625	98.7167

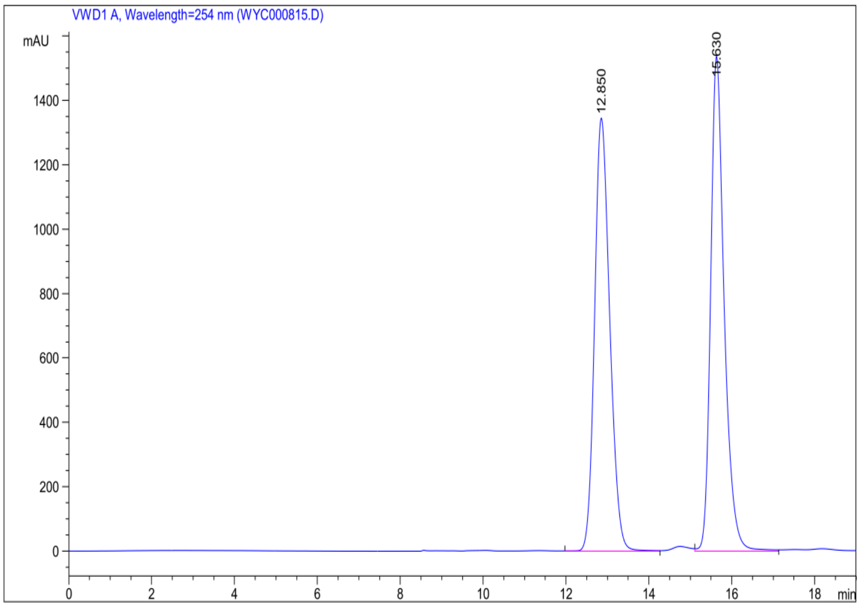

Peak #	RetTime [min]	Type	Width [min]	Area mAU *s	Height [mAU]	Area %
1	12.850	VV	0.3899	3.41284e4	1342.02222	49.4639
2	15.630	VV	0.3469	3.48681e4	1533.13000	50.5361

Supplementary Figure 29. HPLC spectrum for **5f**

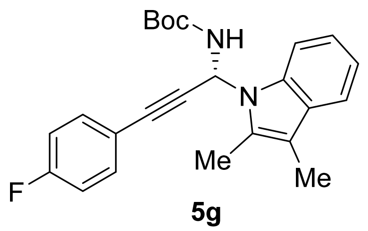

5g

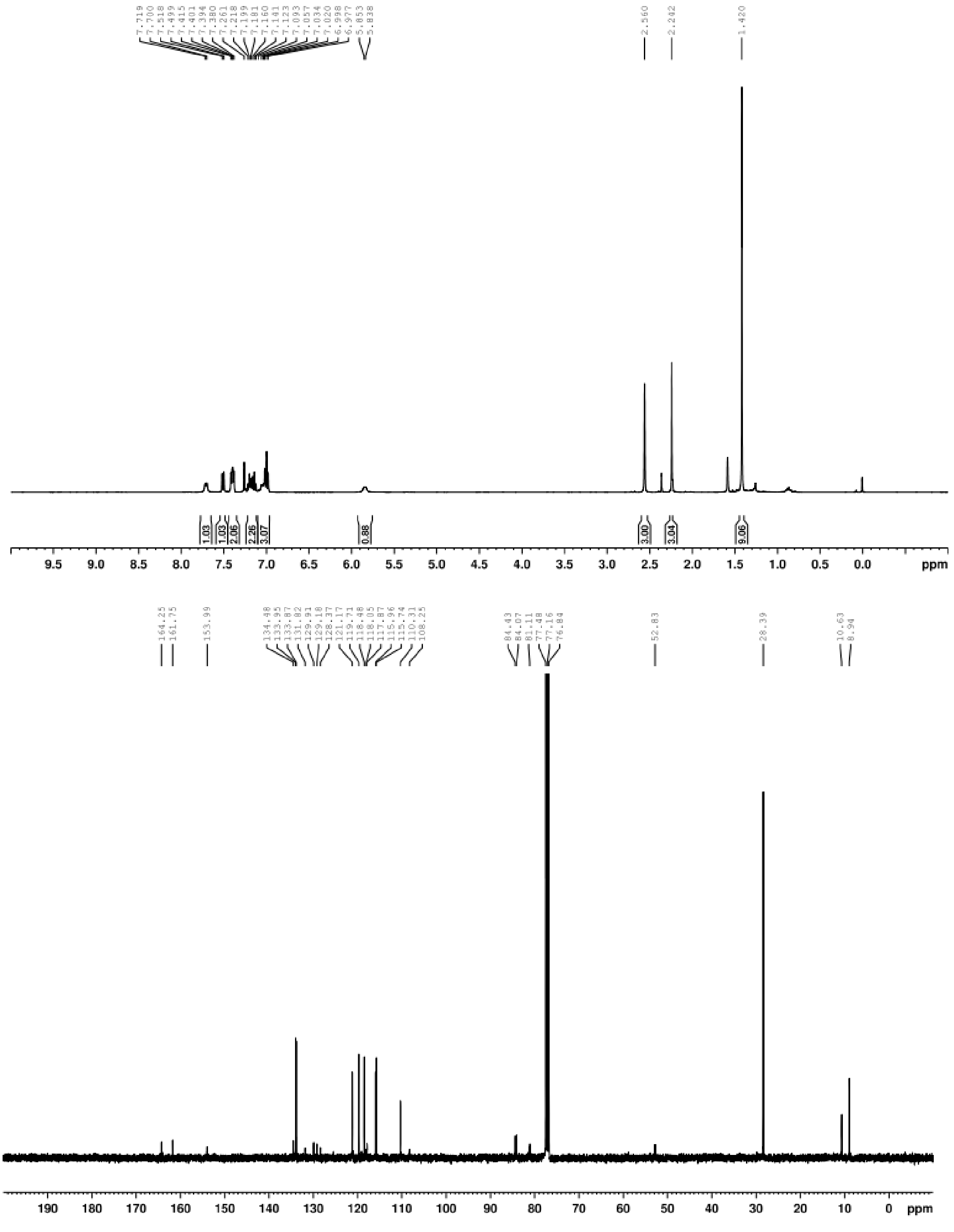

Supplementary Figure 30. ^{1}H and ^{13}C-NMR spectrum for **5g**

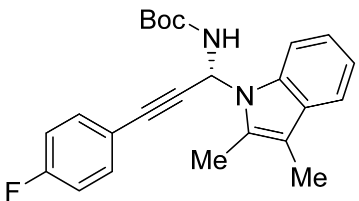

5g

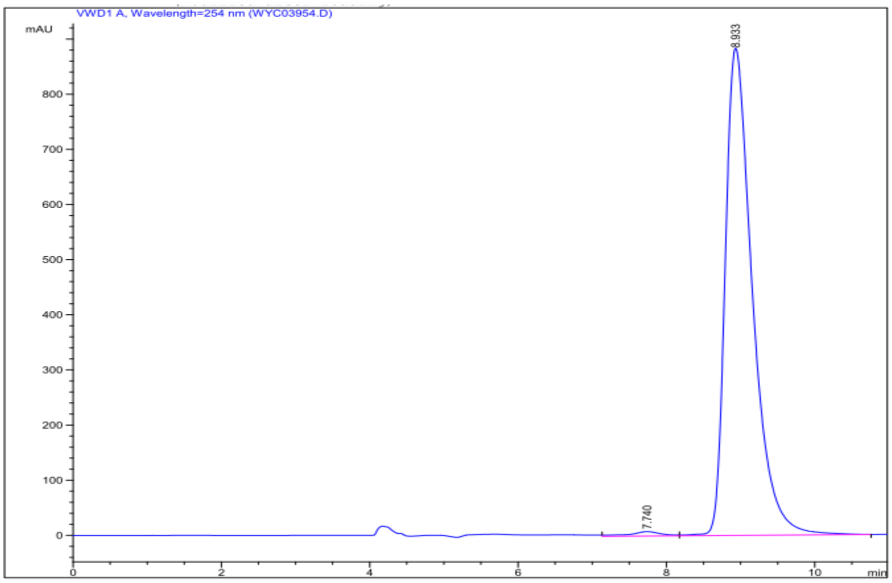

Peak #	RetTime [min]	Type	Width [min]	Area mAU *s	Height [mAU]	Area %
1	7.740	VV	0.4210	240.47897	7.99031	1.0591
2	8.933	VB	0.3855	2.24655e4	883.53369	98.9409

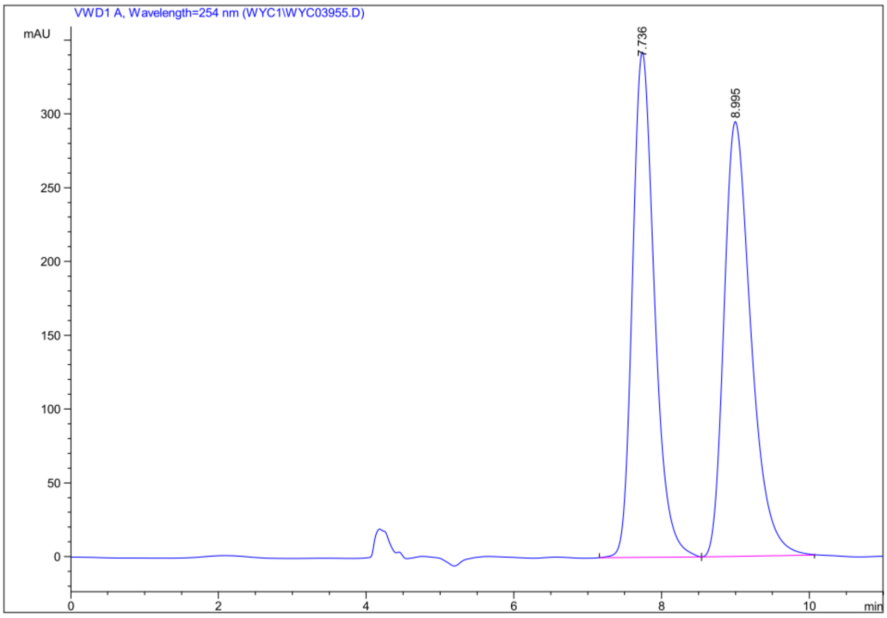

Peak #	RetTime [min]	Type	Width [min]	Area mAU *s	Height [mAU]	Area %
1	7.736	BB	0.3189	7095.56494	342.42569	48.6178
2	8.995	BB	0.3887	7499.01367	294.58240	51.3822

Supplementary Figure 31. HPLC spectrum for **5g**

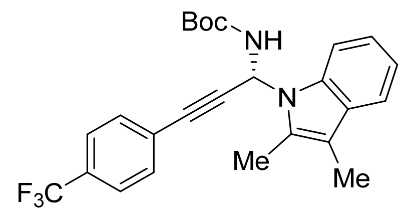

5h

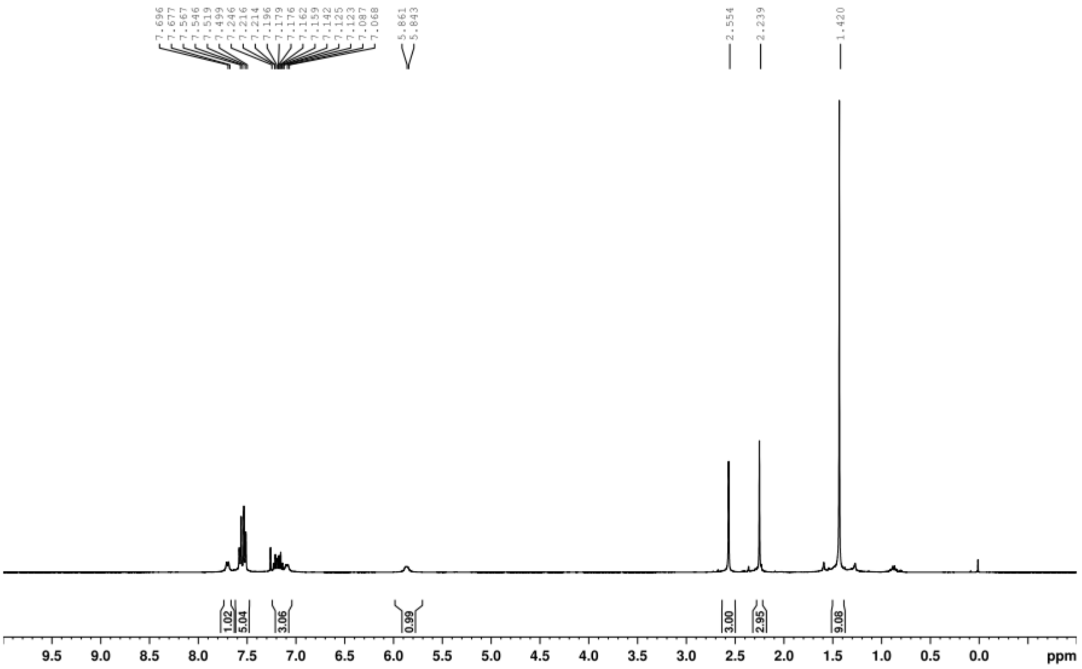

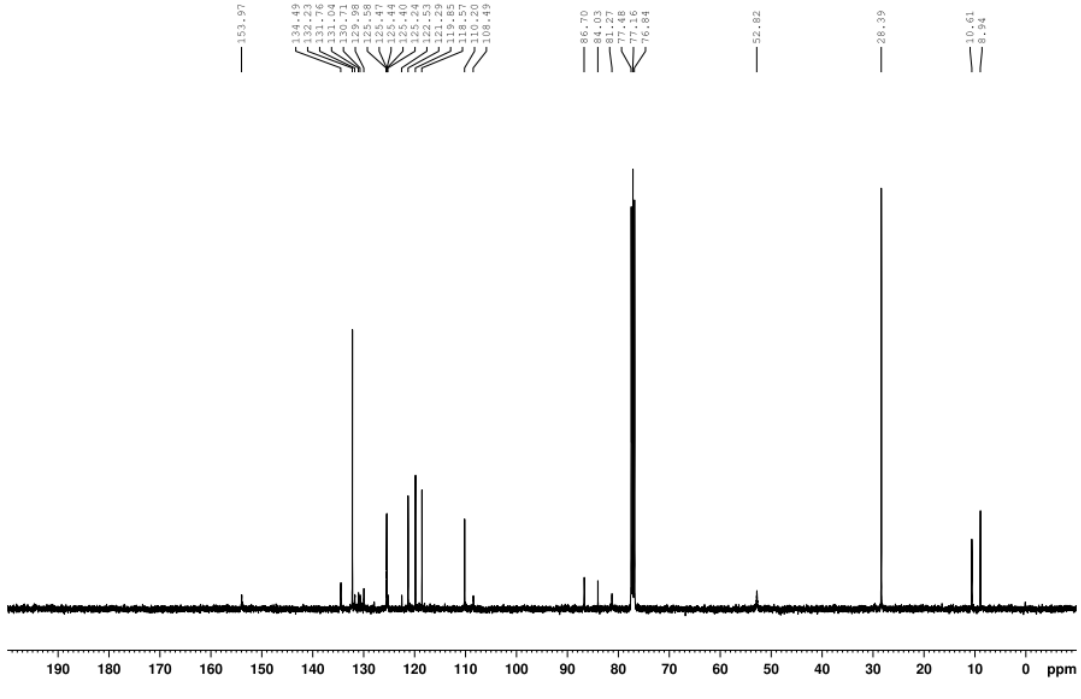

Supplementary Figure 32. ^{1}H and ^{13}C-NMR spectrum for **5h**

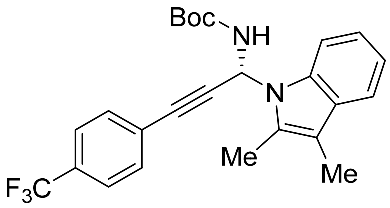

5h

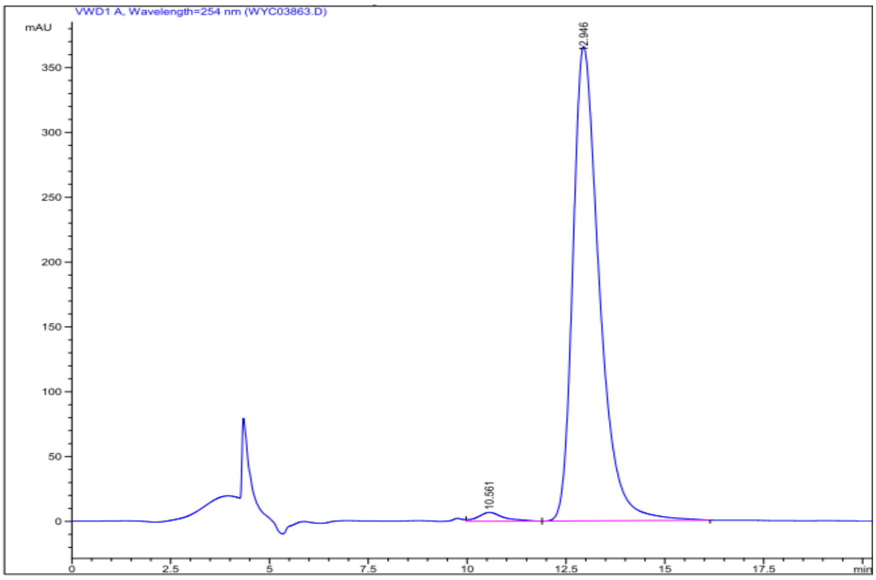

Peak #	RetTime [min]	Type	Width [min]	Area mAU *s	Height [mAU]	Area %
1	10.561	VP	0.6405	300.00931	6.83067	1.7311
2	12.946	VB	0.6987	1.70305e4	365.94638	98.2689

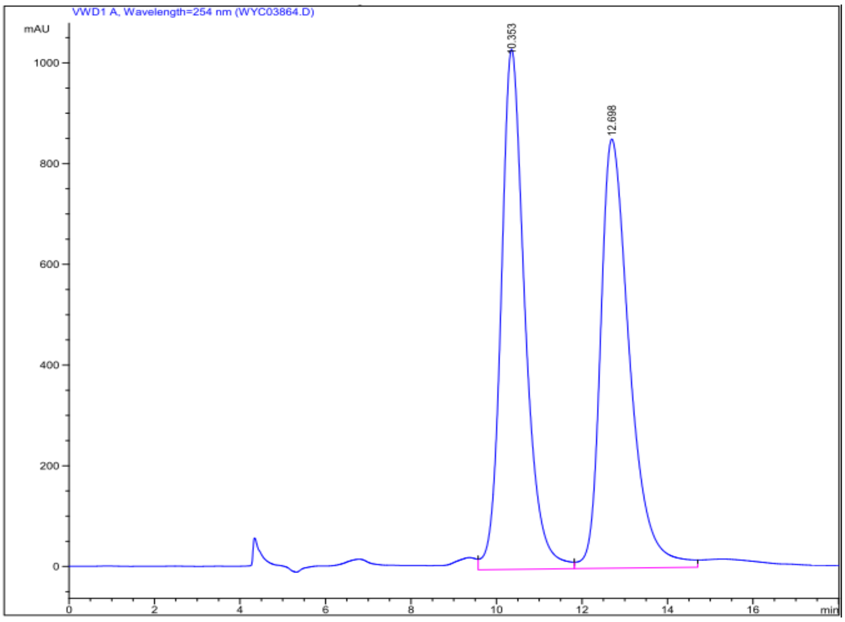

Peak #	RetTime [min]	Type	Width [min]	Area mAU *s	Height [mAU]	Area %
1	10.353	VV	0.5828	3.99953e4	1032.87122	49.8466
2	12.698	VB	0.7169	4.02414e4	852.12817	50.1534

Supplementary Figure 33. HPLC spectrum for **5h**

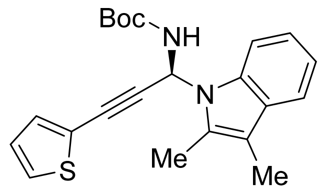

5i

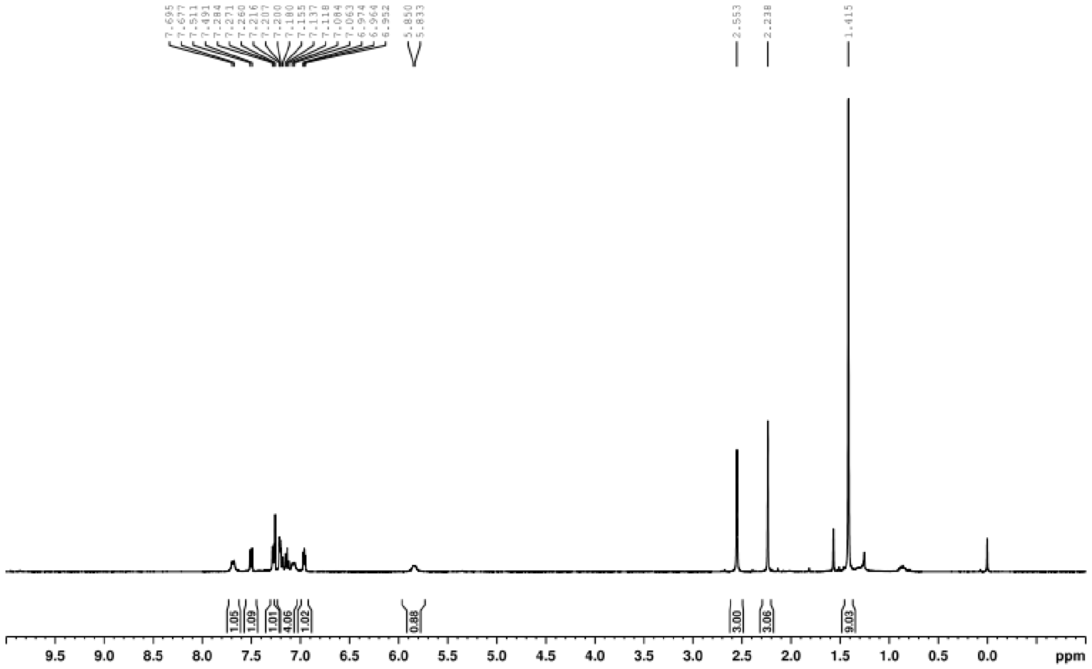

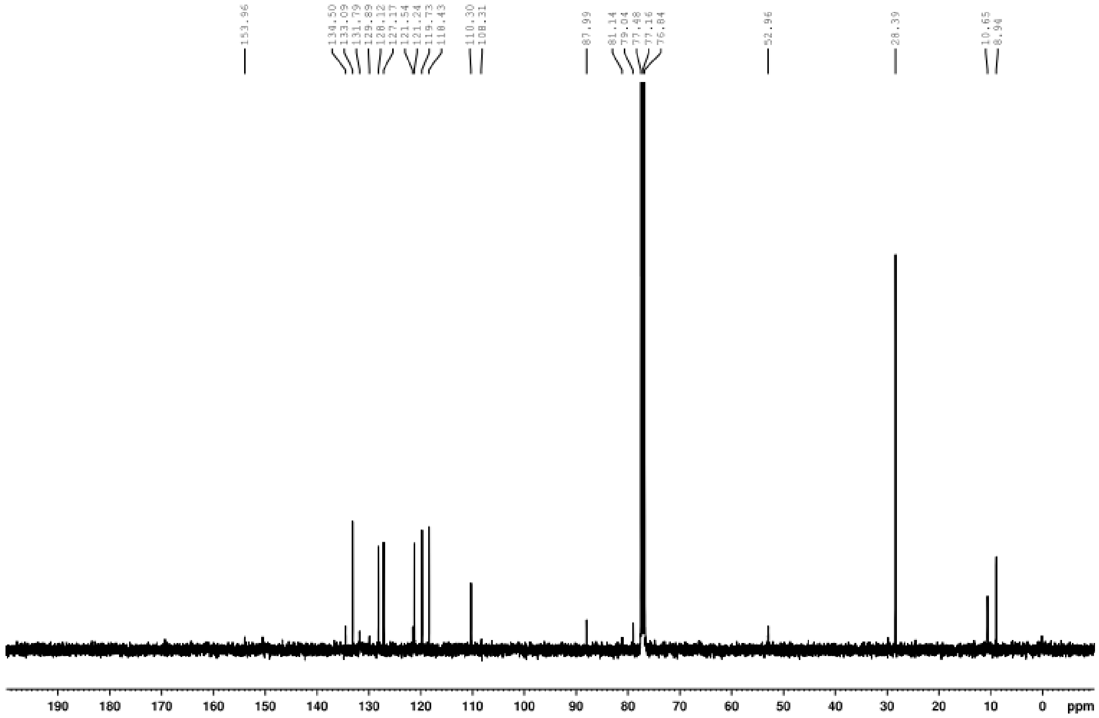

Supplementary Figure 34. ^{1}H and ^{13}C-NMR spectrum for **5i**

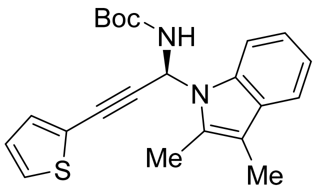

5i

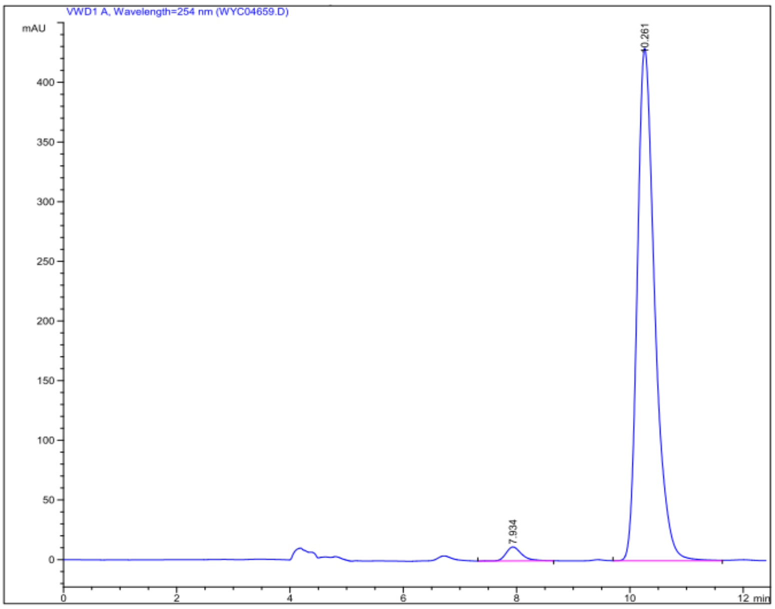

Peak #	RetTime [min]	Type	Width [min]	Area mAU *s	Height [mAU]	Area %
1	7.934	VB	0.2972	227.34654	11.66886	2.4071
2	10.261	VB	0.3275	9217.50586	429.57422	97.5929

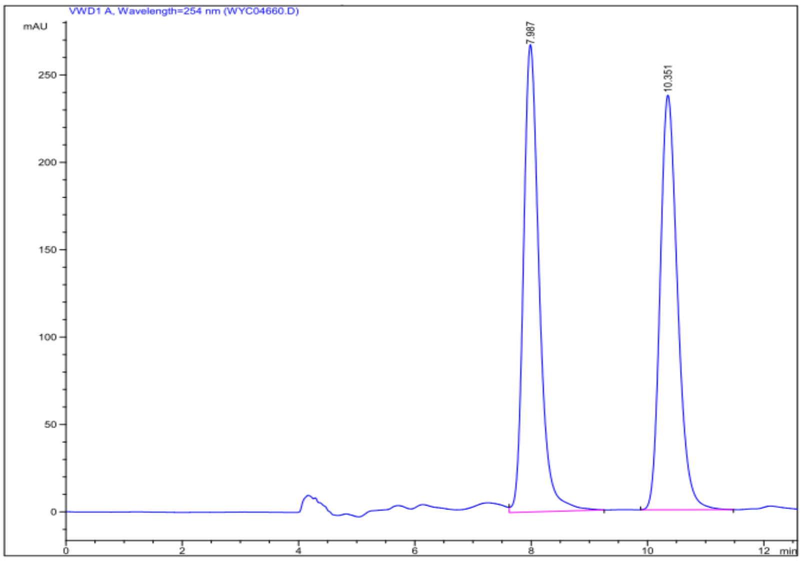

Peak #	RetTime [min]	Type	Width [min]	Area mAU *s	Height [mAU]	Area %
1	7.987	VB	0.2892	5027.10156	267.43787	50.4090
2	10.351	PB	0.3204	4945.52197	237.22501	49.5910

Supplementary Figure 35. HPLC spectrum for **5i**

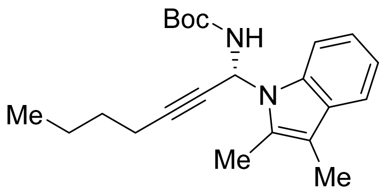

5j

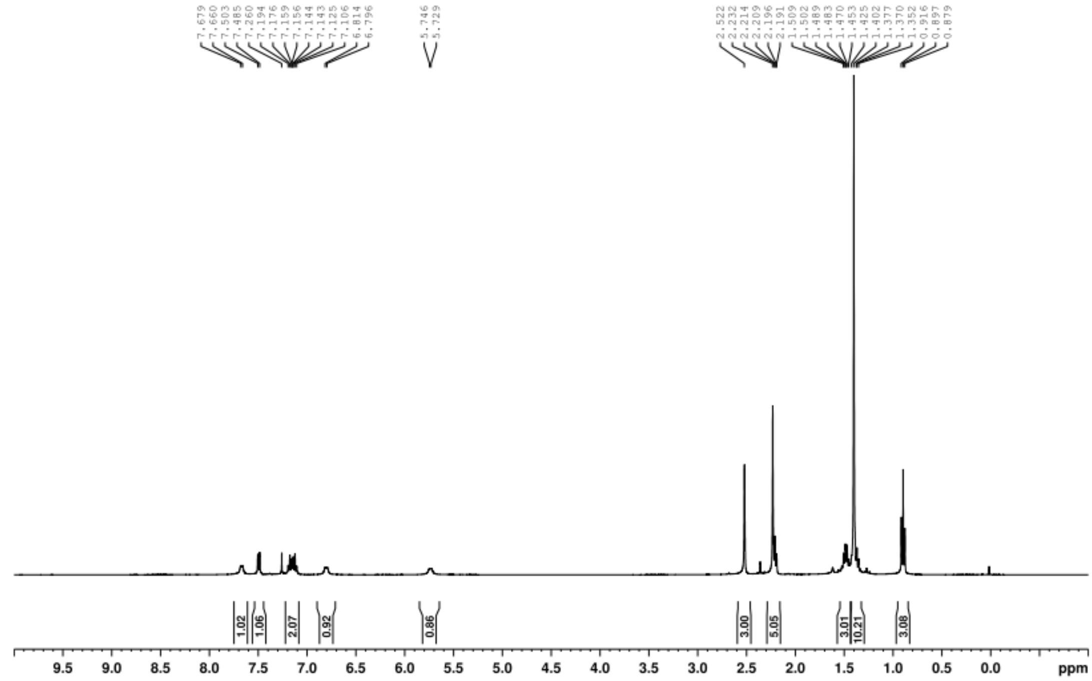

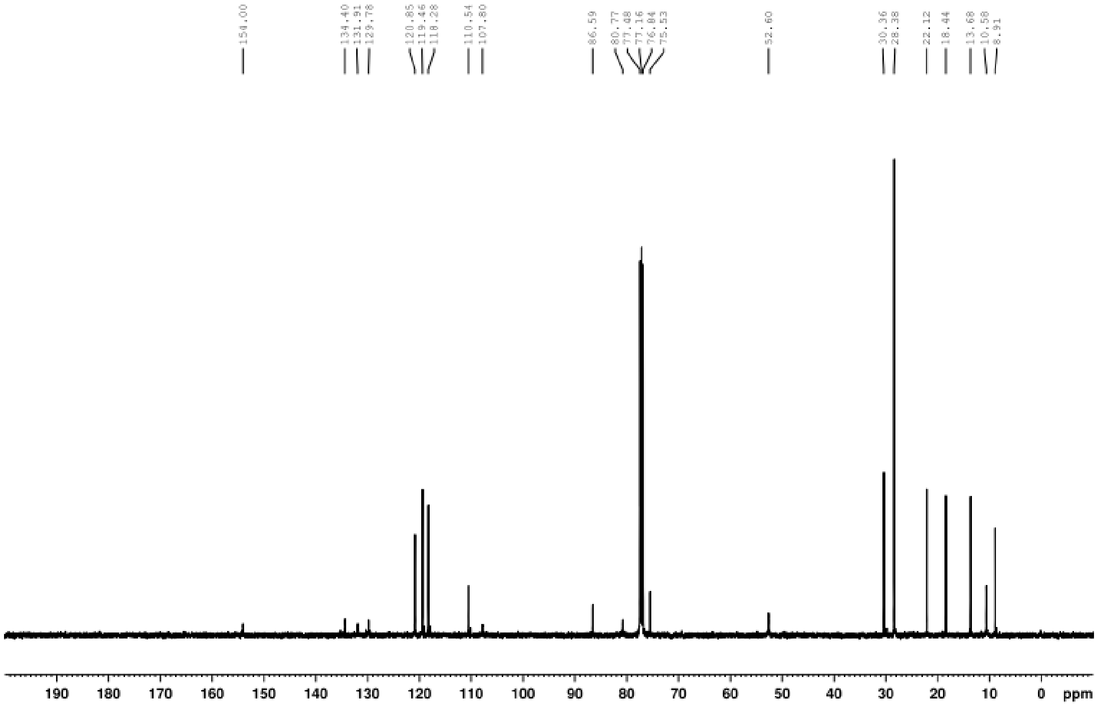

Supplementary Figure 36. ^{1}H and ^{13}C-NMR spectrum for **5j**

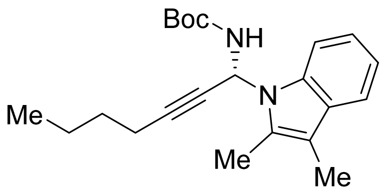

5j

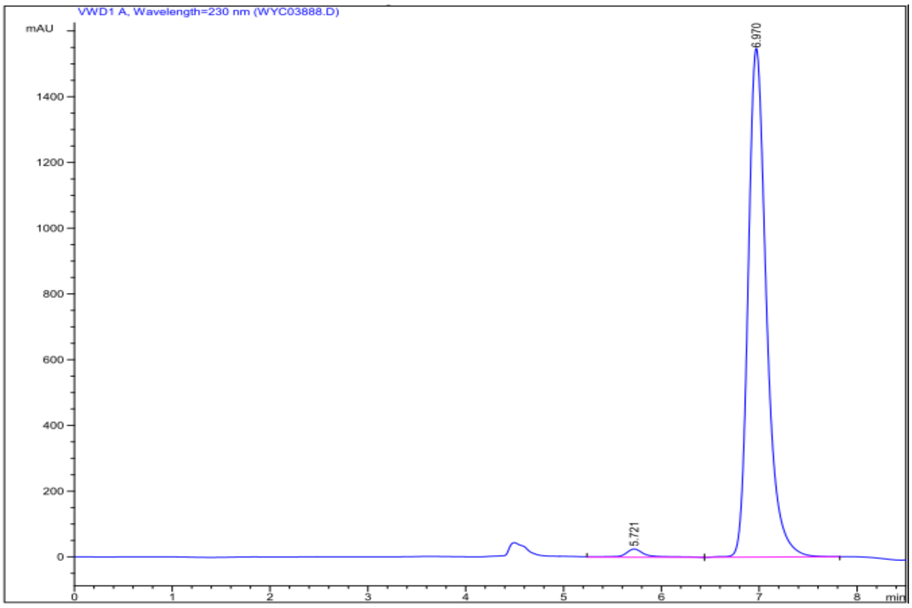

Peak #	RetTime [min]	Type	Width [min]	Area mAU *s	Height [mAU]	Area %
1	5.721	VP	0.2137	375.98413	25.15408	1.8343
2	6.970	VB	0.1997	2.01215e4	1547.95142	98.1657

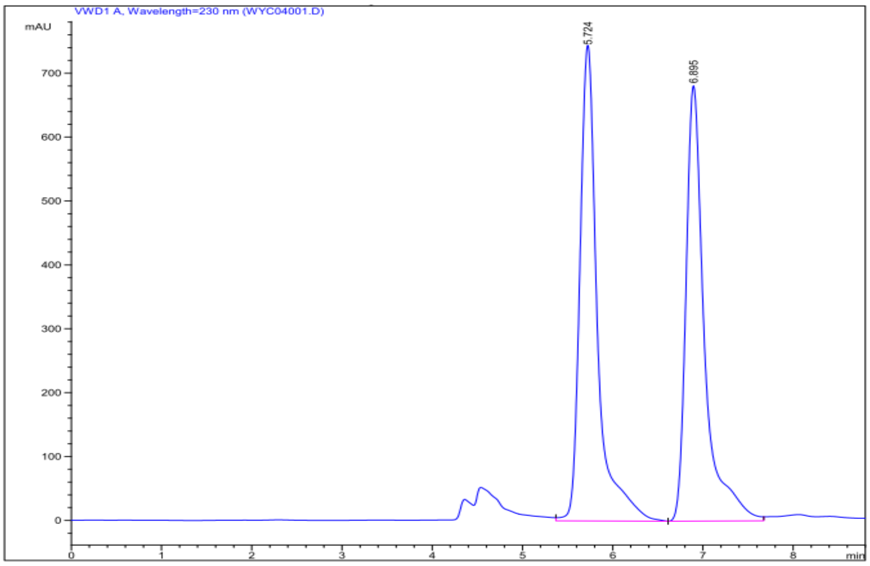

Peak #	RetTime [min]	Type	Width [min]	Area mAU *s	Height [mAU]	Area %
1	5.724	VP	0.2041	1.00513e4	744.57904	50.8758
2	6.895	VV	0.2099	9705.23926	681.37634	49.1242

Supplementary Figure 37. HPLC spectrum for **5j**

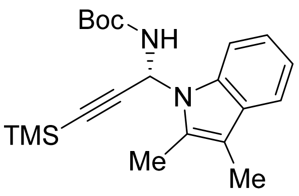

5k

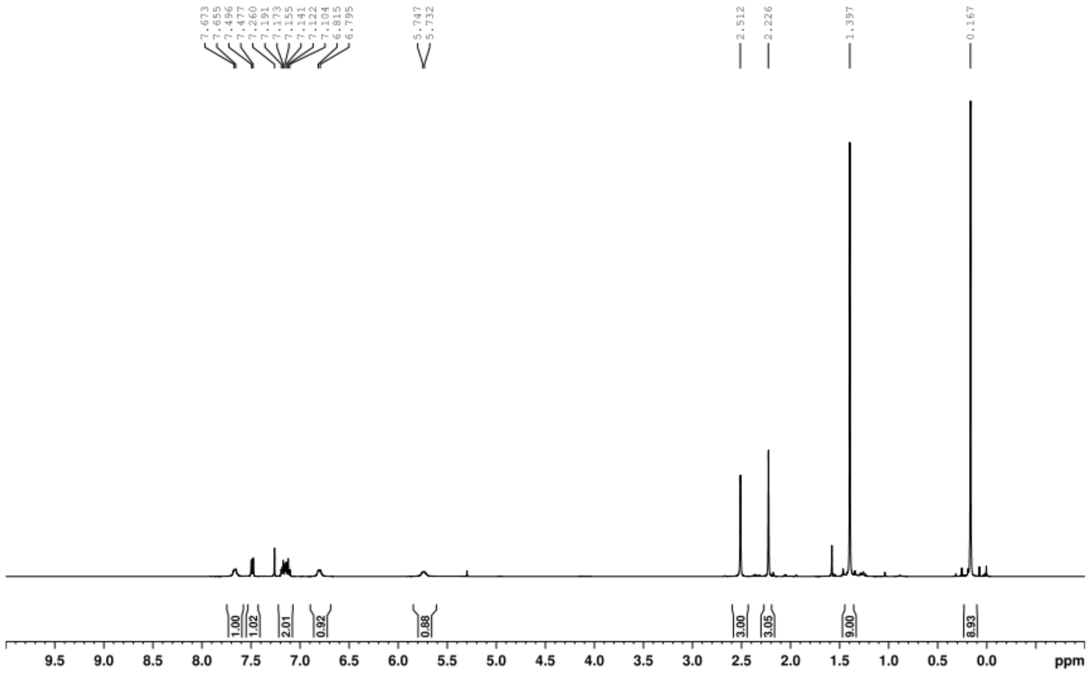

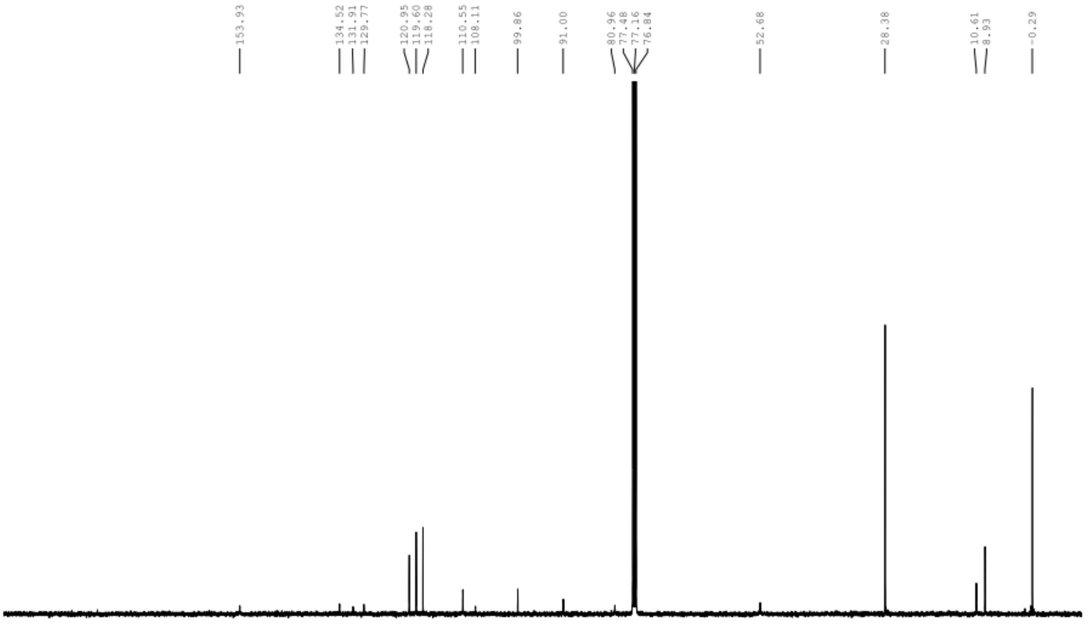

Supplementary Figure 38. ^{1}H and ^{13}C-NMR spectrum for **5k**

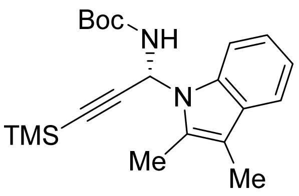

5k

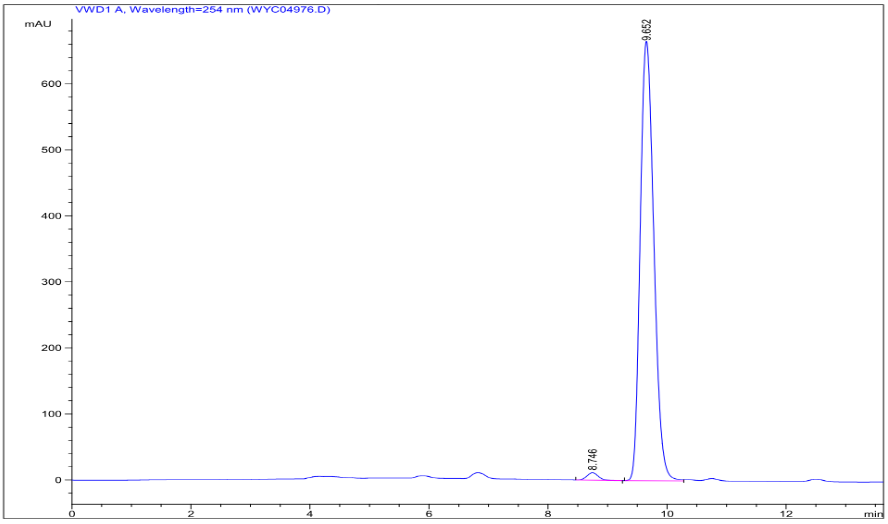

Peak #	RetTime [min]	Type	Width [min]	Area mAU *s	Height [mAU]	Area %
1	8.746	BP	0.2053	151.07297	11.42260	1.4061
2	9.652	BV	0.2526	1.05930e4	665.86334	98.5939

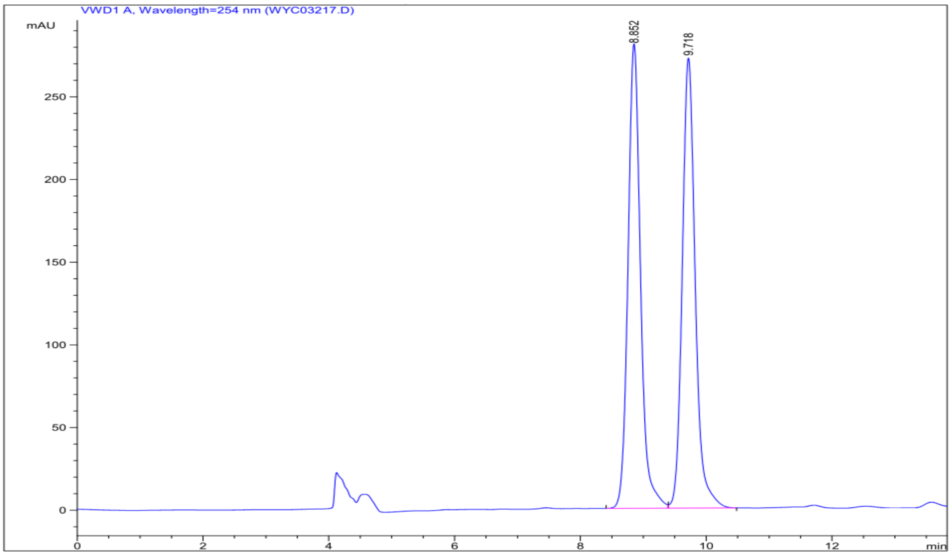

Peak #	RetTime [min]	Type	Width [min]	Area mAU *s	Height [mAU]	Area %
1	8.852	BV	0.2106	3879.56860	280.97919	49.8861
2	9.718	VB	0.2180	3897.28247	272.12311	50.1139

Supplementary Figure 39. HPLC spectrum for **5k**

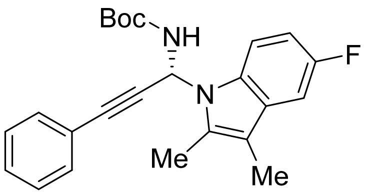

5l

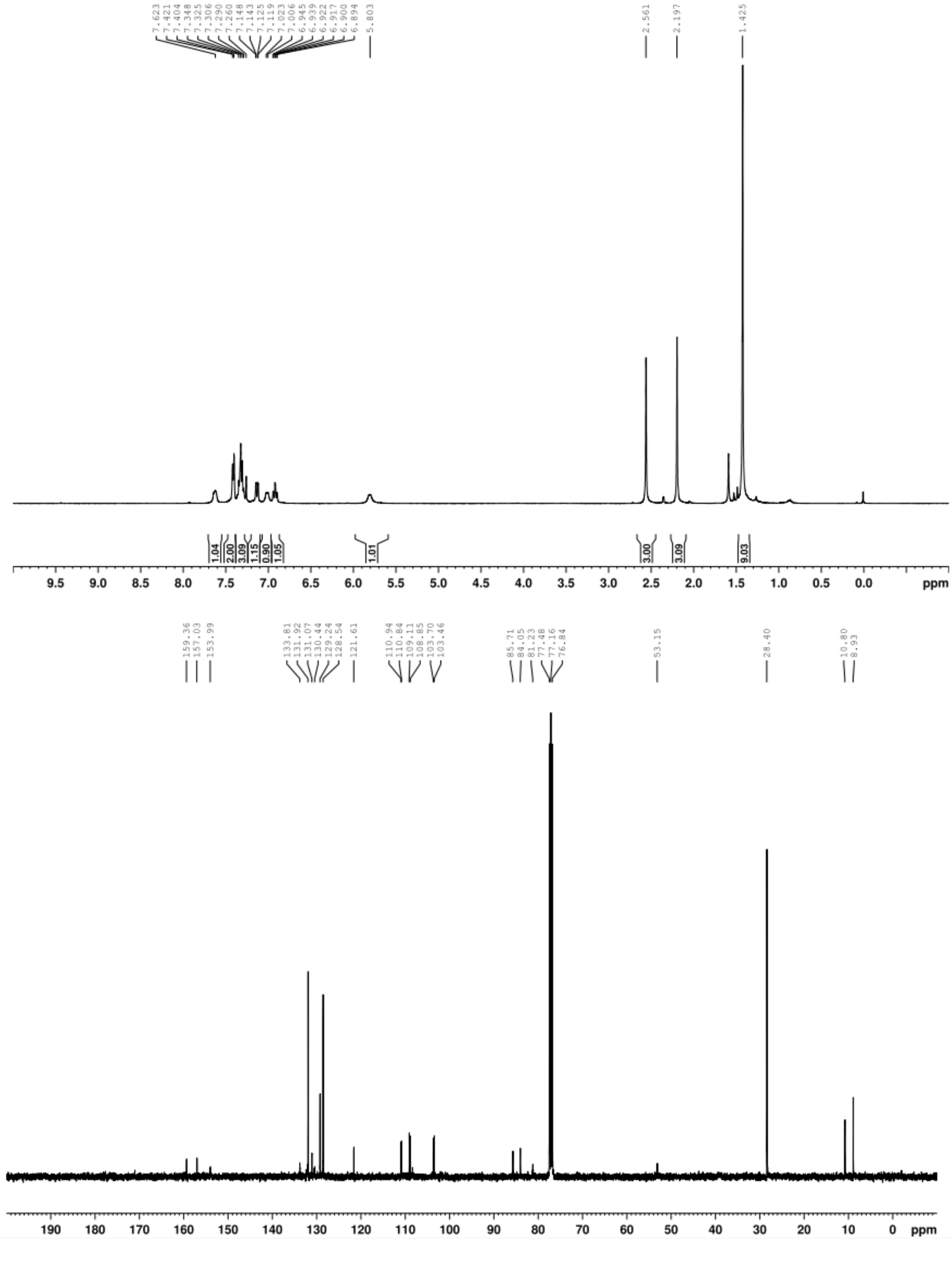

Supplementary Figure 40. ^{1}H and ^{13}C-NMR spectrum for **5l**

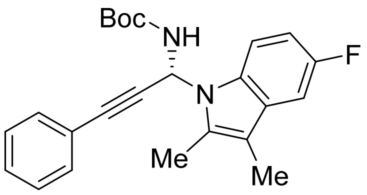

5l

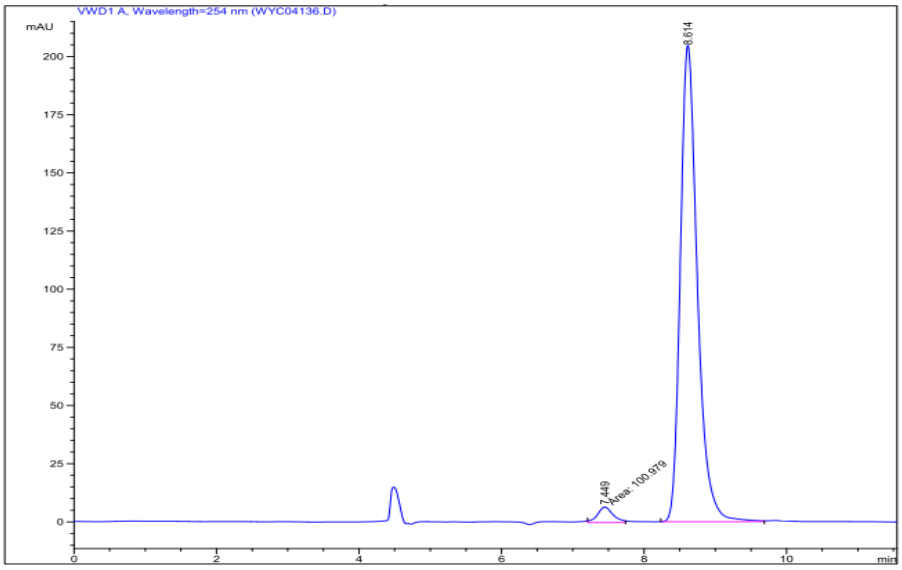

Peak #	RetTime [min]	Type	Width [min]	Area mAU *s	Height [mAU]	Area %
1	7.449	MM	0.2537	100.97854	6.63452	2.8888
2	8.614	BB	0.2532	3394.50342	204.67531	97.1112

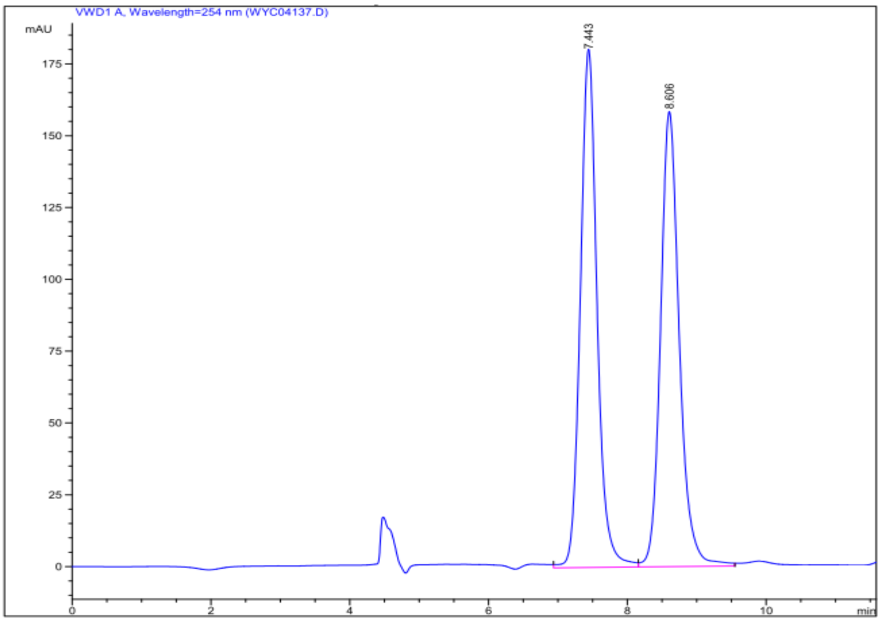

Peak #	RetTime [min]	Type	Width [min]	Area mAU *s	Height [mAU]	Area %
1	7.443	VV	0.2553	3023.28955	180.32428	50.3735
2	8.606	VB	0.2864	2978.46069	158.34947	49.6265

Supplementary Figure 41. HPLC spectrum for **5l**

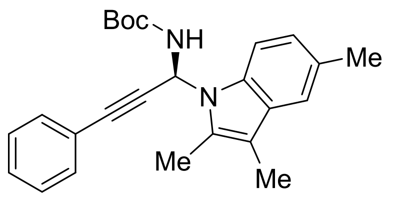

5m

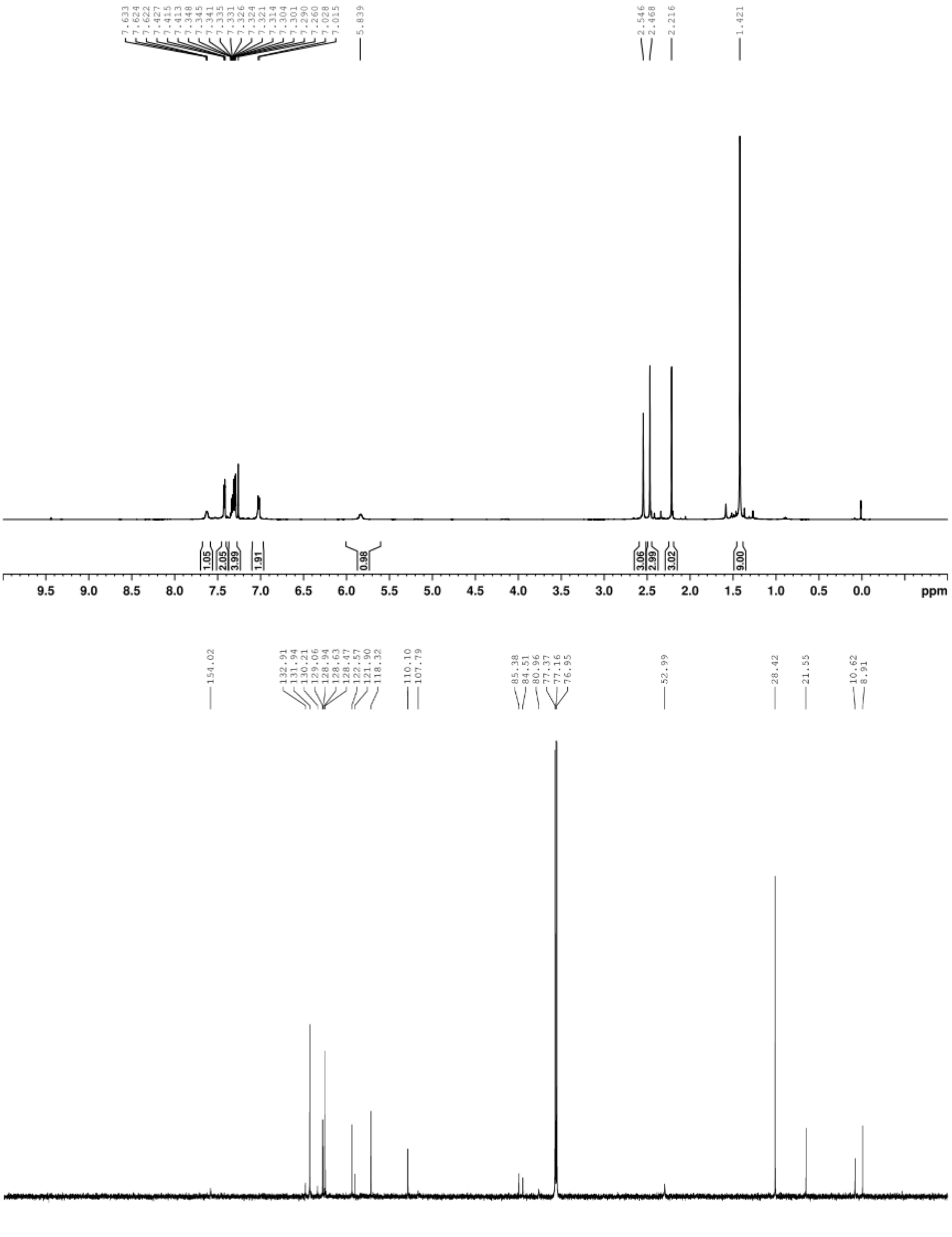

Supplementary Figure 42. ^{1}H and ^{13}C-NMR spectrum for **5m**

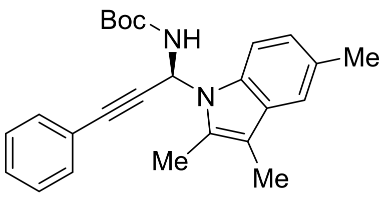

5m

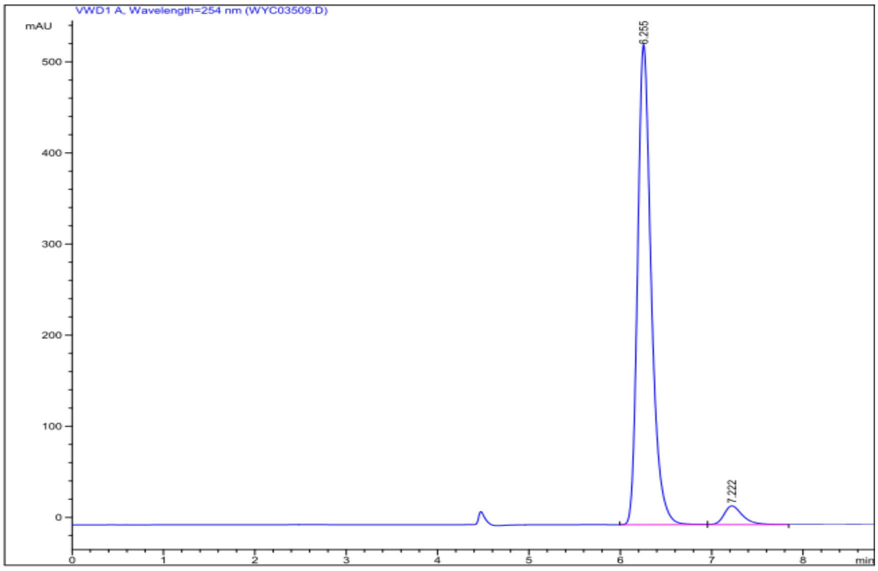

Peak #	RetTime [min]	Type	Width [min]	Area mAU *s	Height [mAU]	Area %
1	6.255	BV	0.1572	5454.06152	526.78619	94.9145
2	7.222	VB	0.2120	292.22678	20.61187	5.0855

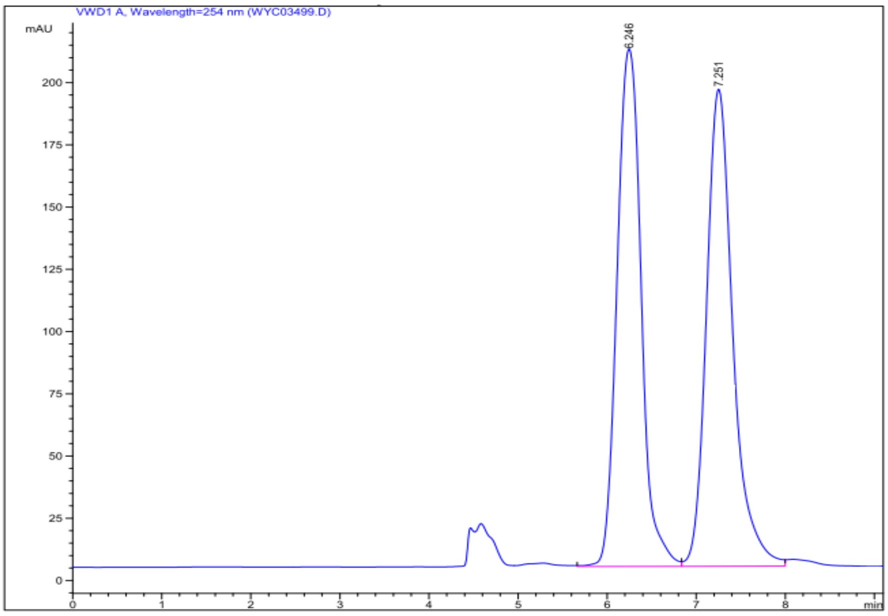

Peak #	RetTime [min]	Type	Width [min]	Area mAU *s	Height [mAU]	Area %
1	6.246	VV	0.2999	3916.90747	207.84843	49.7859
2	7.251	VV	0.3163	3950.59839	191.54413	50.2141

Supplementary Figure 43. HPLC spectrum for **5m**

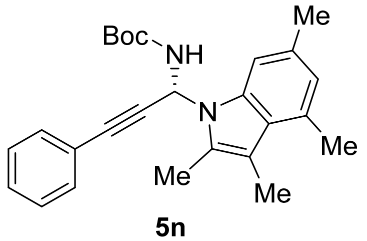

5n

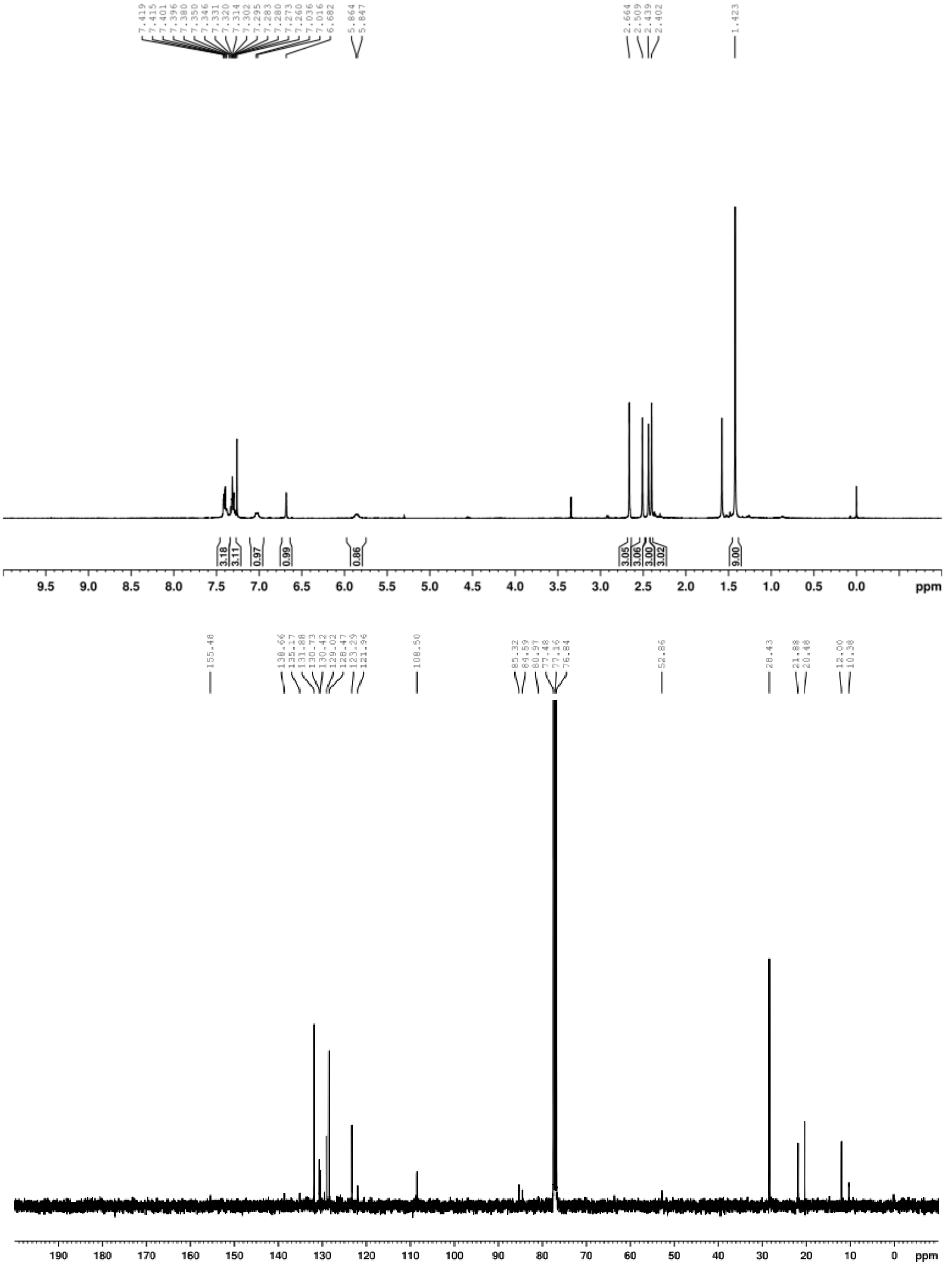

Supplementary Figure 44. ^{1}H and ^{13}C-NMR spectrum for **5n**

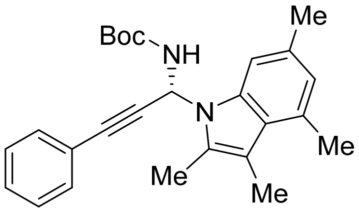

5n

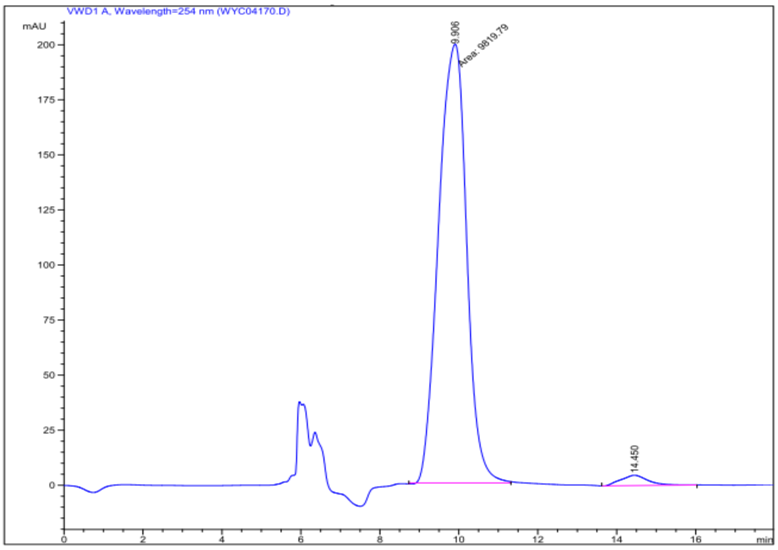

Peak #	RetTime [min]	Type	Width [min]	Area mAU *s	Height [mAU]	Area %
1	9.906	MM	0.8215	9819.78809	199.21681	97.7387
2	14.450	PB	0.6569	227.19518	4.70216	2.2613

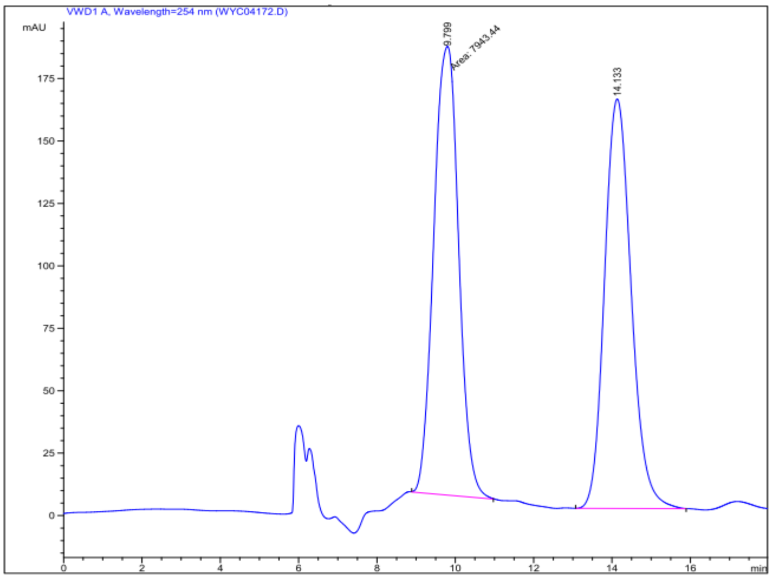

Peak #	RetTime [min]	Type	Width [min]	Area mAU *s	Height [mAU]	Area %
1	9.799	MM	0.7369	7943.43750	179.67050	50.5934
2	14.133	VB	0.7353	7757.09424	163.99855	49.4066

Supplementary Figure 45. HPLC spectrum for **5n**

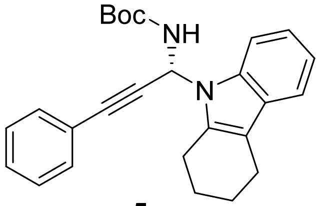

5o

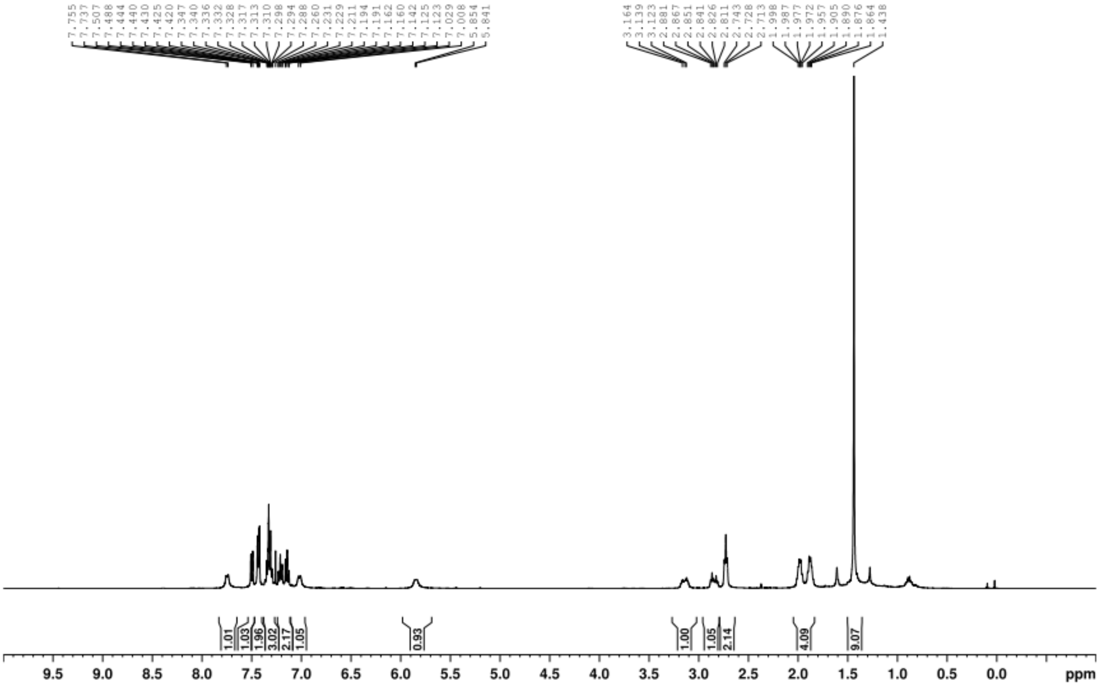

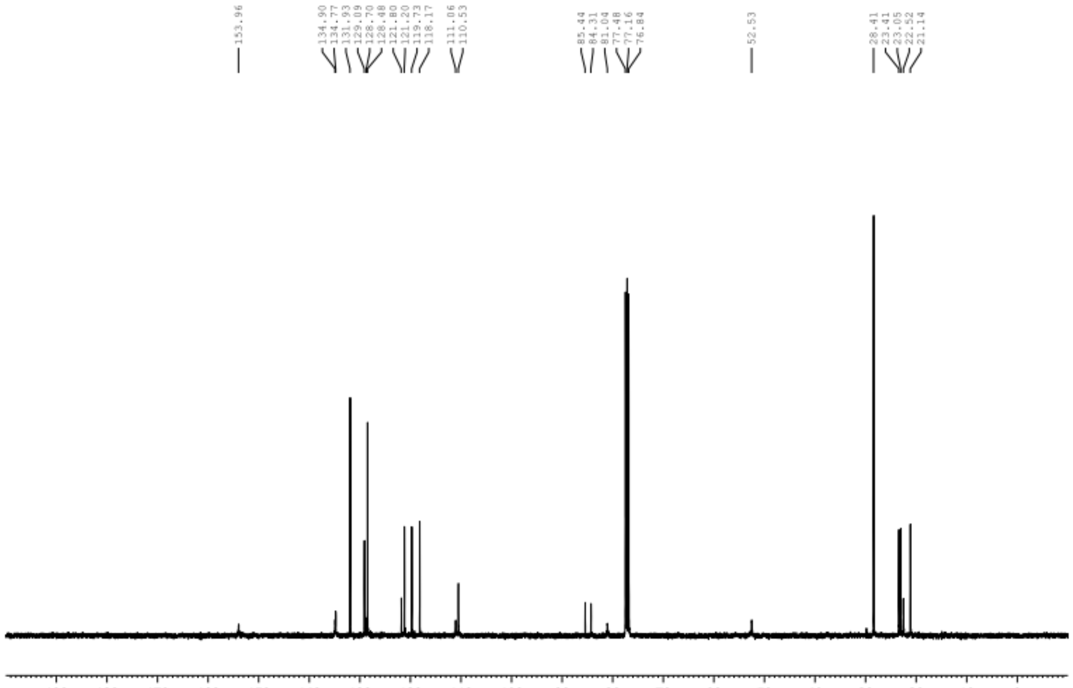

Supplementary Figure 46. ^{1}H and ^{13}C-NMR spectrum for **5o**

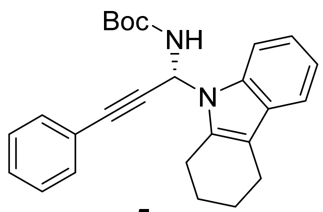

5o

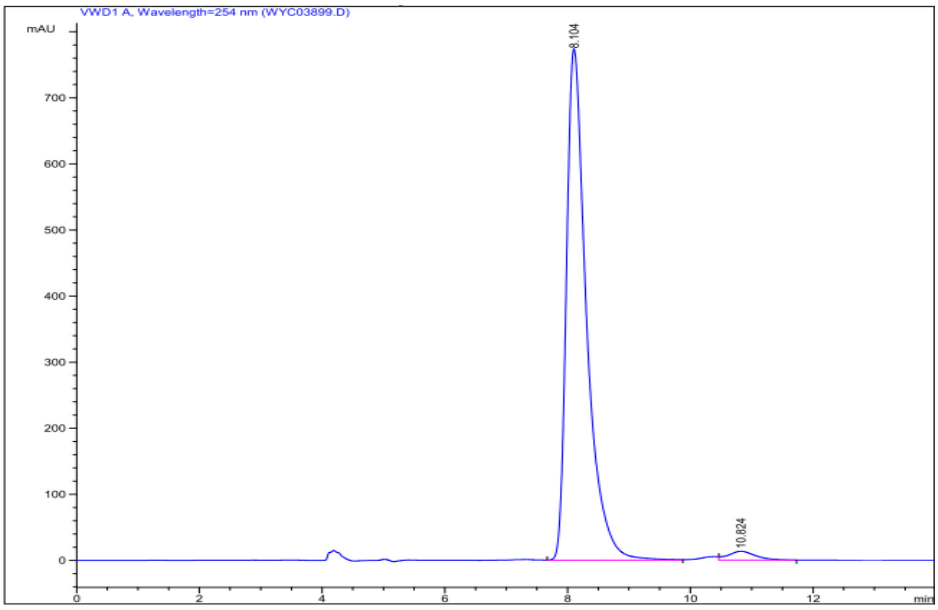

Peak #	RetTime [min]	Type	Width [min]	Area mAU *s	Height [mAU]	Area %
1	8.104	VB	0.3410	1.75966e4	773.83374	97.6912
2	10.824	VB	0.4572	415.86713	13.38337	2.3088

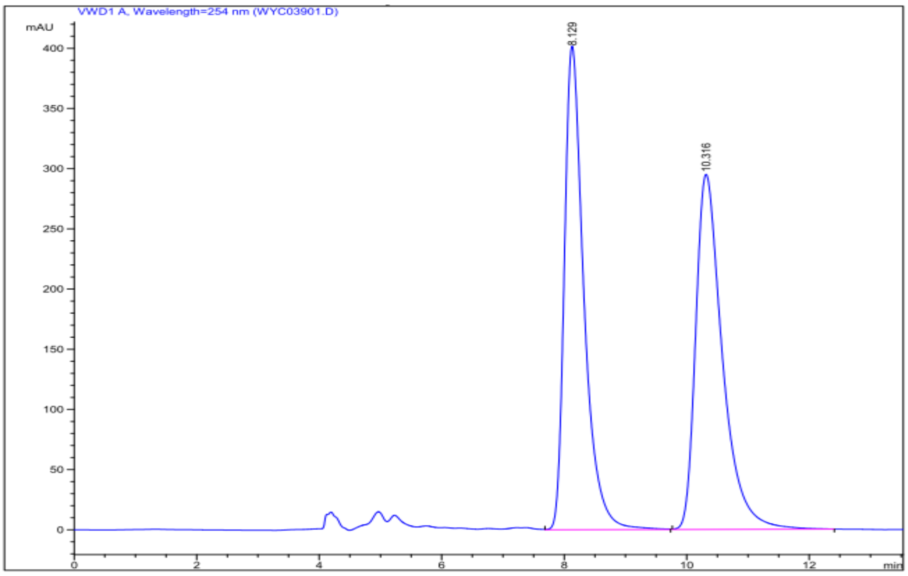

Peak #	RetTime [min]	Type	Width [min]	Area mAU *s	Height [mAU]	Area %
1	8.129	VB	0.3356	8899.59277	401.74197	49.8597
2	10.316	BB	0.4573	8949.67871	295.01956	50.1403

Supplementary Figure 47. HPLC spectrum for **5o**

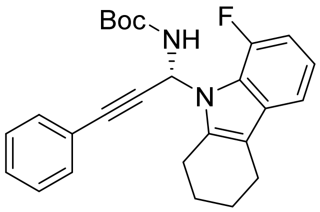

5p

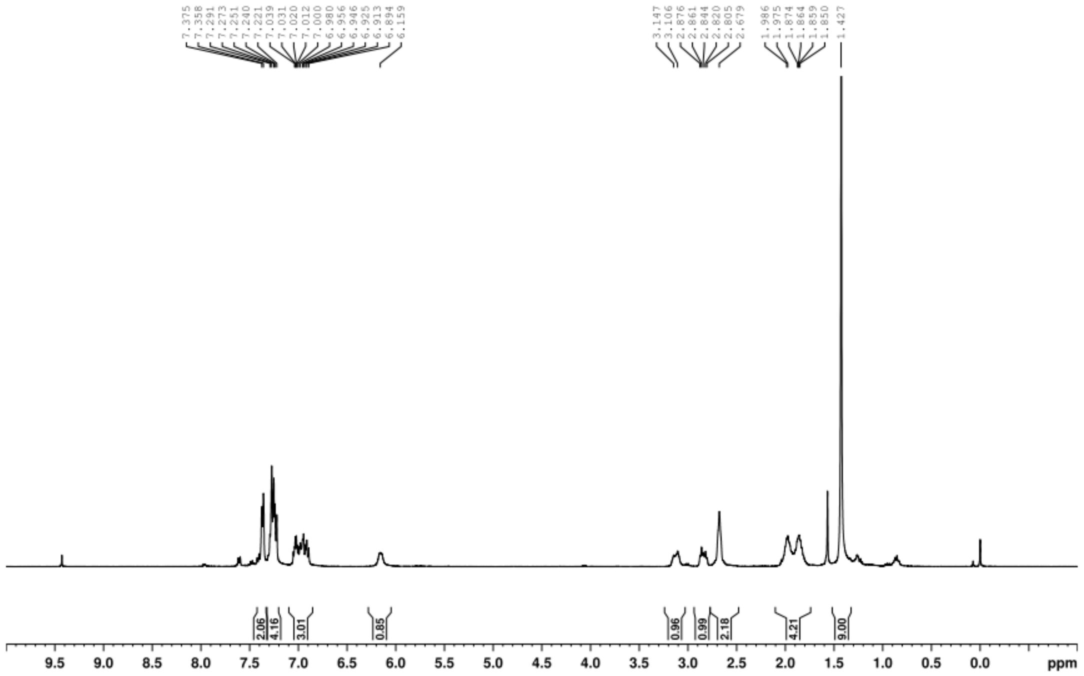

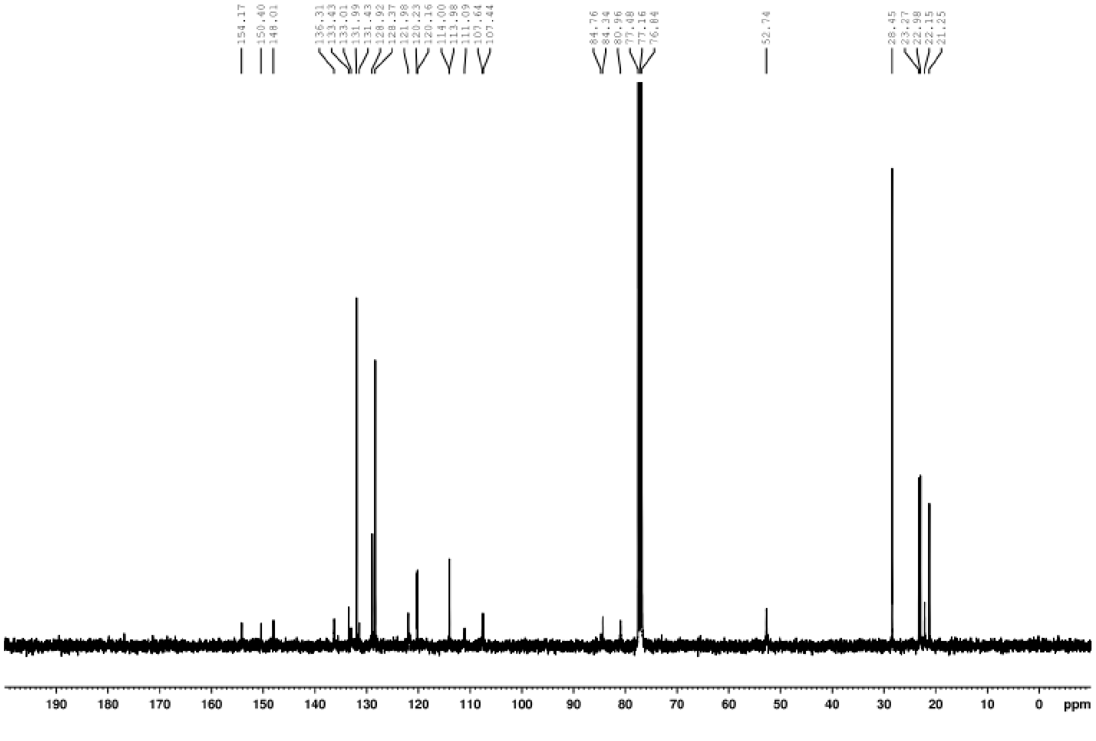

Supplementary Figure 48. ^{1}H and ^{13}C-NMR spectrum for **5p**

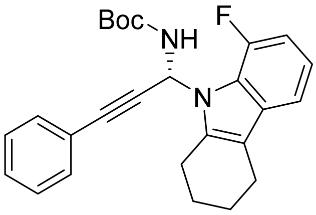

5p

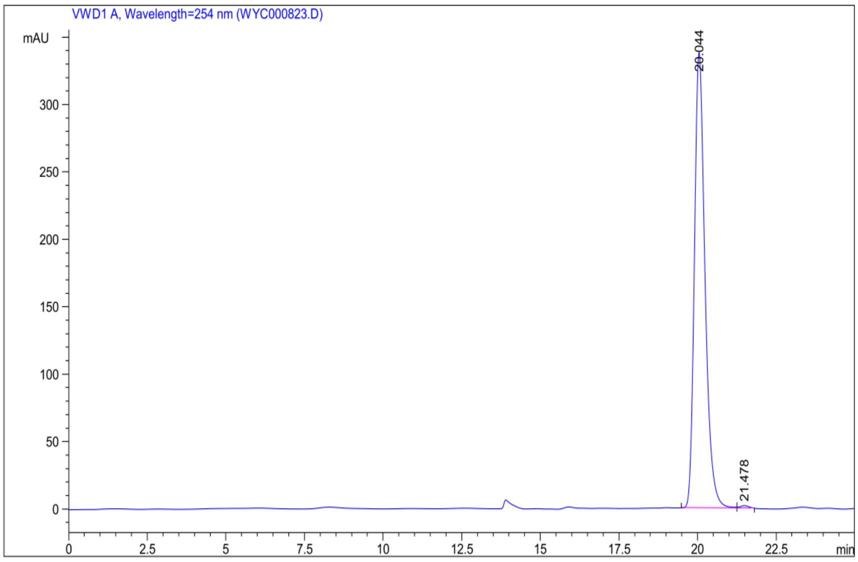

Peak #	RetTime [min]	Type	Width [min]	Area mAU *s	Height [mAU]	Area %
1	20.044	MF	0.3905	7919.42773	337.99261	99.5455
2	21.478	FM	0.3212	36.15817	1.87629	0.4545

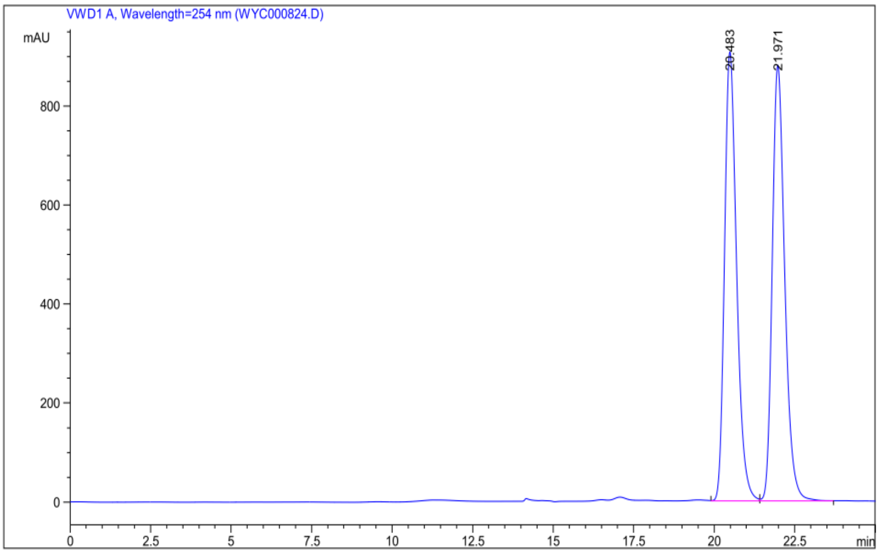

Peak #	RetTime [min]	Type	Width [min]	Area mAU *s	Height [mAU]	Area %
1	20.483	VV	0.4035	2.36766e4	907.30011	49.9354
2	21.971	VB	0.4194	2.37379e4	880.44171	50.0646

Supplementary Figure 49. HPLC spectrum for **5p**

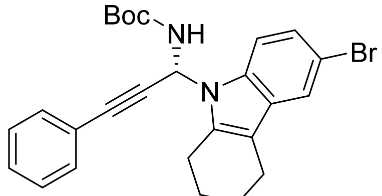

5q

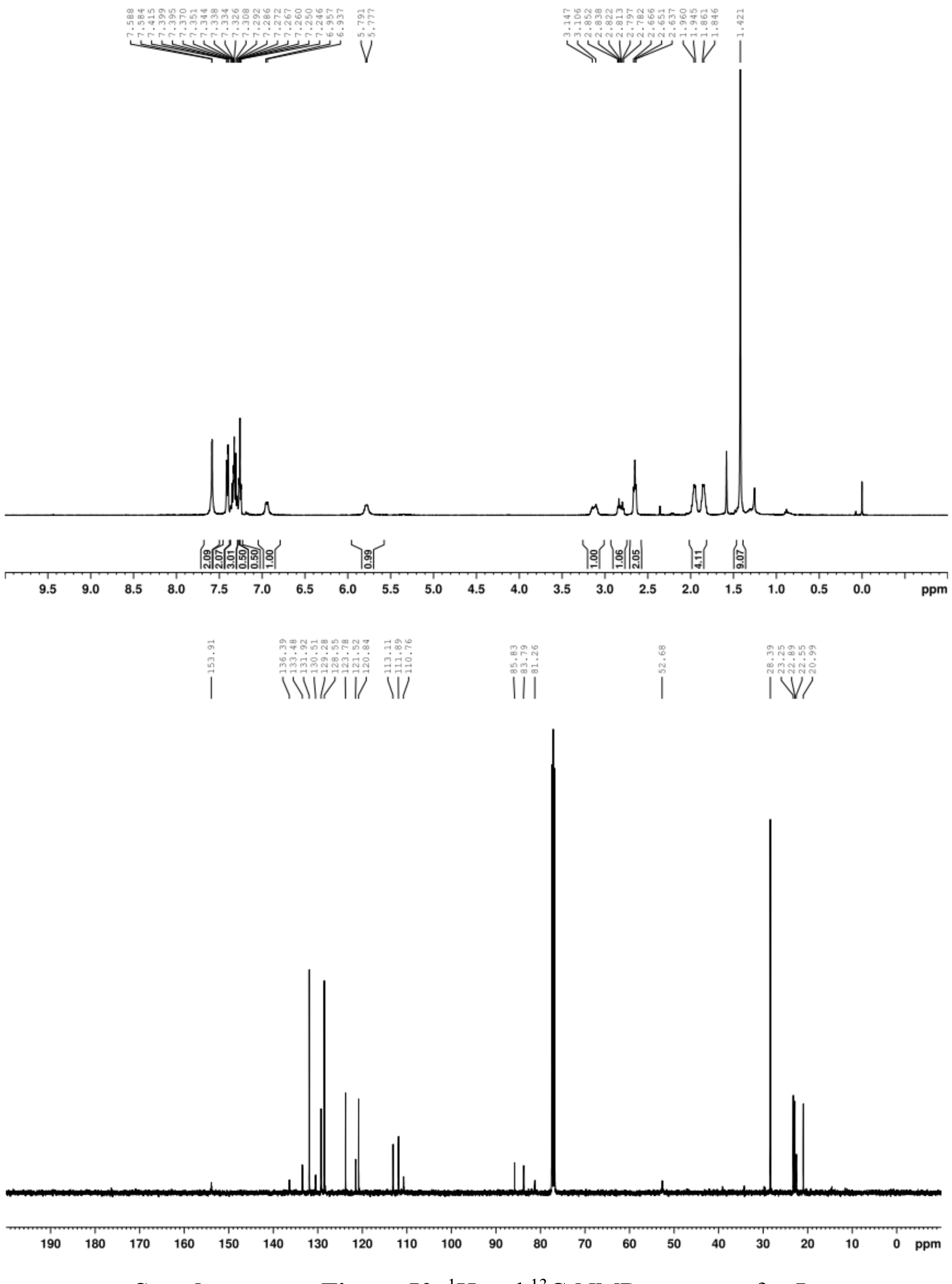

Supplementary Figure 50. ^{1}H and ^{13}C-NMR spectrum for **5q**

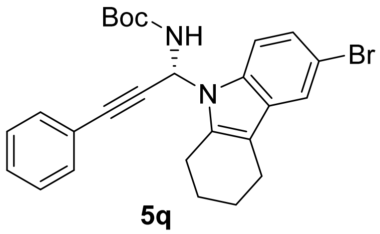

5q

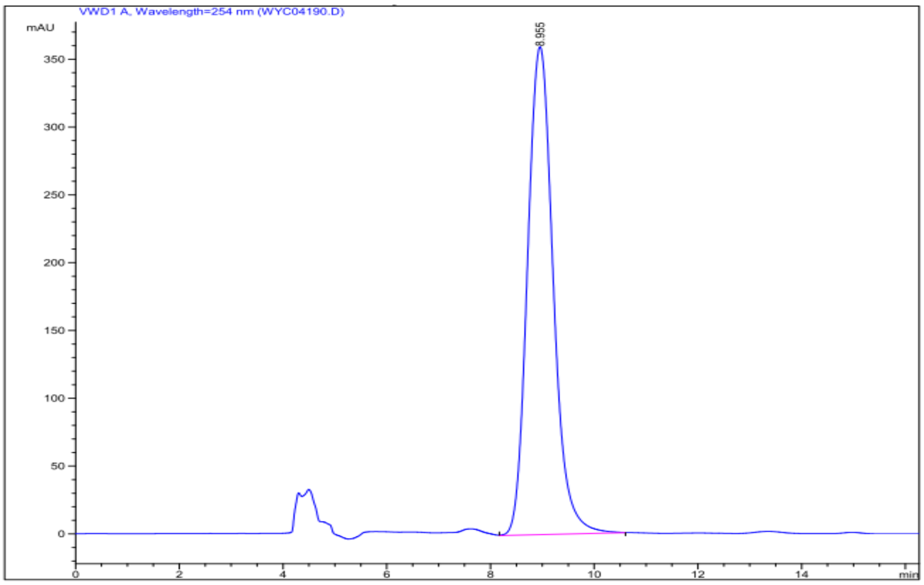

Peak #	RetTime [min]	Type	Width [min]	Area mAU *s	Height [mAU]	Area %
1	8.955	VB	0.5403	1.23497e4	359.74423	100.0000

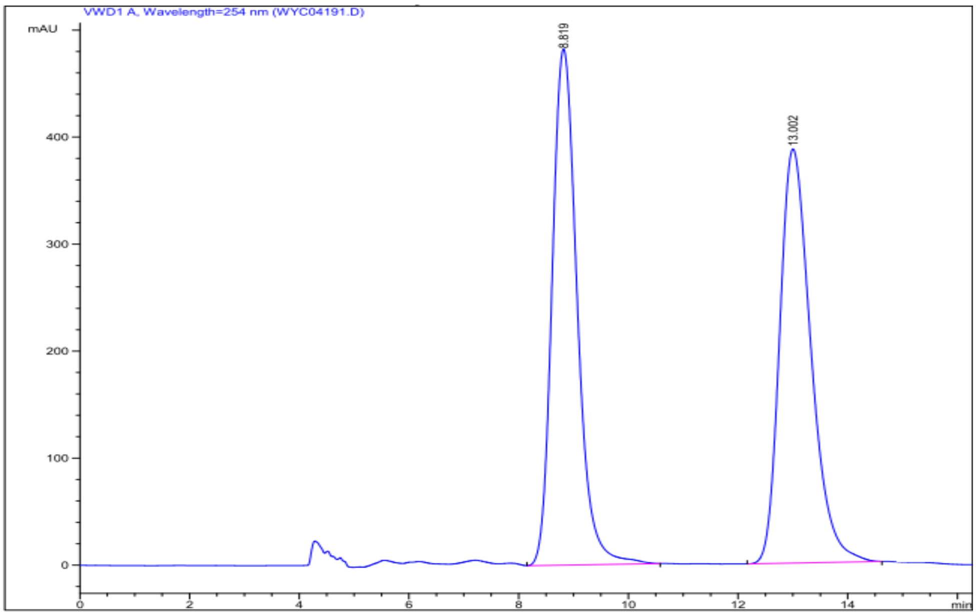

Peak #	RetTime [min]	Type	Width [min]	Area mAU *s	Height [mAU]	Area %
1	8.819	VB	0.4914	1.52574e4	482.30951	49.9772
2	13.002	PB	0.6060	1.52713e4	386.99756	50.0228

Supplementary Figure 51. HPLC spectrum for **5q**

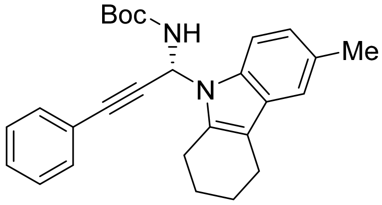

5r

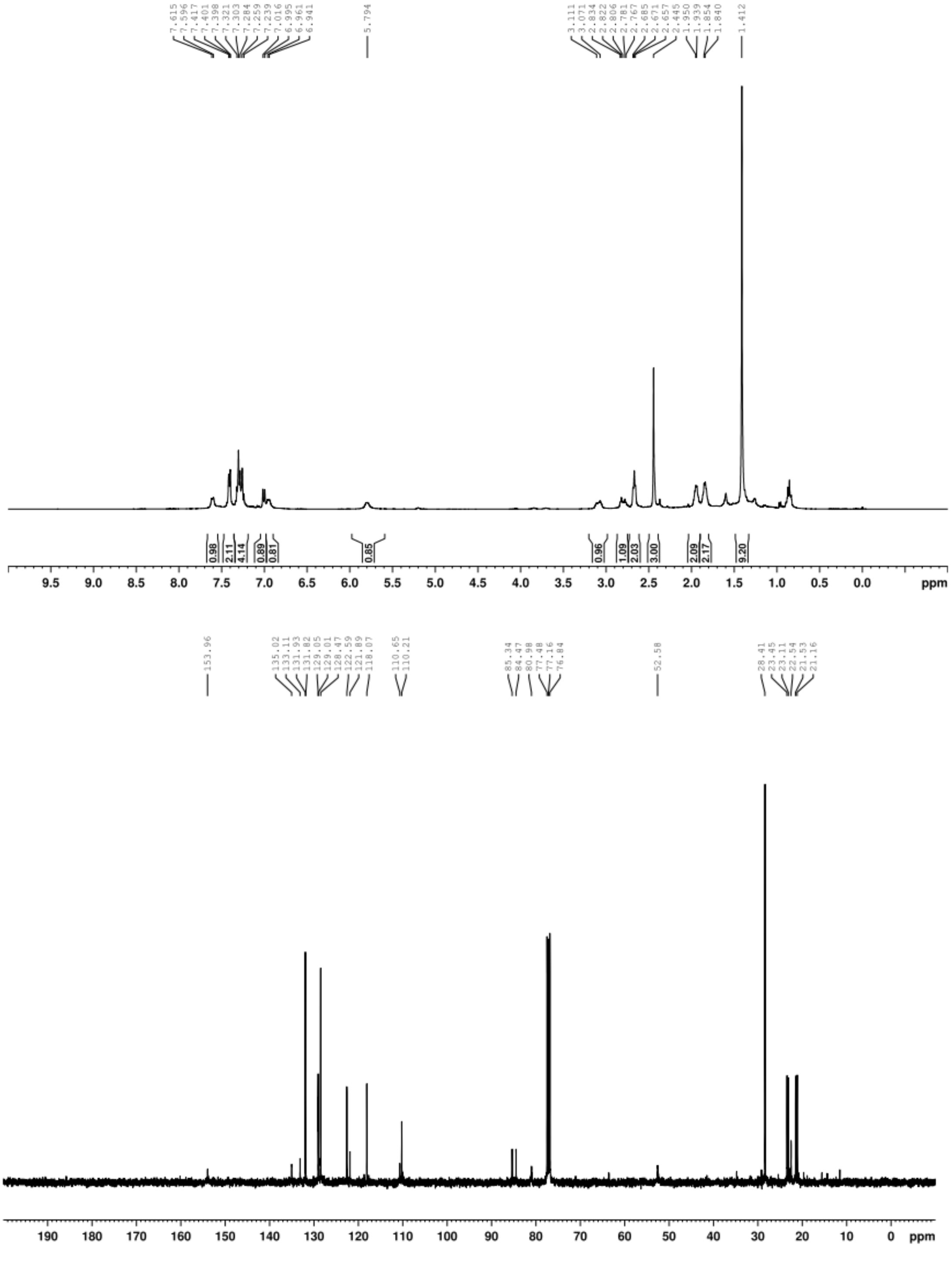

Supplementary Figure 52. ^{1}H and ^{13}C-NMR spectrum for **5r**

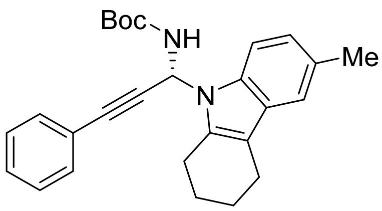

5r

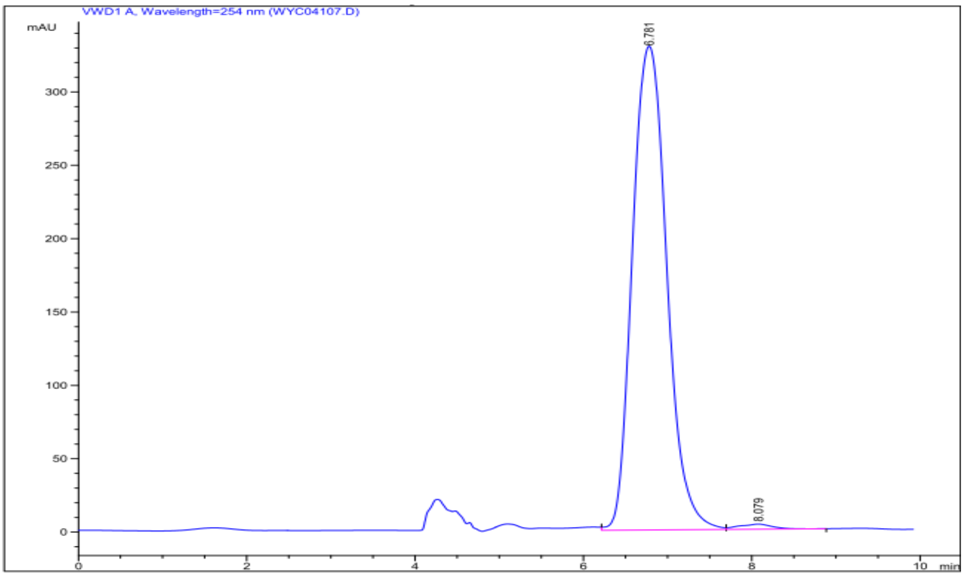

Peak #	RetTime [min]	Type	Width [min]	Area mAU *s	Height [mAU]	Area %
1	6.781	VV	0.4549	9361.19434	329.78592	98.9889
2	8.079	VP	0.3788	95.61584	3.48013	1.0111

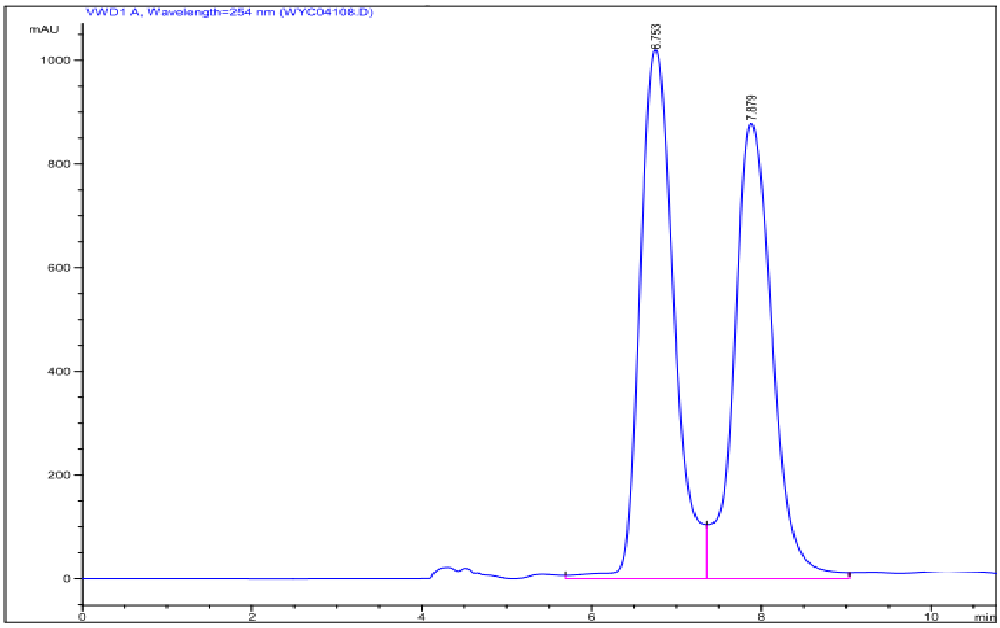

Peak #	RetTime [min]	Type	Width [min]	Area mAU *s	Height [mAU]	Area %
1	6.753	VV	0.4328	2.84194e4	1020.11853	50.8011
2	7.879	VV	0.4909	2.75231e4	878.10156	49.1989

Supplementary Figure 53. HPLC spectrum for **5r**

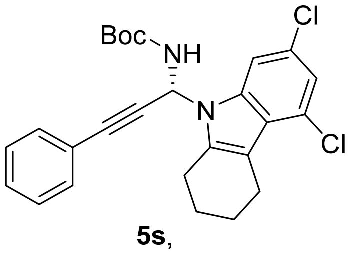

5s,

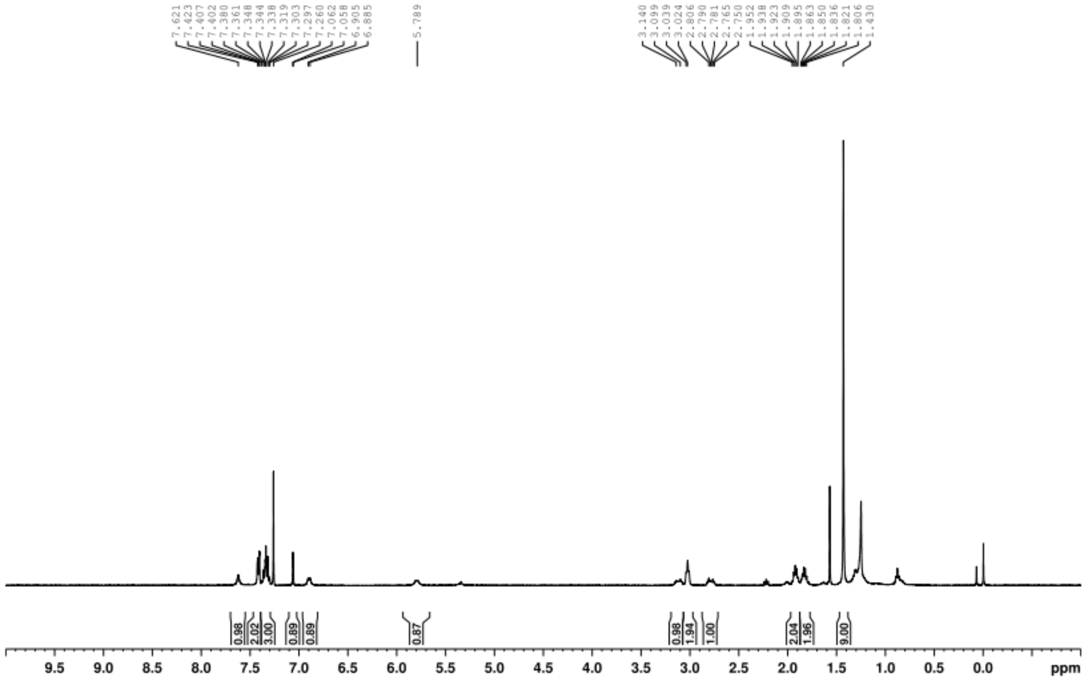

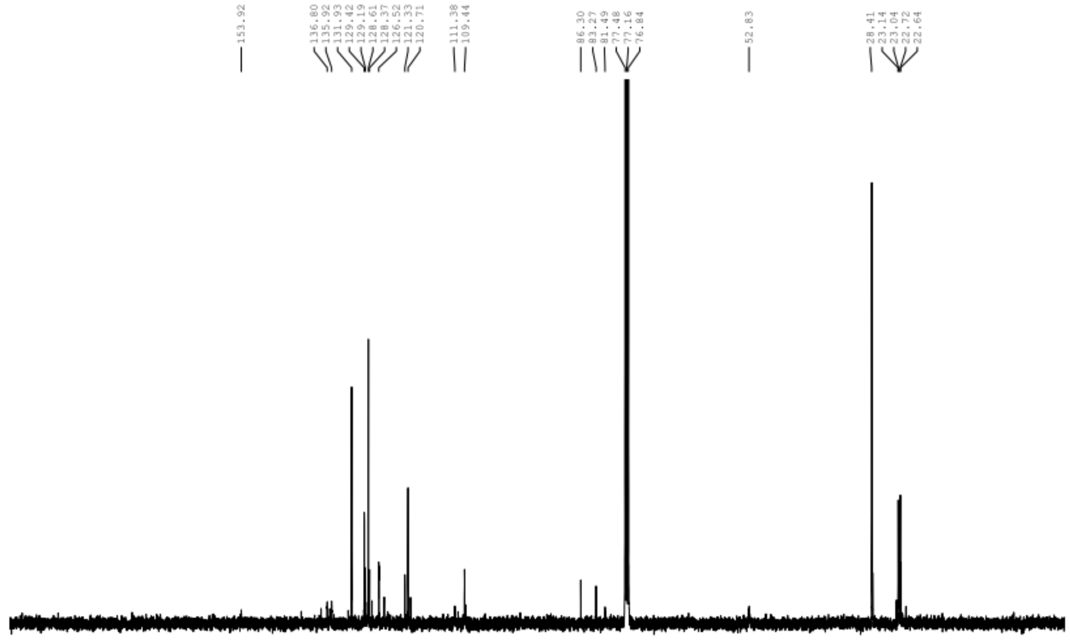

Supplementary Figure 54. ^{1}H and ^{13}C-NMR spectrum for **5s**

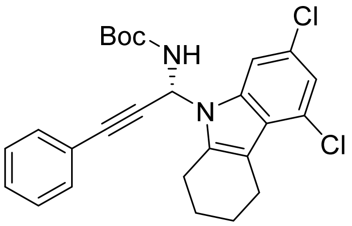

5s,

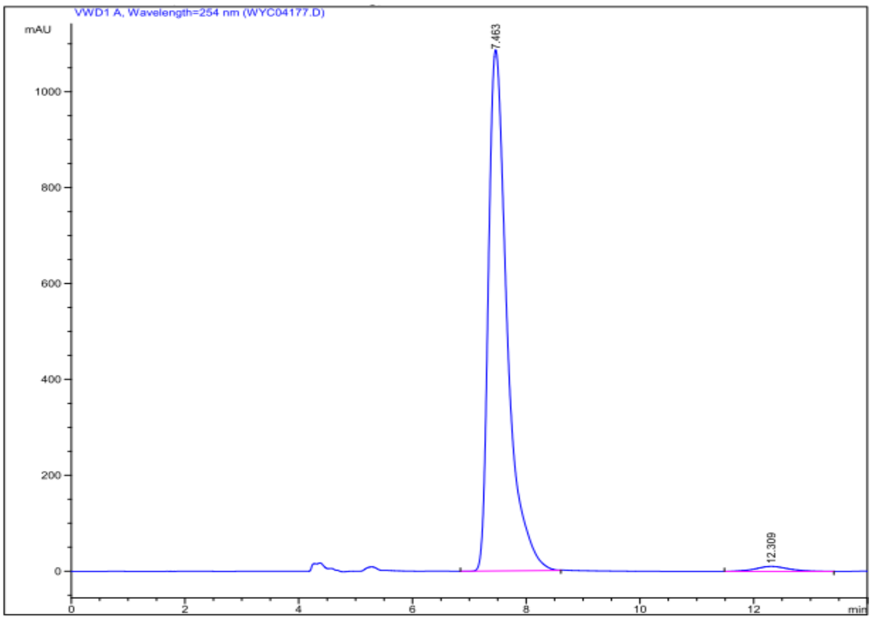

Peak #	RetTime [min]	Type	Width [min]	Area mAU *s	Height [mAU]	Area %
1	7.463	VB	0.3483	2.48476e4	1086.76880	98.3006
2	12.309	BB	0.6168	429.55988	10.44276	1.6994

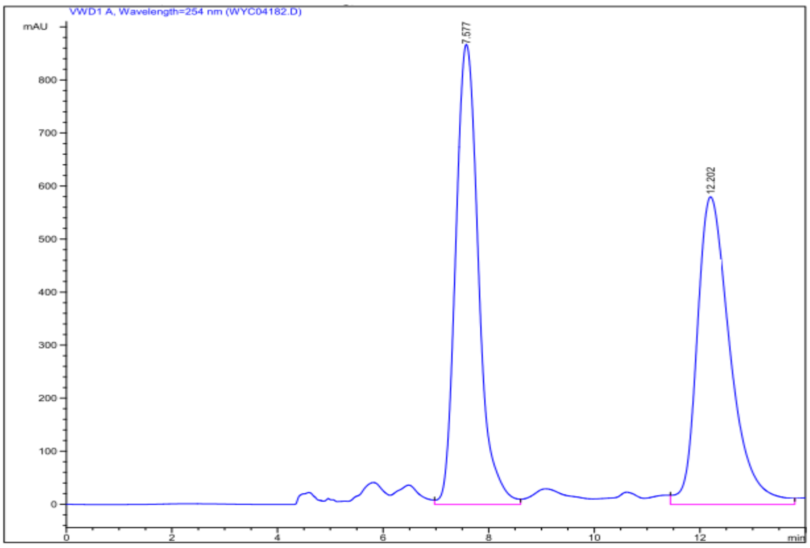

Peak #	RetTime [min]	Type	Width [min]	Area mAU *s	Height [mAU]	Area %
1	7.577	VV	0.4732	2.60838e4	867.33215	50.8023
2	12.202	VV	0.6667	2.52599e4	580.06702	49.1977

Supplementary Figure 55. HPLC spectrum for **5s**

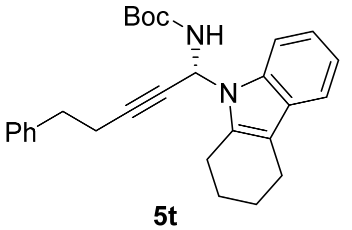

5t

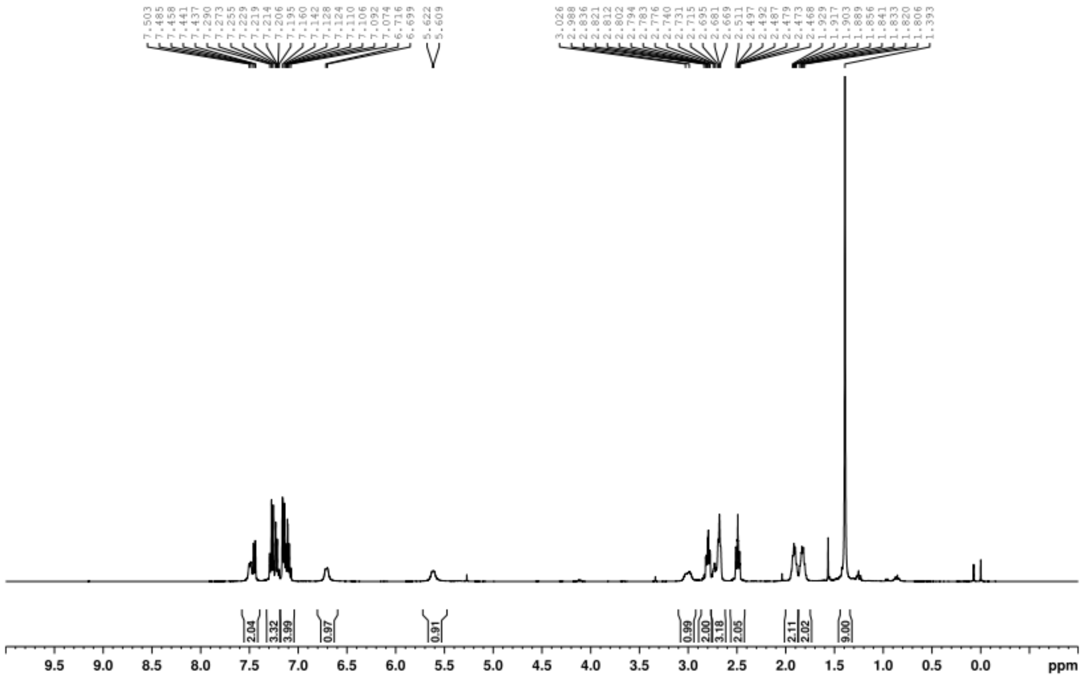

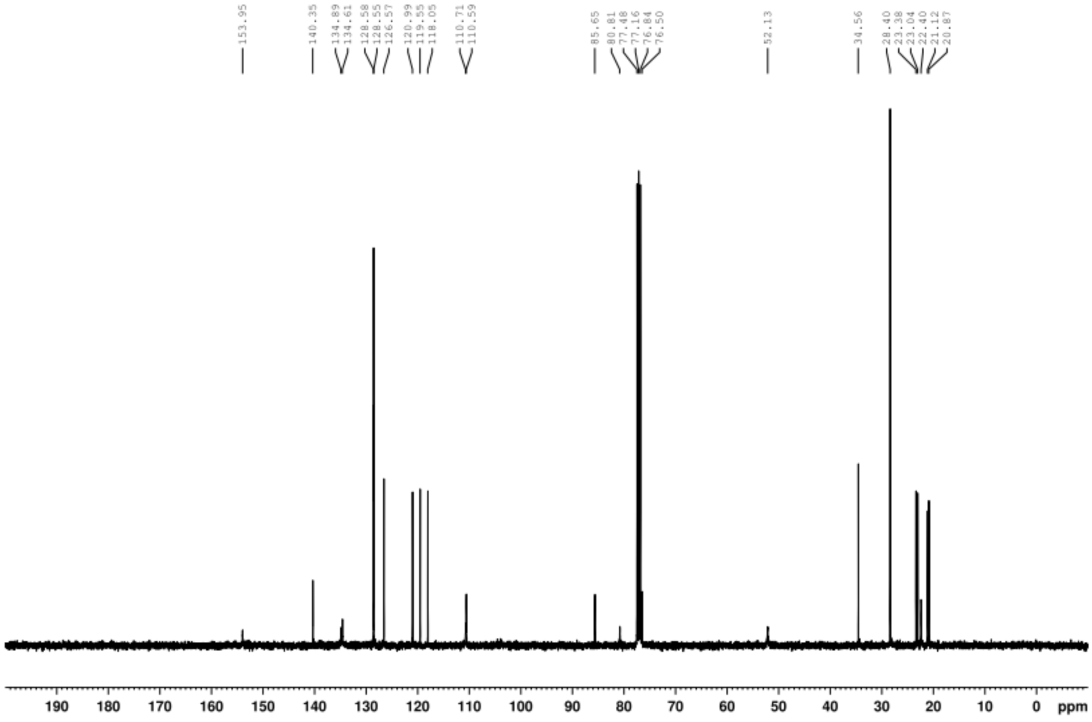

Supplementary Figure 56. ^{1}H and ^{13}C-NMR spectrum for **5t**

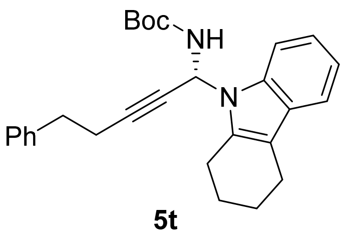

5t

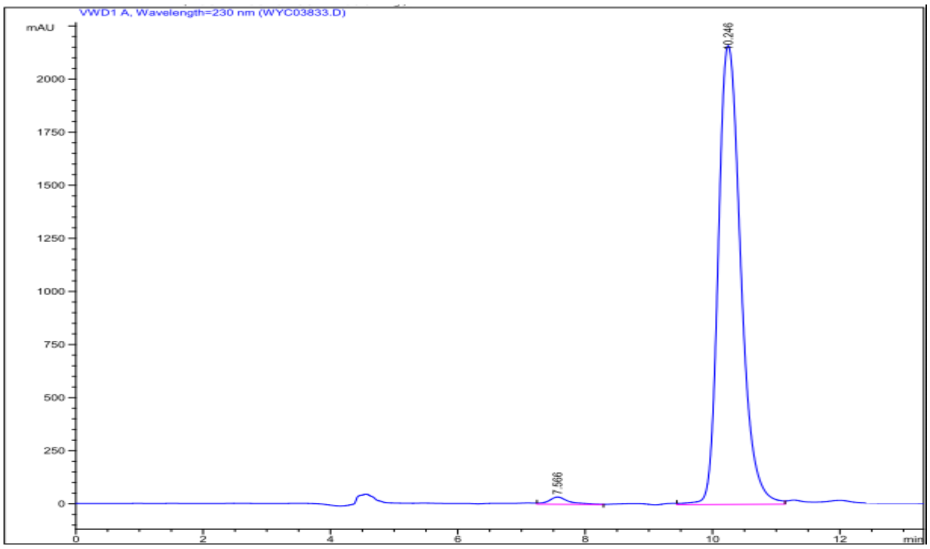

Peak #	RetTime [min]	Type	Width [min]	Area mAU *s	Height [mAU]	Area %
1	7.566	VV	0.3176	749.97510	34.31783	1.3970
2	10.246	VV	0.3804	5.29331e4	2161.12183	98.6030

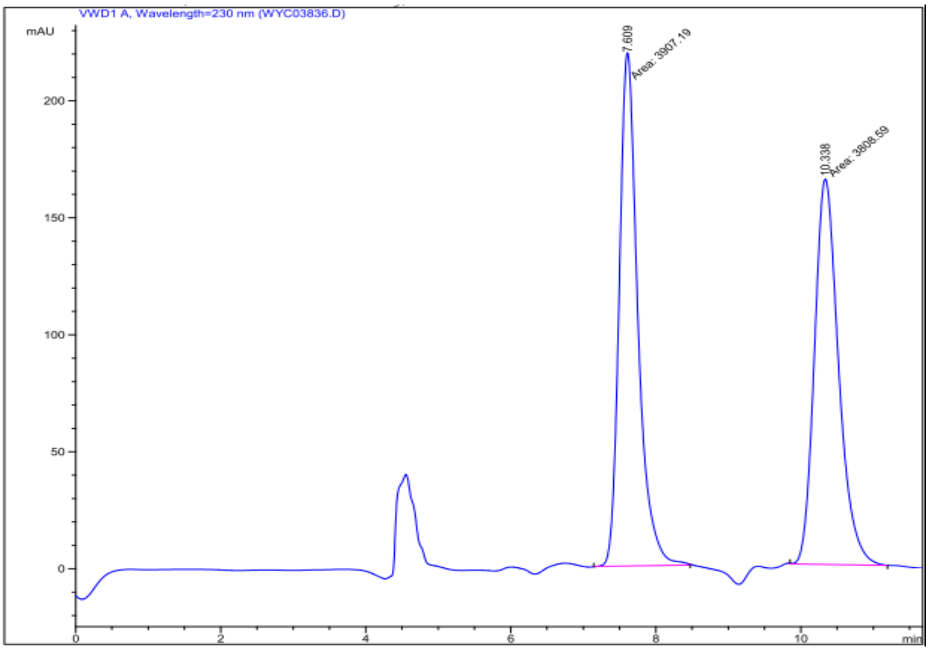

Peak #	RetTime [min]	Type	Width [min]	Area mAU *s	Height [mAU]	Area %
1	7.609	MM	0.2967	3907.19287	219.46524	50.6390
2	10.338	MM	0.3854	3808.59180	164.72064	49.3610

Supplementary Figure 57. HPLC spectrum for **5t**

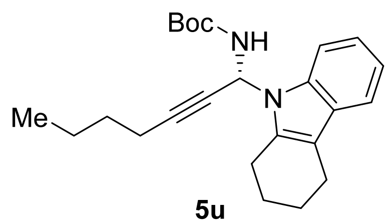

5u

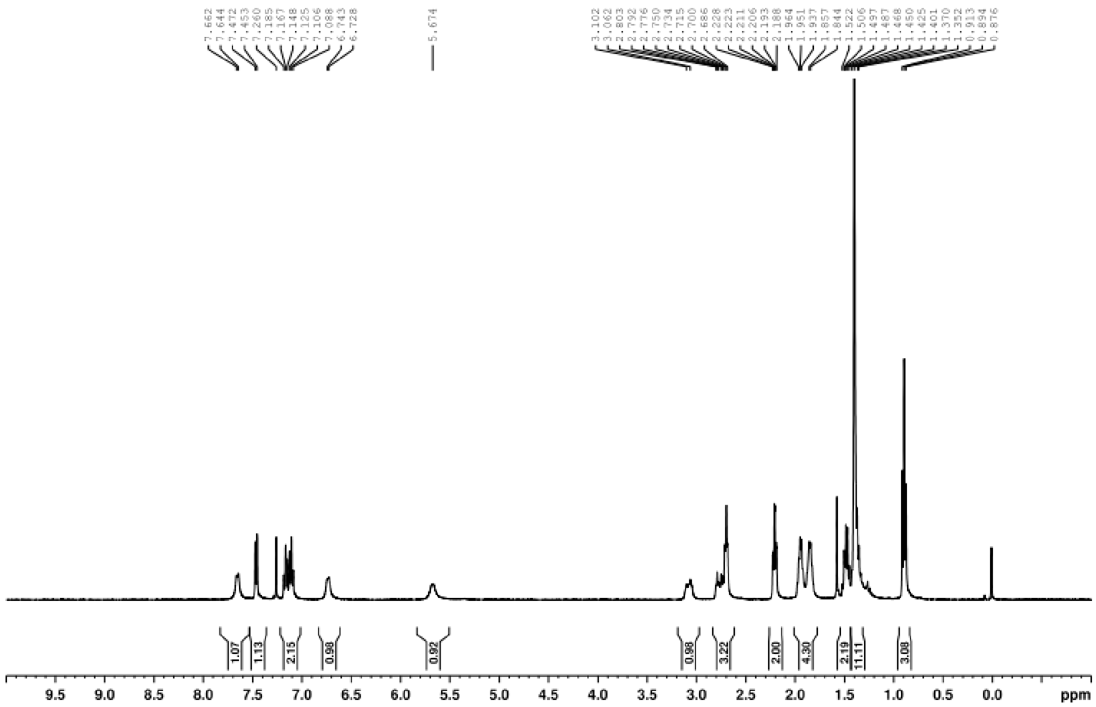

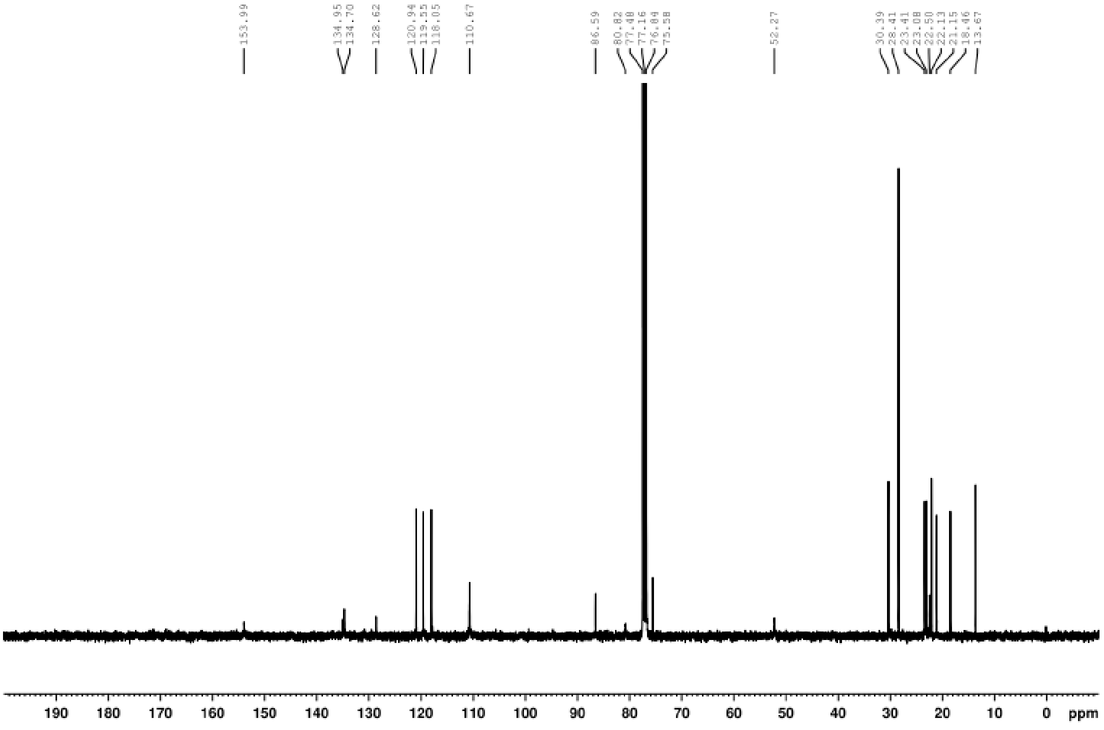

Supplementary Figure 58. ^{1}H and ^{13}C-NMR spectrum for **5u**

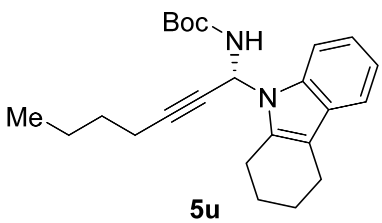

5u

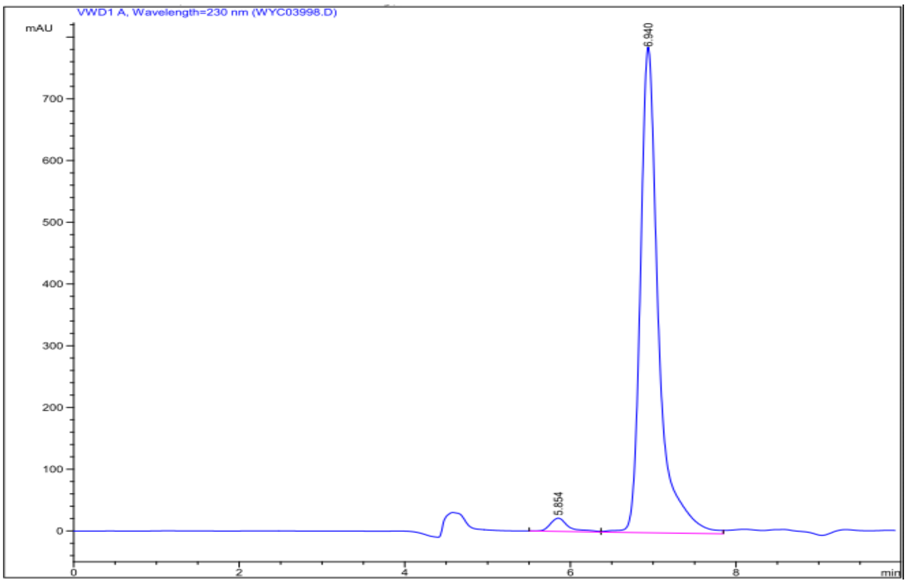

Peak #	RetTime [min]	Type	Width [min]	Area mAU *s	Height [mAU]	Area %
1	5.854	BV	0.2319	331.03537	21.51577	2.6950
2	6.940	VV	0.2268	1.19523e4	786.56360	97.3050

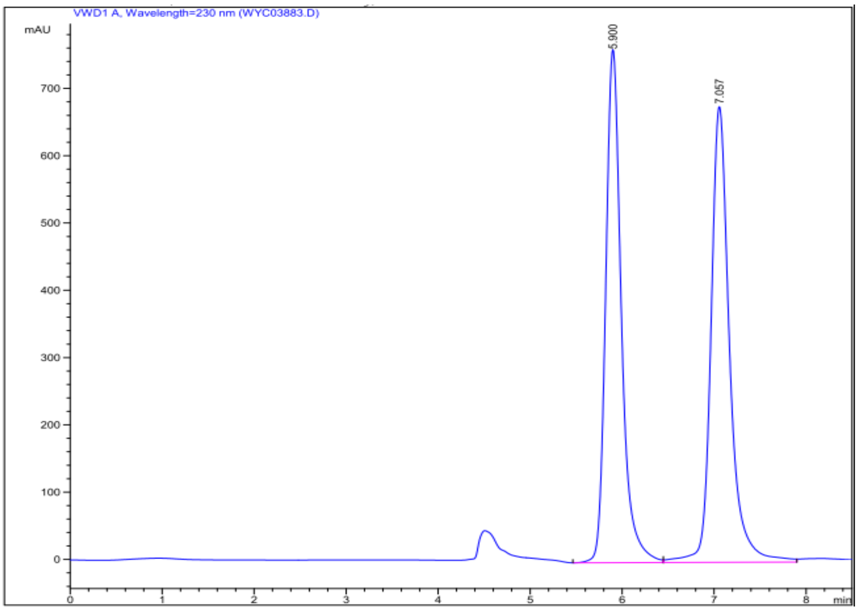

Peak #	RetTime [min]	Type	Width [min]	Area mAU *s	Height [mAU]	Area %
1	5.900	VV	0.1803	9023.76855	762.64667	48.6093
2	7.057	VV	0.2109	9540.09863	677.32208	51.3907

Supplementary Figure 59. HPLC spectrum for **5u**

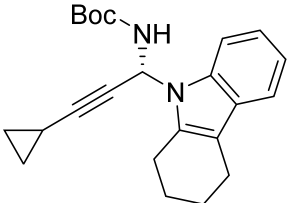

5v

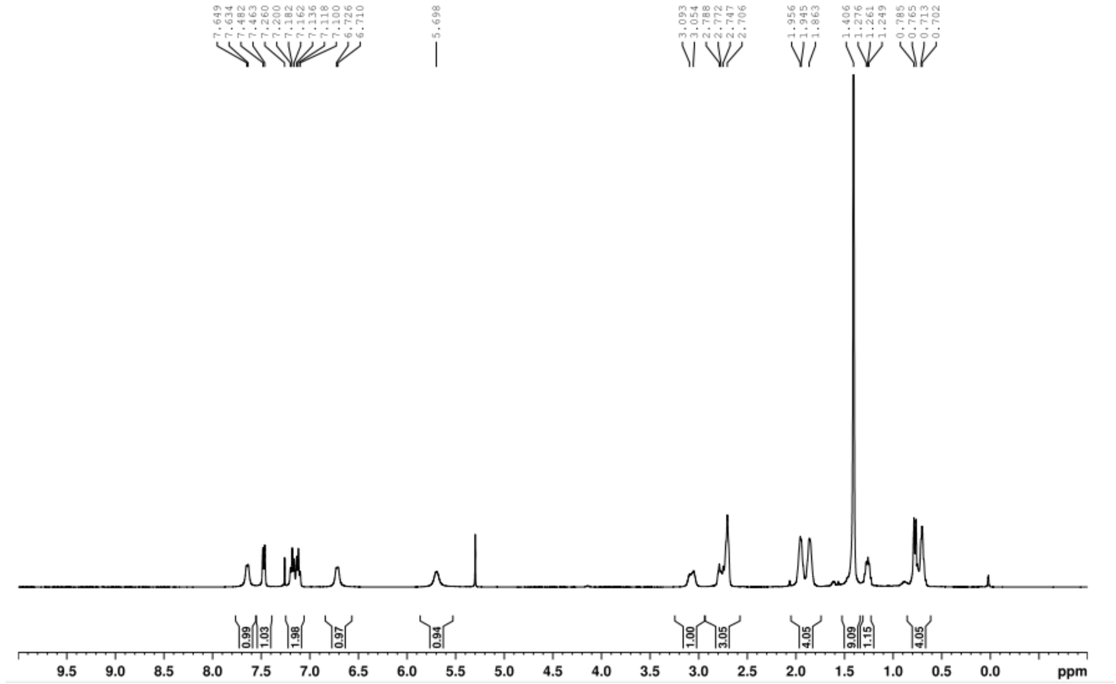

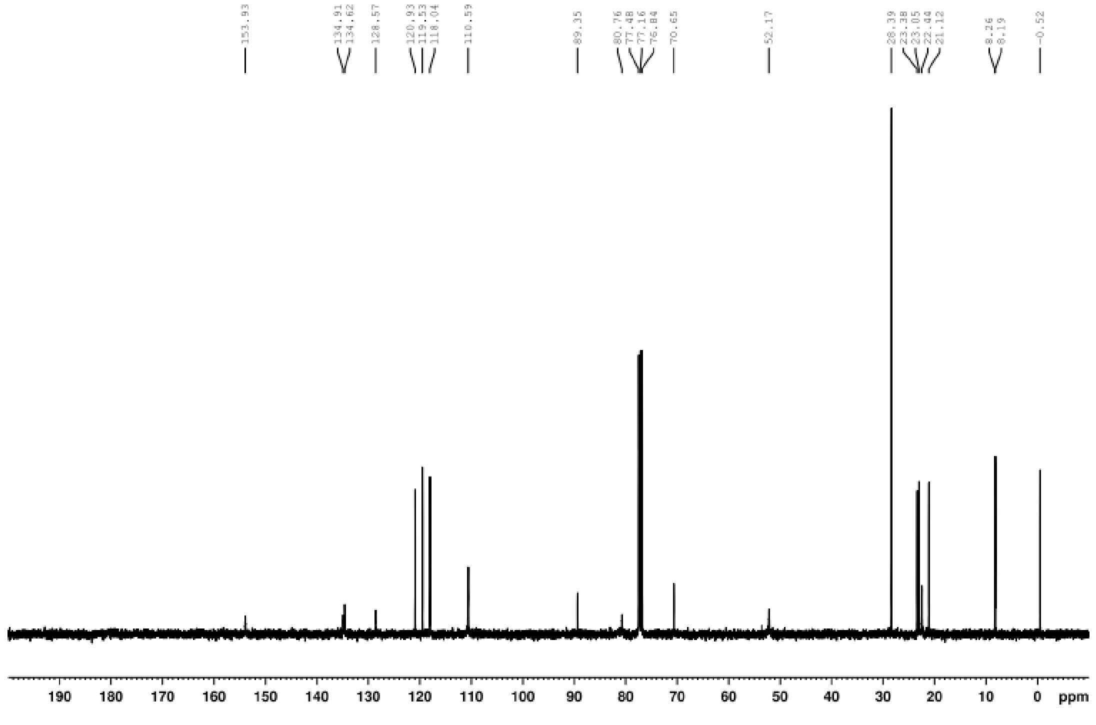

Supplementary Figure 60. ^{1}H and ^{13}C-NMR spectrum for **5v**

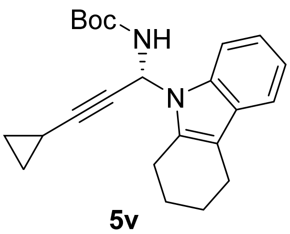

5v

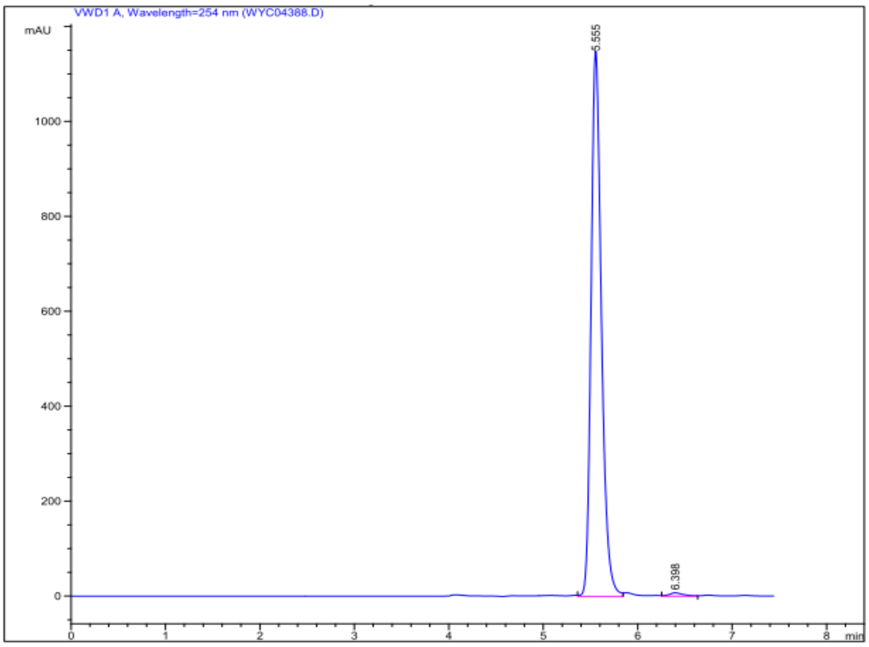

Peak #	RetTime [min]	Type	Width [min]	Area mAU *s	Height [mAU]	Area %
1	5.555	VV	0.1148	8723.49609	1149.24902	99.0869
2	6.398	VV	0.1607	80.39001	7.20511	0.9131

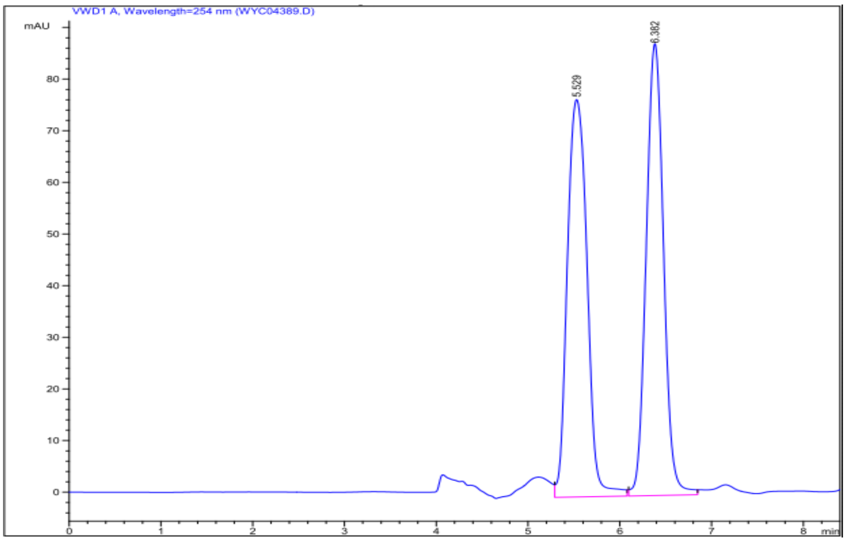

Peak #	RetTime [min]	Type	Width [min]	Area mAU *s	Height [mAU]	Area %
1	5.529	VB	0.2475	1152.42737	76.96123	50.0433
2	6.382	BB	0.2072	1150.43384	87.51048	49.9567

Supplementary Figure 61. HPLC spectrum for **5v**

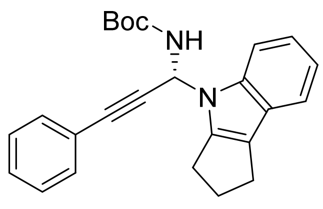

5w

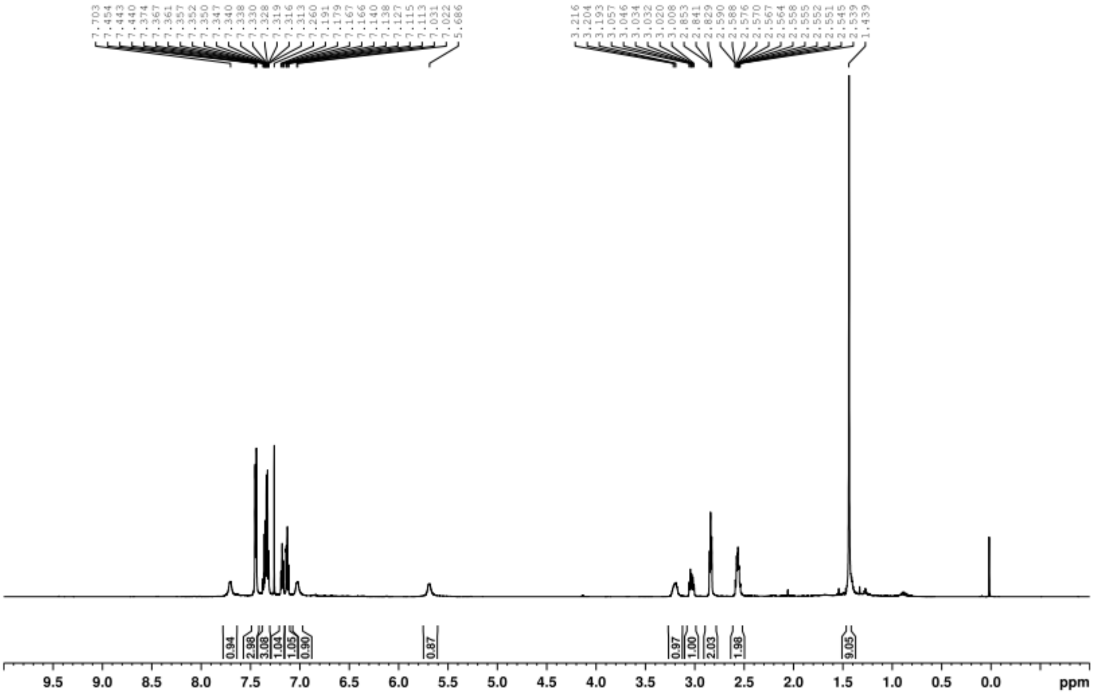

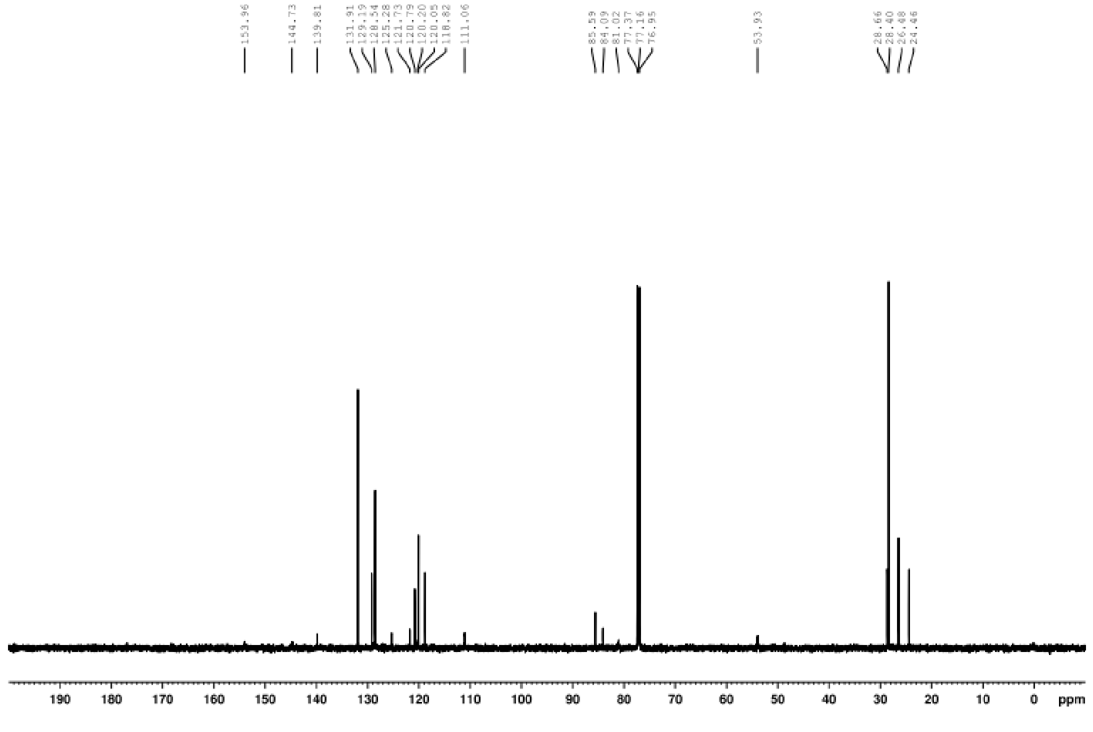

Supplementary Figure 62. ^{1}H and ^{13}C-NMR spectrum for **5w**

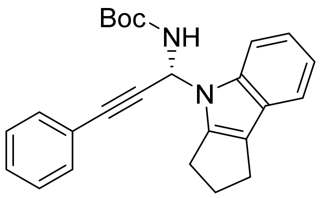

5w

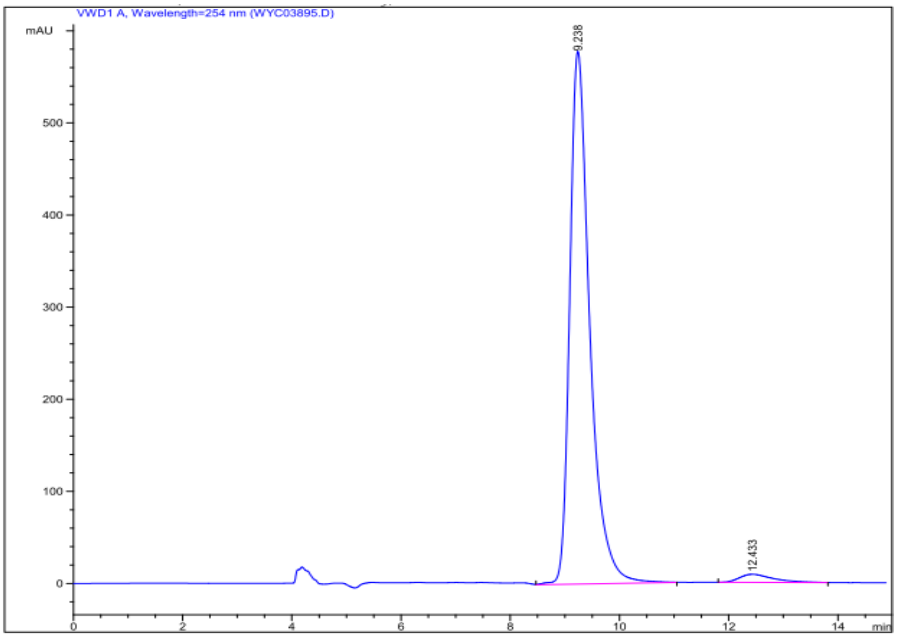

Peak #	RetTime [min]	Type	Width [min]	Area mAU *s	Height [mAU]	Area %
1	9.238	VB	0.3860	1.47288e4	578.25647	97.4947
2	12.433	BB	0.6261	378.48087	8.79047	2.5053

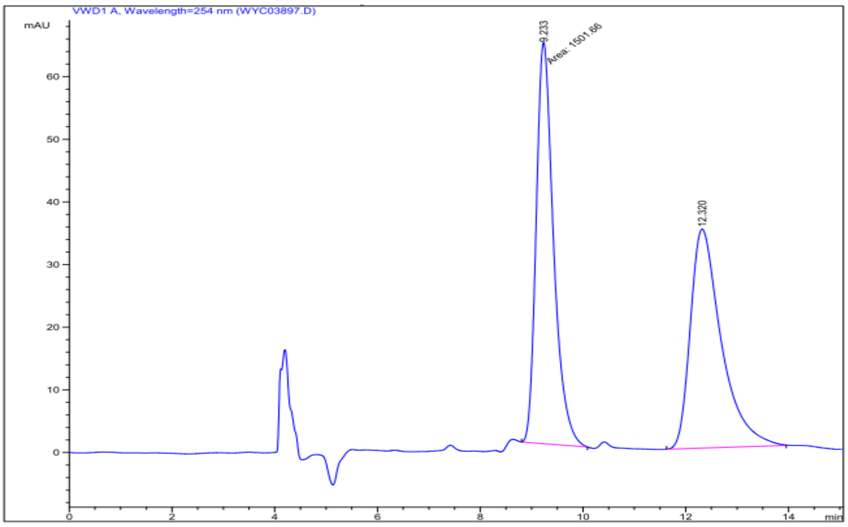

Peak #	RetTime [min]	Type	Width [min]	Area mAU *s	Height [mAU]	Area %
1	9.233	MM	0.3908	1501.66382	64.04678	50.5956
2	12.320	BB	0.6261	1466.30957	34.96953	49.4044

Supplementary Figure 63. HPLC spectrum for **5w**

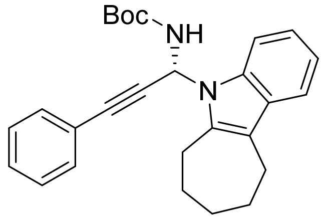

5x

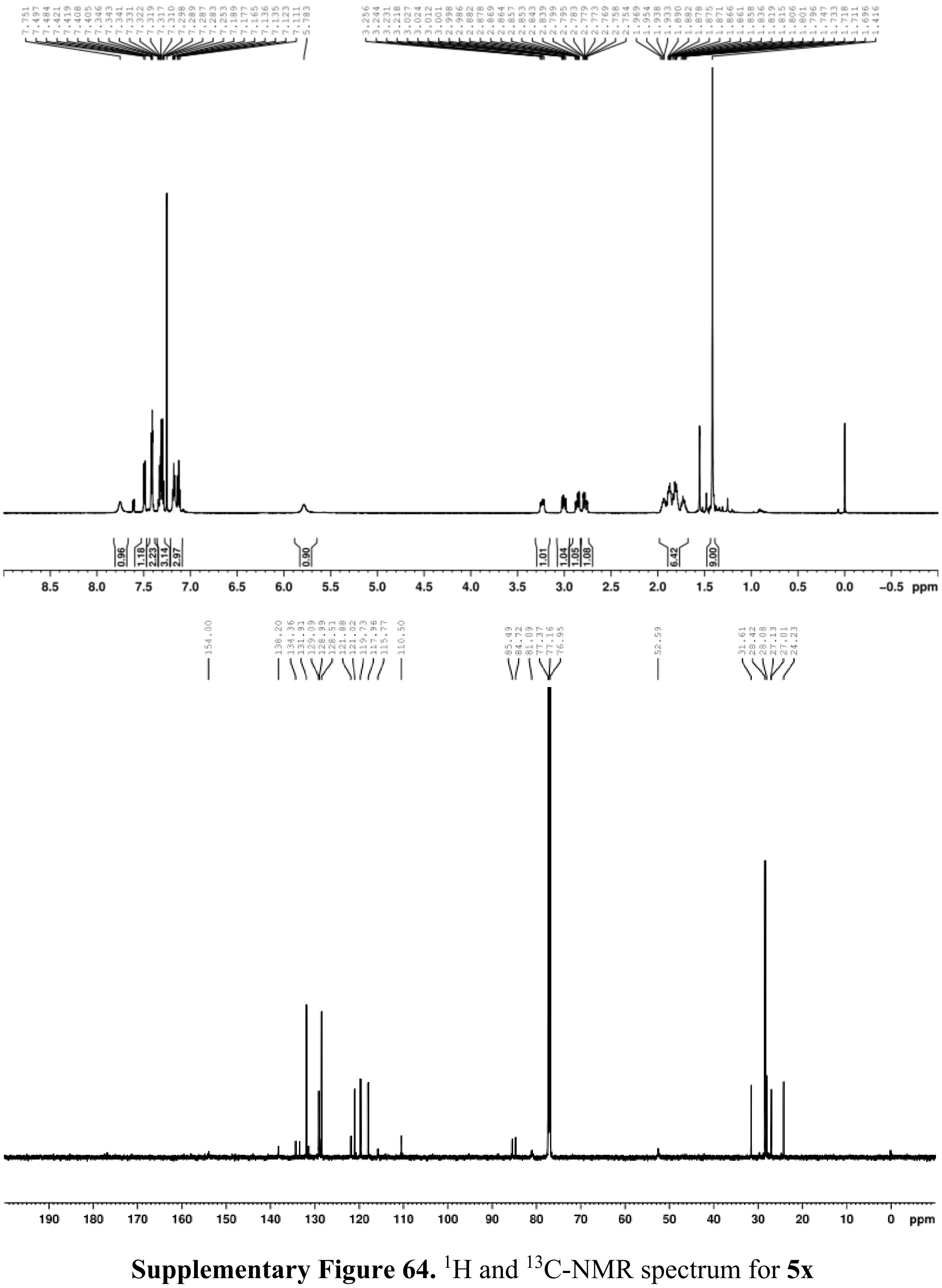

Supplementary Figure 64. ^{1}H and ^{13}C-NMR spectrum for **5x**

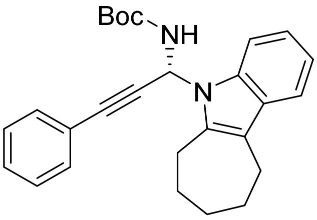

5x

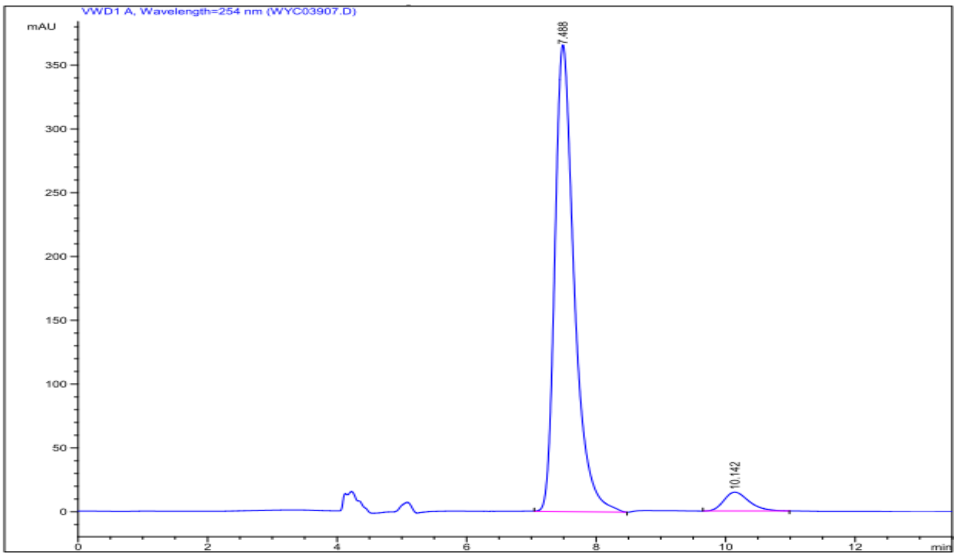

Peak #	RetTime [min]	Type	Width [min]	Area mAU *s	Height [mAU]	Area %
1	7.488	BP	0.3150	7592.78223	365.77686	95.0004
2	10.142	PB	0.4140	399.58841	14.79951	4.9996

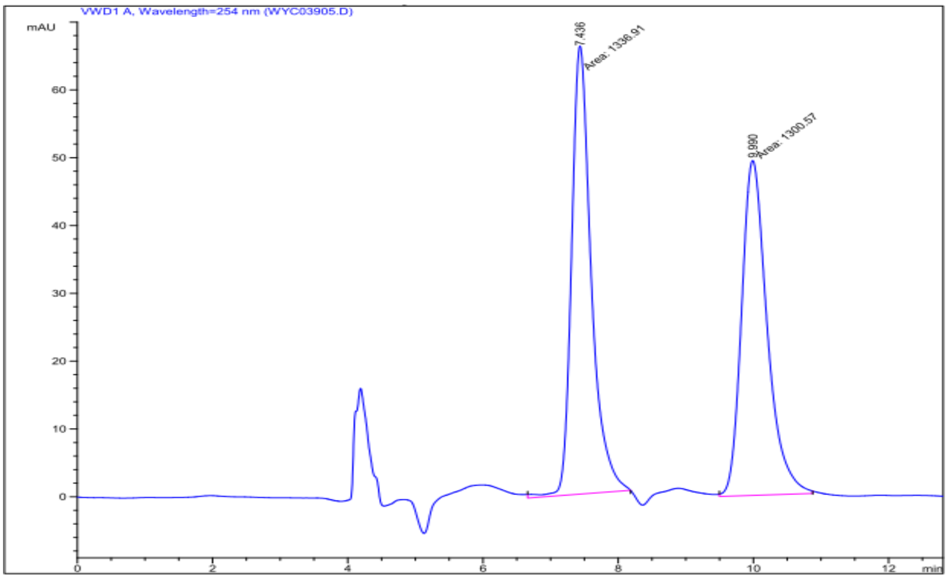

Peak #	RetTime [min]	Type	Width [min]	Area mAU *s	Height [mAU]	Area %
1	7.436	MM	0.3374	1336.91162	66.04803	50.6889
2	9.990	MM	0.4389	1300.57385	49.38469	49.3111

Supplementary Figure 65. HPLC spectrum for **5x**

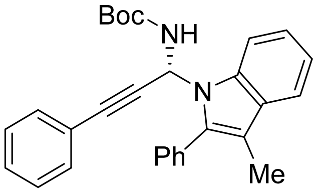

5y

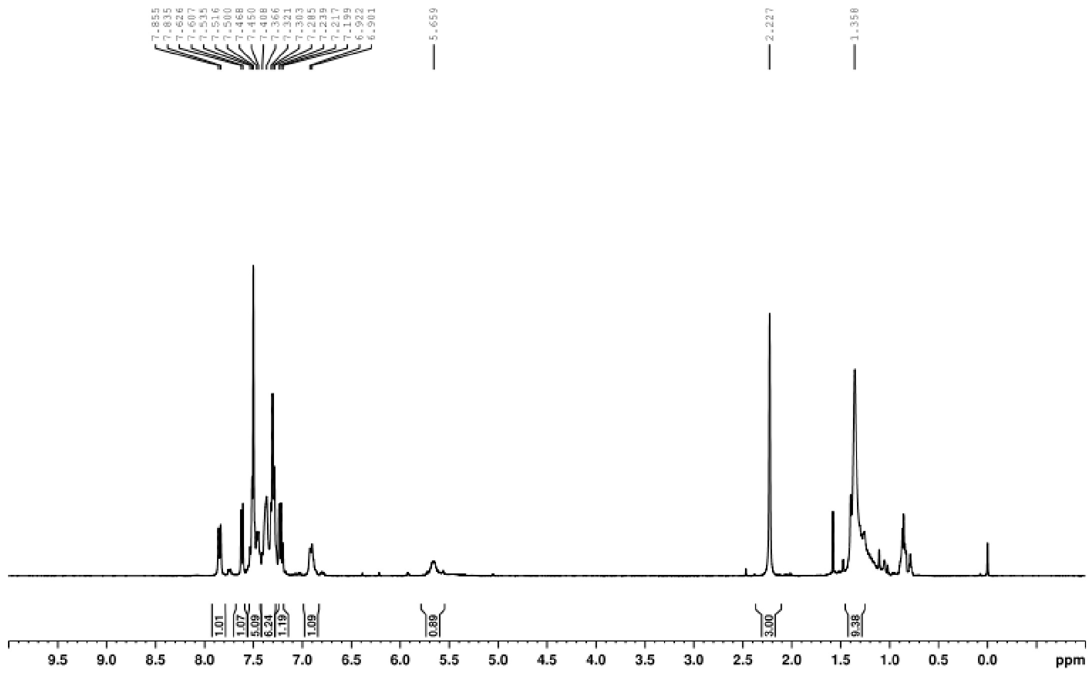

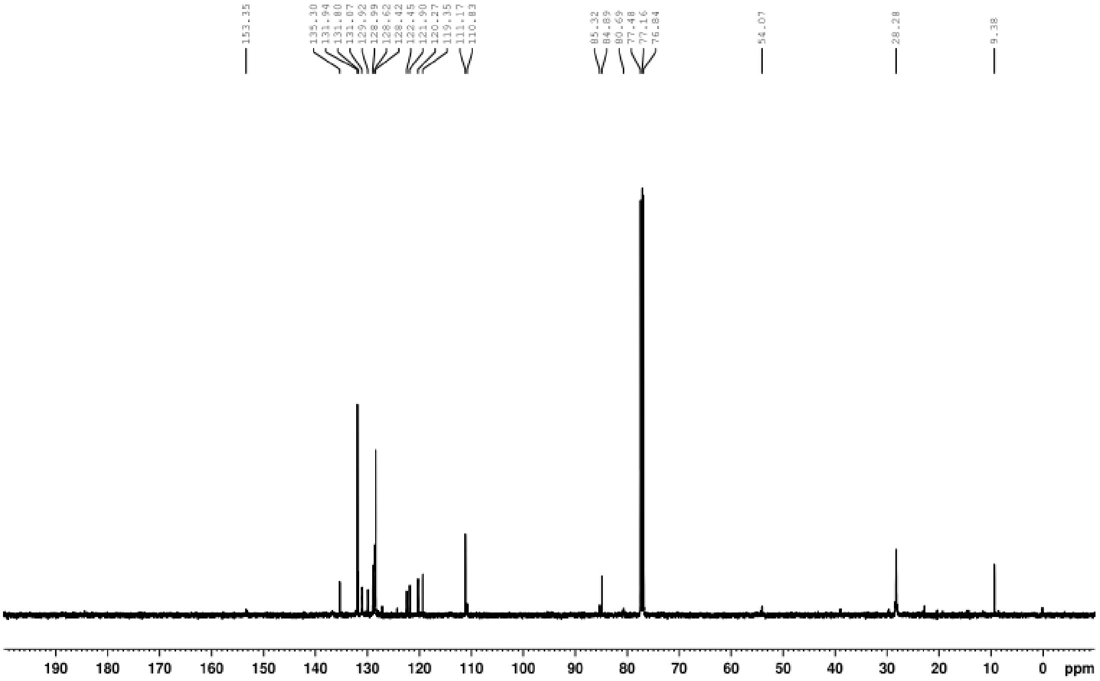

Supplementary Figure 66. ^{1}H and ^{13}C-NMR spectrum for **5y**

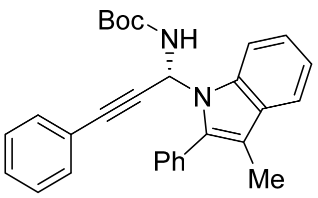

5y

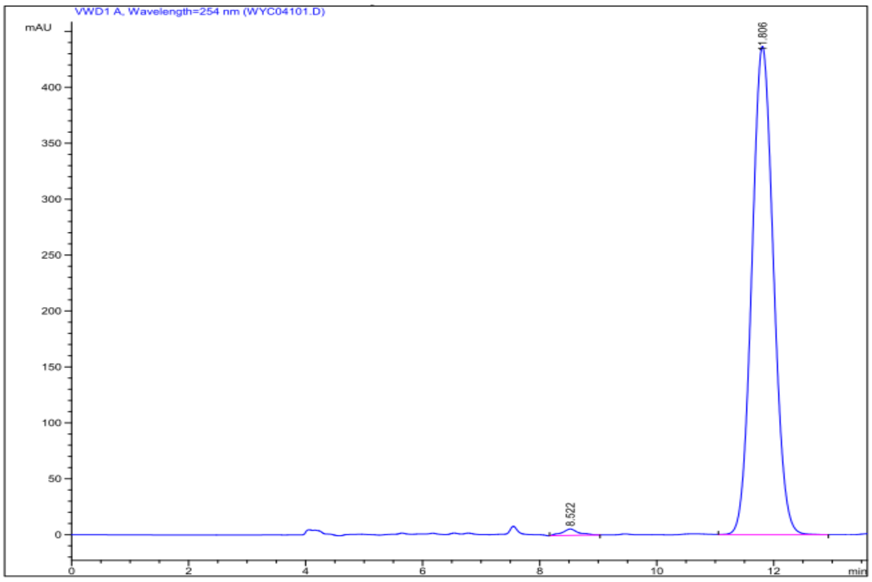

Peak #	RetTime [min]	Type	Width [min]	Area mAU *s	Height [mAU]	Area %
1	8.522	PB	0.2745	113.03307	5.78732	0.9997
2	11.806	VP	0.4010	1.11935e4	436.60129	99.0003

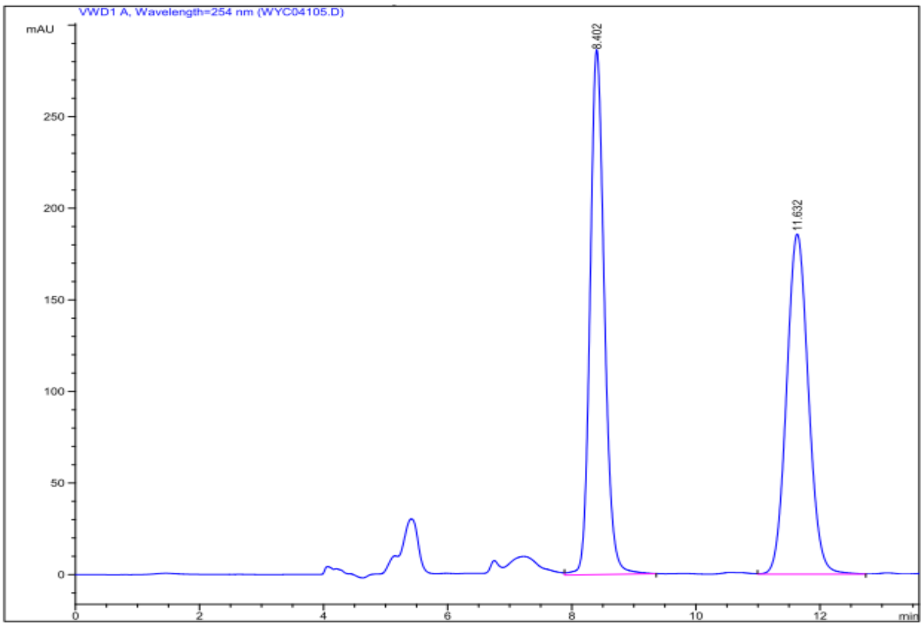

Peak #	RetTime [min]	Type	Width [min]	Area mAU *s	Height [mAU]	Area %
1	8.402	VB	0.2454	4597.77002	286.53555	50.3661
2	11.632	VB	0.3822	4530.92334	185.69521	49.6339

Supplementary Figure 67. HPLC spectrum for **5y**

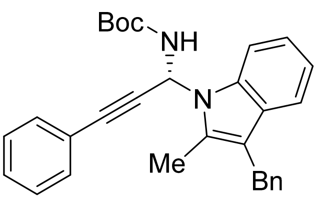

5z

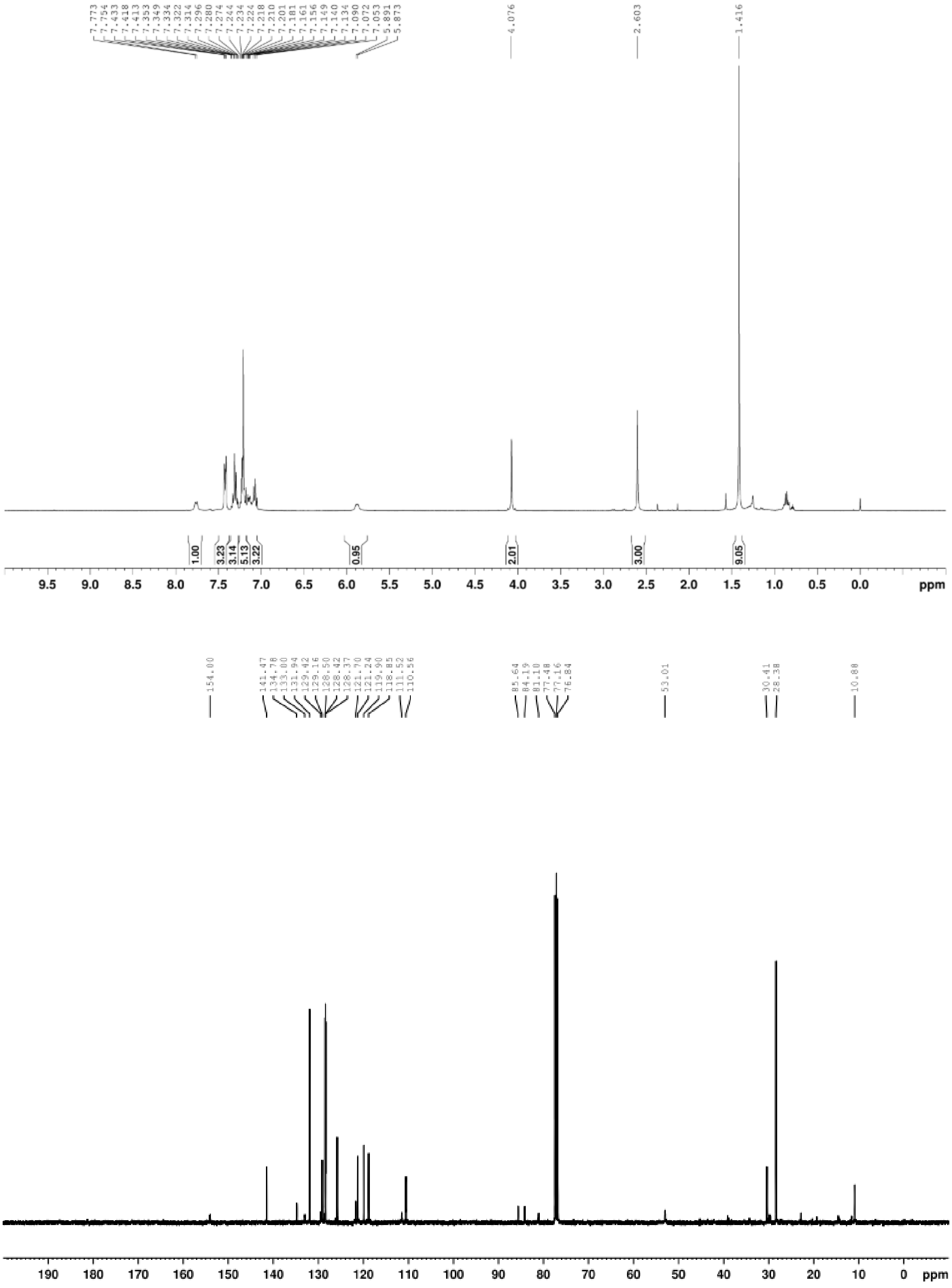

Supplementary Figure 68. ^{1}H and ^{13}C-NMR spectrum for **5z**

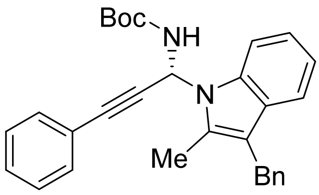

5z

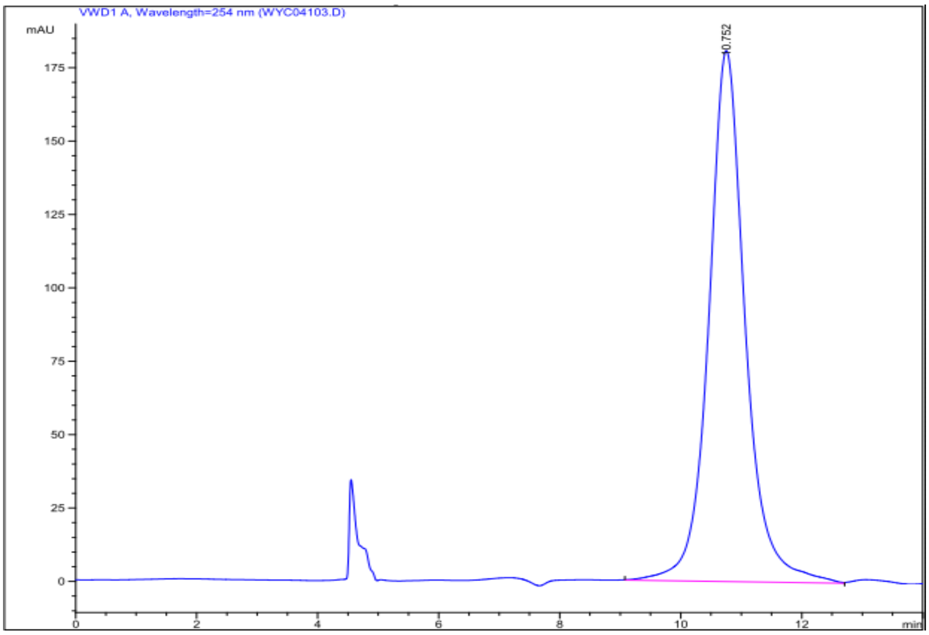

Peak #	RetTime [min]	Type	Width [min]	Area mAU *s	Height [mAU]	Area %
1	10.752	BB	0.6203	7586.36816	180.89452	100.0000

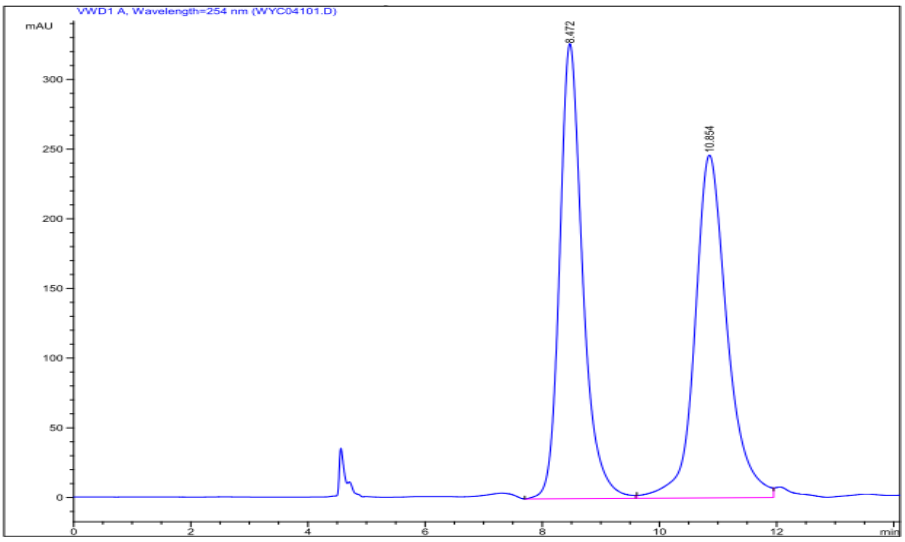

Peak #	RetTime [min]	Type	Width [min]	Area mAU *s	Height [mAU]	Area %
1	8.472	VB	0.4121	8924.71973	326.46597	48.6577
2	10.854	BV	0.5733	9417.11914	245.99792	51.3423

Supplementary Figure 69. HPLC spectrum for **5z**

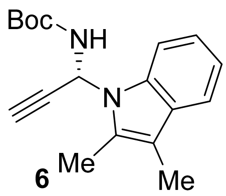

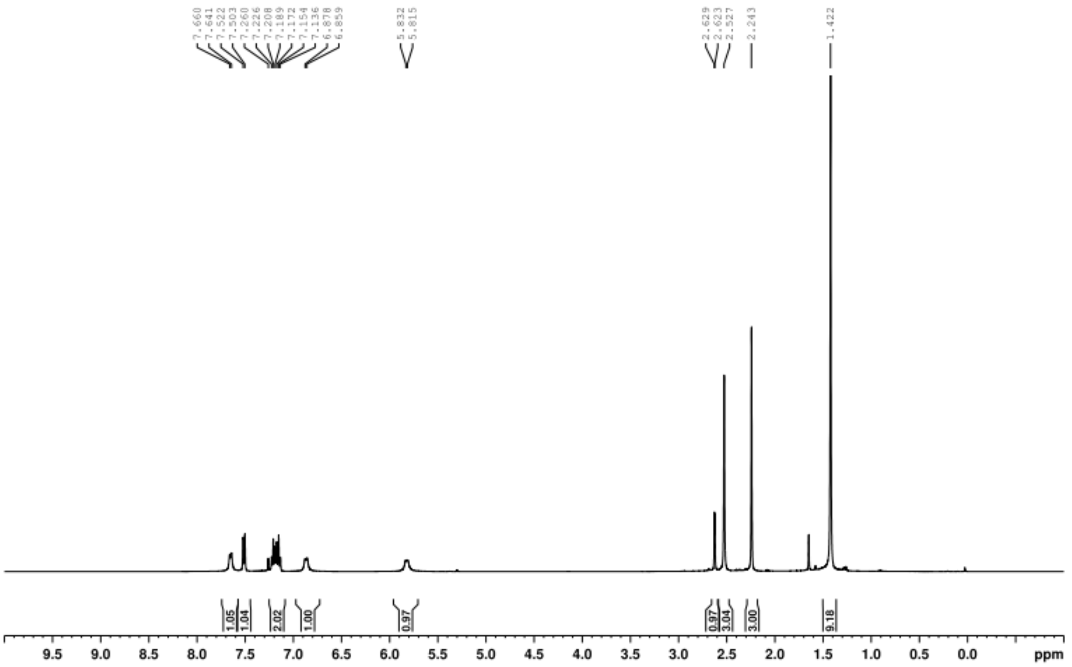

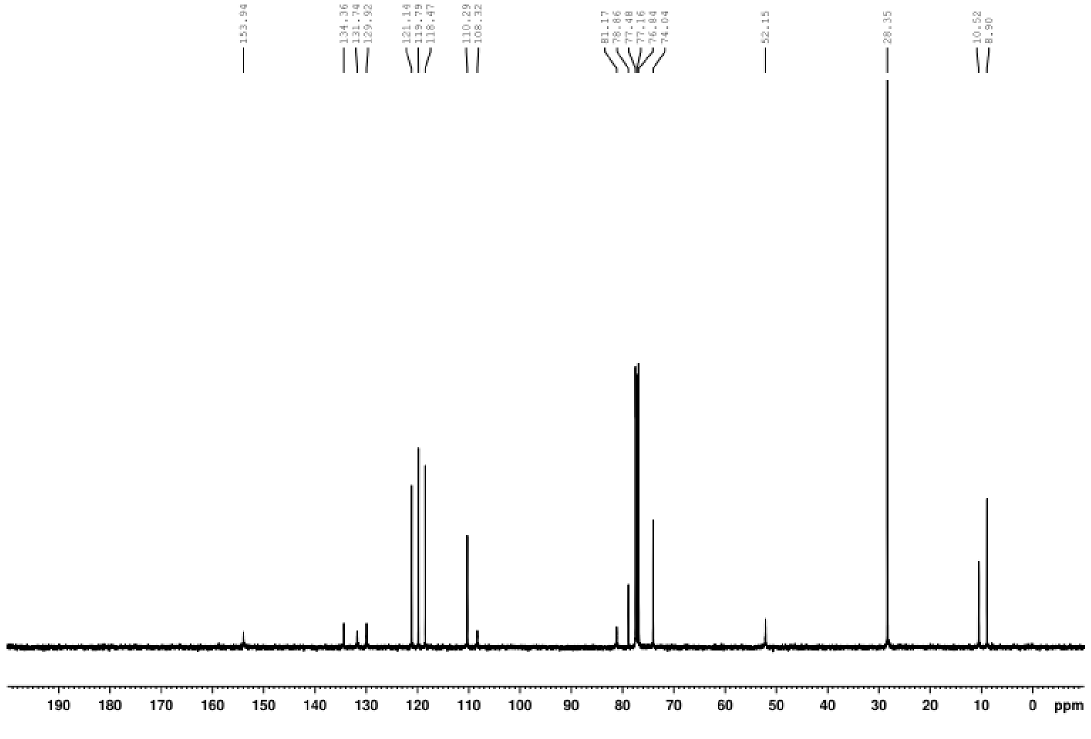

Supplementary Figure 70. ^{1}H and ^{13}C-NMR spectrum for **6**

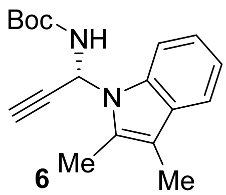

6

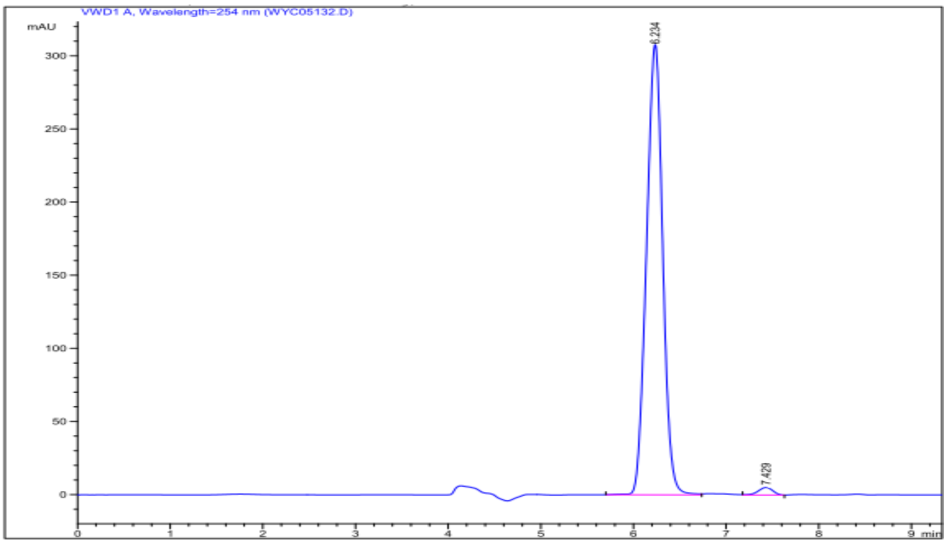

Peak #	RetTime [min]	Type	Width [min]	Area mAU *s	Height [mAU]	Area %
1	6.234	BB	0.1871	3748.53613	307.97827	98.5301
2	7.429	VP	0.1716	55.92291	5.15913	1.4699

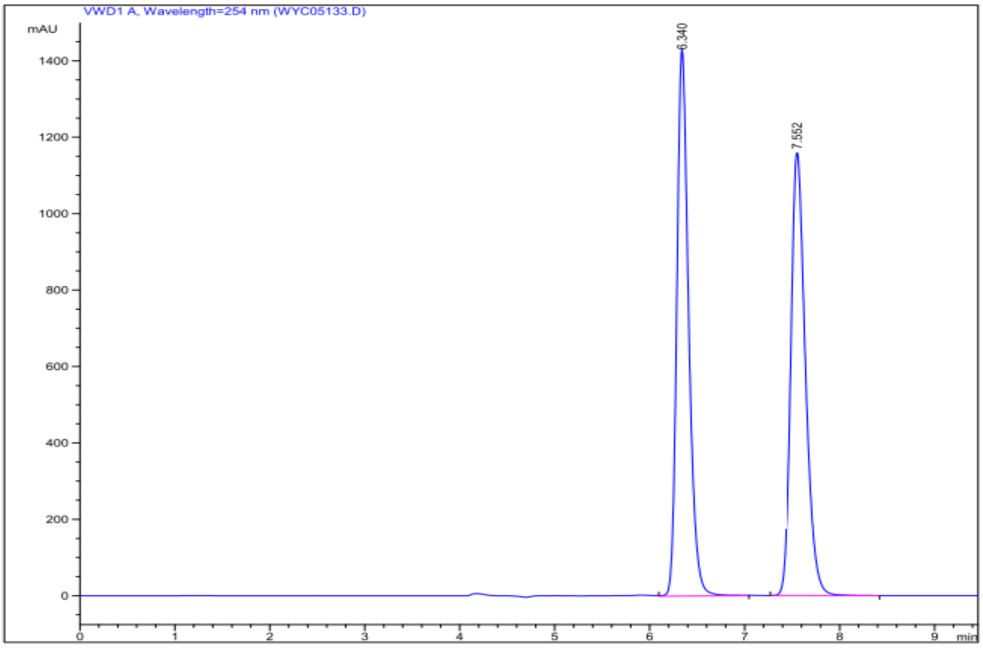

Peak #	RetTime [min]	Type	Width [min]	Area mAU *s	Height [mAU]	Area %
1	6.340	VB	0.1315	1.23622e4	1429.32397	50.0844
2	7.552	PP	0.1617	1.23205e4	1160.86926	49.9156

Supplementary Figure 71. HPLC spectrum for **6**

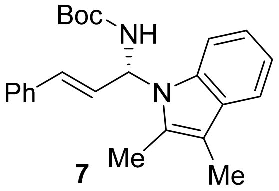

7

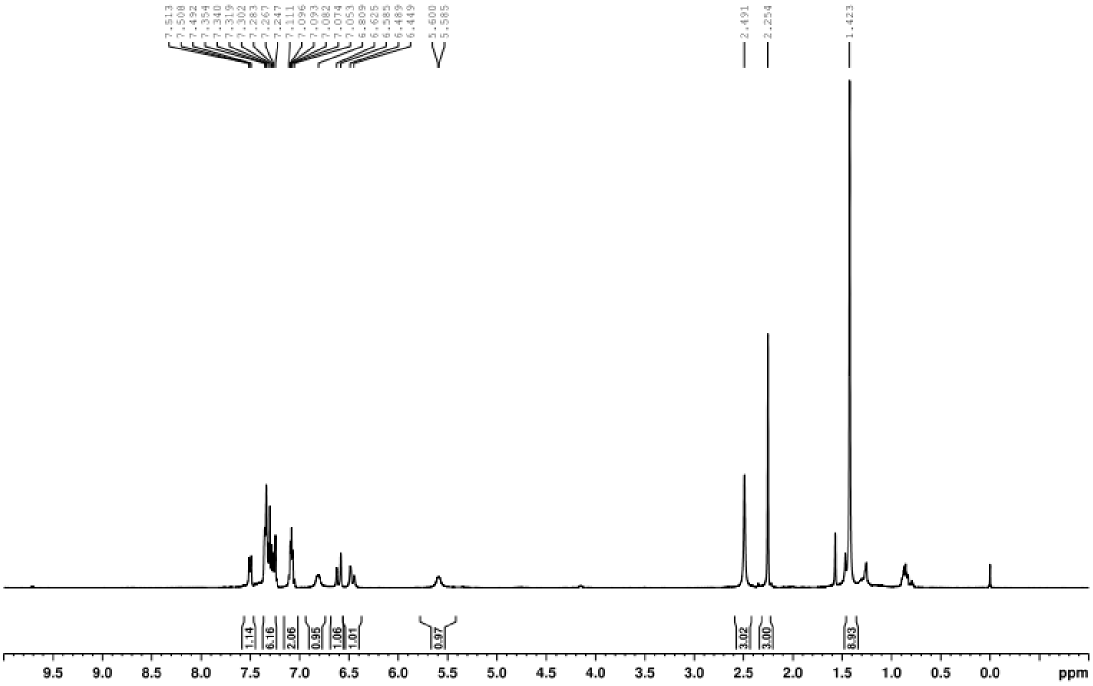

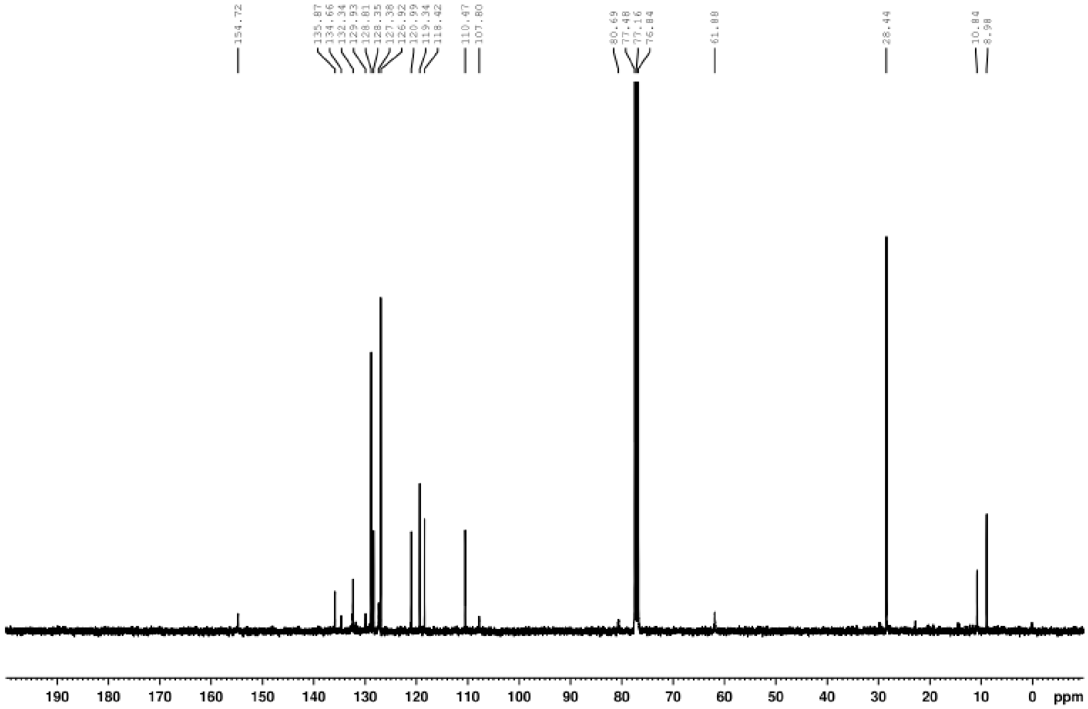

Supplementary Figure 72. ^{1}H and ^{13}C-NMR spectrum for **7**

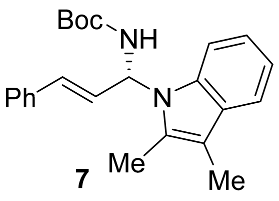

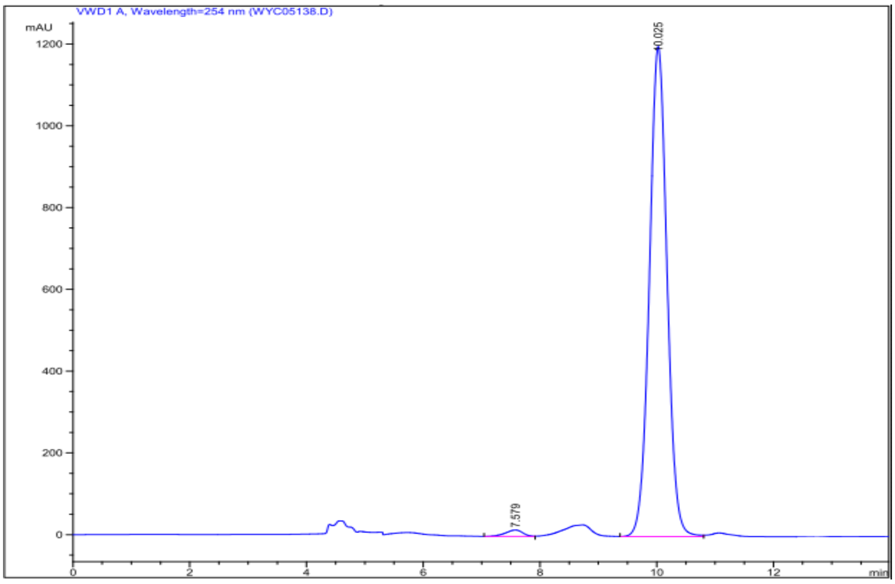

Peak #	RetTime [min]	Type	Width [min]	Area mAU *s	Height [mAU]	Area %
1	7.579	VV	0.3181	329.20618	15.75072	1.2926
2	10.025	VV	0.3233	2.51396e4	1198.80603	98.7074

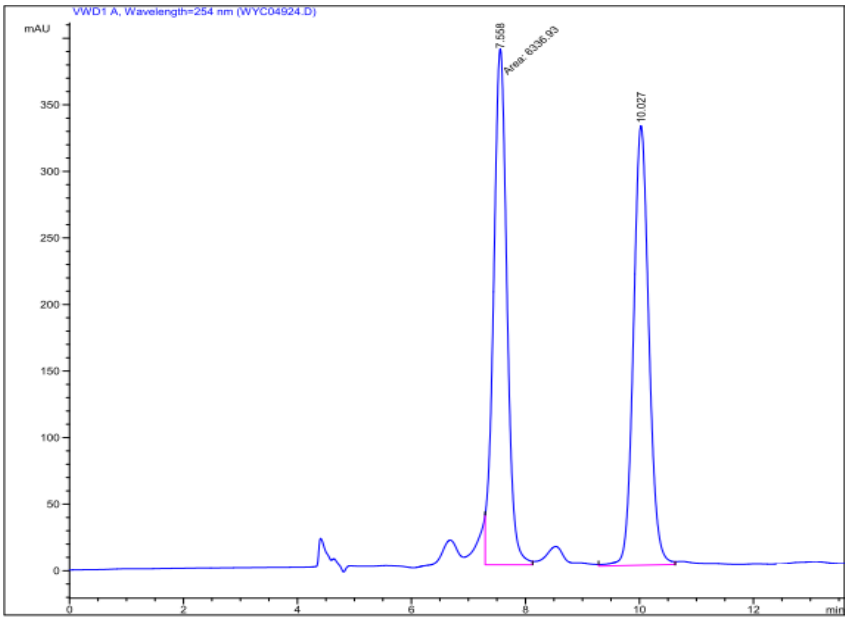

Peak #	RetTime [min]	Type	Width [min]	Area mAU *s	Height [mAU]	Area %
1	7.558	MM	0.2725	6336.93262	387.57632	50.5533
2	10.027	VV	0.2904	6198.22217	330.07959	49.4467

Supplementary Figure 73. HPLC spectrum for **7**

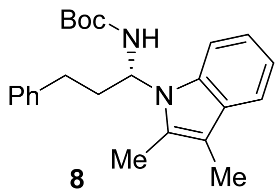

8

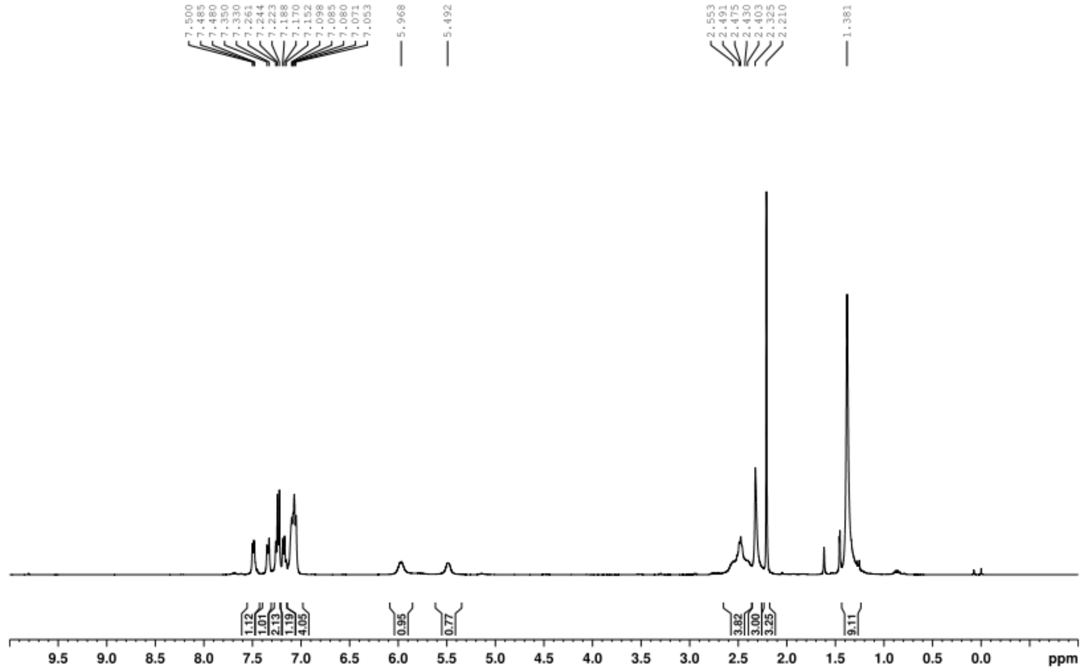

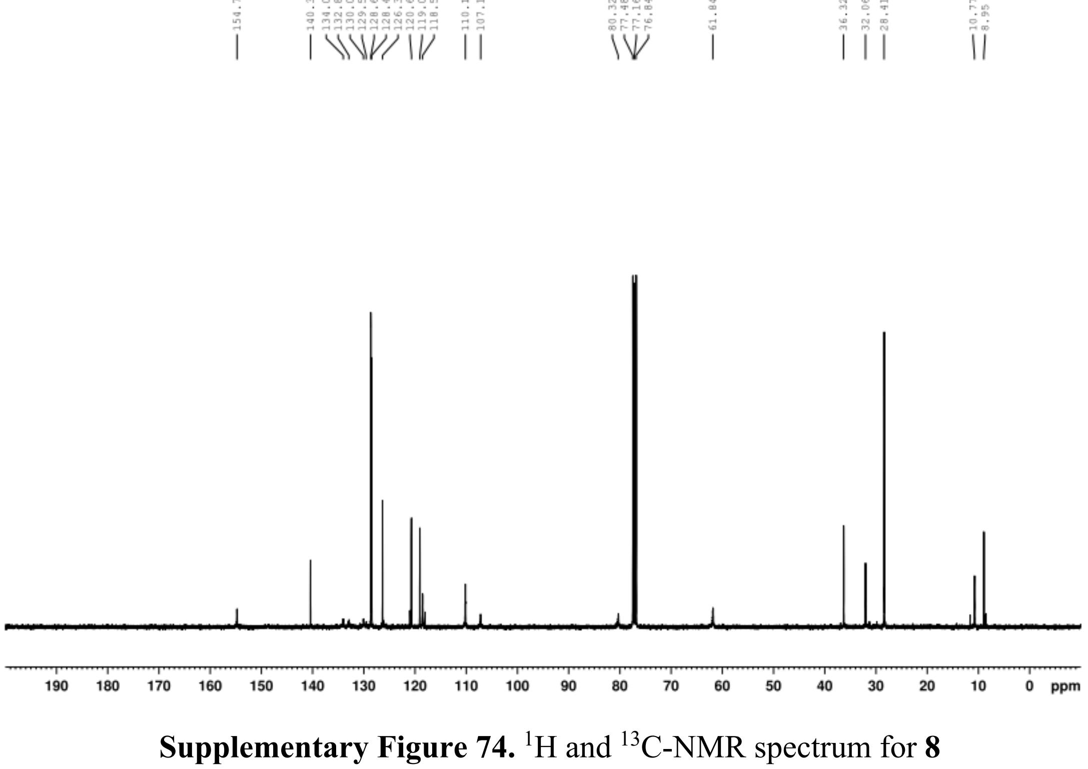

Supplementary Figure 74. ^{1}H and ^{13}C-NMR spectrum for **8**

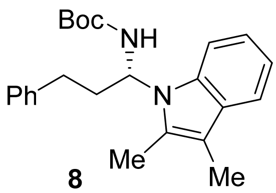

8

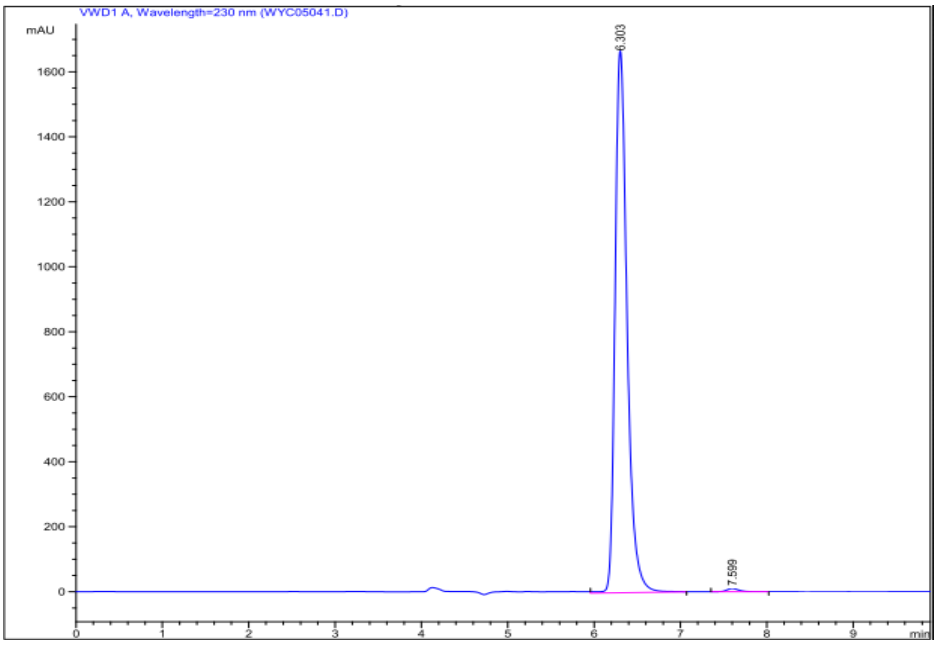

Peak #	RetTime [min]	Type	Width [min]	Area mAU *s	Height [mAU]	Area %
1	6.303	VB	0.1494	1.61497e4	1666.94263	99.3918
2	7.599	PP	0.1529	98.82895	9.89788	0.6082

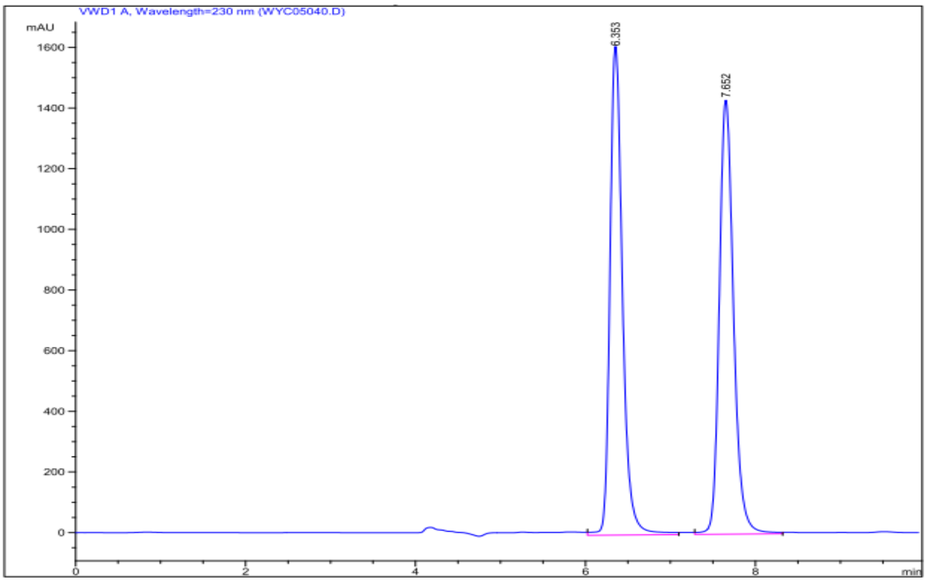

Peak #	RetTime [min]	Type	Width [min]	Area mAU *s	Height [mAU]	Area %
1	6.353	VB	0.1577	1.65555e4	1611.44824	49.7384
2	7.652	BV	0.1800	1.67297e4	1431.68542	50.2616

Supplementary Figure 75. HPLC spectrum for **8**

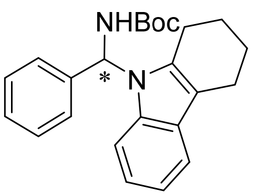

10a

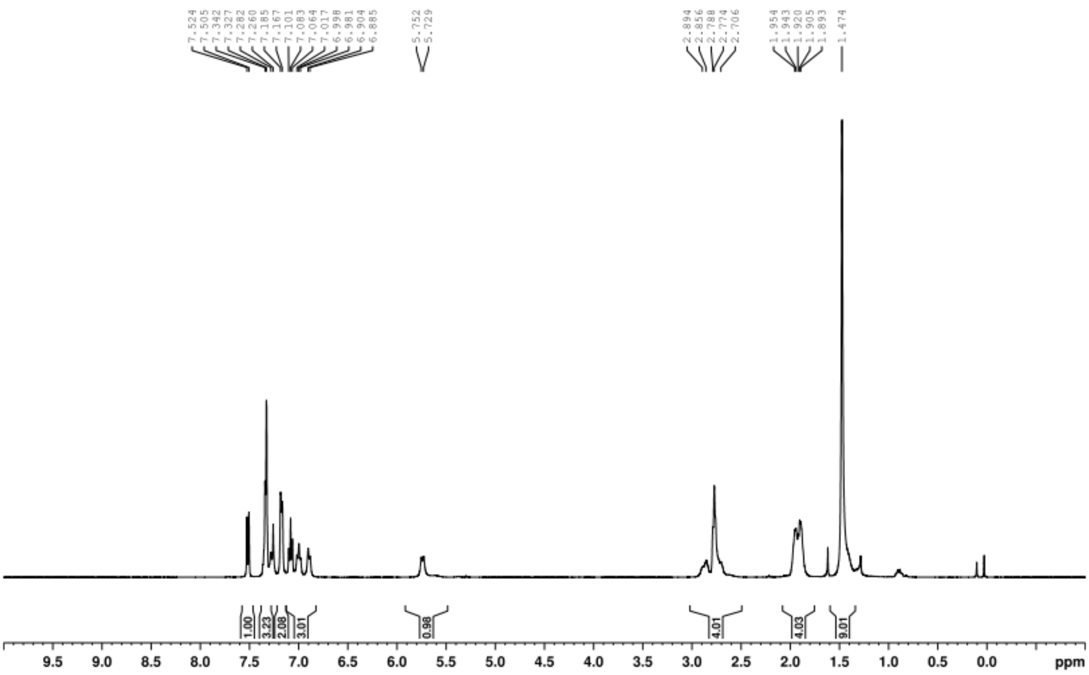

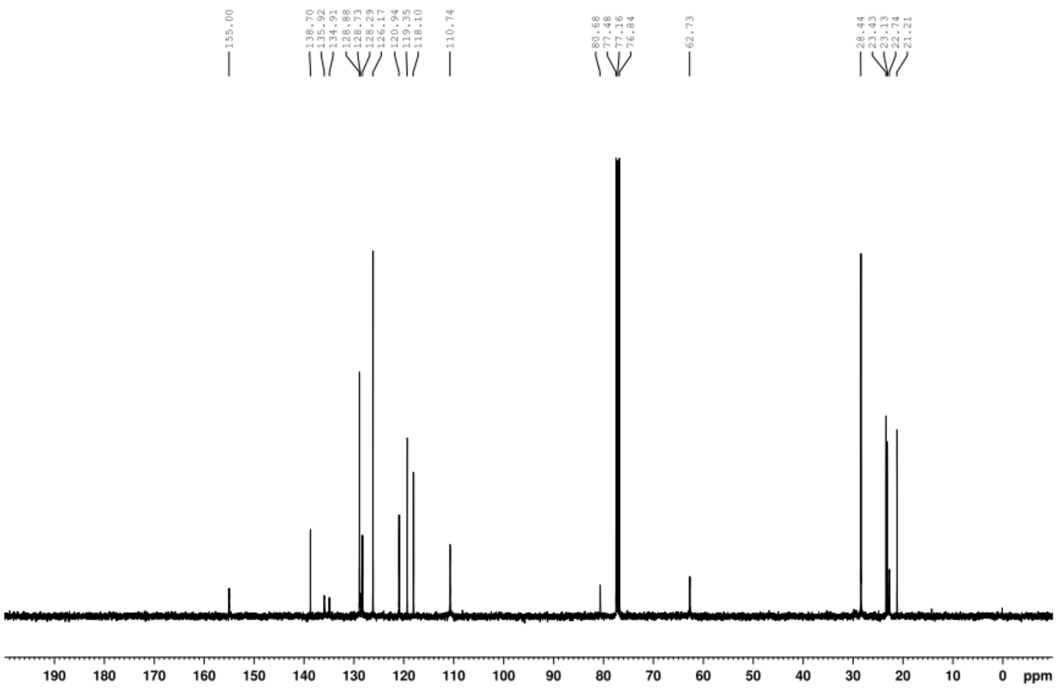

Supplementary Figure 76. ^{1}H and ^{13}C-NMR spectrum for **10a**

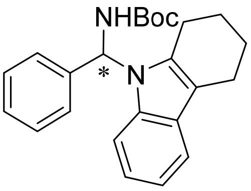

10a

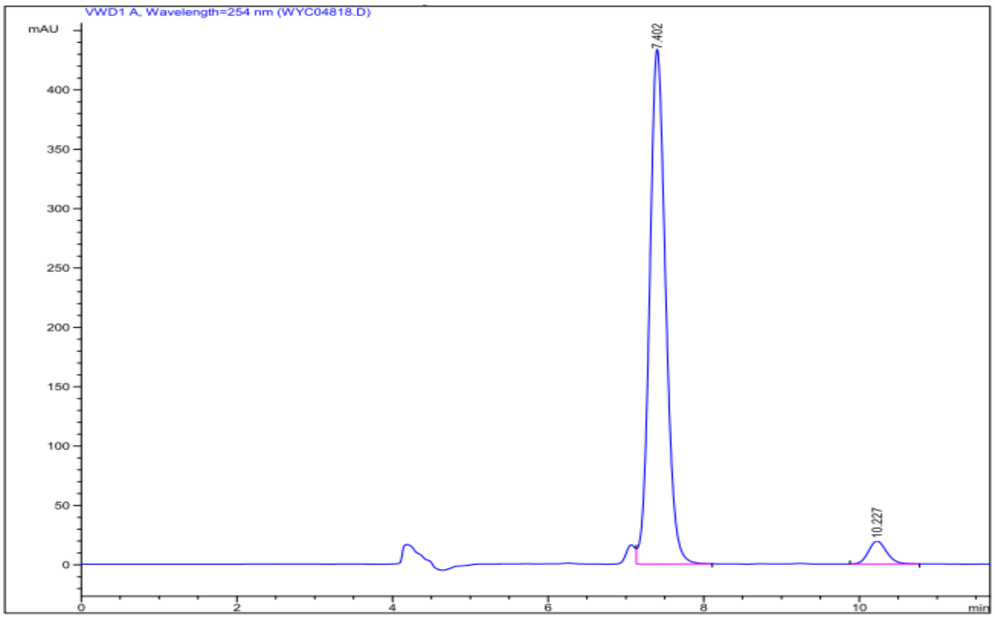

Peak #	RetTime [min]	Type	Width [min]	Area mAU *s	Height [mAU]	Area %
1	7.402	VB	0.2190	6188.24512	433.48233	95.1775
2	10.227	BB	0.2521	313.54861	19.30827	4.8225

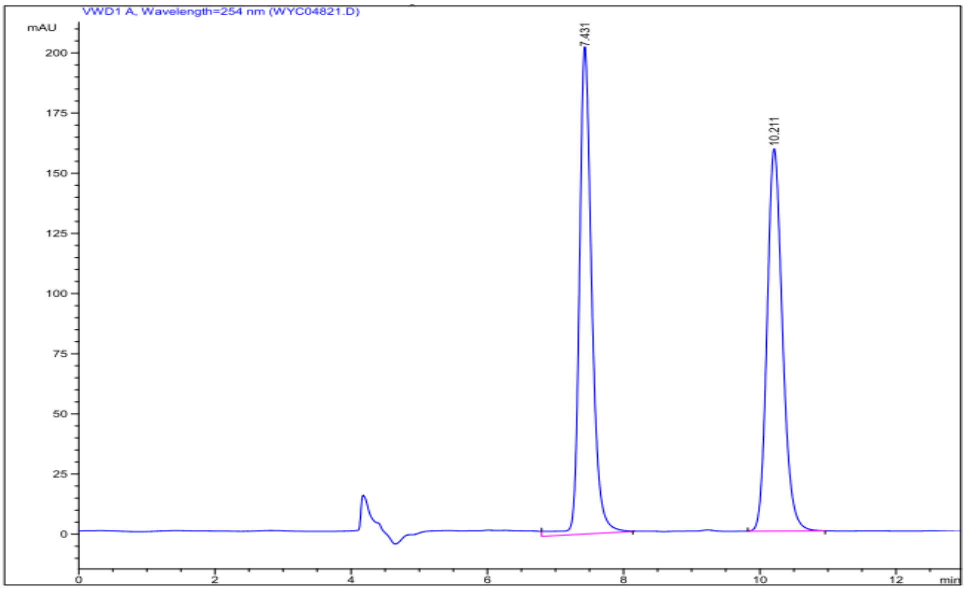

Peak #	RetTime [min]	Type	Width [min]	Area mAU *s	Height [mAU]	Area %
1	7.431	VB	0.1915	2564.81250	202.42896	50.6237
2	10.211	BB	0.2434	2501.61401	158.89964	49.3763

Supplementary Figure 77. HPLC spectrum for **10a**

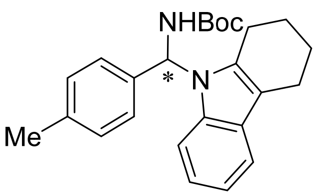

10b

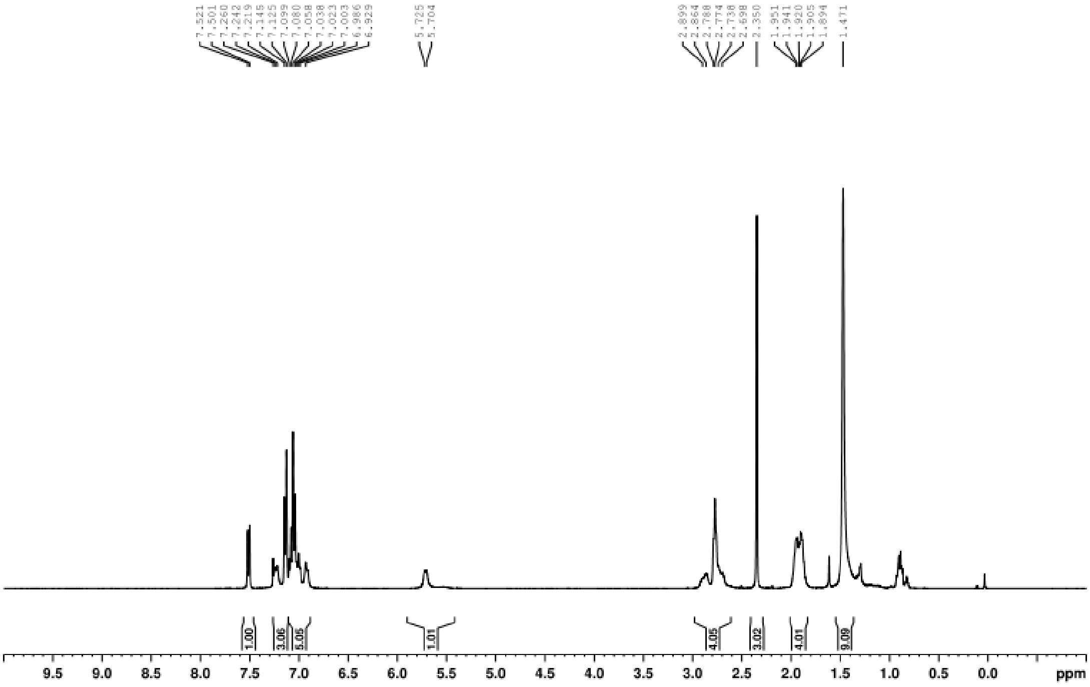

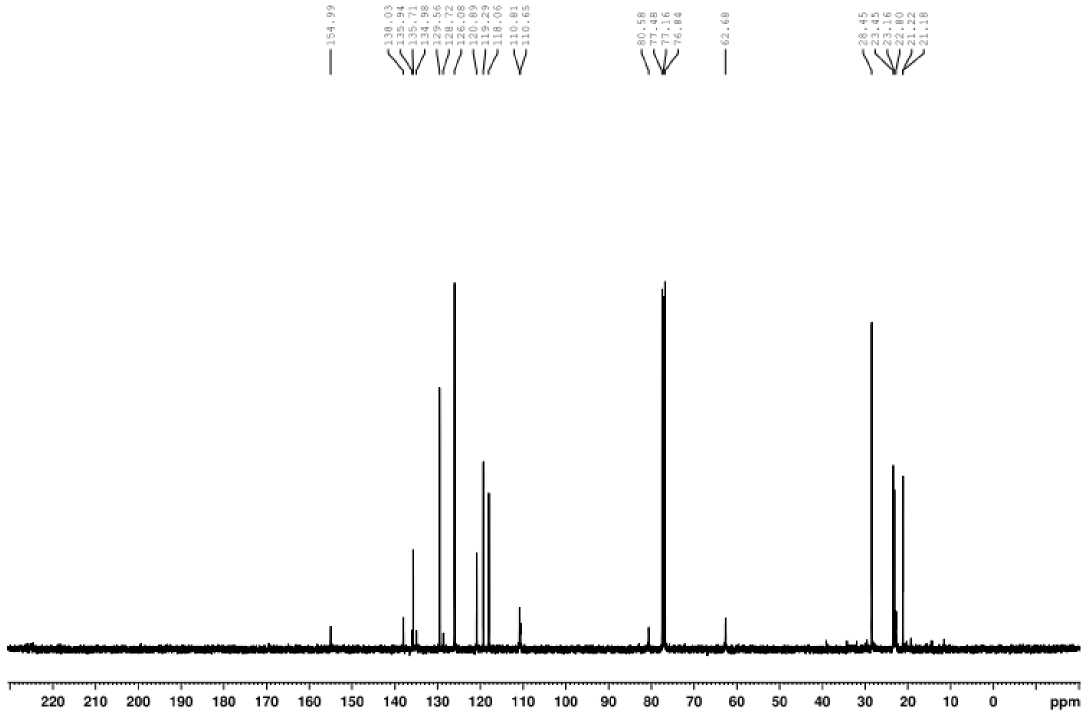

Supplementary Figure 78. ^{1}H and ^{13}C-NMR spectrum for **10b**

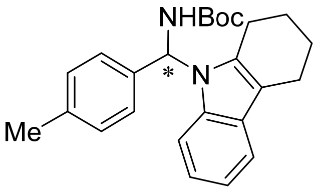

10b

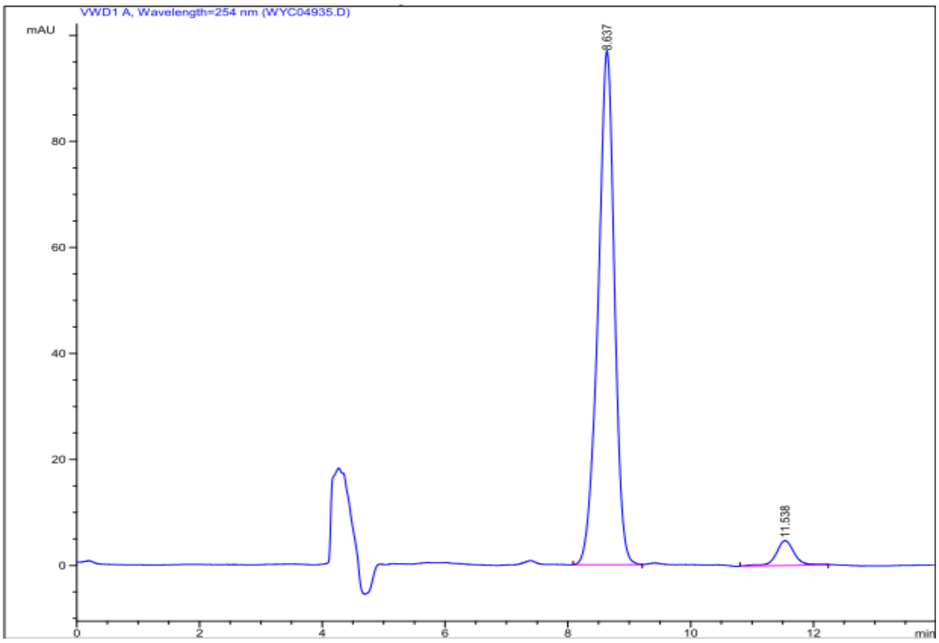

Peak #	RetTime [min]	Type	Width [min]	Area mAU *s	Height [mAU]	Area %
1	8.637	PB	0.2761	1798.35498	96.93125	94.9092
2	11.538	PB	0.3097	96.46175	4.69258	5.0908

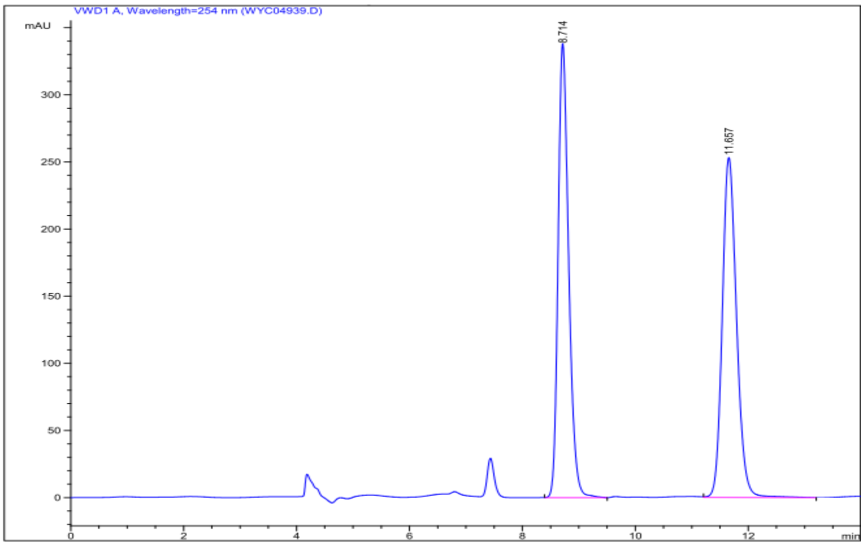

Peak #	RetTime [min]	Type	Width [min]	Area mAU *s	Height [mAU]	Area %
1	8.714	BP	0.1995	4391.50732	338.26035	49.7182
2	11.657	VP	0.2687	4441.28271	253.17355	50.2818

Supplementary Figure 79. HPLC spectrum for **10b**

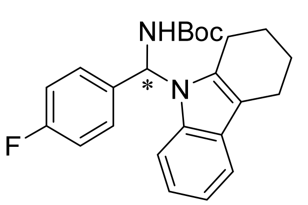

10c

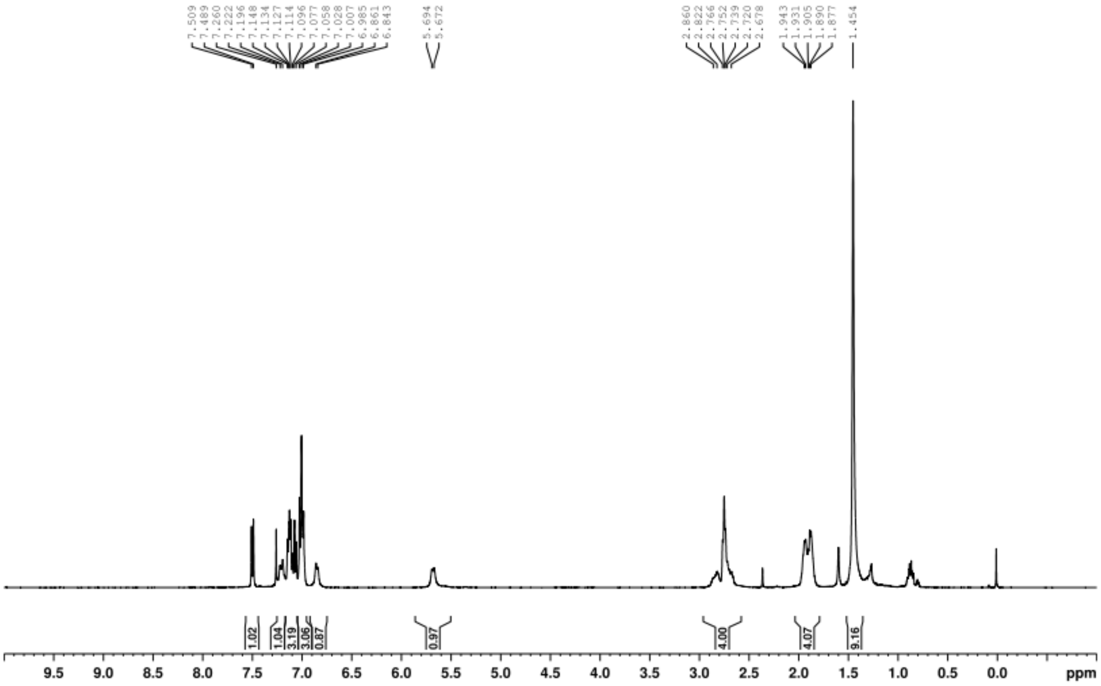

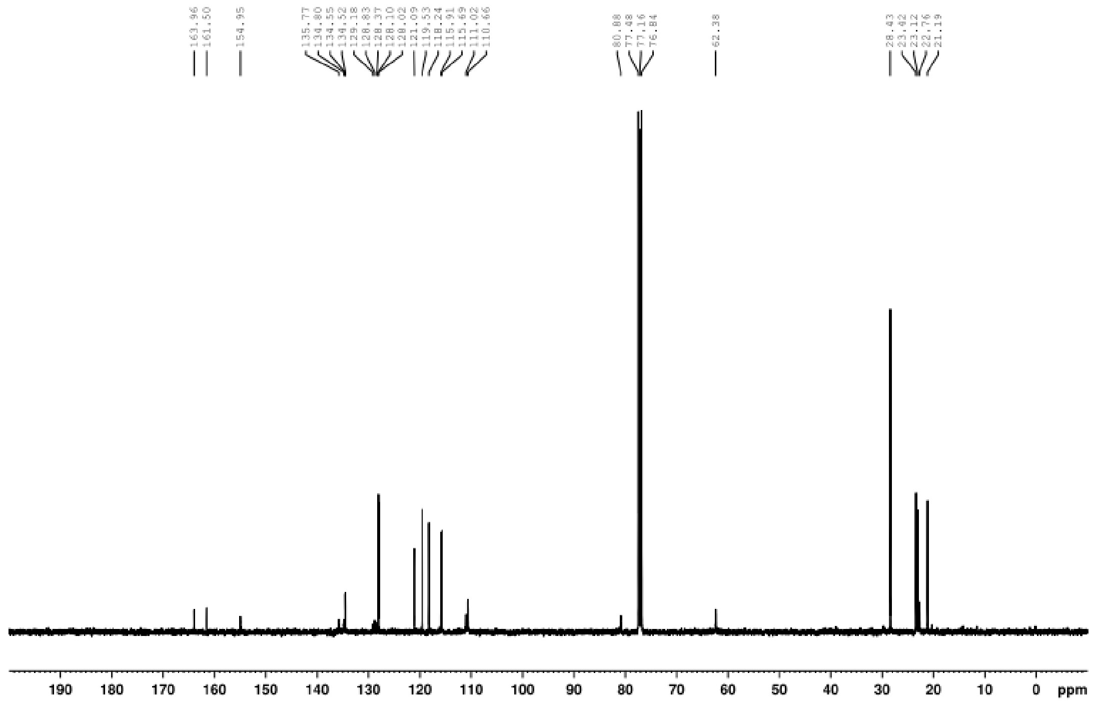

Supplementary Figure 80. ^{1}H and ^{13}C-NMR spectrum for **10c**

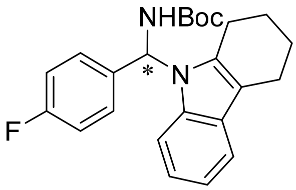

10c

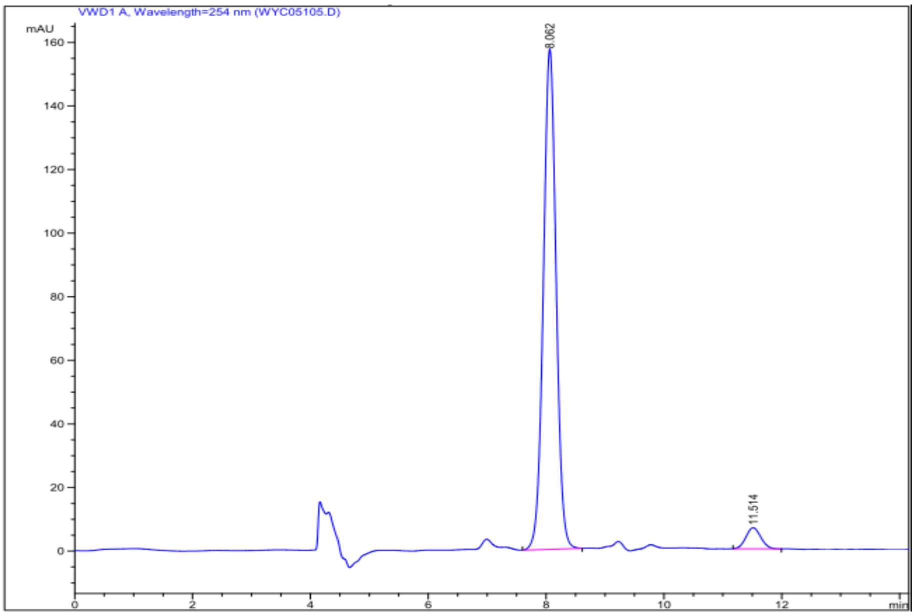

Peak #	RetTime [min]	Type	Width [min]	Area mAU *s	Height [mAU]	Area %
1	8.062	BB	0.2324	2368.33716	157.34300	95.3296
2	11.514	BP	0.2754	116.02862	6.63322	4.6704

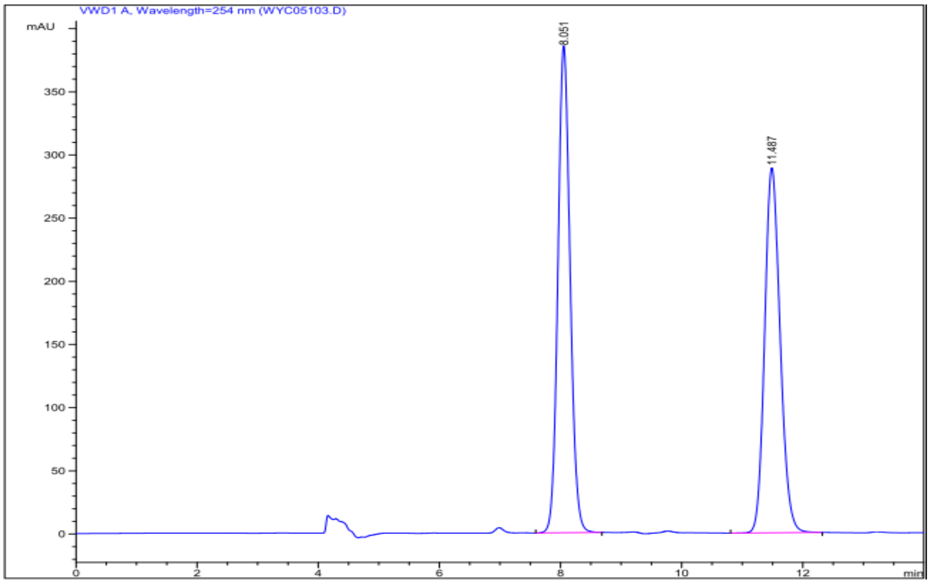

Peak #	RetTime [min]	Type	Width [min]	Area mAU *s	Height [mAU]	Area %
1	8.051	VB	0.2155	5387.45068	385.49136	51.1668
2	11.487	PB	0.2746	5141.74023	288.87119	48.8332

Supplementary Figure 81. HPLC spectrum for **10c**
